# Site-selective C–C modification of proteins at neutral pH using organocatalyst-mediated cross aldol ligations†
†Electronic supplementary information (ESI) available. See DOI: 10.1039/c8sc01617h


**DOI:** 10.1039/c8sc01617h

**Published:** 2018-05-31

**Authors:** Richard J. Spears, Robin L. Brabham, Darshita Budhadev, Tessa Keenan, Sophie McKenna, Julia Walton, James. A. Brannigan, A. Marek Brzozowski, Anthony J. Wilkinson, Michael Plevin, Martin A. Fascione

**Affiliations:** a Department of Chemistry , University of York , York , YO10 5DD , UK . Email: martin.fascione@york.ac.uk; b Department of Biology , University of York , York , YO10 5DD , UK

## Abstract

The bioconjugation of proteins with small molecules has proved an invaluable strategy for probing and perturbing biological mechanisms. The general use of chemical methods for protein functionalisation can be limited however by the requirement for complicated reaction partners to be present in large excess, and harsh conditions which are incompatible with many protein scaffolds. Herein we describe a site-selective organocatalyst-mediated protein aldol ligation (OPAL) that affords stable carbon–carbon linked bioconjugates at neutral pH. OPAL enables rapid modification of proteins using simple aldehyde probes in minimal excess, and is utilised here in the affinity tagging of proteins in cell lysate. Furthermore we demonstrate that the β-hydroxy aldehyde OPAL product can be functionalised again at neutral pH in a tandem organocatalyst-mediated oxime ligation. This tandem strategy is showcased in the ‘chemical mimicry’ of a previously inaccessible natural dual post-translationally modified protein integral to the pathogenesis of the neglected tropical disease Leishmaniasis.

## Abstract 2

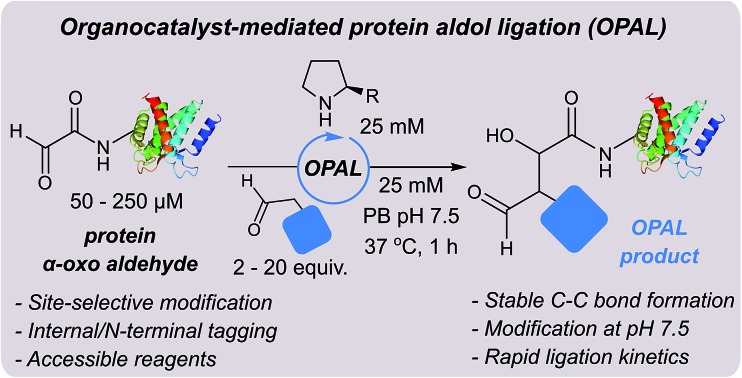
An organocatalyst-mediated protein aldol ligation (OPAL) affords C–C linked bioconjugates at neutral pH.

## Introduction

Protein-small molecule bioconjugates have revolutionised the fields of chemical medicine, chemical biology and cell biology,^1^–^4^ but their utility can be undermined by the instability of the covalent linkages generated by existing protein chemical modification strategies.^5^ Carbon–carbon bonds, the backbone of all organic molecules, are inherently stable across a range of conditions however, and are therefore established as the most coveted linkage in bioconjugation studies. Although a small number elegant strategies for the chemical assembly of protein C–C bonds, including carbon free-radical additions to alkenes,^6^,^7^ Knoevenagel^8^ or Mukaiyama^9^ based condensations, and Pictet–Spengler type ligations,^10^ in addition to other bioorthogonal ligations,^3^,^11^–^13^ have recently demonstrated impact beyond academic labs, the general use of some such methods can be hindered by practical limitations. These include the requirement for chemical probes containing reactive handles accessible only through multi-step syntheses, or probes themselves which are prohibitively expensive and used in large excess, or have reduced reactivity under biological conditions such as in the presence of oxygen. Of particular significance is the frequent requirement for acidic or basic pH during bioconjugation as the folding, stability and function of many proteins (and complexes) depends on the maintenance of a pH close to neutral. Deviations from this optimal pH window can therefore have deleterious effects^14^ exemplified by the disassembly of the histone octamer nucleosome core,^15^ human hemoglobin dissociation,^16^ aggregation of antibodies^17^ and protein aggregation events associated with neurodegenerative diseases such as Alzheimer's^18^ and Prion diseases.^19^ Indeed, acidic pH is a characteristic feature of the lysosome which facilitates the degradation of proteins. There is therefore a pressing need to develop fully biocompatible ligations that address these limitations and enable efficient C–C bioconjugation of proteins at neutral pH. Using affordable simple probes in ratios acceptable for small molecule chemistry would also allow wider access to the methodology in non-specialist labs or those with limited resources.

In comparison to bioconjugation chemistry the synthesis of C–C bonds in small molecule chemistry is well established, and in the 2000's was redefined by the emergence of ‘organocatalysts’^20^,^21^ capable of optimising existing transformations as well as inspiring new reactions.^22^,^23^ Our interest in this area was piqued by the prominent role aldehyde chemistry has played in the exponential development of the field.^24^ This is notable as aldehydes are chemical handles which are easily installed into proteins through modification of both natural^25^,^26^ and unnatural amino acids,^27^ with efficiencies akin to the installation of widely utilised protein modification tags such as dehydroalanine (from cysteine^7^,^28^ or phosphoserine^6^) and azides or alkynes.^29^ We therefore sought to explore whether the challenges of constructing C–C modified proteins could be overcome through exploitation of aldehyde handles, and the development of a novel ligation which merged established small molecule aldehyde organocatalysis methods with developing bioconjugation chemistry techniques.

**Fig. 1:**
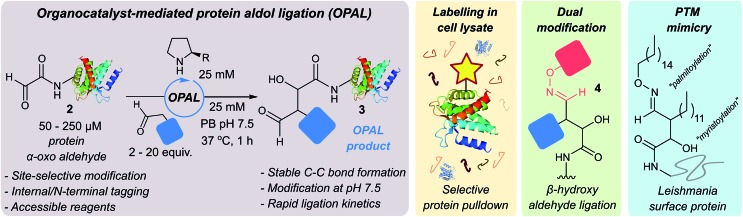
Schematic representation of organocatalyst-mediated protein aldol ligation (OPAL) and its application.

We were attracted to Northrup and Macmillan's seminal work on cross aldol reactions of aldehydes using l-proline **1** as an organocatalyst,^30^ and the water compatibility of this chemistry.^31^ We envisioned that an analogous cross aldol reaction on protein aldehydes might have widespread utility because it would enable site selective C–C modification of proteins using uncomplicated aldehyde probes and non-toxic affordable organocatalysts. As an additional benefit, we anticipated the β-hydroxy aldehyde product of the cross aldol reaction could be subjected to alternative aldehyde ligation conditions to afford dual modified proteins, which are of increasing utility.^32^ Herein we disclose the realisation of this strategy in an ‘organocatalyst-mediated protein aldol ligation’ (OPAL) that enables site-selective formation of stable C–C bonds to protein aldehydes **2** at neutral pH at internal sites within folded proteins, and at the N-terminus (Fig. 1, enamine activation mode). OPAL is a stand-alone protein bioconjugation reaction which is highly efficient and complete within 1 h using as few as 2 equivalents of simple aldehyde probe. Additionally the selectivity of the ligation is also demonstrated in complex mixtures through the affinity purification of a pH sensitive protein from a cell lysate. Furthermore, we establish that the β-hydroxy aldehyde OPAL product **3** can subsequently take part in an organocatalyst-mediated β-hydroxy oxime ligation unexpectedly accelerated at neutral over acidic pH, affording access to dual differentially modified proteins **4**. The utility of this tandem method is showcased in the ‘chemical mimicry’ of a dual post-translationally lipidated surface protein, integral to the pathogenesis of Leishmaniasis.^33^

## Results and discussion

### Feasibility studies on proteins

**Fig. 2:**
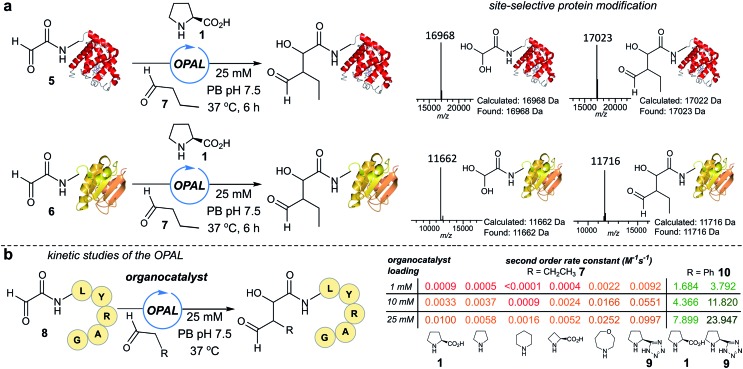
(a) Left: OPAL bioconjugations using butyraldehyde **7** as an aldol donor and myoglobin **5** and thioredoxin **6** as aldol acceptors. Right: Mass spectrum traces of hydrated protein aldehyde starting material, and products following OPAL. (b) Aldol organocatalyst screen using butyraldehyde **7** and phenylacetaldehyde **10** as donors, with observed second order rate constants for individual organocatalysts colour coded red (slowest) < pale orange < dark orange < light green < dark green (fastest).

To establish conditions for the OPAL we studied two model protein systems, haem co-factor containing horse heart myoglobin **5**, and disulfide bond containing thioredoxin **6**, both bearing non-enolisable N-terminal α-oxo aldehydes^34^,^35^ (for aldehyde installation methods see ESI Fig. 1†). Preliminary ligations in phosphate buffer (PB) afforded full conversion to the desired β-hydroxy protein aldehydes within 6 h at neutral pH with 100 mM l-proline organocatalyst **1**, using butyraldehyde **7** as an aldol donor (Fig. 2a). Importantly, only a single organocatalyst-dependent aldol modification occurred, confirming the expected stability of the β-hydroxy aldehyde motif to further aldol reactions.^36^ Additionally UV/Vis spectroscopic measurements of the haem group in the modified myoglobin demonstrated no compromise to the protein's tertiary structure had occurred (ESI Fig. 2†) and trypsin digest and LC-MS/MS analysis of the resulting peptide fragments confirmed the site-selective nature of OPAL (ESI Fig. 3†).

### Optimisation on peptides and proteins

Encouraged by the biological compatibility and site-selectivity of these preliminary ligations, attention next turned to optimising OPAL by focusing on the choice of organocatalyst and aldehyde donor species. We screened a panel of secondary amines to investigate their ability to catalyse the ligation using the well studied model peptide substrate α-oxo-aldehyde-LYRAG **8** and butyraldehyde **7**, and determined second order rate constants for each catalyst at 1, 10, and 25 mM loadings (Fig. 2b, see ESI Table 1,† ESI Fig. 4† for MS/MS analysis, and the ESI† page S35 for a discussion on stereochemistry). There was a 60-fold range in the magnitude of the rate constants across the panel with tetrazole **9** exhibiting the highest value.^37^,^38^ Similar reactivity correlations were also evident using a protein substrate (ESI Fig. 5†). Further peptide screens demonstrated that the nature of the α-carbon substituent of the aldehyde donor also significantly affected the rate of ligation, as in the presence of l-proline **1** and tetrazole **9**, donors such as phenylacetylaldehyde **10**, bearing an aryl substituent, reacted ∼240-fold faster than aldehyde donors bearing alkyl substituents. Overall, the rate constant of ∼24 M^–1^ s^–1^ using tetrazole **9** compares favorably with the fastest aldehyde bioconjugations reported in the literature at any pH,^24^,^39^ demonstrating that judicious choice of both organocatalyst and donor species is essential for achieving optimal rates of ligation.

### Site-selective functionalisation of proteins

**Fig. 3:**
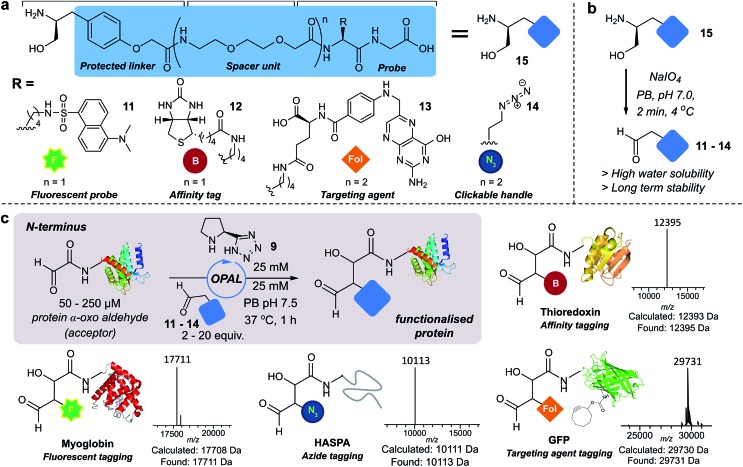
(a) OPAL probe precursors bearing functional tags. (b) Mild NaIO_4_ unmasking of α-aryl aldehyde probes. (c) OPAL functionalization of N-terminal α-oxo aldehyde containing proteins (50–250 μM), using probes **11–14** (0.5–1.5 mM).

Next we set out to explore the scope of these optimised conditions in the site-selective bioconjugation of a range of proteins using functionalised α-aryl aldehyde probes. Guided by a desire to simplify protein chemical modification procedures, we designed a practical synthetic route to access α-aryl substituted aldehyde donors bearing functional tags, including a fluorescent label **11**, a biotin affinity tag **12**, a folate targeting moiety **13**, and a bioorthogonal azide handle **14** (Fig. 3a). Probes bearing 1,2 amino-alcohols **15** were constructed using solid phase peptide synthesis (SPPS) from readily available building blocks, and unmasked using a biologically compatible periodate oxidation to reveal the desired aldehyde (Fig. 3b). These aldehyde probes were then deployed in the site-selective OPAL modification of a variety of α-oxo aldehyde containing proteins using 25 mM tetrazole **9** at neutral pH (Fig. 3c). Thioredoxin, myoglobin and hydrophilic acylated surface protein A (HASPA) from *Leishmania donovani*,^33^ all bearing α-oxo aldehydes at their N-termini, were modified in quantitative conversion within 1 h using 2–20 equivalents of aldehyde probe, with no modification observed on proteins that did not bear the required aldehyde functionality (see ESI† Section 6 for seven further protein bioconjugation examples). GFP protein bearing an α-oxo aldehyde in addition to a bioorthogonal strained alkyne (cyclooctyne-lysine) at position 39 was also compatible with the OPAL conditions and quantitatively modified with both folate (Fig. 3c), and biotin tags (see ESI† page S58). Additionally OPAL linkages showed no liability after incubation in 25 mM PB pH 7.5 over 72 hours at 37 °C (ESI Fig. 6†).

### Functionalisation of proteins at internal residues and in cell lysate

**Fig. 4:**
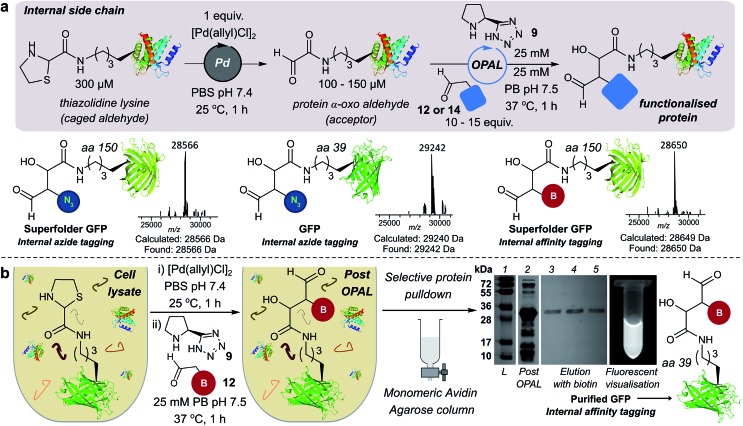
(a) Incorporation of internal α-oxo aldehydes through Pd-mediated decaging, and tandem OPAL functionalisation of proteins. ESI-MS traces of OPAL products following spectra deconvolution. (b) Pd-mediated decaging and OPAL biotinylation in cell lysate. SDS-PAGE lane 1: ladder; lane 2: cell lysate post OPAL; lanes 3, 4, 5: biotin elution.

We also demonstrated the compatibility of OPAL in bioconjugations at internal sites within folded green fluorescent protein (GFP) and superfolder GFP (sfGFP) (Fig. 4a, ESI Fig. 7†). α-Oxo aldehydes were first quantitatively installed into the proteins at neutral pH using a biocompatible Pd-mediated decaging (1 equivalent for 1 h)^27^ of an incorporated unnatural thiazolidine-lysine (ThzK) amino acid, recently developed in our lab. Both internal aldehydes were then functionalised using OPAL to install azide handles at position 39 of GFP and position 150 of sfGFP in 1 h using aldehyde probe **14** with no observable loss of fluorescence. Furthermore we demonstrated the compatibility of both the Pd-mediated decaging and OPAL modification in cell lysate (Fig. 4b, ESI Fig. 8–10† for uncropped gels). Following expression of the ThzK containing GFP in *E. coli*, the cell lysate was subjected to a 1 h Pd-mediated decaging followed by a further 1 h OPAL using biotin tag **12** at neutral pH. The lysate was then loaded onto an avidin affinity column, which was washed prior to elution with 2 mM biotin to afford the OPAL biotinylated GFP protein (see ESI† page S16 for protein recovery at each step). The ability to selectively pull-down only the α-oxo aldehyde containing GFP from the lysate in a 2 h procedure showcases the selectivity and efficiency of OPAL for the modification of an internal aldehyde in folded GFP within a mixture of proteins, and the retention of GFP fluorescence (which is quenched at mildly acidic pH (ref. 40)) also highlights the significant advantage of functionalisation at neutral pH.

### Optimising bi-functional modification

**Fig. 5:**
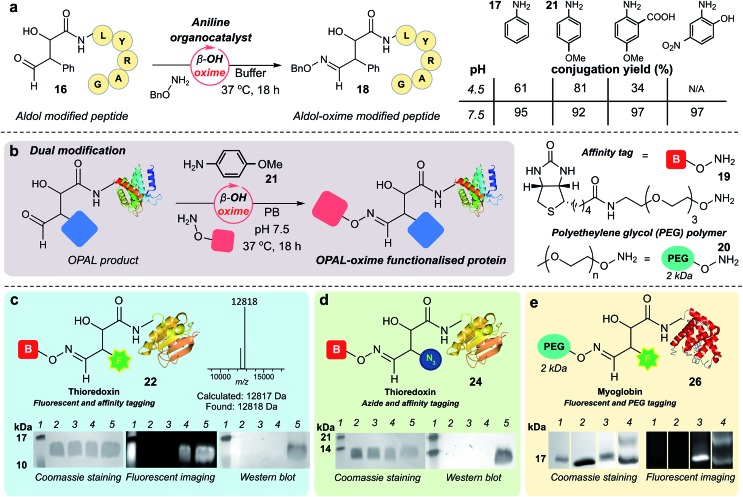
(a) pH dependence of β-hydroxy oxime ligation using aniline organocatalysts. (b) Schematic representation of organocatalyst-mediated oxime ligation of protein β-hydroxy aldehydes. (c) Product **22** of tandem organocatalyst-mediated bioconjugations of thioredoxin **6** and fluorescent OPAL product **23**, analysed by SDS-PAGE and Western blot. (d) Product **24** of tandem organocatalyst-mediated bioconjugation of thioredoxin **6** analysed by Western blot. Lane 1 in (c) and (d) = molecular weight ladder; lane 2 in (c) and (d) = wt thioredoxin, prior to α-oxo aldehyde installation; lane 3 in (c) and (d) = α-oxo thioredoxin **6**; lane 4 in (c) and (d) = thioredoxin OPAL products; lane 5 in (c) and (d) = thioredoxin OPAL-β-hydroxy oxime products. (e) Product **26** of tandem organocatalyst-mediated bioconjugations of myoglobin **5** analysed by SDS-PAGE. Lane 1 = molecular weight ladder; lane 2 = α-oxo myoglobin **5**; lane 3 = OPAL-myoglobin **25**; lane 4 = Top band: OPAL-β-hydroxy oxime myoglobin **26**; bottom band: presumed OPAL-myoglobin starting material **25** with non-covalent association of **20**. Conditions for tandem β-hydroxy oxime ligations: proteins 25–50 μM, 15 mM 19 or 20, 10 mM 21, PB (50 mM), pH 7.5, 37 °C, 18–42 h.

As previously observed by Macmillan in small molecule cross aldol reactions,^36^ the β-hydroxy aldehyde product of the OPAL displayed no reactivity in further aldol reactions.^41^ So we next determined whether this aldehyde was reactive under alternative conditions which would enable construction of challenging but coveted differentially bi-functionalised proteins (Fig. 5), which have limitless potential applications.^32^,^42^,^43^ Using peptide β-hydroxy aldehyde-LYRAG **16**, we screened the reactivity of the β-hydroxy aldehyde as an electrophilic partner in two high yielding literature bioconjugation reactions, the iso-Pictet–Spengler ligation^44^ and the 2-amino benzamidoxime (ABAO) ligation^45^ (both with optimal reactivity at acidic pH). Although conversion to bi-functionalised product was observed, yields were disappointingly low (ESI Fig. 11†), emphasising the relative stability of the β-hydroxy aldehyde moiety compared to other aldehydes previously used in bioconjugation studies.^25^ We therefore turned our attention to the classical acid-catalysed oxime ligation (pH 4.5 optimum), which proceeds more slowly at neutral pH but can be accelerated by the addition of aniline **17** as an organocatalyst.^46^ Gratifyingly, in studies using β-hydroxy aldehyde-LYRAG **16** and an aminooxy nucleophile at pH 4.5 we achieved 61% conversion to bi-functionalised product **18** in the presence of aniline organocatalyst **17**. Unexpectedly however, the conversion to the β-hydroxy oxime product **18** was further increased to 95% when the reaction was performed at pH 7.5 (Fig. 5a, aniline Schiff base activation mode), which is a reversal of the precedent for oxime formation with other aldehyde handles.^46^ This trend was again evident when screening alternative aniline organocatalysts which have been previously reported for hydrazone/oxime ligation,^47^ as well as with alternative peptide and protein β-hydroxy aldehyde substrates (ESI Fig. 12†), suggesting a general trend in reactivity and improved biological compatibility. Akin to observations made for imine formation at *ortho*-substituted aromatic aldehydes in water,^48^ this reversal may be governed by intramolecular H-bonding between the β-hydroxy group and the protonated aldehyde or aniline Schiff base intermediates formed *en route* to oxime formation, and merits further investigation.

### Bi-functional modification of proteins through β-hydroxy oxime ligations

Having established an unexpected reversal in pH dependence for the rate of oxime formation we subsequently demonstrated the potential utility of the organocatalyst-mediated β-hydroxy-oxime ligation in tandem with OPAL for the construction of dual N-terminal functionalised proteins using both a biotin aminooxy affinity handle **19**, and a polyethylene glycol (PEG) aminooxy reagent **20** (Fig. 5b). A two-step organocatalyst-mediated tandem modification of thioredoxin **6** was achieved at neutral pH, firstly using OPAL to quantitatively install a fluorescent label into the protein using 25 mM tetrazole **9** and probe **11**, followed by tandem oxime ligation under optimised reaction conditions of 10 mM *p*-anisidine **21** organocatalyst in PB (50 mM, pH 7.5) and biotin affinity handle **19**, to afford differentially functionalised thioredoxin **22** (Fig. 5c, ESI Fig. 13† for uncropped gels). SDS-PAGE and Western blot (anti-biotin-alkaline phosphatase antibody detection) analysis of the unmodified α-oxo aldehyde thioredoxin **6** (lane 3), fluorescent OPAL product **23** (lane 4), and the bi-functional construct **22** (lane 5), demonstrated that the protein could be detected by fluorescence only after OPAL modification with aldehyde probe **11** (lane 4), and by fluorescence and Western blotting only after tandem oxime formation using biotin **19** (lane 5). Under identical organocatalyst conditions thioredoxin could also modified with both an azide and biotin probe with detection of the bi-functionalised product **24** by Western blot (Fig. 5d, ESI Fig. 14†) as well as myoglobin with both azide and biotin (ESI† page S67). Fluorescent myoglobin OPAL product **25** was also further functionalised by PEGylation (Fig. 5e, ESI Fig. 15†), a strategy used for increasing the circulatory lifetime of therapeutic proteins. Following treatment with aminooxy probe **20**, PEGylated fluorescent protein **26** (lane 4) was characterised using SDS-PAGE by an increase in molecular weight in over both unmodified **5** (lane 2) and fluorescent myoglobin **25** (lane 3). Additionally, we noted the β-hydroxy oxime linkages resulting from tandem bioconjugation reactions, showed no degradation over 30 days at neutral pH (ESI Fig. 16†).

### Towards chemical mimicry of natural dual PTMs of HASPA

**Fig. 6:**
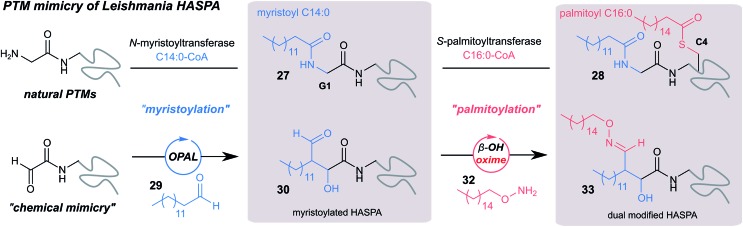
Schematic representation of native dual PTM of Leishmania HASPA (top), and proposed ‘chemical mimicry’ using organocatalyst-mediated tandem bioconjugation (bottom).

Finally, we sought to explore the suitability of the tandem OPAL-oxime ligation for the ‘chemical mimicry’ of a natural dual post-translational modification (PTM) integral to the pathogenesis of the neglected tropical disease Leishmaniasis. Hydrophilic acylated surface proteins (HASPs) are present in all human infective *Leishmania* parasites. They are highly immunogenic and form the basis of a visceral leishmaniasis vaccine currently undergoing clinical trials in humans.^49^ Their expression is stage regulated during human infection, however the exact role they play in the parasite lifecycle has yet to be determined.^33^ Non-classically, HASPA is dually acylated at the N-terminus (Fig. 6, top) with both myristoyl **27** (at Gly1) and palmitoyl **28** (at Cys4) lipids, PTMs which are thought to govern its ability to associate with plasma membranes^50^ but not fully rationalised. Whilst co-translational myristoylation of HASPA by the parasite *N*-myristoyl transferase (NMT) can be recapitulated *in vitro*, the *S*-palmitoyltransferase is unknown and the Cys4 containing protein is prone to precipitation, which has limited recombinant access to natural dual lipidated protein for further study. We therefore designed a strategy using our tandem biocompatible ligations to chemically mimic the structural modifications of HASPA for the first time (Fig. 6, bottom), and provide access to a dual lipidated construct.

**Fig. 7:**
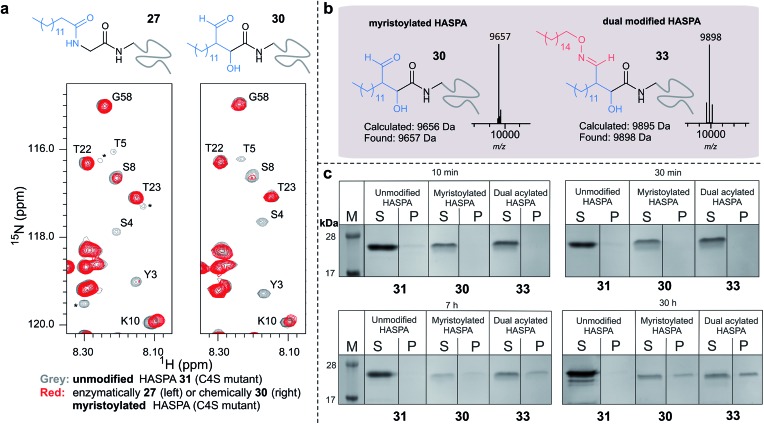
(a) Regions of 2D (^1^H, ^15^N) HSQC spectra of [^15^N] labelled HASPA (C4S mutant). Comparison of unmodified **31** (grey) and enzymatically myristoylated (red) HASPA **27** (left). Comparison of unmodified **31** (grey) and chemically myristoylated (red) HASPA **30** (right). (^1^H, ^15^N) Resonance assignments are indicated. Unassigned peaks are denoted by asterisks. (b) ESI-MS characterisation following spectra deconvolution of chemically myristoylated HASPA **30** (left), and dual modified HASPA mimic **33** (right), note only peaks for **30** and **33** are visible, in the magnified spectra on page S83 aldol condensation product with loss of H_2_O is also visible. Notably in all OPAL reactions performed, aldol condensation product is only observed in this example. (c) SDS-PAGE analysis of liposome sedimentation assays over 30 h. 20 μg unmodified HASPA **31** (left panel), OPAL myristoylated HASPA **30** (central panel, myristoylated), and dual modified chemical mimic **33** HASPA (right panel, dual acylated), were added to a suspension of PC:Ch liposomes and dialysed in the presence of detergent and then sedimented. The supernatant (S) and resuspended liposome pellet (P) were analyzed by SDS-PAGE (M = molecular weight ladder). See ESI Fig. 19–22† for associated preparation of liposomes, negative controls in the absence of protein or liposome, and SDS-PAGE analysis.

Using myristoyl aldehyde **29** as donor and recombinantly expressed *Leishmania donovani* HASPA bearing an N-terminal α-oxo aldehyde as a substrate (C4S mutant, see ESI Fig. 17†) we firstly used OPAL to construct a chemical mimic **30** of the natural myristoylated protein at neutral pH in quantitative conversion (ESI-MS characterisation, Fig. 7b). To establish that this bioconjugate structurally mimics myristoylation we used NMR spectroscopy to characterise both the unmodified HASPA **31** and enzymatically myristoylated HASPA **27**, lipidated using purified recombinant *N*-myristoyl transferase.^51^ Following resonance assignment of backbone nuclei (ESI Fig. 18†), comparison of the 2D (^1^H, ^15^N) HSQC spectra revealed that myristoylation of the native protein caused exchange broadening of resonances for residues near the N-terminus, including Y3, S4, T5 and S8 (Fig. 7a, left panel). Subsequent NMR characterisation of the chemically myristoylated HASPA **30** was also then performed and the HSQC spectra revealed to be highly comparable to that of the enzymatically modified protein **27** (Fig. 7a, right panel), with residues near the N-terminus also displaying the characteristic exchange broadening following modification. These data therefore demonstrate that OPAL modification of HASPA replicates the *in vitro* solution properties and structure of the enzymatically modified protein, and also highlight the simplicity of the OPAL procedure for use in the chemical mimicry of protein myristoylation, which in this example requires only three affordable commercial reagents, sodium periodate for aldehyde installation (see ESI Fig. 1†), and aldehyde **29** and organocatalyst **9** for the OPAL.

To begin to investigate the potential function of *in vivo* dual modification of HASPs, we next subjected the OPAL myristoylated HASPA **30** to tandem organocatalyst-mediated β-hydroxy oxime ligation using palmitoyl aminoxy **32**. The dual modified product **33**, a chemical mimic of myristoylation and palmitoylation, was also characterised by ESI-MS (Fig. 7b). Finally both the OPAL myristoylated HASPA **30** and this dual modified HASPA **33** were characterised using time course liposome sedimentation assays to assess their capacity to bind to model biological membranes (Fig. 7c). SDS-PAGE analysis revealed that over a 30 h period both lipid modified HASPs displayed substantially greater levels of *in vitro* association to membranes than the unmodified HASPA **31** (Fig. 7c) and approximately twice as much protein was bound to the membrane when HASPA was dual lipidated in **33** compared to mono myristoylation in **30** (ESI Fig. S22†). Results from this *in vitro* ‘chemical model system’ therefore substantiate the notion that the *in vivo* role of the lipid PTMs is to facilitate attachment of HASPA to parasite membranes and determine its localisation to the cell surface,^50^ and emphasise that two lipid modifications may be required to maximise *in vivo* binding, with the caveat that further in-depth functional comparison of single and dual lipidated HASPs *in cellulo* is still warranted.

## Conclusion

In conclusion, we have validated the OPAL as a powerful stand-alone C–C forming bioconjugation strategy using simple aldehyde probes for the mild site-selective modification of a range of proteins at both internal and N-terminal sites demonstrating flexibility in both positioning and functionalisation. We also demonstrated the compatibility and selectivity of both Pd-mediated decaging of internal α-oxo aldehydes and subsequent OPAL modification in a mixture of proteins within cell lysate to enable affinity pull-down of GFP, which maintains its fluorescence as a consequence of purification at neutral pH. We anticipate the potential applicability and simplicity of this strategy may also serve to nucleate further organocatalyst-mediated protein modification studies.

In contrast to other protein bioconjugations, OPAL not only ‘survives’ but ‘thrives’ in biocompatible conditions. This is because at aqueous neutral pH the β-hydroxy aldehyde product of the OPAL predominates over the enal (aldol condensation) product. The β-hydroxy group of this newly installed aldehyde prevents further aldol reactions facilitating a single site-selective modification and in an additional benefit serves as a “pH switch” for a novel tandem β-hydroxy oxime ligation which is unexpectedly accelerated at neutral pH over acidic pH. Oxime ligations are perhaps the most widely used protein bioconjugation reaction,^47^ but limited in that they are most efficient at acidic pH even when using aniline organocatalysts. The presence of the β-hydroxy group seemingly alters this acidic bias and constitutes a new aldehyde scaffold for bioconjugations, which has an in-built preference to react, and is stable at neutral pH.

Finally we utilised OPAL in the chemical mimicry of *N*-terminal myristoylation of a HASP protein integral to the pathogenesis of Leishmaniasis and demonstrated the ability to mimic the structural effects of enzymatic myristoylation through characterisation by protein NMR. Subsequent chemical palmitoylation by a tandem β-hydroxy oxime ligation generated a construct that also mimics the previously inaccessible natural dual modified protein, and furthermore was used for exploring the effects of single *vs.* dual lipid modification on binding to membranes. Notably this strategy may lend itself to the *in vitro* study of other proteins bearing multiple post-translational lipid modifications^52^ and may also prove broadly applicable in the functionalisation and immobilisation of other biomolecules.

## Author contributions

R. J. S. performed protein and peptide bioconjugations; D. B., R. J. S., and R. B. performed chemical synthesis; T. K., R. J. S., R. B., S. M., and J. W. prepared and characterised proteins, J. A. B. constructed plasmids; S. M. and M. J. P. performed protein NMR experiments. A. M. B., A. J. W., M. J. P., and M. A. F. supervised the project, and R. J. S., M. P., A. J. W. and M. A. F. wrote the paper and designed the study. All authors analysed the data and commented on the paper.

## Additional information

All data reported are available in ESI,† and also archived in the University of York research database; accession DOI: 10.15124/487666f2-cf0f-46ca-9255-b54d3ace43ff.

## Conflicts of interest

M. A. F., R. J. S., R. L. B., D. B., and T. K. are authors on a patent PCT/GB2017/052896 filed by the University of York that covers Pd-mediated decaging of α-oxo aldehydes, OPAL modification of protein α-oxo aldehydes, and oxime ligation of β-hydroxy aldehydes.

## Supplementary Material

Supplementary information
