# Supplementary material for: Site-selective C–C modification of proteins at neutral pH using organocatalyst-mediated cross aldol ligations
Source: Chem Sci. 2018 May 31;9(25):5585–93. doi: 10.1039/c8sc01617h (PMC6049525; doi:10.1039/c8sc01617h)
Supplement: Supplementary file 1 [file SC-009-C8SC01617H-s001.pdf]

Electronic Supplementary Material (ESI) for Chemical Science.
This journal is © The Royal Society of Chemistry 2018

Supplementary Information for

Site-selective C-C modification of proteins at neutral pH using organocatalyst-mediated cross aldol ligations

Authors: Richard J. Spears,[1] Robin L. Brabham,[1] Darshita Budhadev,[1] Tessa Keenan,[1] Sophie McKenna,[1] Julia Walton,[1] Jim. A. Brannigan,[1] A. Marek Brzozowski,[1] Anthony J. Wilkinson,[1] Michael Plevin,[2] Martin A. Fascione[1]*

[1]Department of Chemistry, University of York, York, YO10 5DD, UK.

[2]Department of Biology, University of York, York, YO10 5DD, UK.

*Correspondence to: martin.fascione@york.ac.uk

Table of contents

1. General procedures and materials

2. Main text supplementary figures and tables

3. Synthesis of small molecules

4. Solid Phase Peptide synthesis (SPPS) and donor synthesis

5. Protein expression and purification

6. Peptide and protein chemical modifications

7. Mass spectrometry data of modified peptides

8. Mass spectrometry data of proteins and modified proteins

9. Tandem mass spectrometry data of aldol-oxime modified peptide

10. Kinetic data for OPAL

11. NMR data

12. References

1. General procedures and materials

Unless otherwise specified, all chemical reagents were obtained from commercial sources and used without further purification. Cyclooctyne-Lysine was purchased from Sirius Fine Chemicals SiChem GmbH. Chromatography solvents were used without distillation. Thin layer chromatography was carried out on Merck silica gel 60F254 pre-coated aluminium foil sheets and were visualised using UV light (254 nm) and stained with ninhydrin stain/ acidic ethanolic *p*-anisaldehyde stain. Flash column chromatography was carried out using slurry packed Fluka silica gel (SiO2), 35–70 μm, 60 Å, under a light positive pressure, eluting with the specified solvent system. Myoglobin from equine heart (M1882) and Thioredoxin from *Escherichia coli* (T0910) were purchased from Sigma and used without further purification. Sequencing Grade Modified Trypsin V5111 was purchased from Promega. Pierce™ Monomeric Avidin Agarose was purchased from ThermoFisher Scientific.

Small molecule NMR spectroscopy

^{1}H and ^{13}C NMR spectra were measured using either Jeol 400-MR or Bruker 500-MR spectrometer at The University York Centre for Magnetic Resonance at 400/500 MHz for ^{1}H and 100/125 MHz for ^{13}C with Me_4Si as the internal standard. Multiplicities are given as singlet (s), broad singlet (br s), doublet (d), doublet of doublets (dd), triplet (t), quartet (q), pentet (p) or multiplet (m). Resonances were assigned using HH-COSY and CH-HSQC. All NMR chemical shifts (δ) were recorded in ppm and coupling constants (J) are reported in Hz. TopSpin 3.5pl7 and MestReNova were primarily used for processing the spectral data.

HASPA protein NMR spectroscopy

^{1}H, ^{15}N backbone resonance assignments of HASPA were obtained from standard analysis of 3D HNCACB and HN(CO)CACB spectra. Data were collected using a 0.9 mM sample of [^{13}C,^{15}N] labeled HASPA in 20 mM HEPES pH 6.5, 50 mM NaCl. Additional assignment information was obtained with [^{13}C,^{15}N] labeled HASPA samples prepared with selective unlabelling of: (a) lysine; (b) arginine; and (c) leucine and valine residues[1]. ^{1}H and ^{15}N assignments of myristoylated HASPA were confirmed by analysis of a 3D ^{15}N-TOCSY HSQC spectrum. ^{1}H and ^{15}N assignments of 2D (^{1}H,^{15}N) HSQC spectra of G1S HASPA and chemically myristoylated-G1S HASPA were established by comparison to spectra of unmodified HASPA. All NMR spectra were recorded using pulse sequences from the Bruker library on a Bruker Avance II 700 MHz spectrometer equipped with a triple-resonance room temperature probe. Data were processed with Bruker TopSpin 2.0, NMRpipe and CCPN Analysis v2.

FTIR and Optical rotation analysis

Fourier transform infrared (FTIR) spectra were recorded on a PerkinElmer UATR 2 spectrometer by attenuated total reflectance (ATR) technique. Optical rotations were measured using Jasco Dip-370 digital polarimeter equipped with a sodium vapor lamp. Concentration is denoted as c and was calculated as grams per milliliters (g / 100 mL) whereas the solvent was indicated in parentheses (c, solvent).

UV/Vis analysis

UV/Vis analysis was performed using either a U1900 spectrometer (HITACHI) in line with UV solutions 2.2 software (HITACHI), or using a DS-11 FX+ spectrophotometer/fluoremter (DeNovix) in line with DS-11/DS-11 FX Software (Version 3.15).

High performance liquid chromatography instrumentation

Analytical HPLC was performed using either an Accucore C18 coulmn 2.6 µm column, 2.1 × 150 mm, or a Phenomenex Kinetex 5µ phenyl-hexyl 100 A column of dimension 250 × 4.6 mm. Shimadzu Prominence LC-20AD pump and SDP-M20A Diode Array detector were used during the analysis.

Liquid Chromatography Mass Spectrometry instrumentation

High Performance Liquid Chromatography-Electrospray Ionisation Mass Spectrometry (LC-MS) was accomplished using a Dionex UltiMate® 3000 LC system (ThermoScientific) equipped with an UltiMate® 3000 Diode Array Detector (probing 250-400 nm) in line with a Bruker HCTultra ETD II system (Bruker Daltonics), using Chromeleon® 6.80 SR12 software (ThermoScientific), Compass 1.3 for esquire HCT Build 581.3, esquireControl version 6.2, Build 62.24 software (Bruker Daltonics), and Bruker compass HyStar 3.2-SR2, HyStar version 3.2, Build 44 software (Bruker Daltonics) at The University York Centre of Excellence in Mass Spectrometry (CoEMS). All mass spectrometry was conducted in positive ion mode unless stated otherwise. Data analysis was performed using ESI Compass 1.3 DataAnalysis, Version 4.1 software (Bruker Daltonics).

LC-MS analysis of peptide and protein ligations

Prior to analysis by LC-MS, peptide or protein ligation mixture was diluted 1:3 in water and then further diluted 1:1 in acetonitrile with 1 % (v/v) formic acid. Peptide samples were analysed using an Accucore™ C18 2.6 µm column (50 x 2.1 mm) (ThermoScientific). Water with 0.1 % (v/v) formic acid (solvent A) and acetonitrile with 0.1 % (v/v) formic acid (solvent B) were used as the mobile phase at a flow rate of 0.3 mL/min at room temperature (RT). A multi-step gradient of 6.5 min was programmed as follows: 90% A for 0.5 min, followed by a linear gradient to 95% B over 3.5 min, followed by 95% B for an additional 0.5 min. A linear gradient to 95% A was used to re-equilibrate the column Under these conditions all peptides typically eluted between 2-5 min. Protein samples were analysed without the use of a column at RT. Water with 0.1 % (v/v) formic acid (solvent A) and acetonitrile with 0.1 % (v/v) formic acid (solvent B) were used as the mobile phase at a 1:1 ratio over the course of 3 min as follows: 0.05 mL/min to 0.25 mL/min for 1 min, 0.025 mL/min for 1 min, followed by 1.0 mL/min for 1 min. Under these conditions, all proteins typically eluted between 0.1-1.5 min.

Green fluorescent protein and Superfolder green fluorescent protein Mass Sepectrometry instrumentation

Electrospray ionisation mass spectrometry (ESI-MS) of samples relating to green fluorescent protein (GFP) or superfolder green fluorescent protein (sfGFP) were obtained using a solariX XR FTMS 9.4T (Bruker) using ftms Control, ftmsControl 2.1.0 Build: 98 software (Bruker Daltonics) at The University York Centre of Excellence in Mass Spectrometry (CoEMS). All

mass spectrometry was conducted in positive ion mode unless stated otherwise. Data analysis was performed using ESI Compass 1.3 DataAnalysis Version 4.1 software.

Green fluorescent protein and Superfolder green fluorescent protein Mass Sepectrometry analysis

Prior to analysis, samples containing GFP or sfGFP were desalted using either a PD SpinTrap G25 column (GE Healthcare Life Sciences), or using PD MiniTrap G25 column (GE Healthcare Life Sciences), eluting with water. The desalted protein sample (50 μL) was then diluted by addition of 50 μL of a 1:1 solution of water:acetonitrile 1% (v/v) formic acid for analysis.

Analysis of trypsin digest samples

Tryptic digestion samples were analysed using a Symmetry® C18 5μm 3.0 x 150 mm reverse-phase column (Waters). Water with 0.1% (v/v) formic acid (solvent A), and acetonitrile with 0.1% (v/v) formic acid (solvent B) were used as the mobile phase at a flow rate of 0.08 ml/min at 40 °C. A multi-step gradient of 45 min was programmed as follows: 95% A for 0.1 min, followed by a linear gradient to 80% B over 40 min, followed by a linear gradient to 95% B for 1 min, followed by a linear gradient to 95% A for 4 min.

Analysis of conjugation yields v1

Conversion from the designated starting material to the desired material (conjugation yields, %) was calculated by analysing the peak intensities of starting material and product species, and using Equation 1:

$$\frac{Product\ peak\ intensity}{Starting\ material\ peak\ intensities + Product\ peak\ intensity}\ x\ 100 = \%\ conversion \qquad \text{Equ. 1}$$

Analysis of conjugation yields v2

Conversion from the designated starting material to the desired material (conjugation yields, %) was calculated by analysing the peak area of the starting material and product species, and using Equation 2:

$$\frac{Peak\ area\ of\ product}{Peak\ area\ of\ starting\ material + Peak\ area\ of\ product}\ x\ 100 = \%\ conversion \qquad \text{Equ. 2}$$

Kinetic studies

Kinetic data was obtained from reactions performed on a 20 μL scale using an adapted LC-MS method.[2] Reactions were quenched by addition of 80 μL 1:1 H_2O:MeCN (1% formic acid) at time points 2 min, 5 min, 10 min, 20 min, and 30 min, and then analysed by LC-MS. Reaction yields at each time point were calculated and the second-order rate constants (k_2) were determined by fitting the data to the following equation 3:

$$y = \frac{\left(ln\frac{[A]_0[D]_t}{[A]_t[D]_0}\right)}{([D_0]-[A]_0)} = k_2 t \quad \text{Equ. 3}$$

where $[A]_0$ and $[D]_0$ are the initial concentrations of the acceptor (peptide aldehyde) and donor (small molecule aldehyde) respectively, and $[A]_t$ and $[D]_t$ are the concentrations of the acceptor and donor at time *t*.

Sodium dodecyl sulfate polyacrylamide gel electrophoresis (SDS-PAGE) analysis

For expression, purification, site-selective modification experiments, and liposome assay experiments relating to hydrophilic surface acylated protein A (HASPA), green fluorescent protein (GFP) and superfolder green fluorescent protein (sfGFP), all SDS-PAGE analysis was performed using 12% or 4-20% gradient polyacrylamide gels[3]. For experiments relating to dually modified proteins, all SDS-PAGE analysis was performed using 15% acrylamide gels. Samples were reduced by boiling for 5-10min (2% SDS, 2mM 2-mercaptoethanol, 4% glycerol, 40mM Tris-HCl pH 6.8, 0.01% bromophenol blue). Molecular weight markers used were either PageRuler Plus Prestained Protein Ladder (ThermoScientific) or SDS-PAGE Molecular Weight Standards, Low Range (Bio-Rad). Each gel was run at 200 volts for 45-80 min.

Coomassie stain

For Coomassie stain experiments, the gel was washed with fixing solution (40% MeOH, 10% AcOH), stained with 0.1% Coomassie Brilliant Blue R-250 (50% MeOH, 10% AcOH), and finally destained with solution (50% MeOH, 10% AcOH). Images of the resulting gels were captured and analysed using a Syngene G:BOX Chemi XRQ equipped with a Synoptics 4.0 MP camera, with GeneSys software (Version 1.5.7.0).

Fluorescent imaging

For fluorescent imaging of fluorescently modified proteins, the SDS PAGE gel was washed with fixing solution (40% MeOH, 10% AcOH). Visualisation of protein fluorescence, and images of the resulting gels, were captured using a Syngene G:BOX Chemi XRQ equipped with a Synoptics 4.0 MP camera in line with GeneSys software (Version 1.5.7.0).

Western Blot analysis

For western blot analysis of biotinylated protein samples (12 µg) were run on 15% SDS–PAGE and transferred onto a nitrocellulose membrane filter (0.45µm, Amersham Protran Sandwich, GE Healthcare) using an electroblot apparatus (Bio-Rad, Hercules, CA) at 100V, 350mA for 1h in cooled transfer buffer (25 mM Tris–HCl pH 8.3, 192 mM glycine, 0.1% SDS, 20% (v/v) methanol). The membrane was incubated in blocking solution (Phosphate buffered saline (PBS) tablets, Sigma)) containing 5% non-fat dry milk powder for 16 hours at 4 °C. The membrane was processed through sequential incubations with primary antibody, alkaline phosphatase anti-biotin (goat, Vector Labs, CA) 1:1000 dilution in PBS for 1 hour at room temperature, followed by washing in PBS, 0.01% Tween-20, and then incubation with visualising substrate BCIP/NBT Alkaline Phosphatase Substrate Kit (Vector Labs, CA) until immunoreactive proteins on the membrane were visible. The reaction was stopped by

washing the membrane in distilled water. The membranes were imaged using a Syngene G:BOX Chemi XRQ equipped with a Synoptics 4.0 MP camera, with GeneSys software (Version 1.5.7.0).

Procedure for trypsin digestion

A 100 µL solution of OPAL product with a total protein content of 1 mg was prepared. The product was analysed by MS before being subjected to the trypsin digest.

The 100 µL solution of OPAL product was dialysed into 50 mM Tris-HCl, pH 8.0. 36 mgs of solid urea was then added to the solution, giving a final concentration of 6M Urea. To this solution was added DTT (5 µL of a 200 mM solution in 50 mM Tris-HCl, pH 8.0). The mixture was allowed to stand at room temperature for 1 h. The solution was then charged with iodoacetamide (20 µL of a 200 mM solution in 50 mM Tris-HCl, pH 8.0), gently vortexted, and allowed to stand at room temperature in the dark for 1 h. DTT (20 µL of a 200 mM solution in 50 mM Tris-HCl, pH 8.0) was then added to consume any unreacted iodoacetamide, and the solution was allowed to stand at room temperature in the dark for 1 h. 775 µL of a 50 mM Tris-HCl, 1 mM $CaCl_2$ (pH 7.6) was then added to reduce the urea concentration to >0.6 M. Trypsin solution (0.2 µg/µL, 100 µL in resuspension buffer, 50 mM acetic acid) was then added. The reaction mixture was gently vortexted and incubated for 16 h at 37 ^{o}C.

(NB: As much of this procedure as possible was performed in a laminar flowhood)

To stop the trypsin digest procedure, 1 µL of formic acid was added to bring the pH of the solution to pH 3-4 (checked by pH paper). A 50 µL aliquot of this solution was analysed directly by LC-MS. The remaining solution was stored in a -20 ^{o}C freezer and defrosted if more samples were required.

2. Main text supplementary figures and tables

Overview of α-oxo aldehyde installation into proteins

a) ***Periodate oxidation (for proteins such as Thioredoxin)***

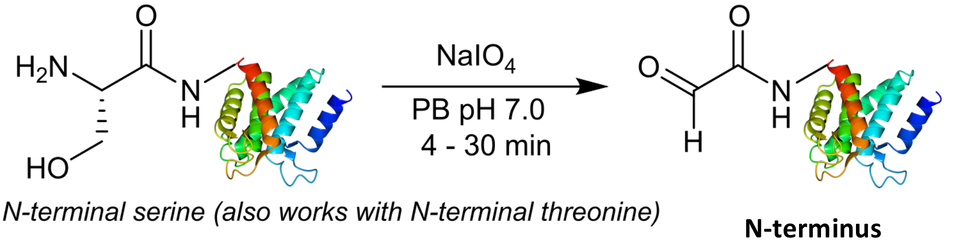

b) ***Biomimetic transamination (for proteins such as Myoglobin)***

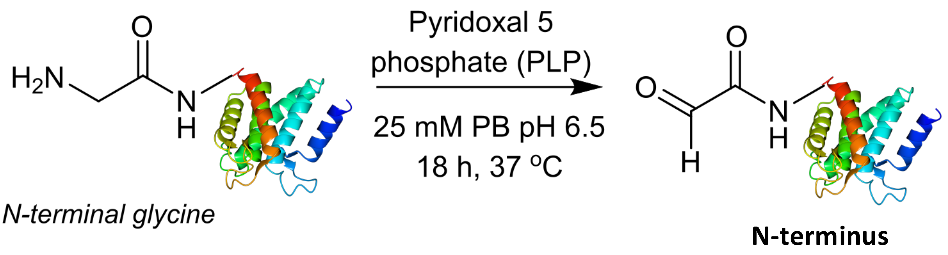

c) ***Thiazolidine decaging***

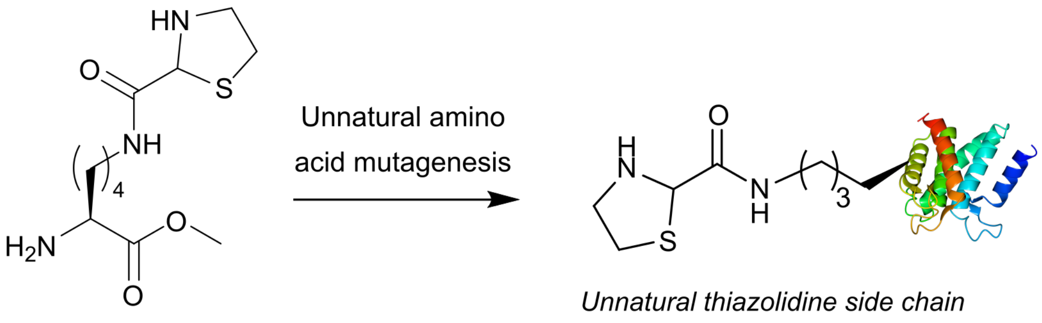

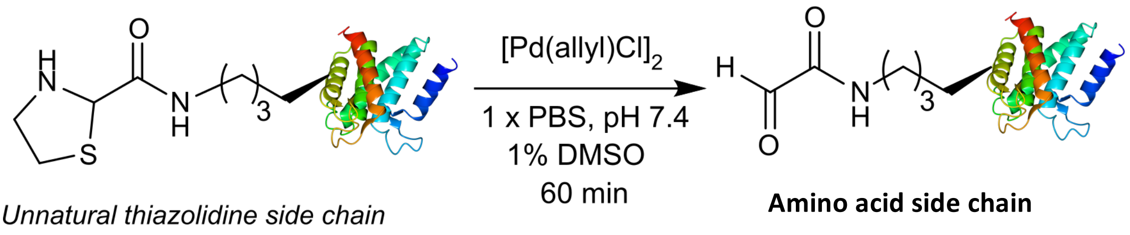

Supplementary Figure 1: Different methods of installing α-oxo aldehydes into proteins. a) Oxidation of N-terminal serine (or threonine) using sodium periodate. b) Biomimetic transamination of N-terminal glycine using pyridoxal-5-phosphate (PLP). c) Incorporation of thiazolidine lysine into a protein via unnatural amino acid mutagenesis, followed by subsequent palladium-mediated ‘decaging’ to reveal the side chain α-oxo aldehyde

UV/Vis data for α-ethyl-β-hydroxy aldehyde myoglobin

A control sample of myoglobin **S1** was prepared by dissolving lyophilised myoglobin in 25 mM PB pH 7.5, and a sample of α-ethyl-β-hydroxy aldehyde myoglobin **S2** was prepared as described previously (desalted using a PD SpinTrap G25 desalting column (GE Healthcare Life Sciences), eluting into 25 mM PB pH 7.5). UV-Vis measurements were obtained for unmodified myoglobin (without desalting), and aldol myoglobin. Based on the absorbance at 410 nm that is characteristic of the myoglobin heme group, the protein structure is retained post modification.

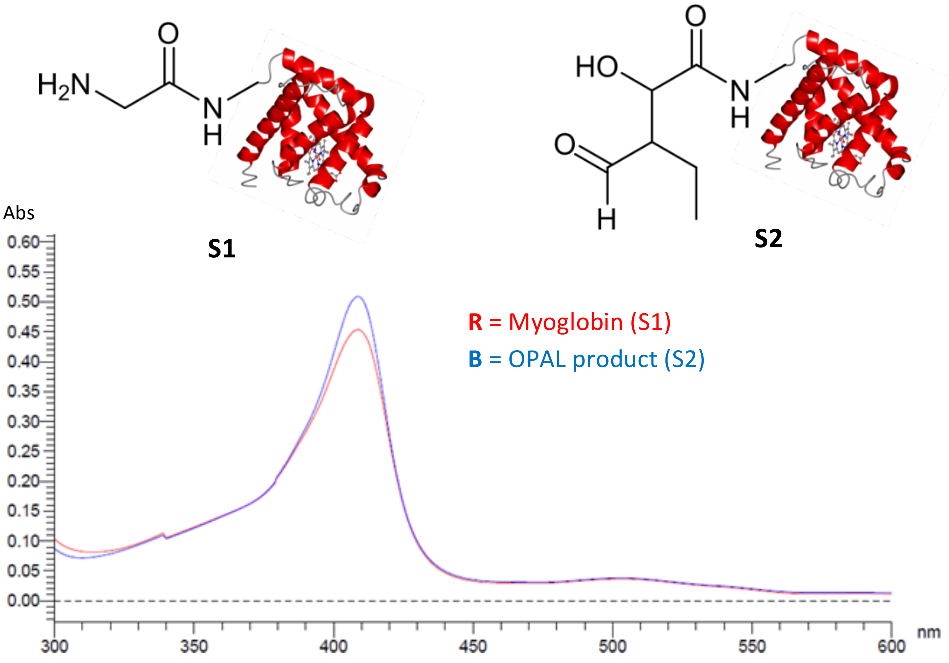

Supplementary Figure 2: UV-Vis measurements of myoglobin (red line) and α-ethyl-β-hydroxy aldehyde myoglobin (blue line).

Tandem mass spectrometry data of trypsin digest

Note on peptide nomenclature: For all analyses of MS/MS data of aldol/dually modified peptide/protein products, all peptides are treated as 'H_2N-LSDGEWQQVLNVWGK-OH' species that have been modified at their *N*-terminus. This allows for simplification of the MS/MS data, and is in line conventional peptide fragmentation analysis.

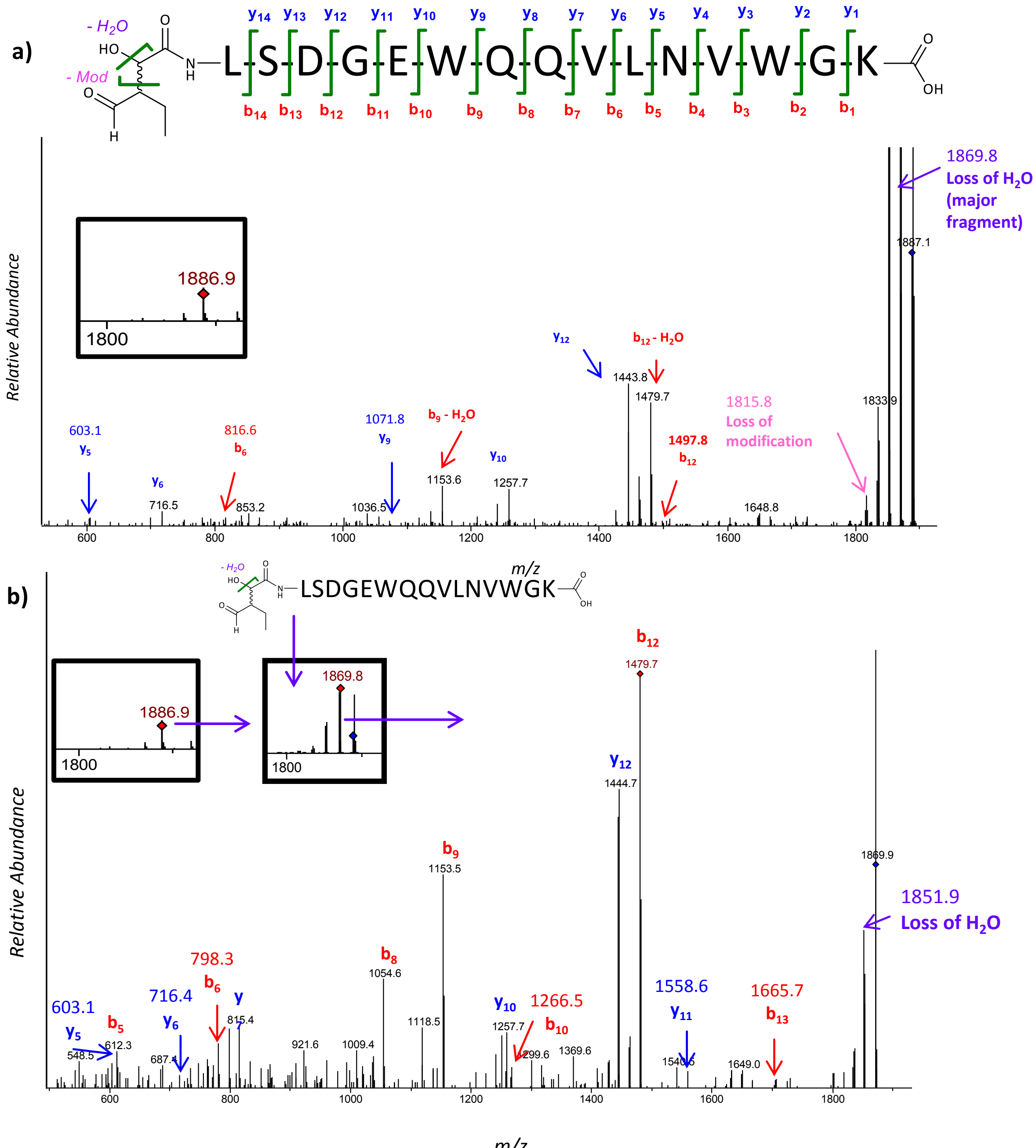

Supplementary Figure 3: a) MS/MS data of the anticipated *N*-terminal fragment of α-ethyl-β-hydroxy aldehyde-myoglobin **S2** resulting from trypsin digestion. The major peak corresponds to a loss of 18 Da, arising from a loss of H_2O at the β-hydroxy-aldehyde position. b) MS/MS, followed by MS/MS of the major fragment of the anticipated *N*-terminal fragment of α-ethyl-β-hydroxy aldehyde-myoglobin **S2** resulting from trypsin digestion. The resulting fragments from the 1869.8 Da fragment confirm both the presence of the β-hydroxy aldehyde group, and that the modification has occurred site-selectively at the α-oxo aldehyde position.

Tabulated kinetic data

Supplementary Table 1: Tabulated kinetic data for the OPAL.

Donor	Organocatalyst	Organocatalyst loading (mM)	Rate Constant (M^{-1} s^{-1})	Error of Rate Constant ($M^{-1}s^{-1}$)
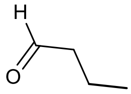 **7**	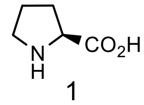 1	1	0.0009	0.00006
		10	0.0033	0.0001
		25	0.0100	0.0006
	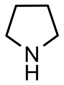 S3	1	0.0005	0.00003
		10	0.0037	0.0002
		25	0.0058	0.0005
	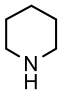 S4	1	>0.00001	-
		10	0.0009	0.00006
		25	0.0016	0.00016
	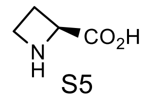 S5	1	0.0004	0.00003
		10	0.0024	0.00008
		25	0.0052	0.00008
	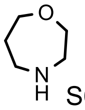 S6	1	0.0022	0.00015
		10	0.0166	0.0012
		25	0.0252	0.0035
	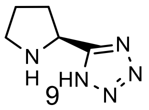 9	1	0.0092	0.001
		10	0.0551	0.004
		25	0.0977	0.009
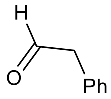 **10**	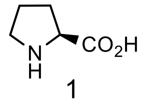 1	1	1.6840	0.106
		10	4.6620	0.219
		25	7.8990	0.579
	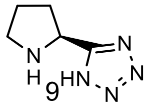 9	1	3.7920	0.294
		10	11.8200	1.18
		25	23.9470	1.98

Tandem mass spectrometry data of modified peptide

Note on peptide nomenclature: For all analyses of MS/MS data of aldol/dually modified peptide/protein products, all peptides are treated as 'H_2N-LYRAG-OH' species that have been modified at their *N*-terminus. This allows for simplification of the MS/MS data, and is in line conventional peptide fragmentation analysis.

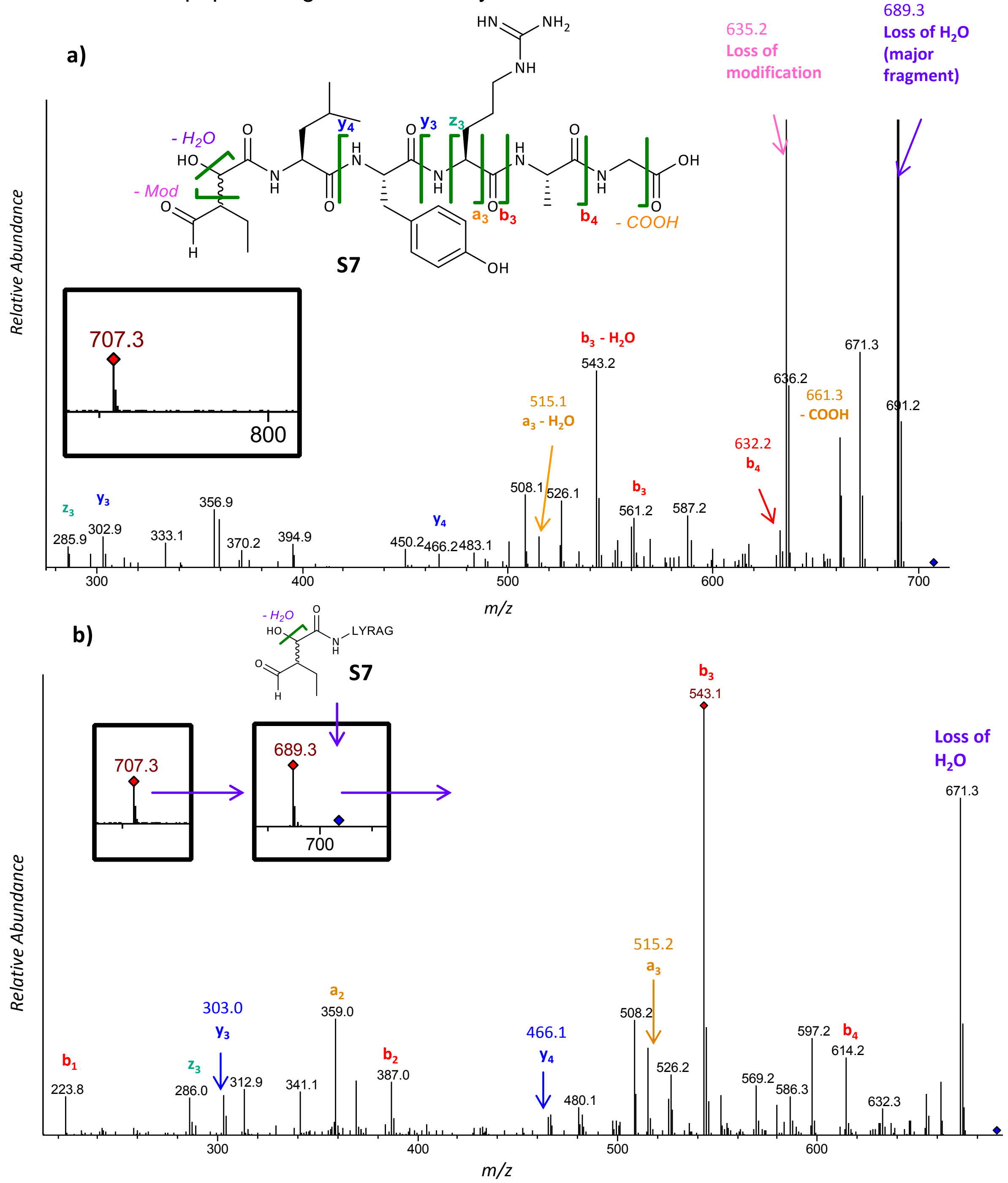

Supplementary Figure 4: a) MS/MS data of α-ethyl-β-hydroxy aldehyde LYRAG **S7**. The major peak corresponds to a loss of 18 Da, arising from a loss of H_2O at the β-hydroxy-aldehyde position. b) MS/MS, followed by MS/MS of the major fragment of α-ethyl-β-hydroxy aldehyde LYRAG **S7**. The resulting fragments from the 689.3 Da fragment confirm both the presence of the β-hydroxy aldehyde group, and that the modification has occurred site-selectively at the α-oxo aldehyde position.

Effects of organocatalyst and aldehyde donor on the OPAL of proteins

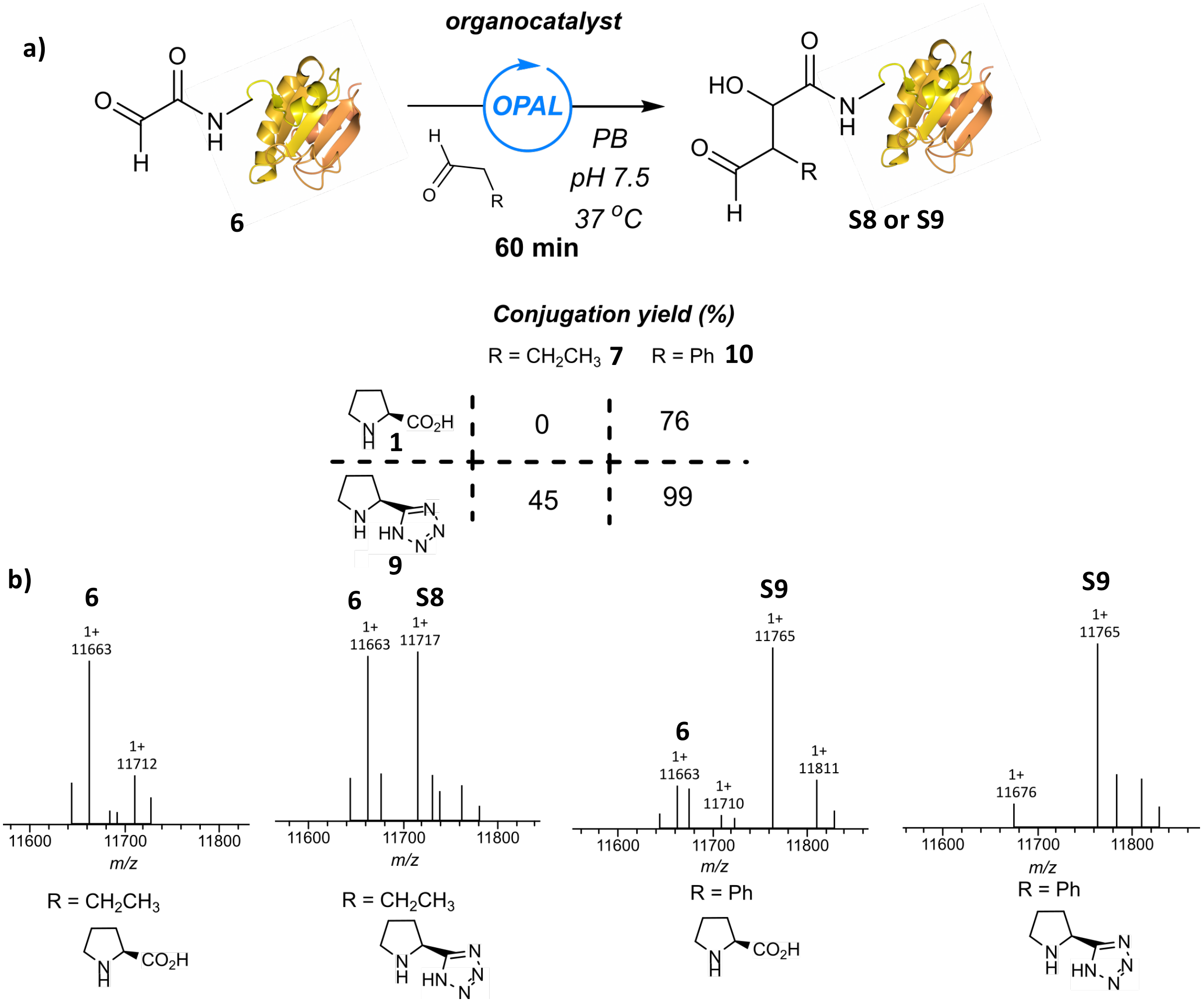

Supplementary Figure 5: a) Outline of screening the effects of different organocatalysts and aldehyde donor species for optimisation of the OPAL using glyoxyl-thioredoxin **6**, and the obtained conjugation yields for each combination of organocatalyst and aldehyde donor. b) Associated ESI-MS data.

Testing hydrolytic stability of azide labelled thioredoxin

A desalted aliquot of azide labelled thioredoxin (25 µM, 25 µL, 25 mM PB pH 7.5, desalted using a PD MiniTrap G-25 column (GE Healthcare Life Sciences), eluting into 25 mM PB pH 7.5) was incubated at 37 °C over the course of 72 h. LC-MS data of the sample was collected at 24 h intervals. No hydrolysis of azide labelled thioredoxin to give glyoxyl-thioredoxin was observed as judged by LC-MS, highlighting the hydrolytic stability of the OPAL products.

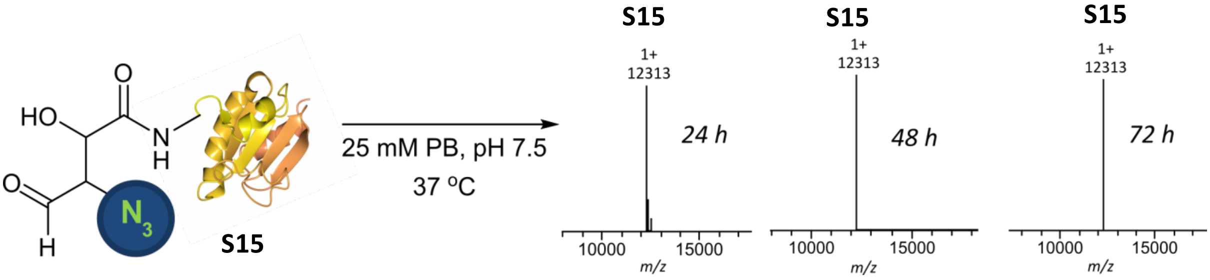

Supplementary Figure 6: Structure of OPAL product azide tagged thioredoxin **S15**, and associated MS data after periods of incubation at 37 °C to determine hydrolytic stability.

Installation of an unnatural α-oxo aldehyde side chain

Synthesis of unnatural amino acid **S10**, and expression and purification of sfGFP(N150ThzK) **S11** and GFP(Y39ThzK) **S12** was performed as previously described[4] using unnatural thiazolidine lysine (ThzK) amino acid and pEVOL pylRS WT [5].

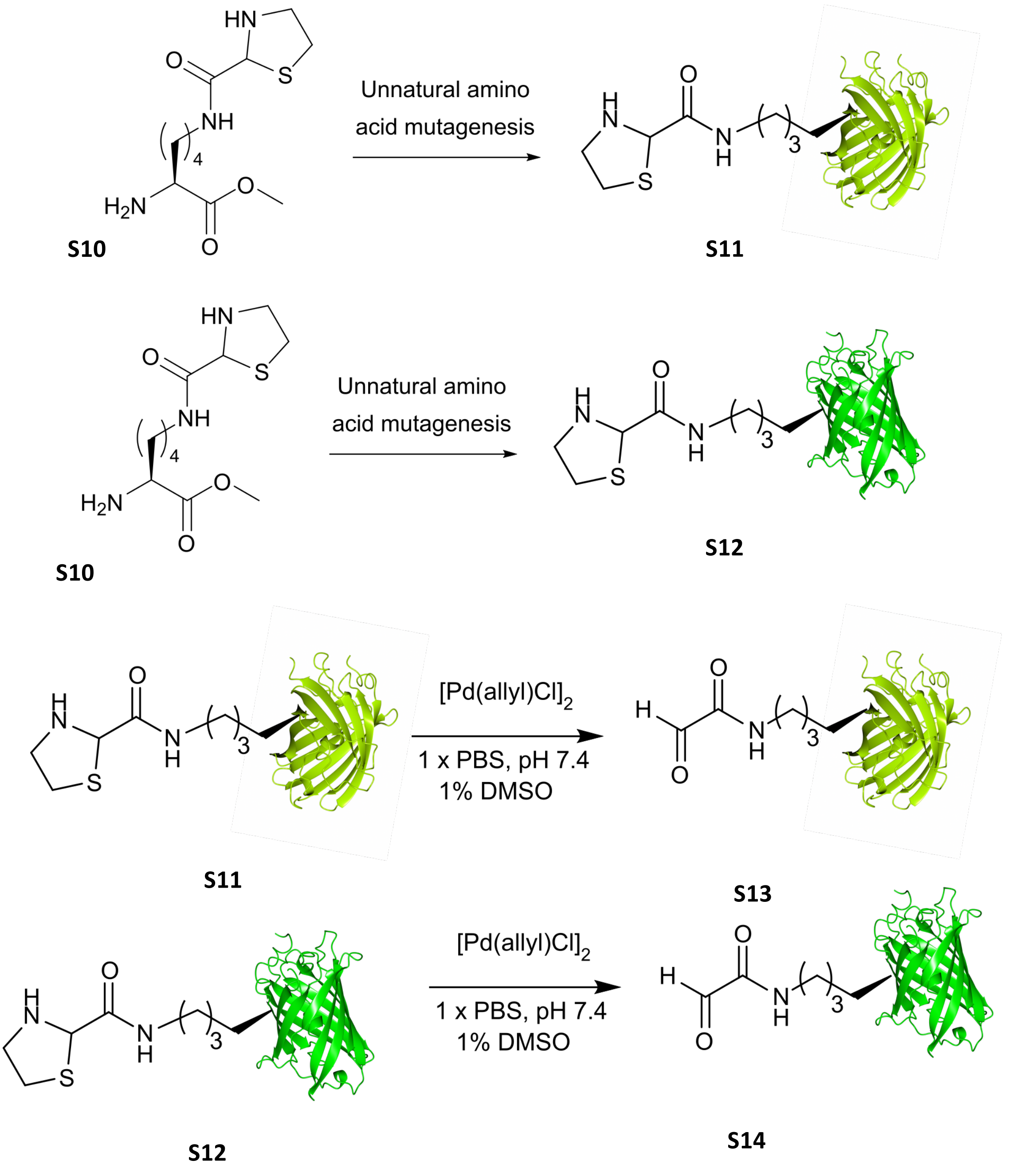

Supplementary Figure 7: Installation of an unnatural thiazolidine side chain using unnatural amino acid mutagenesis, and palladium mediated decaging to reveal the unnatural α-oxo aldehyde side chain as previously described[4] and on page S53-54.

Site-selective biotinylation of GFP in cell lysate and subsequent protein pulldown

A 10 mL culture of cells expressing Ser-GFP(Y39ThzK) **S12** (prepared as described previously [4]) was harvested by centrifugation. The resulting cell pellets were resuspended in 1.25 mL of 4 x PBS and lysed by sonication on ice for 9 x 30s with 30 s intervals. The cell lysate was clarified by centrifugation (17000 x g, 4 ^{o}C, 15 min), and the pelleted, insoluble matter was discarded. The supernatant was retained, and both the concentration and content of GFP **S12** was determined by UV/Vis absorbance measured at 488 nm assuming a molar extinction coefficient of ε = 55,000 M^{-1} cm^{-1} for GFP (GFP conc^{n} = 0.643 mg mL^{-1}, GFP content = 803 μg). A 1 mL sample of the supernatant was then carried forward for palladium-mediated decaging (GFP conc^{n} = 0.643 mg mL^{-1}, GFP content = 0.643 μg). Ten 100 μL aliquots of cell lysate were charged with 1 μL of a 30 mM allylpalladium(II) chloride dimer stock solution in DMSO (final conc^{n} = 300 μM). Following mixing by pipetting, the reactions were allowed to sit at 25 ^{o}C for 1 h without further agitation. The reactions were then quenched by addition of 10 μL of a 3-mercaptopropanoic acid solution, 1% v/v solution, 10 x PBS (final conc^{n} = 0.1% v/v) to each aliquot, and allowed to sit at 25 ^{o}C for 15 min without further agitation. The reactions were pooled, desalted using PD MiniTrap G-25 columns (GE Healthcare Life Sciences), eluting with 25 mM PB pH 7.5, and concentrated to 180 μL using 10,000 MWCOs (Amicon Ultra-0.5 mL Centrifugal Filters) to give the 'post-decaged' lysate containing GFP(ThzK39Oxo) **S14** (GFP conc^{n} = 2.5 mg mL^{-1}, GFP content = 451 μg, protein recovery from initial supernatant sample used= 70%). A 125 μL aliquot of 'post-decaged' lysate (GFP conc^{n} = 2.5 mg mL^{-1}, GFP content = 313 μg) was then carried forward for site-selective biotinylation. Five 25 μL aliquots of 'post-decaged' lysate in 25 mM PB pH 7.5 were charged with 5 μL of a 200 mM proline tetrazole **9** stock solution in 25 mM PB pH 7.5. The five solutions were then charged with 10 μL of a 5 mM biotin affinity tag **12** stock solution in 25 mM PB pH 7.5. Following mixing by pipetting, the reactions were allowed to sit at 37 ^{o}C for 60 min without further agitation. Excess affinity tag **12** was removed via spin concentration using 10,000 MWCOs (Amicon Ultra-0.5 mL Centrifugal Filters) to give 100 μL of the 'post-OPAL' lysate containing internally biotinylated GFP **S16** (GFP conc^{n} =1.73 mL^{-1}, GFP content = 173 μg, protein recovery from 'post-decaged' lysate sample used = 55%). A 'post-OPAL' lysate sample containing a 5 μg GFP content was retained for SDS-PAGE analysis. The remaining 'post-OPAL' lysate (GFP content = 168 μg) was loaded onto a 2 mL monomeric avidin agarose column (prepared in house using Pierce™ Monomeric Avidin Agarose according to the user guide provided, ThermoFisher Scientific), washed with 1 x PBS pH 7.4, and eluted using 2 mM biotin in 1 x PBS pH 7.4, collecting 1 mL fractions, according to the user guide provided. In total, one 2 mL fraction of flowthrough was collected (Fraction FT), six fractions of 2 mL washes with 1 x PBS were collected (Fractions 1-6), and 14 fractions of 1 mL washes with 2 mM biotin in 1 x PBS pH 7.4 were collected (Fractions 7-20). Fractions were first visualised for protein fluorescence using a Syngene G:BOX Chemi XRQ equipped with a Synoptics 4.0 MP camera in line with GeneSys software (Version 1.5.7.0), and fractions of interest were subsequently analysed via SDS-PAGE. Fractions 1, 7, 8, 9, 10, 11, 12, 13, and 14, were also analysed by UV/Vis absorbance measured at 488 nm for GFP content. Fraction 1 was determined to contain 40 μg total GFP content, whereas fractions 7, 8, 9, 10, 11, 12, 13, and 14, were determined to contain 123 μg total GFP content, leaving 5 μg of 168 μg 'post-OPAL' GFP material unaccounted for. Overall, the pooled fractions 7-14 resulted in a 73% recovery of internally biotinylated GFP **S16** that was originally loaded onto the monomeric avidin agarose column.

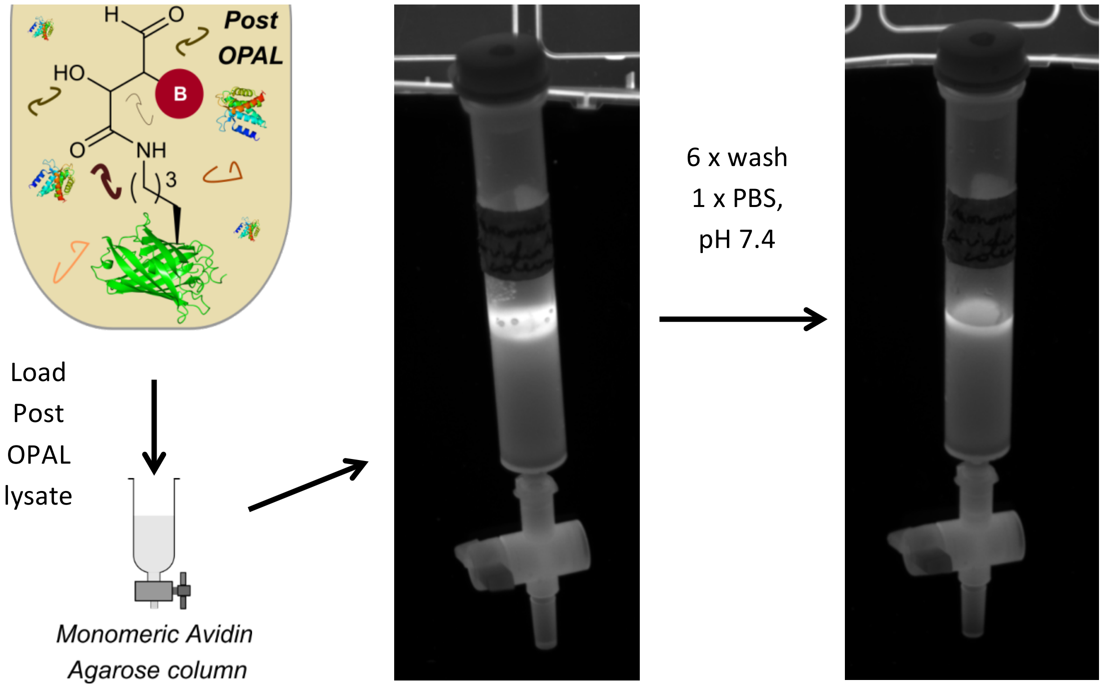

Supplementary Figure 8: Schematic of loading cell lysate containing internally biotinylated GFP **S16** onto monomeric avidin agarose column. Visualisation of column pre and post washing with 1 x PBS pH 7.4 reveals GFP material bound to the column. Visualisation of fluorescence was performed using a Syngene G:BOX Chemi XRQ equipped with a Synoptics 4.0 MP camera in line with GeneSys software (Version 1.5.7.0)

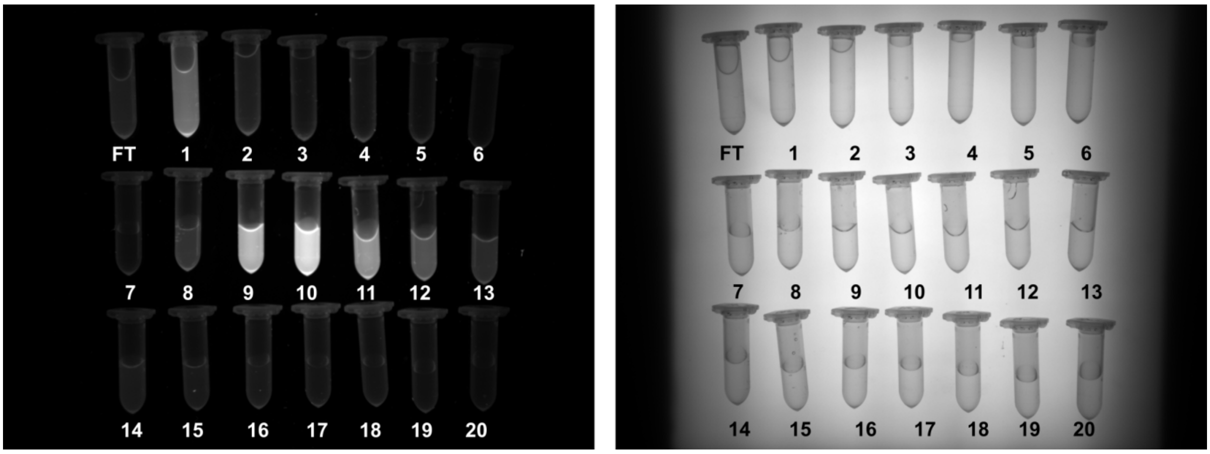

Supplementary Figure 9: Collected fractions from purification of cell lysate containing internally biotinylated GFP **S16** using a monomeric avidin agarose column. FT = Flowthrough. Fractions 1-6 = Washed with 1 x PBS pH 7.4. Fractions 7-20 = Elution with 2 mM biotin in 1 x PBS pH 7.4. Left: Fluorescent imaging of collected fractions. Right: White light imaging of collected fractions. Images were captured and analysed using a Syngene G:BOX Chemi XRQ equipped with a Synoptics 4.0 MP camera, with GeneSys software (Version 1.5.7.0).

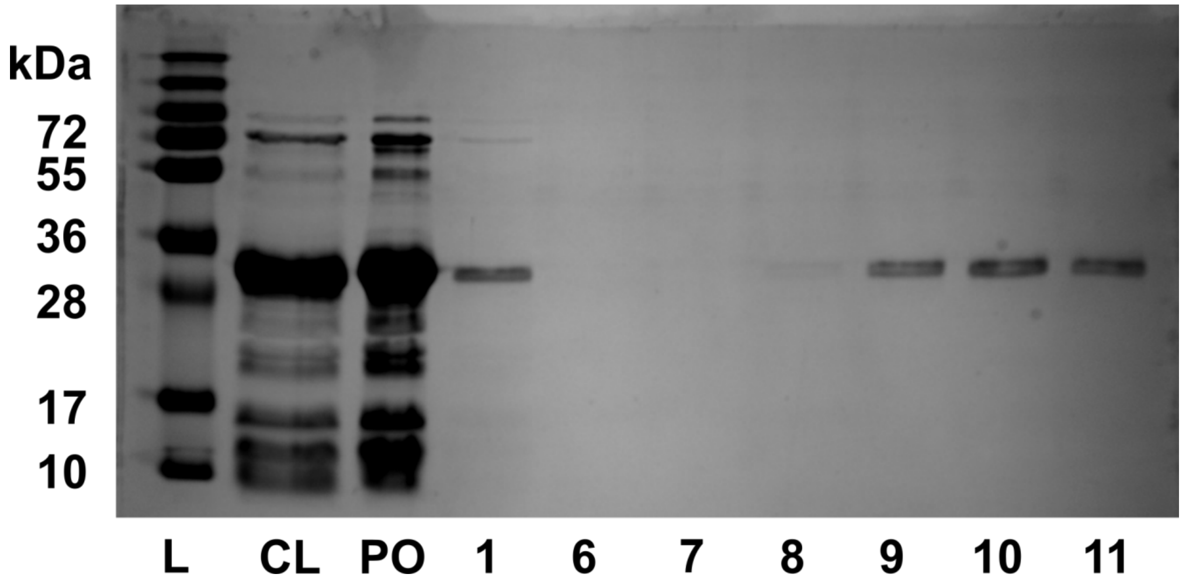

Supplementary Figure 10: SDS-PAGE analysis of cell lysate samples and fractions of interest collected from purification of cell lysate containing internally biotinylated GFP **S16** using a monomeric avidin agarose column. L = Ladder. CL = Cell lysate (before addition of allylpalladium(II) chloride dimer). PO = Post OPAL (following removal of excess affinity tag **12** and directly before loading onto the monomeric avidin agarose column). Collected fractions follow same numerical labelling as seen in Supplementary Figure 9.

These results demonstrate that site-selective modification of proteins via the OPAL strategy can be successfully carried out in complex biological media without compromising protein integrity, and that selective protein pulldown can be achieved through site-selective biotinylation and subsequent purification using a monomeric avidin agarose column.

Dual modification of peptides through the iso-Pictet Spengler and ABAO ligations

a)

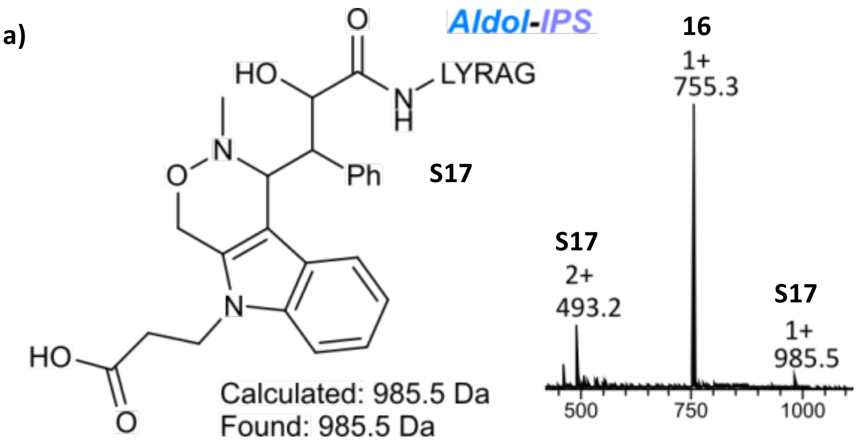

b)

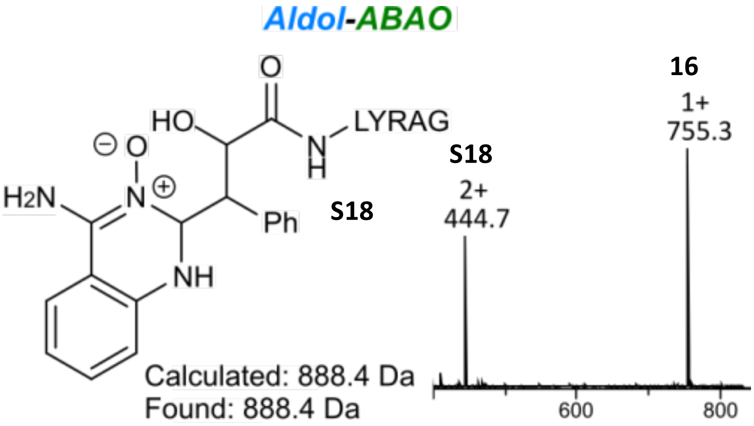

Supplementary Figure 11: a) Structure of α-phenyl-β-hydroxy iso-pictect-spengler-LYRAG **S17** and associated ESI-MS data. b) Structure of α-phenyl β-hydroxy-aminobenzamidoxime-LYRAG **S18**, and associated ESI-MS data.

Screening of aniline catalysts

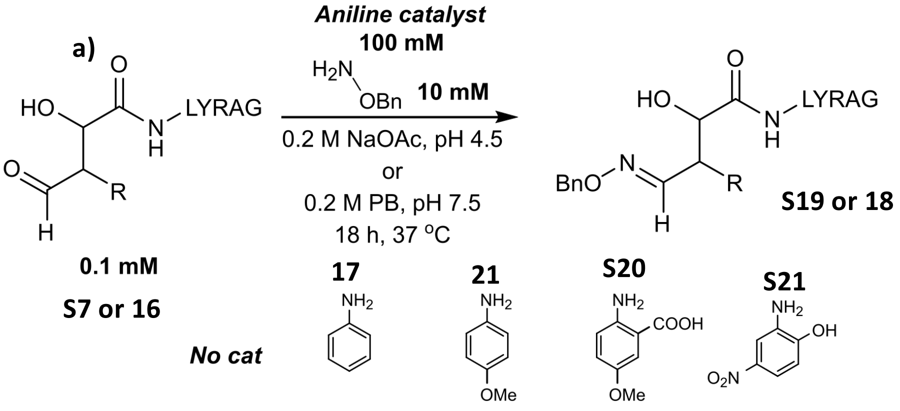

R = CH_2CH_3 (S7)

pH	No cat	17	21	S20	S21
	Conjugation yield (%)				
4.5	29	46	56	42	*Precipitates*
7.5	18	88	96	95	99

R = Ph (16)

pH	No cat	17	21	S20	S21
	Conjugation yield (%)				
4.5	42	61	81	34	*Precipitates*
7.5	67	95	92	97	97

A

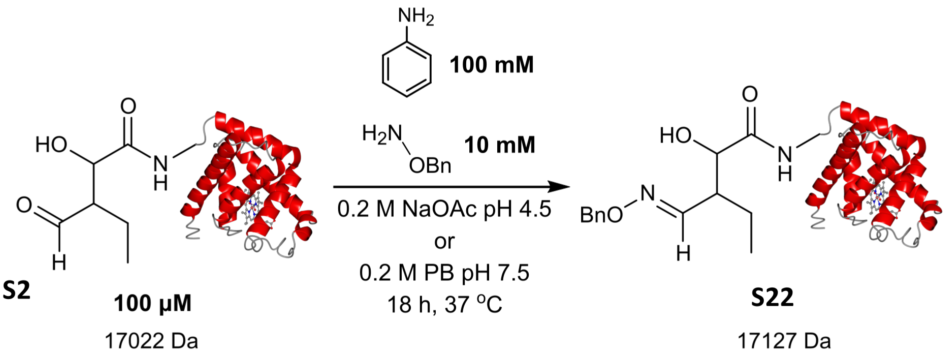

B

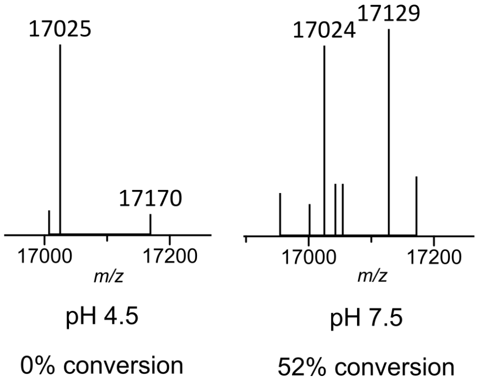

Supplementary Figure 12: a) Screening of aniline catalysts at pH 4.5 and pH 7.5 in the aniline catalysed oxime ligation of α-substituted-β-hydroxy aldehyde LYRAG **S7** or **16**, and the obtained conversions to the α-substituted-β-hydroxy benzyloxyimino-LYRAG **S19** or **18** (as judged by LC-MS) in each case. b) Screening the effects of pH on aniline catalysed oxime ligation of α-ethyl-β-hydroxy aldehyde myoglobin **S2**, and the obtained conversions to the α-ethyl-β-hydroxy aldehyde myoglobin **S22** (as judged by LC-MS) in each case.

Mass spectrometry and SDS-PAGE analysis of bi-functional protein constructs

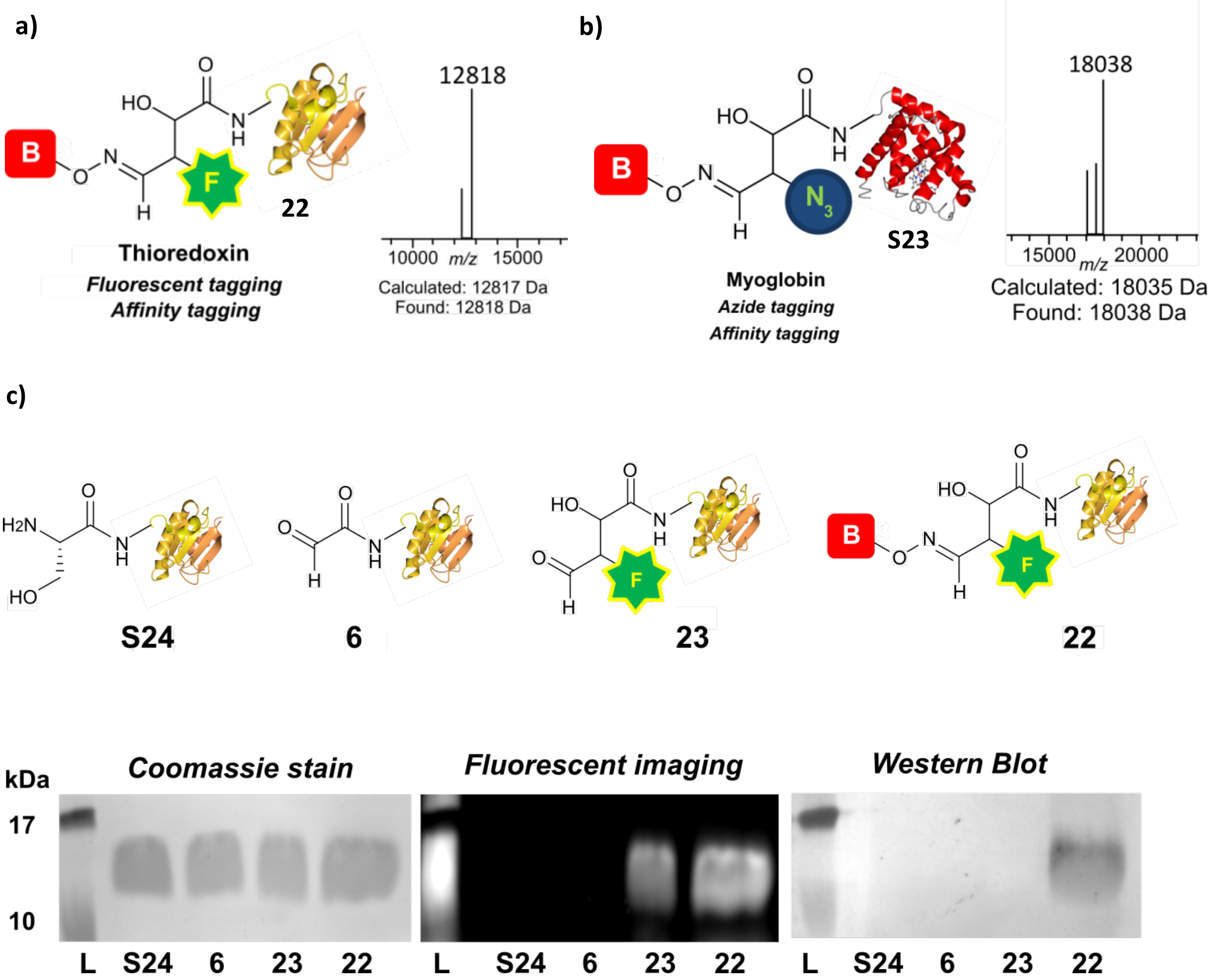

Supplementary Figure 13: a) Structure of fluorescently, biotinylated thioredoxin **22**, and associated ESI-MS data. b) Structure of azide labelled, biotinylated myoglobin **S23**, and associated ESI-MS data. c) SDS-PAGE analysis of various thioredoxin constructs. L = Ladder (Molecular weight marker).

Coomassie staining of thioredoxin **S24**, glyoxyl-thioredoxin **6**, fluorescently labelled thioredoxin **23**, and fluorescently labelled, biotinylated thioredoxin **22** confirm the presence of each protein respectively. As anticipated, only fluorescent proteins **23** and **22** were detected in the fluorescent imaging experiment, and only biotinylated protein **22** was detected in the Western Blot experiment (detecting for biotin).

SDS-PAGE analysis of azide labelling and biotinylation of thioredoxin

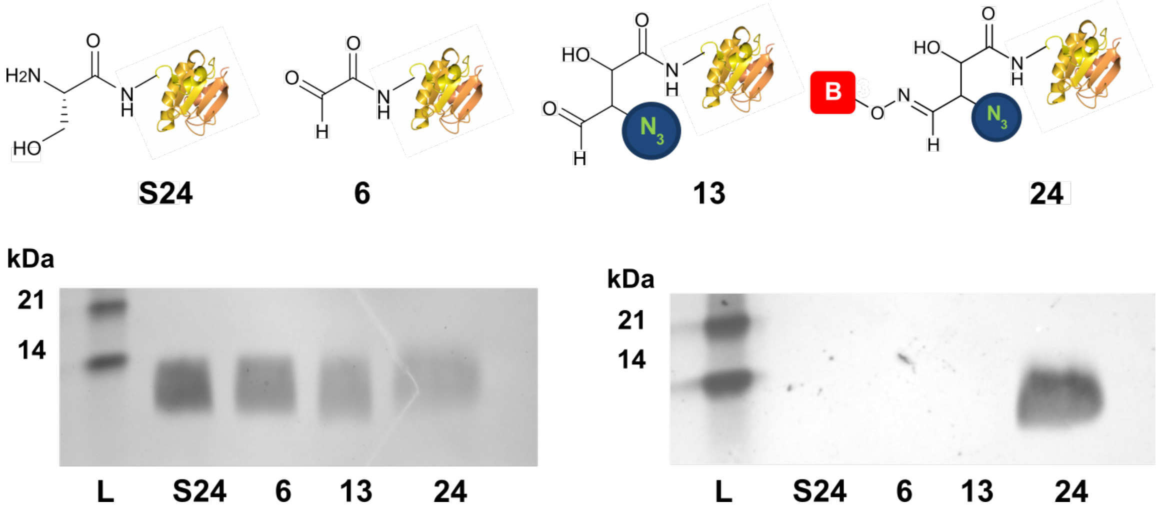

Supplementary Figure 14: a) SDS-PAGE analysis of various thioredoxin constructs. L = Ladder (Molecular weight marker).

Coomassie staining of thioredoxin **S24**, glyoxyl-thioredoxin **6**, azide labelled thioredoxin **13**, and azide labelled, biotinylated thioredoxin **24** confirm the presence of each protein respectively. As anticipated, only biotinylated protein **24** was detected in the Western Blot experiment.

SDS-PAGE analysis of fluorescent labelling and PEGylation of myoglobin

a)

b)

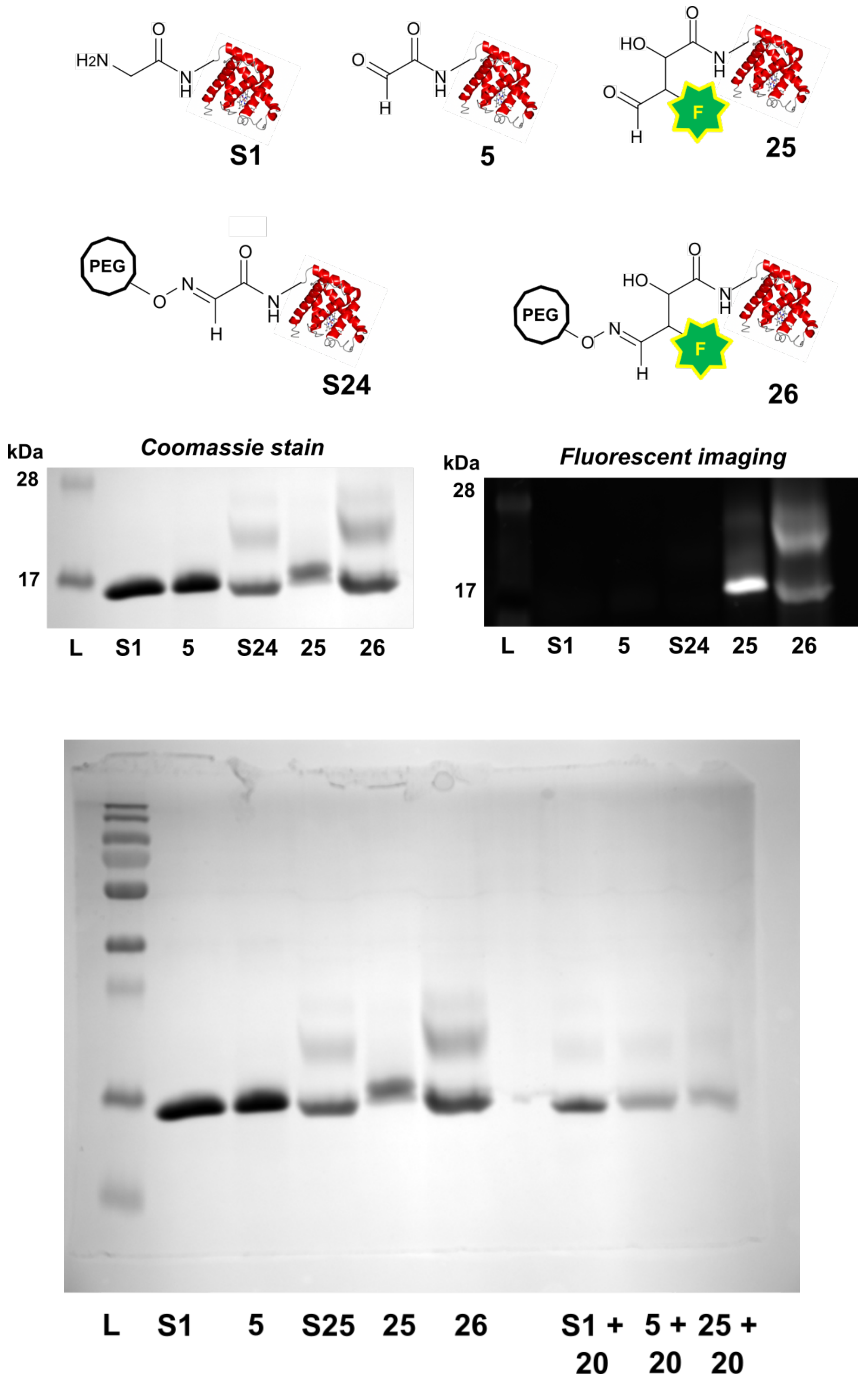

Supplementary Figure 15: a) SDS-PAGE analysis of various myoglobin constructs. L = Ladder (Molecular weight marker). b) Coomassie stained SDS-PAGE analysis of myoglobin constructs, and associated myoglobin-PEG related controls

Coomassie staining of myoglobin **S1**, glyoxyl-myoglobin **5**, mono-PEGylated myoglobin **S25**, fluorescently labelled myoglobin **25**, and fluorescently labelled, PEGylated myoglobin **26** confirm the presence of each protein respectively. As anticipated, only samples containing fluorescently labelled proteins **25** and **26** were detected in the fluorescent imaging

experiment. For protein samples treated with aminooxy PEG 2k **20** two protein bands are observed, with the upper band corresponding to a single addition of the polymer unit to the protein. The results obtained for this experiment were consistent with samples containing both unmodified protein (**5** Or **25**) and PEGylated protein (**S25** or **26** respectively).

It is notable that in this set of experiments is the lower protein band in samples treated with aminooxy PEG 2k **20** runs slightly lower than expected compared to samples containing unmodified protein that have not been treated with aminooxy PEG **20**. To investigate this phenomenon, three samples of myoglobin **S1**, glyoxyl myoglobin **5**, and fluorescently labelled myoglobin **25** were treated with aminooxy PEG 2k **20** , allowed to sit at 37 °C for 3 hr, and then analysed by SDS PAGE analysis. We found that these samples were also observed at a slightly lower molecular weight than expected.

Testing the hydrolytic stability of bi-functional peptides

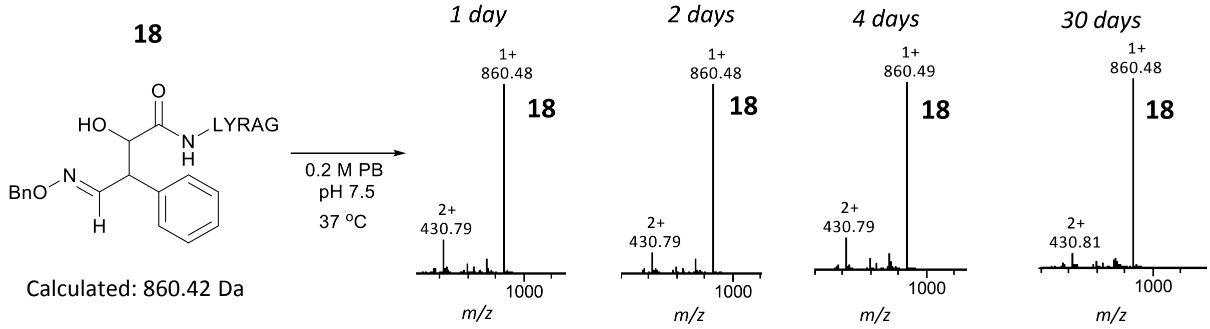

Supplementary Figure 16: The α-phenyl-β-hydroxy benzyloxyimino-LYRAG **18**, and associated MS data after periods of incubation at 37 °C.

HASPA bearing an *N*-terminal α-oxo aldehyde

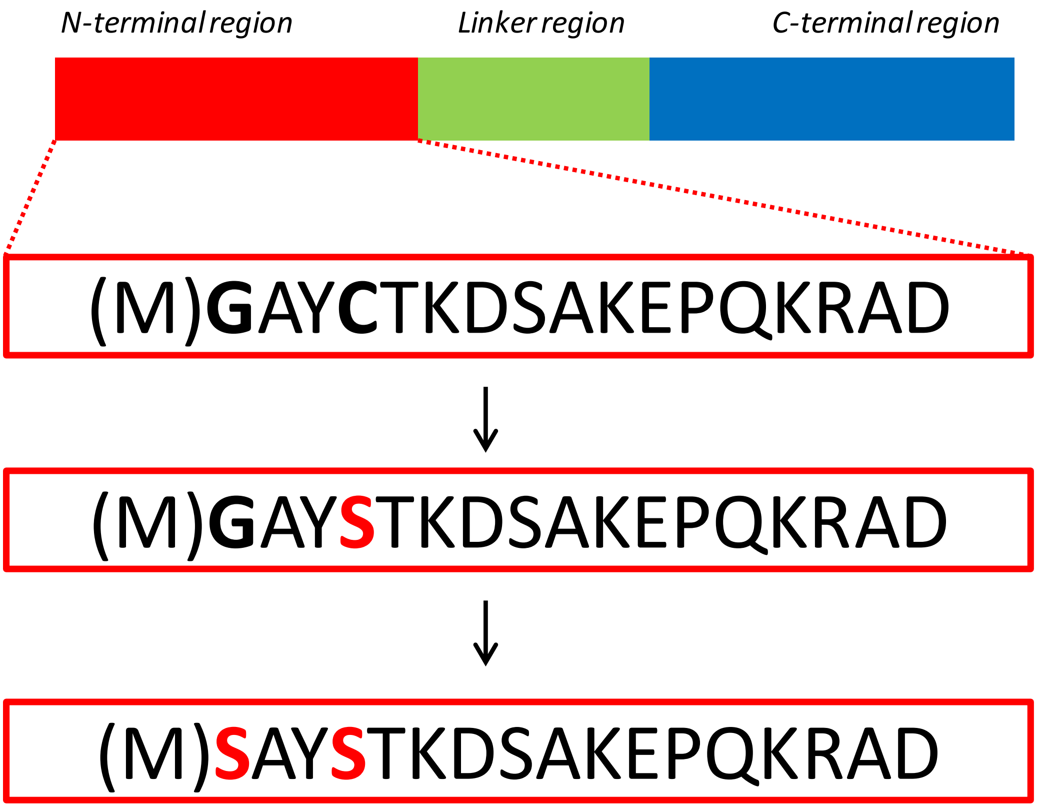

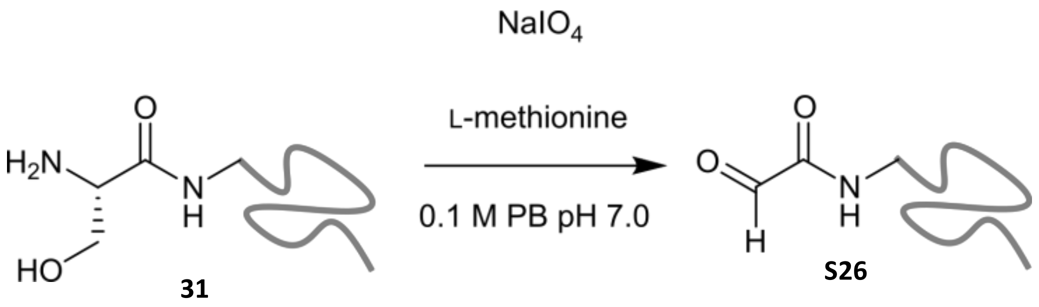

Supplementary Figure 17: a) Domain composition of hydrophilic acylated surface protein A (HASPA). For chemical myristoylation experiments, the N-terminal domain comprises of both a G1S mutation, and a C4S mutation. b) Outline of preparing HASPA bearing an *N*-terminal α-oxo aldehyde.

Note: Upon expression of HASPA proteins, the *N*-terminal Met is removed to generate an amino-terminal Gly and a substrate for *N*-myristoyltransferase. This Gly residue is designated G1 in all HASPA proteins expressed in this work.

Protein NMR of HASPA

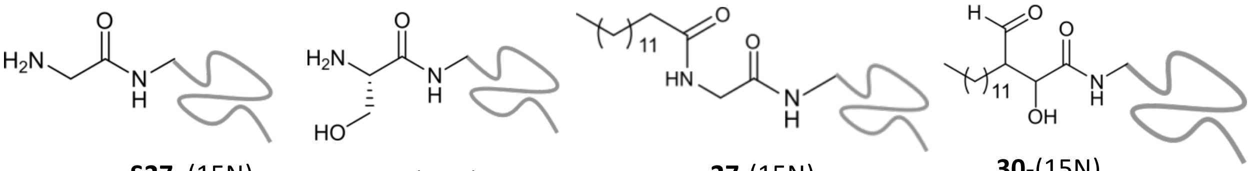

S27-(15N) **31**-(15N) **27**-(15N) **30**-(15N)

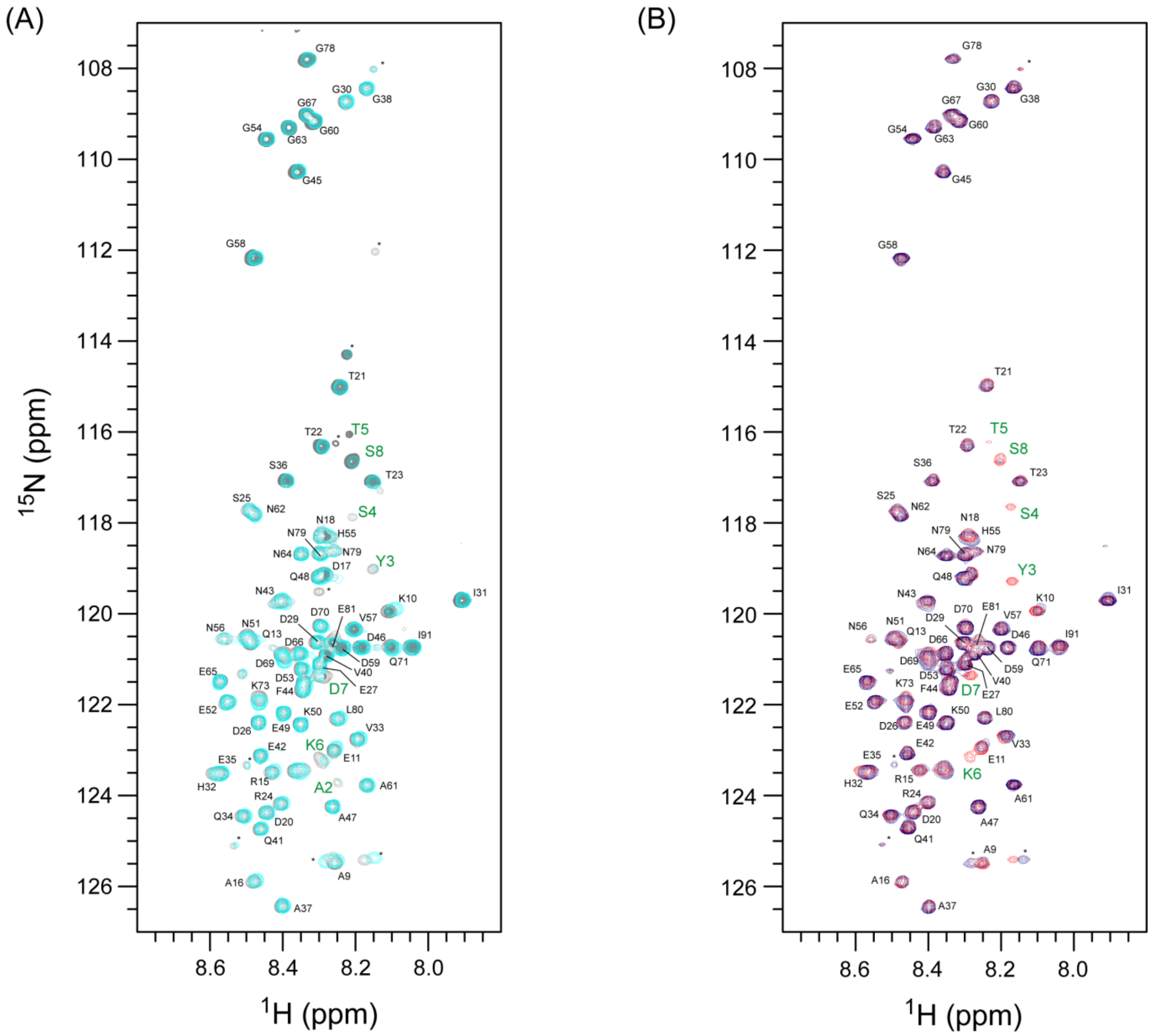

Supplementary Figure 18: 2D (^{1}H, ^{15}N) HSQC spectra of [^{15}N]labelled HASPA. (A) Comparison of unmodified (grey) **S27** and enzymatically myristoylated (cyan) HASPA **27**. (B) Comparison of unmodified HASPA G1S **31** (red) and chemically myristoylated (blue) HASPA G1S **30**. Spectra were recorded of 100 µM HASPA samples in 20 mM HEPES, pH 6.5, 50 mM NaCl. (^{1}H,^{15}N) resonance assignments are indicated. Unassigned peaks are denoted by asterisks.

Liposome data for HASPA

Preparation of liposomes

Liposomes were prepared in order to investigate whether chemically myristoylated and palmitoylated HASPA associates with membrane lipids. Liposomes were prepared by using 1,2-Diacyl-*sn*-glycero-3-phosphocholine (PC) and cholesterol (Ch). Lipids were solubilised in 9:1 chloroform-methanol (v/v), stocks were prepared at a 7:1 ratio of PC to Ch and the solvent was evaporated under N_2. Dried lipids were hydrated to a final concentration of 1-2 mM in lipid rehydration buffer (100 mM NaCl, 1 mM $CaCl_2$, and 50 mM Tris-Cl [pH 7.4]) or PBS + 1 mM $CaCl_2$ for 30 min at room temperature. The rehydrated lipids were subjected to four freeze/thaw cycles in liquid nitrogen and a 45 °C water bath, and extruded through a 100 nm Nanosizer Liposome mini extruder (T & T Scientific Corporation) to produce liposomes. Dynamic light scattering (DLS) was used to confirm the size of the liposomes (Supplementary Figure 19).

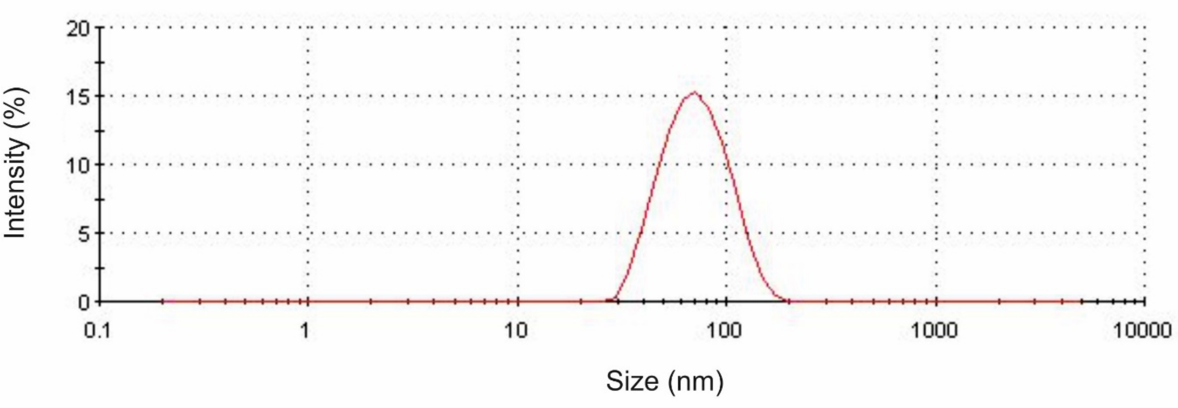

Supplementary Figure 19: DLS analysis of POPC/cholesterol liposomes after extrusion through a 100 nm Nanosizer Liposome mini extruder

Liposome binding assays

Chemically myristoylated HASPA

Chemically myristoylated G1S HASPA **30** was dialysed into phosphate buffer saline (PBS) using a Slide-A-Lyzer dialysis cassette (MwCO 3500 Da). After dialysis, HASPA **30** was quantified after SDS-PAGE analysis, by comparison to a known amount of unmodified G1S HASPA **31**. The protein was then lyophilised and stored at -20 °C. For the liposome sedimentation assay, chemically myristoylated HASPA **30** and unmodified G1S HASPA **31** (20 µg) respectively, was incubated with 50 µL of 1 mM PC:Ch liposomes (0.66 mM final conc.) in 75 µL of lipid rehydration buffer at RT for 45 min. No lipid and no protein controls were analysed alongside the binding assay. 10% of each sample was saved as the loading control. The samples were ultracentrifuged at 100 000 rpm (4 °C, 1 h) and the unbound fraction saved. The pellet was suspended in 65 µL of lipid rehydration buffer and the

samples incubated at 37 °C for 30 min. The total, unbound and pellet fractions were analysed by SDS-PAGE (Supplementary Fig. 16) Approximately 50% of the myristoylated HASPA **30** was retained in the liposome pellet fraction (Supplementary Figure 20, A, lane:3). None of the unmodified G1S HASPA **31** was retained in the lipid pellet fraction as expected (Supplementary Figure 20, B, lane:3). The no liposome and no protein negative controls were as expected. These findings suggest that chemically myristoylated HASPA **30** associates with PC:Ch liposomes.

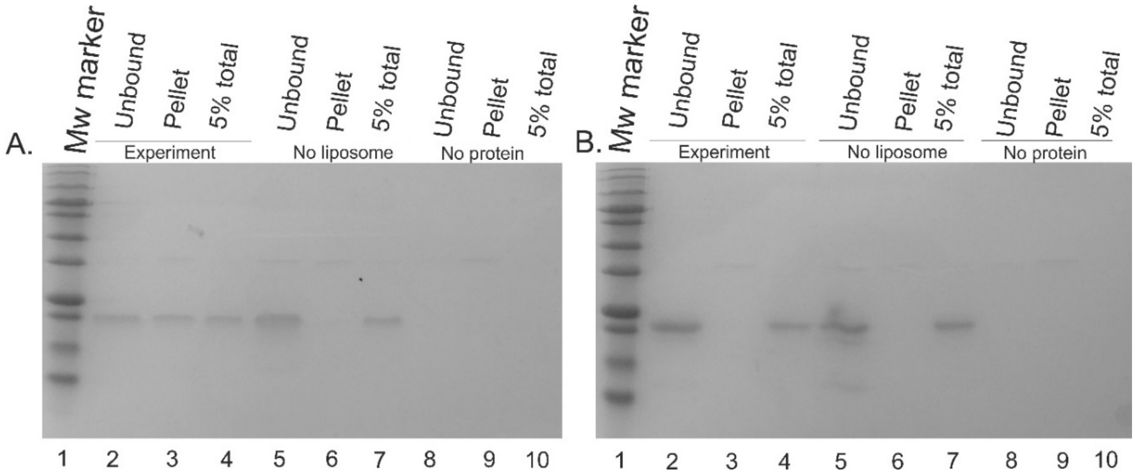

Supplementary Figure 20. Comparison of lane 3 in gel *A* (chemically myristoylated HASPA **30**) and gel *B* (unmodified HASPA **31)** confirms that HASPA binds PC:Ch liposomes only after modification.

Chemically myristoylated and dual modified HASPA

For effective comparison of the chemically myristoylated HASPA **30** and dual modified HASPA **33**, the liposome binding assay used above was adapted to include detergent. The chemically myristoylated HASPA **30** was desalted using a PD MiniTrap G-25 column (GE Healthcare Life Sciences), eluting into water, and lyophilised. The protein was resuspended in nickel binding buffer (PBS + 1% w/v sodium cholate, 20 mM imidazole). The dual modified HASPA **33** was diluted to 20% (v/v) EtOH in nickel binding buffer. In order to purify the modified proteins from any residual reaction components, both the chemically myristoylated **30** and the dual modified HASPA **33** were purified using His Spintrap columns (GE Healthcare). The proteins were eluted from the His Spintrap columns using nickel elution buffer (PBS + 1% w/v sodium cholate, 500 mM imidazole). The proteins were quantified after analysis by SDS-PAGE, by comparison to a known amount of unmodified G1S HASPA **31**.

For the liposome binding assays, 75 µg of unmodified, chemically myristoylated **30** or dual modified HASPA **33** was added to 250 µL of 2 mM PC:Ch liposomes and made up to 500 µL in nickel elution buffer. The protein/liposome suspensions were dialysed into PBS + 1mM $CaCl_2$ for 30 h at 4 °C using D-tube Dialyzer Midi dialysis casettes (MERCK, MwCo 3.5 kDa). Samples of 100 µL were taken after 10 min, 30 min, 2 h, 7 h and 30 h. Each sample was sedimented by ultracentrifugation (100 000 x g, 30 min, 4 °C). The liposome pellet was resuspended in 100 µL of PBS and the samples incubated at 37 °C for 20 min. The unbound and pellet fractions were analysed by SDS-PAGE (Supplementary Figure 21). Both the

chemically myristoylated **30** and the dual modified HASPA **33** bound to PC:Ch liposomes. No liposome binding was observed in reactions with the unmodified HASPA **31**.

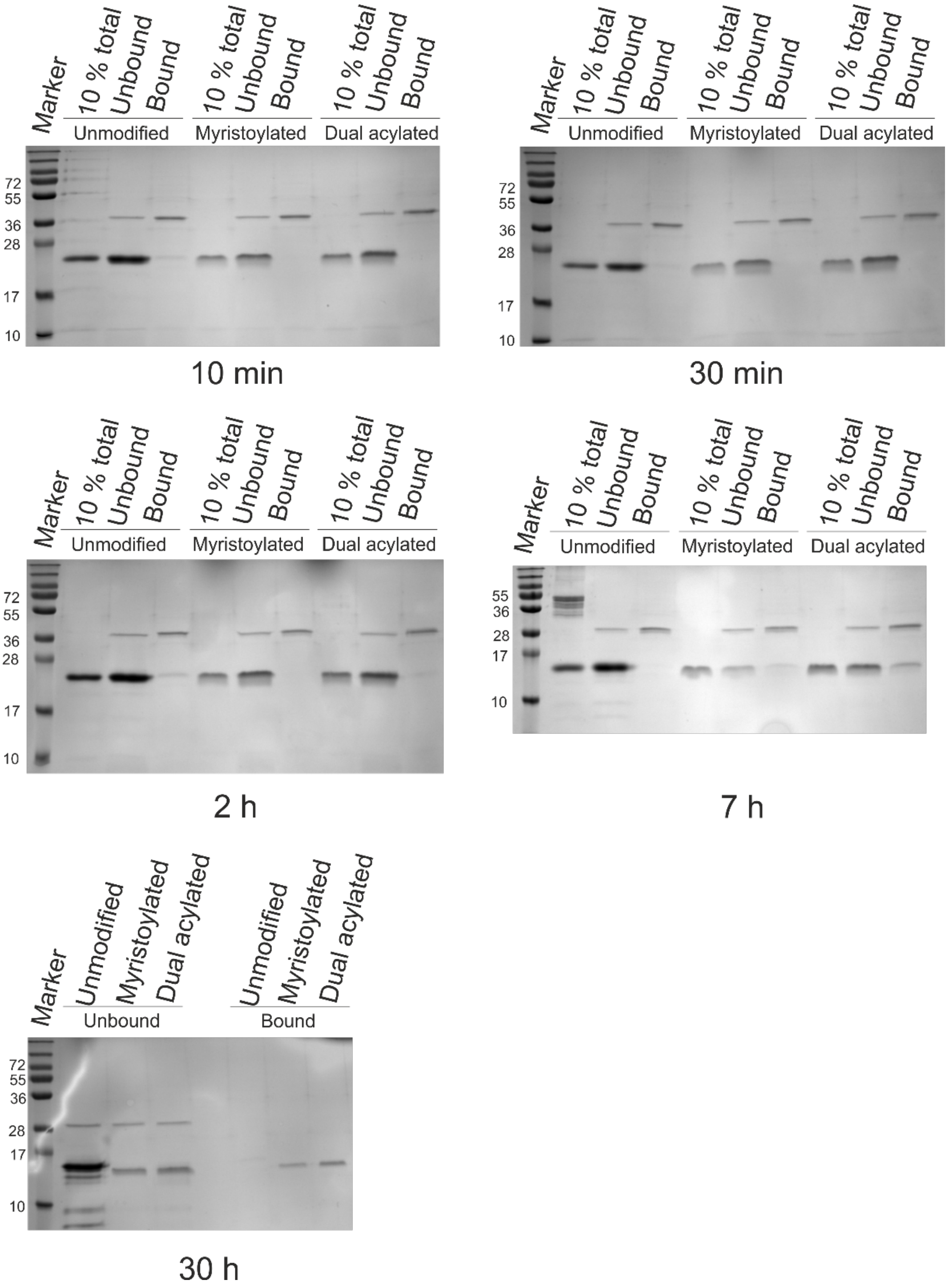

Supplementary Figure 21. Chemically myristoylated **30** (referred to as myristoylated) and dual modified HASPA **33** (referred to as Dual acylated) timecourse PC:Ch liposomes binding experiment over 30h. The band at 36 kDa is an unidentified degradation product or contaminant, which was frequently observed following incubation with liposomes and centrifugation at 100,000 x g. Note that the HASPA proteins are observed at different molecular weight in 14% SDS-PAGE gels (10 min-7 h), or 4-20% gradient gels (30h).

GelQuant.Net software provided by biochemlabsolutions.com was used to estimate the quantity of protein unbound (in the soluble fraction) and bound (in the pellet fraction) for each of **31**, **30**, and **33** at each time point, and expressed as a percentage of the total protein in both fractions (Supplementary Figure 22) for comparison. At 30 h, an estimated 0% of total unmodified HASPA **31** is bound to the liposomes, an estimated 16% of total chemically myristoylated HASPA **30** is bound to the liposomes, and an estimated 34% of dual modified HASPA **33** is bound to the liposomes.

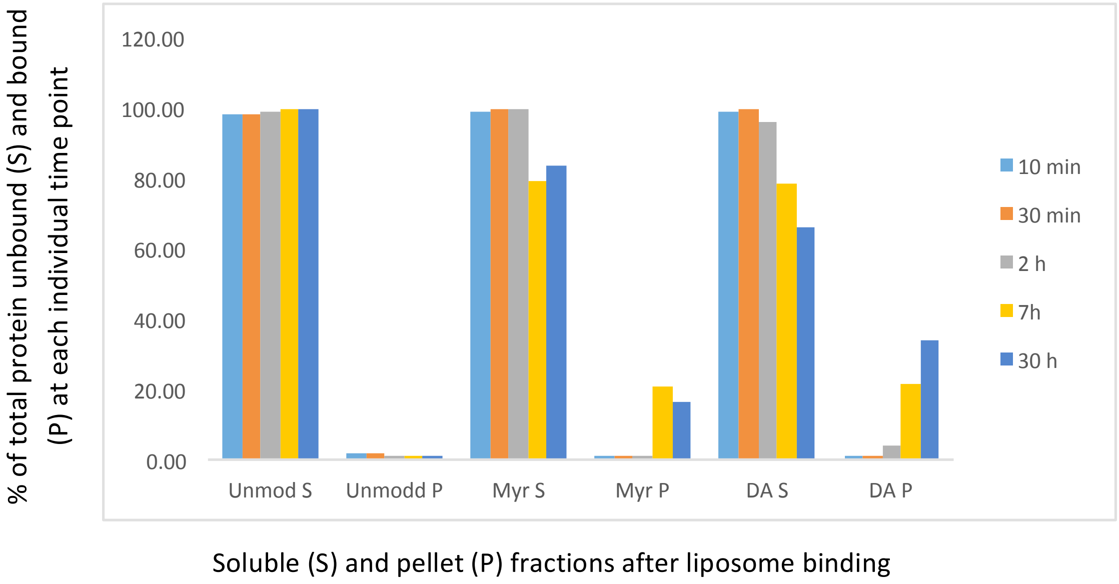

Supplementary Figure 22. GelQuant.Net analysis of SDS-PAGE protein band intensity for HASPA liposome binding experiments in Supplementary Figure 21. *Unmod* = unmodified HASPA **31**, *Myr* = chemically myrisoylated HASPA **30**, *DA* = dual modified HASPA **33**. Protein band intensities for both the soluble and pellet fractions of individual experiments using **31**, **30**, and **33** were combined for each time point, and the quantity of protein unbound (S fraction) and bound (P fraction) at each individual time point expressed as a % of total protein.

3. Synthesis of small molecules

Linker building block

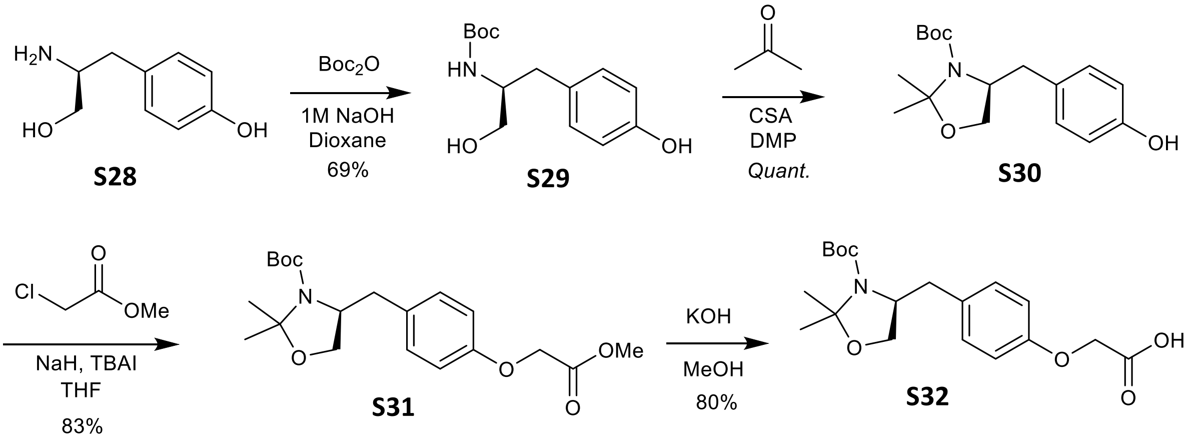

Supplementary Figure 23: Synthesis of linker building block

(*S*)-*tert*-butyl (1-hydroxy-3-(4-hydroxyphenyl)propan-2-yl)carbamate S29:

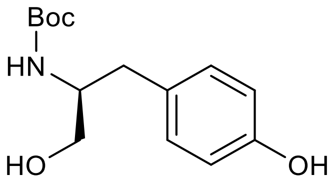

To a solution of L-tyrosinol hydrochloride **S28** (2.2 g, 10.8 mmol) in 1,4-dioxane (20 mL) at 0 °C, 1(N) NaOH solution (21.6 mL, 21.6 mmol) followed by Boc_2O (2.4 g, 10.8 mmol) were added. Reaction was stirred at room temperature for 3 hours when TLC (*n*-hexane- EtOAc 1:1) indicated complete conversion of the starting material. The solvent was evaporated and the resulting residue was dissolved in EtOAc and successively washed with 5% w/v aq. citric acid and H_2O. The organic layer was collected and dried over Na_2SO_4 and concentrated *in vacuo*. The crude residue was purified by column chromatography using *n*-hexane-EtOAc (1:1) as an eluent to furnish compound **S29** (2 g, 69%) as a white solid. $[\alpha]_D$ -24 (*c* 1, MeOH). R_f (*n*-hexane-EtOAc, 1:1) 0.16; **^{1}H NMR** (500 MHz, CD_3OD): δ 7.03 (d, *J* = 8.3 Hz, 2H, ArH_m), 6.70 (d, *J* = 8.3 Hz, ArH_o), 3.69 (m, 1H, CHN), 3.47 (brs, 2H, CH_2O), 2.76 (dd, *J* = 6.2 Hz, 13.7 Hz, 1H, $ArCH_2$), 2.58 (dd, *J* = 7.8 Hz, 13.2 Hz, 1H, $ArCH_2$'), 1.38 (s, 9H, $C(CH_3)_3$); **^{13}C NMR** (125 MHz, CD_3OD): δ 158.3, 157.0, 131.5 (2), 130.9, 116.3 (2), 80.1, 64.6, 55.7, 37.8, 29.0. **IR (ATR, cm^{-1})** 3400, 1670. **ESI-HRMS:** Found $[M+Na]^+$ 290.1353, $C_{14}H_{21}NNaO_4$, requires 290.1363.

(*S*)-*tert*-butyl 4-(4-hydroxybenzyl)-2,2-dimethyloxazolidine-3-carboxylate S30:

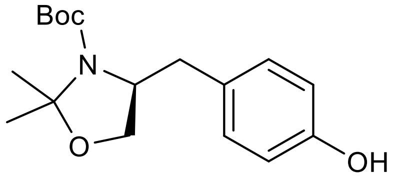

Compound **S29** (2 g, 7.5 mmol) was dissolved in anhydrous acetone (20 mL) and treated with 2,2-dimethoxypropane (2.8 mL, 22.5 mmol) and 10-camphorsulfonic acid (40 mg). The reaction went to completion after stirring at room temperature for 3 hours as indicated by TLC (*n*-hexane- EtOAc 3:1). Et_3N was added to neutralize the solution and the solvent was evaporated *in vacuo*. The crude residue obtained, was purified by flash chromatography using *n*-hexane-EtOAc (3:1) as an eluent to furnish compound **S30** (2.3 g, quantitative) as a white solid. $[\alpha]_D$ -28.6 (*c* 1, MeOH). R_f (*n*-hexane-EtOAc, 3:1) 0.29; **^{1}H NMR** (500 MHz, CD_2Cl_2, rotA(*): rotB(^) = ~ 50:50): δ 7.06 (brd, *J* = 7.5 Hz, 4H, ArH_m for both rotamers), 6.80* (d, *J* = 7.5 Hz, 2H, ArH_o), 6.76^ (d, *J* = 7.5 Hz, 2H, ArH_o), 6.27 (brs, 2 H, ArOH for both rotamers), 4.05* (m, 1H, CHN), 3.94^ (m, 1H, CHN), 3.80-3.77* (m, 2H, CH_2O), 3.75^ (dd, *J* = 1.4 Hz, 9 Hz, CH_2O), 3.07* (d, *J* = 13.1 Hz, 1H, one proton from $ArCH_2$), 3.02^ (d, *J* = 13 Hz, 1H, one proton from $ArCH_2$), 2.62* (d, *J* = 13.1 Hz, 1H, one proton from $ArCH_2$), 2.60^ (d, *J* = 13.1 Hz, 1H, one proton from $ArCH_2$), 1.58* (s, 3H, one Me from $C(CH_3)_2$), 1.52 (brs, 21H, $C(CH_3)_3$ for both rotamers and one Me from $C(CH_3)_2$ of rotamerA), 1.47^ (s, 6H, $C(CH_3)_2$); **^{13}C NMR** (125 MHz, CD_2Cl_2): δ 155.5, 152.9,152.5, 131.2, 131.0, 130.8, 130.7, 116.0, 94.6, 94.3, 81.0, 80.3, 66.6, 66.4, 59.9, 59.8, 39.2, 38.2, 28.9, 27.9, 27.2, 24.9, 23.6. **IR (ATR, cm^{-1})** 3330, 2978, 2885, 1675. **ESI-HRMS:** Found $[M+Na]^+$ 330.1666, $C_{17}H_{25}NNaO_4$, requires 330.1676.

(*S*)-*tert*-butyl 4-(4-(2-methoxy-2-oxoethoxy)benzyl)-2,2-dimethyloxazolidene-3-carboxylate S31:

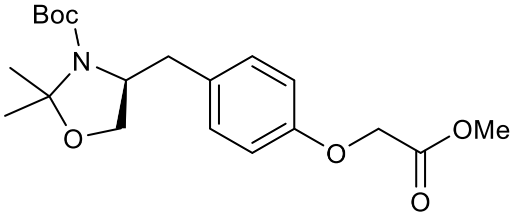

To a solution of **S30** (2.3 g, 7.5 mmol) in anhydrous THF (30 mL), sodium hydride (0.54 g, 22.5 mmol, 60% in mineral oil) was added at 0 °C. After stirring at the same temperature for 20 minutes, the solution was treated with TBAI (0.28 g, 0.75 mmol) followed by dropwise addition of methyl chloroacetate (1.64 mL, 18.7 mmol). The solution was stirred at room temperature for 12 h which resuted in the generation of a new spot just above the starting material, as indicated by TLC (*n*-hexane- EtOAc 5:2). Methanol (10 mL) was added to quench the reaction. The solvent was evaporated *in vacuo* and the residue obtained was dissolved in CH_2Cl_2 (30 mL) and washed successively with sodium thiosulphate (50 mL) and

water (50 mL). The organic layer was collected, dried over Na_2SO_4 and concentrated *in vacuo*. The crude product was then purified by flash chromatography using *n*-hexane-EtOAc (3:1) as an eluent to furnish compound **S31** (2.4 g, 83%) as a white solid. $[\alpha]_D$ -20.1 (*c* 1, MeOH). R_f (*n*-hexane-EtOAc, 5:2) 0.46; **^{1}H NMR** (500 MHz, CD_2Cl_2, rotA(*): rotB(^) = ~ 60:40): δ 7.16* (brd, *J* = 8.2 Hz, ArH_m), 7.13^ (brd, *J* = 8.2 Hz, ArH_m), 6.85 (d, *J* = 7.9 Hz, ArH_o for both rotamers), 4.61 (s, CH_2CO_2Me for both rotamers), 4.03* (m, CHN), 3.92^ (m, CHN), 3.77 (s, CO_2Me for both rotamers), 3.72 (dd, *J* = 1.2 Hz, 9 Hz, CH_2O for both rotamers), 3.07* (d, *J* = 13 Hz, one proton from $ArCH_2$), 3.02^ (d, *J* = 13 Hz, one proton from $ArCH_2$), 2.62 (m, one proton from $ArCH_2$ for both rotamers), 1.57* (s, one Me from $C(CH_3)_2$), 1.50 (brs, $C(CH_3)_3$ for both rotamers and one Me from $C(CH_3)_2$ of rotamerA), 1.45^ (s, $C(CH_3)_2$); **^{13}C NMR** (125 MHz, CD_2Cl_2): δ 169.9 (CO), 157.1, 152.6, 152.2, 132.6, 131.1, 131.0, 115.1, 115.0, 94.4, 94.0, 80.3, 79.9, 66.6, 66.4, 65.8, 59.8, 52.6, 39.3, 38.2, 28.8, 28.7, 27.9, 27.2, 24.9, 23.5. **IR (ATR, cm^{-1})** 2964, 2884, 1770, 1761, 1693, 1386, 1207, 1080. **ESI-HRMS:** Found $[M+Na]^+$ 402.1885, $C_{20}H_{29}NNaO_6$, requires 402.1887.

(*S*)-2-(4-((3-(*tert*-butoxycarbonyl)-2,2-dimethyloxazolidin-4-yl)methyl)phenoxy)acetic acid S32:

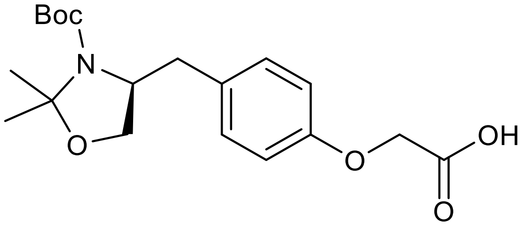

To the methanolic solution (15 mL) of compound **S31** (1.3 g, 3.5 mmol), KOH (0.38 g, 7 mmol) was added. The reaction was heated at 35 °C for 3 h, when TLC (*n*-hexane- EtOAc 1:1) confirmed conversion of starting material to a polar product present on the baseline. The solvent was evaporated and the crude residue dissolved in water (50 mL) and washed with diethyl ether (2×30 mL). The aqueous layer was collected and acidified with 6 N HCl dropwise. The desired carboxylic acid **S32** precipitated on standing and was filtered off and dried *in vacuo* to form a white solid (1 g, 80%). $[\alpha]_D$ -19.5 (*c* 1, MeOH). **^{1}H NMR** (500 MHz, CD_2Cl_2, rotA(*): rotB(^) = ~ 50:50): δ 7.15 (m, 4H, ArHm for both rotamers), 6.85 (t, *J* =7.6 Hz, 4H, ArH_o for both rotamers), 4.66 (s, 4H, CH_2CO_2Me for both rotamers), 4.04* (m, CHN), 3.93^ (m, CHN), 3.78-3.75* (m, 2H, CH_2O), 3.72^ (brd, *J* = 9 Hz, CH_2O), 3.08* (d, *J* = 13.2 Hz, 1H, one proton from $ArCH_2$), 3.05^ (d, *J* = 13.2 Hz, 1H, one proton from $ArCH_2$), 2.63* (d, *J* = 13.2 Hz, 1H, one proton from $ArCH_2$), 2.61^ (d, *J* = 13.2 Hz, 1H, one proton from $ArCH_2$), 1.57* (s, 3H, one Me from $C(CH_3)_2$), 1.50 (brs, 21H, $C(CH_3)_3$ for both rotamers and one Me from $C(CH_3)_2$ of rotamerA), 1.45t^ (s, 6H, $C(CH_3)_2$); **^{13}C NMR** (125 MHz, CD_2Cl_2): δ 172.7, 172.5, 156.8, 152.9, 152.3, 132.7, 132.6, 131.2, 131.1, 115.2, 115.1, 94.5, 94.2, 81, 80.2, 66.5, 66.3, 65.5, 59.8, 59.7, 39.2, 38.1, 28.8, 28.7, 27.8, 27.1, 24.8, 23.5. **IR (ATR, cm^{-1})** 3351, 2933, 1959, 1745, 1685, 1510, 1365, 1234, 1165, 1080. **ESI-HRMS:** Found $[M+Na]^+$ 388.1738, $C_{19}H_{27}NNaO_6$, requires 388.1731.

3-(2-(((methylamino)oxy)methyl)-1H-indol-1-yl)propanoic acid S33:

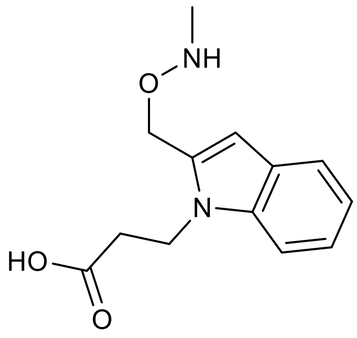

The compound was synthesized according to reported method [6] and the NMR was consistent with the literature:

^{1}H NMR (500 MHz, DMSO-d_6): δ 7.5 (d, *J* = 7.8 Hz, 1H, ArH), 7.47 (d, *J* = 8.3 Hz, 1H, ArH), 7.15-7.12 (m, 1H, ArH), 7.03-7.0 (m, 1H, ArH), 6.42 (s, 1H, ArH), 4.80 (s, 2H, CH_2O), 4.44 (t, *J* = 7.5 Hz, 2H, NCH_2), 2.71 (t, *J* = 7.5 Hz, 2H, CH_2CO_2H), 2.55 (s, 3H, NMe); **^{13}C NMR** (125 MHz, DMSO-d_6): δ 172.6 (CO_2H), 136.5, 136.0, 127.0, 121.5, 120.3, 119.2, 109.8, 102.7, 66.3 (ArC), 39.0 (NCH_2), 38.6 (NCH_3), 34.5 (CH_2CO_2H). **ESI-HRMS:** Found $[M+Na]^+$ 271.1049, $C_{13}H_{16}N_2NaO_3$, requires 271.1053.

Benzamidoxime S34:

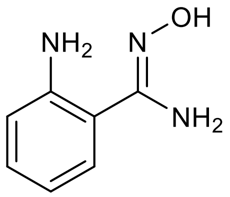

Synthesis of benzamidoxime **S34** was performed as previously reported[7]. To the ethanolic solution (40 mL) of 2-amino benzonitrile (2 g, 17 mmol) and hydroxylamine hydrochloride (1.3 g, 18.7 mmol), aqueous $NaHCO_3$ (1.71 g, 20.4 mmol) solution (12 mL) was added. The mixture was refluxed overnight, allowed to cool to room temperature, and diluted with 40 ml ethanol. The solid was filtered off and washed with cold ethanol (2 x 10 ml). All ethanol fractions were pooled and concentrated *in vacuo*. The crude solution was then purified by flash chromatography (DCM:MeOH 95:5) to give the pure benzamidoxime **S34** as a light orange, flaky solid (1.7 g, 65%). **^{1H} NMR** (400 MHz, DMSO-d_6): δ 9.57 (s, 1H), 7.37-7.34 (d, *J* = 7.79, 1H), 7.04-6.99 (t, *J* = 9.62 1H), 6.67-6.4 (d, *J* = 8.24 1H), 6.54-6.50 (t, *J* = 6.87 1H), 6.21 (br, 2H), 5.72 (br, 2H). **^{13}C NMR** (100 MHz, DMSO-d_6): 152.88, 146.79, 129.00, 127.29, 115.46, 114.85, 114.19. **ESI-HRMS**: Found $[M+H]^+$ 152.0817, $C_7H_{10}N_3O$, requires 152.0818.

Synthesis of OPAL modified dipeptide

To ascertain whether chiral organocatalysts under the conditions of the OPAL afford β-hydroxy protein aldehydes with stereochemical control we performed the OPAL on model dipeptide **S38**, bearing an existing stereocentre. Following exposure to OPAL conditions using **1** and **10**, the crude product was analysed and both HPLC and NMR analysis indicated the formation of four diastereomers **S40** in the ratio ~ 1 : 0.91: 0.89: 0.72 (consistent by both HPLC and ^{1}H-NMR). This model reaction suggests that under the aqueous reaction conditions described using chiral aldol acceptors, the use of chiral organocatalysts likely provides little steroechemical control over aldol bond formation.

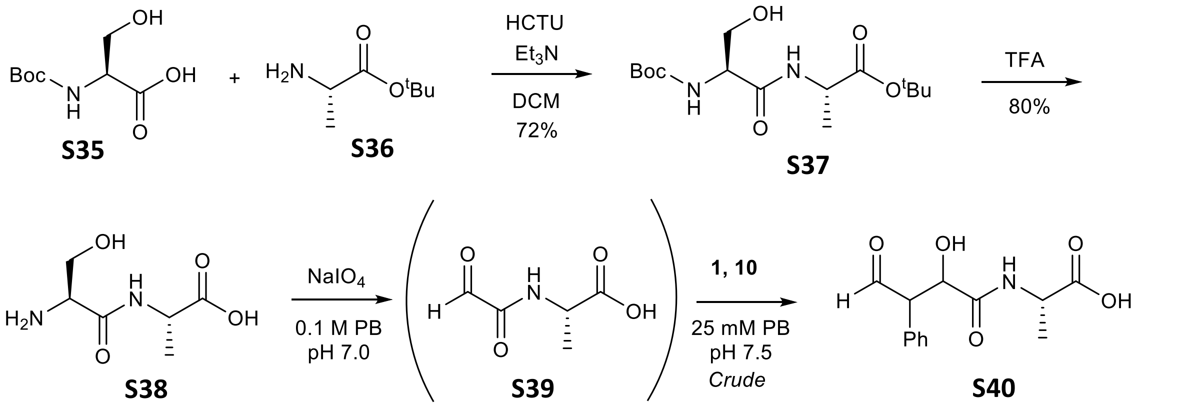

Supplementary Figure 24: Synthesis of OPAL modified dipeptide

(S)-*tert*-butyl2-((S)-2-((tert-butoxycarbonyl)amino)-3-hydroxypropanamido) propanoate S37

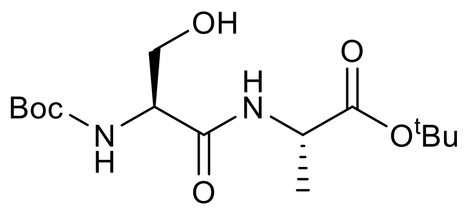

To a solution of Boc-L-serine **S35** (2 g, 9.7 mmol) in 20 mL of anhydrous CH_2Cl_2 was added L-alanine *tert*-butyl ester hydrochloride **S36** (2.1 g, 11.7 mmol), followed by the addition of TEA (2.7 mL, 19.5 mmol) and HCTU (4.8 g, 11.7 mmol). The reaction was stirred at rt overnight. Solvent was evaporated and the residue was dissolved in EtOAc (50 mL). The organic phase was washed with saturated citric acid (50 mL) and saturated aq. $NaHCO_3$ (50 mL) and then dried over anhydrous Na_2SO_4, filtered, and concentrated *in vacuo*. The residual crude product was purified by flash column chromatography (*n*-hexane-EtOAc 1:1) to afford the product **S37** as white solid (2.33 g, 72%). $[\alpha]_D$ -3.1 (*c* 1, CH_2Cl_2). **^{1}H NMR** (500 MHz, $CDCl_3$): δ 7.2 (d, J = 5.9 Hz, 1H, NHBoc), 5.69 (d, J = 7.4 Hz, 1H, NH), 4.4 (p, J_{H, CH_3}, $J_{H, NH}$ = 7.3 Hz, 1H, C*H*CH$_3$), 4.2 (brs, 1H, C*H*CH$_2$OH), 3.94 (d, J = 9 Hz, C*H*$_2$OH), 3.81 (brs, 1H, OH), 3.64 (brs, 1H, C*H*$_2$'OH), 1.42, 1.4 (2s, 18H, NHCO$_2$C((C*H*$_3$)$_3$), CO$_2$C((C*H*$_3$)$_3$), 1.34

(d, J = 7.2 Hz, CHCH_3). **^{13}C NMR** (125 MHz, $CDCl_3$): δ 172.1, 170.8, 155.9, 82.2, 80.2, 63.0, 55.2, 48.9, 28.2, 27.9, 17.9. **IR (ATR, cm^{-1})** 3312, 2681, 2167, 1666, 1599, 1530, 1449, 1396, 1356, 1296, 1250, 1165, 1060. **ESI-HRMS:** Found $[M+Na]^+$ 355.1838, $C_{15}H_{28}N_2NaO_6$, requires 355.1840.

(S)-2 -((S)-2-(amino-3-hydroxypropanamido) propanoic acid S38

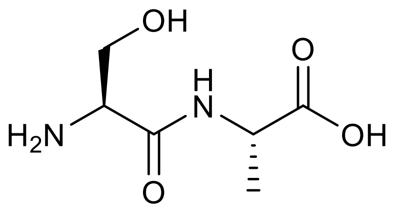

Compound **S37** (2 g, 6.02 mmol) was subjected to TFA:H_2O:TIPS (95:2.5:2.5) mixture (20 mL) and the solution was stirred at rt for 3 h. To it, cold diethyl ether was added which resulted in the formation of white precipitate. The precipitate was filtered and the white residue was lyophilized to afford the product **H** as white solid (850 mg, 80%). $[\alpha]_D$ -2.3 (c 1, MeOH). **^{1}H NMR** (500 MHz, D_2O): δ 4.33 (q, J = 7.4 Hz, 1H, CHCH$_3$), 4.04 (dd, J = 4.1 Hz, 6 Hz, 1H, CHCH$_2$OH), 3.93 (dd, J = 4.1 Hz, 12.5 Hz, CH_2OH), 3.85 (dd, 1H, J = 6 Hz, 12.5 Hz, CH_2'OH),1.34 (d, J = 7.4 Hz, CHCH_3). **^{13}C NMR** (125 MHz, D_2O): δ 176.1, 167.4, 60.1, 54.5, 48.9, 16.1. **IR (ATR, cm^{-1})** 3075, 1963, 1659, 1555, 1459, 1432, 1186, 1132. **ESI-HRMS:** Found $[M+Na]^+$ 199.0687, $C_6H_{12}N_2NaO_4$, requires 199.0689.

(2,4,4-trihydroxy- 3-phenylbutanoyl)-L-alanine S40

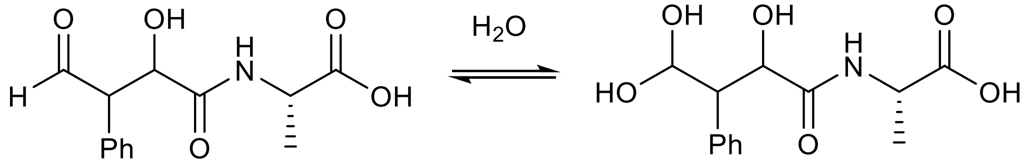

To a solution of compound **S38** (0.05 g, 0.27 mmol) in 0.1 M PB pH 7.0 (700 µL) was added $NaIO_4$ (0.06 g, 0.28 mmol). The reaction was mixed until complete dissolution was achieved, and then allowed to sit at rt in the dark for 45 min**.** Complete oxidation of **S38** to **S39** was observed by LC-MS analysis To this solution, L-proline **1** (0.006 g, 0.05 mmol) and phenylacetaldehyde **10** (0.031 mL, 0.032 g, 0.027 mmol) were added. The reaction mixture was mixed thoroughly, and then allowed to sit at 37 °C for 1 hour. Conversion to the desired aldol product **S40** was monitored by LC-MS. The solvent was removed *in vacuo*, and the residue resuspended in ethyl acetate, resulting in the precipitation of L-proline **1** which was filtered off. The filtrate was evaporated *in vacuo* and the crude reaction mixture was subjected to HPLC, LC and NMR analysis. LC-MS confirmed full conversion of the starting material to the aldol product. HPLC and ^{1}H-NMR of **S40-Hyd** indicates presence of 4 diastereomers in the ratio a:b:c:d = 1: 0.91: 0.89: 0.72 (as obtained from relative area values from HPLC).

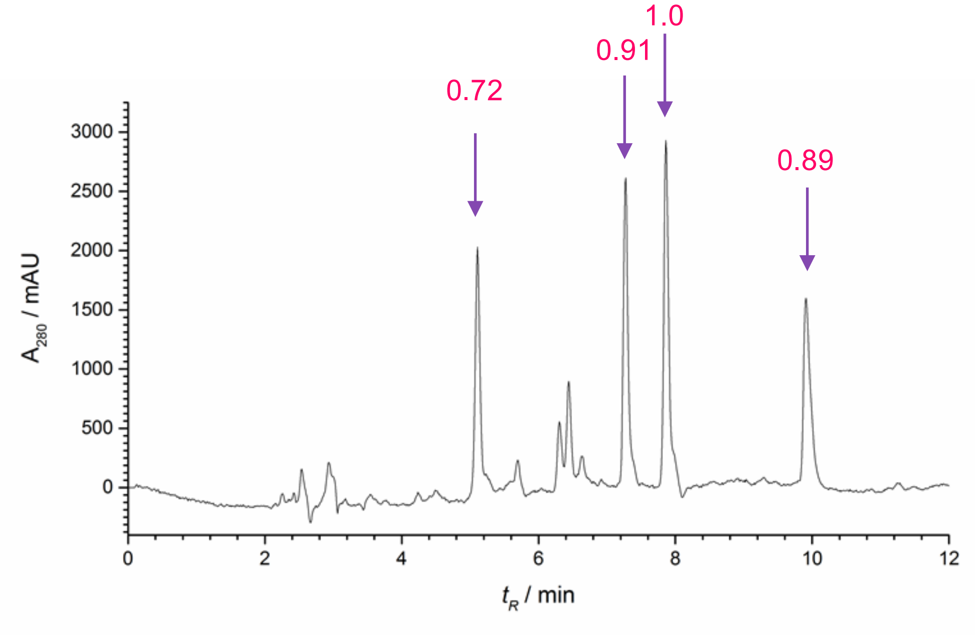

Supplementary Figure 25: Analytical HPLC of the crude aldol product (Kinetex phenyl hexyl 100 A), eluents A = H_2O + 0.1% formic acid; B = MeCN + 0.1% formic acid, gradient : 5% B (0 min) → 20% B (in 12 min) at Temperature 55 °C with the flowrate of 1 mL/min

^{1}H NMR (500 MHz, DMSO-d6): δ 7.40-7.08 (m, ArH), 5.35-5.25 (m, C*H*(OH)$_2$), 4.65 (d, *J* = 7.6 Hz, H-2d), 4.60 (d, *J* = 10.3 Hz, H-2b), 4.55 (d, *J* = 10.2 Hz, H-2c), 4.51 (d, *J* = 7.6 Hz, H-2a), 4.52 (q, *J* = 7.65 Hz, C*H*aCH$_3$), 4.47 (q, *J* = 7.65 Hz, C*H*bCH$_3$), 4.35 (q, *J* = 7.6 Hz, C*H*cCH$_3$), 4.22 (q, *J* = 7.6 Hz, C*H*dCH$_3$), 3.41 (d, *J* = 7.65 Hz, H-3d), 3.3 (dd, *J* = 1.9 Hz, 7.7Hz, H-3a), 3.23 (dd, *J* = 4.8 Hz, 10.3 Hz, H-3b), 3.06 (dd, *J* = 4.7 Hz, 10.2 Hz, H-3c), 1.4 (d, *J* = 7.6 Hz, CHCH$_3$d), 1.37 (d, *J* = 7.6 Hz, CHCH$_3$c), 1.32 (d, *J* = 7.6 Hz, CHCH$_3$b), 1.25 (d, *J* = 7.6 Hz, CHCH$_3$a). **^{13}C NMR** (125 MHz, DMSO-d6): δ 174.4, 173.8,173.4, 172.2, 171.8, 171.7, 170.4, 170.1 (C=O), 131.3-125.6 (ArC), 85.5, 84.0, 83.5, 80.4, 74.1, 73.9, 70.7, 69.8, 69.5, 59.8, 57.8, 57.3, 57.1, 53.5, 52.9, 50.9, 50.6, 50.3, 49.7, 30.7, 20.8, 16.6, 16.5, 14.7, 14.1 (CH*C*H$_3$). **ESI-LRMS:** Found $[M+H]^+$ 265.89, $C_{13}H_{13}NO_5$, requires 266.10.

Palmitoyl phthalimide S41

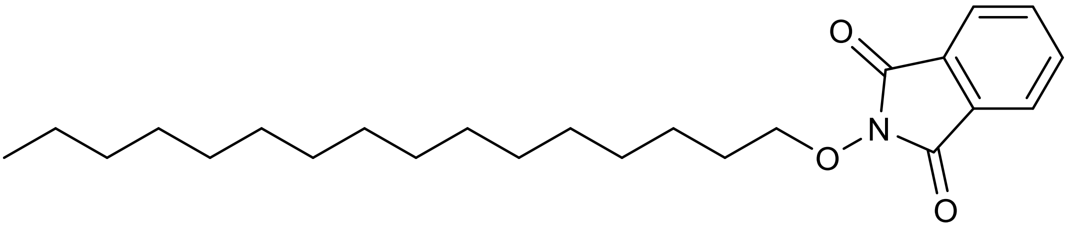

Synthesis of this compound was adapted from a protocol previously described for the synthesis of alkyl phthalimides[8]. To a stirred solution of cetyl alcohol (1.19 g, 4.90 mmol), N-

hydroxyphthalimide (0.96 g, 5.88 mmol), and PPh_3 (1.70 g, 6.47 mmol) in THF (17 mL) was added DIAD (1.27 mL, 5.88 mmol). The solution was stirred under a nitrogen atmosphere at room temperature overnight. The solvent was removed in vacuo and the resultant white powder was dissolved in hexane and filtered. The solvent was removed in vacuo to give compound **S41** as a white powder (0.61 g, 32%). **^{1}H NMR** (500 MHz, $CDCl_3$): δ 7.83 , 7.74 (2d, 4H, *J* = 3.1 Hz, ArH), 4.19 (t, 2H, *J* = 6.8 Hz, OCH_2), 1.78 (p, 2H, *J* = 6.9 Hz, CH_2), 1.47 (p, 2H, *J* = 6.9 Hz, CH_2), 1.25 (bs, 24H, CH_2), 0.87 (t, 1H, *J* = 6.9 Hz, CH_3). **^{13}C NMR** (125 MHz, $CDCl_3$): δ 163.6 (2) (C=O), 134.4 (2), 128.9 (2), 123.4 (2) (ArC), 78.6 (OCH_2), 31.9, 29.7 (2), 29.64, 29.63, 29.61, 29.5, 29.4, 29.3, 29.2, 28.1, 25.5, 22.7 (all CH_2), 14.1 (CH_3). **ESI-HRMS:** Found $[M+Na]^+$ 410.2662, $C_{24}H_{37}NNaO_3$, requires 410.2666.

Palmitoyl aminooxy 32

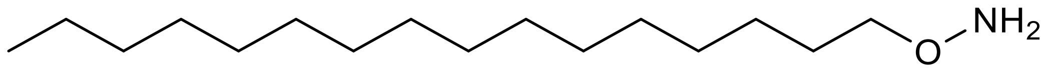

To a solution of **S41** (0.039 g, 0.151 mmol) in DCM (1 ml) was added hydrazine monohydrate (76 µl, 2.47 mmol). The solution was stirred vigorously for 45 minutes, during which time a white solid appeared. The solution was filtered through cotton wool, and the filtrate was collected. The resulting filtrate then concentrated under a stream of nitrogen to give **S41** as a white solid in quantitative yield that was used without further purification. **^{1}H NMR** (500 MHz, $CDCl_3$): δ 3.64 (t, 2H, *J* = 6.7 Hz, OCH_2), 1.56 (p, 2H, *J* = 6.8 Hz, CH_2), 1.25 (bs, 26H, CH_2), 0.87 (t, 1H, *J* = 6.9 Hz, CH_3). **^{13}C NMR** (125 MHz, $CDCl_3$): δ 76.2 (OCH_2), 31.9, 29.67 (2), 29.65 (2), 29.6 (2), 29.57, 29.56, 29.5, 29.3, 28.4, 26.0, 22.7 (all CH_2), 14.1 (CH_3). **ESI-HRMS:** Found [M+H]+ 258.2793, $C_{16}H_{36}NO$, requires 258.2791

PEG2K phthalimide S42

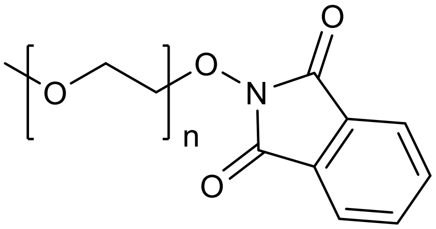

(2kDa)

Synthesis of this compound was performed as previously reported[9]. Under an atmosphere of nitrogen, a solution of poly(ethylene glycol) monomethyl ether, average molecular weight 2000 g mol^{-1} (2.00 g, 0.994 mmol), *N*-hydroxyphthlimide (194 mg, 1.19 mmol), and PPh_3 (312 mg, 1.19 mmol) in DCM (10 ml) was charged with diisopropyl azodicarboxylate (212

µL, 1.09 mmol) via dropwise addition. The reaction mixture was then allowed to stir under nitrogen for 18 h at room temperature. The solution was then directly added to 400 ml of diethyl ether, and the suspension was stirred vigorously for 20 min. The suspension was filtered, and the resulting solid was washed with diethyl ether (3 x 70 ml), and residual solvent was removed *in vacuo*. The dry solid was then subjected to the same procedure a second time to give the product as a white powder that was used without further purification (1.5 g, 75%). ^{1}**H NMR** (500 MHz, $CDCl_3$): δ 7.82, 7.73 (2d, 4H, *J* = 3.1 Hz, ArH), 3.63 (bs, 3H, OCH_2), 3.62 (bs, 165 H, OCH_2), 3.36 (s, 3H, OCH_3). ^{13}**C NMR** (125 MHz, $CDCl_3$): δ 163.4 (2) (C=O), 134.4 (2), 128.9 (2), 123.4 (2) (ArC), 71.8, 70.5 (OCH_2), 58.9 (OCH_3).

PEG2K aminooxy 32

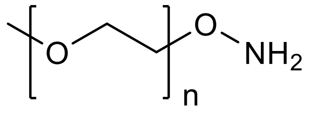

(2kDa)

Synthesis of this compound was performed as previously reported[9]. To a solution of **S42** (1.00 g, 0.494 mmol) in DCM (10 ml) was added hydrazine hydrate (76 µL, 2.47 mmol). The solution was stirred vigorously for 30 min, during which time a white solid appeared. The solution was filtered through cotton wool, and the filtrate was collected. The resulting filtrate was then concentrated under a stream of nitrogen to give **32** as a white solid in quantitative yield that was used without further purification. ^{1}**H NMR** (500 MHz, $CDCl_3$): δ 3.43 (bs, 168 H, OCH_2), 3.16 (s, 3H, OCH_3). ^{13}**C NMR** (125 MHz, $CDCl_3$): δ 69.9 (OCH_2), 58.3 (OCH_3)

4. Solid Phase Peptide Synthesis (SPPS) and donor synthesis

Peptides were synthesised via manual solid phase peptide synthesis (SPPS) using an *in situ* neutralisation/HCTU activation procedure for Fmoc chemistry on an H-Gly-2-ClTrt resin (Sigma) using Fmoc protected amino acids as described below:

Preloaded resin preparation. The preloaded 2-chlorotrityl resin was weighed out into a 2 mL SPPS cartridge fitted with a PTFE stopcock, swollen in DMF for 30 min and then filtered.

Amino acid coupling. DIPEA (11.0 eq.) was added to a solution of amino acid (5.0 eq.) and HCTU (5.0 eq.) dissolved in the minimum volume of DMF and the solution added to the resin. The reaction mixture was gently agitated by rotation for 1 h, and the resin filtered off and washed with DMF (3 × 2 min with rotation).

Fmoc deprotection. A solution of 20% piperidine in DMF was added to the resin and gently agitated by rotation for 2 minutes. The resin was filtered off and repeated four more times, followed by washes with DMF (5 × 2 min with rotation).

Cleavage and Isolation. Resins containing full synthesised peptides were washed with DCM (3 × 2 min with rotation) and MeOH (3 × 2 min with rotation). The resin was dried on a vacuum manifold and further dried on a high vacuum line overnight. A solution of cleavage cocktail 95:2.5:2.5 (v/v) TFA:H_2O:triisopropylsilane was then added to the resin, and the resulting mixture was gently agitated by rotation for 60 min. The reaction mixture was drained into ice-cold Et_2O and centrifuged at 6000 rpm at 4 °C until pelleted (*ca*. 5-10 min). The supernatant was carefully decanted and subsequently resuspended, centrifuged and supernatant decanted three more times. The precipitated peptide pellet was then either dissolved 10% MeCN or in 10% aq. AcOH and lyophilised. Lyophilised peptides were then stored at -20 ^{o}C until required.

Notes on folate containing peptides

For designing peptides containing lysine modified at the N_ε position with folic acid, Fmoc-Lys (Dde)-OH was incorporated into the peptide chain as described above. Upon synthesising the desired peptide chain, and prior to **Cleavage and Isolation**, the resin bound peptide was treated with $NH_2NH_2.H_2O$ (2% in DMF) and gently agitated by rotation for 5 min. This process was repeated, and the resin bound peptide was washed with DMF (3 × 2 min with rotation). A solution of folic acid (2.5 eq), HCTU (2.5 eq.), and DIPEA (5.0 eq.) in 1:1 DMSO:DMF was then added to the resin, and the resulting mixture was gently agitated by rotation for 8 h. The resin was then filtered off, washed with DMF (9 x 2 min with rotation), and the desired peptide was then obtained following the **Cleavage and Isolation** step mentioned prior. The desired peptide was then further purified via size-exclusion chromatography (Sephadex LH-20 in water), and fractions containing pure, desired peptide were lyophilised and stored at -20 ^{o}C until required.

Synthesis of SLYRAG S43

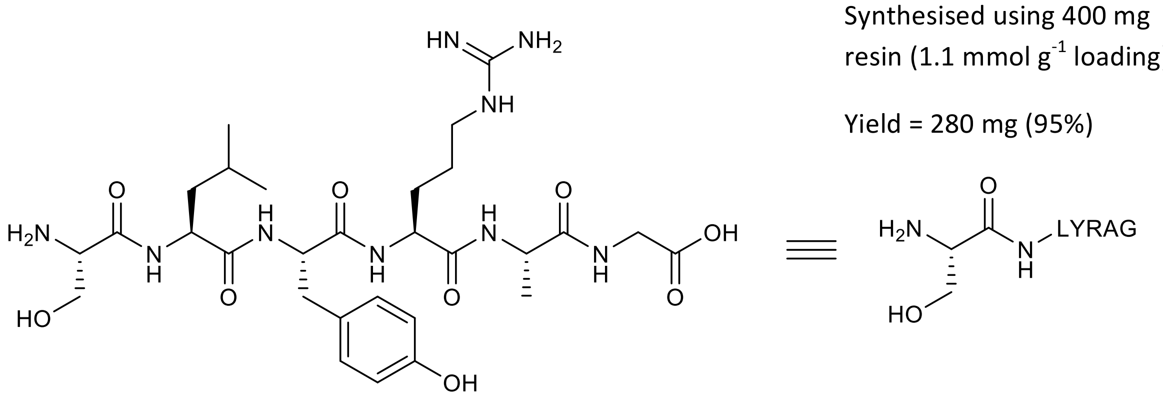

Synthesised using 400 mg resin (1.1 mmol g^{-1} loading)

Yield = 280 mg (95%)

SLYRAG was synthesised as previously described[10].

Synthesis of fluorescent label precursor S44

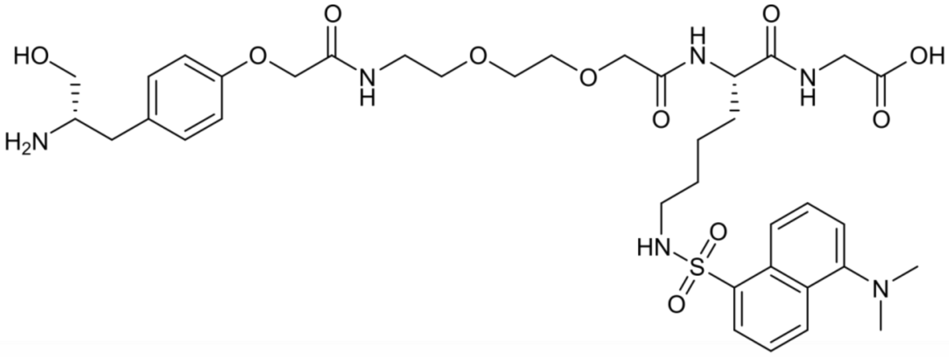

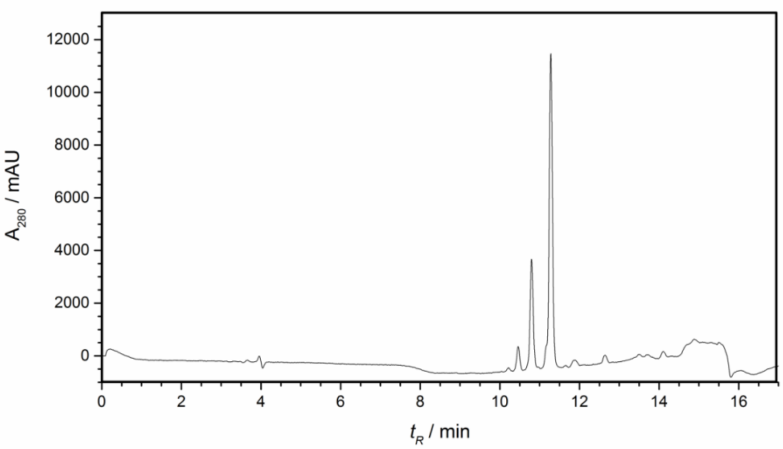

Synthesised using 85 mg resin (0.54 mmol g^{-1} loading)

Yield = 33 mg (92%)

HRMS: Found $[M+H]^+$ 789.3511, $C_{37}H_{53}N_6O_{11}S$, requires 789.3488. **HPLC**:t_R 11.28 min.

Synthesis of fluorescent label 11

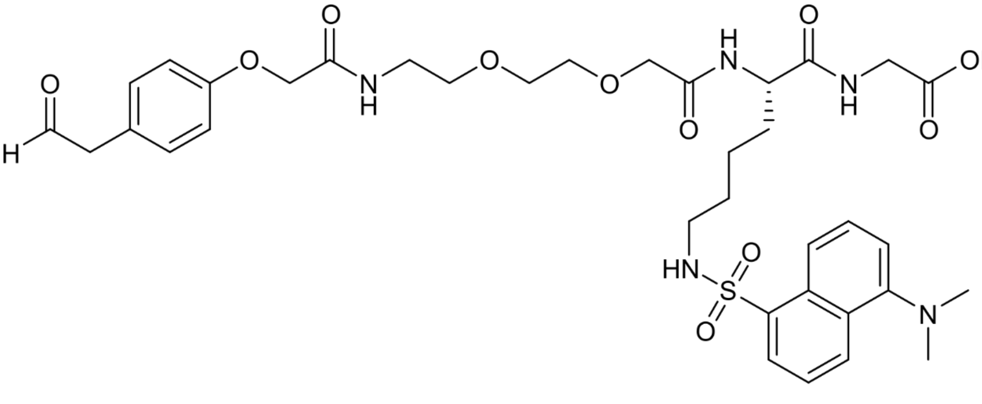

To a solution of **S44** (10 mg in 500 µL, 10 mM, 0.1 M PB, 0.1 M NaCl pH 7.0) was added methionine (250 µL, 200 mM, 0.1 M PB, 0.1M NaCl pH 7.0) and $NaIO_4$ (210 µL, 112 mM, 0.1 M PB, 0.1 M NaCl pH 7.0). The reaction was mixed thoroughly, and allowed to sit for 2 min on ice in the dark. The solution was then loaded onto a solid phase extraction cartridge (Grace Davison Extract Clean, 8 ml reservoir, Fisher Scientific) equilibrated with water/acetonitrile. After initial washing with water, the product was eluted over a gradient of acetonitrile. The product was then diluted with water, and subsequently lyophilised to give **11** as a pale yellow, fluffy powder (4 mg, 40%). **LRMS**: Found $[M+H]^+$ 758.34, $C_{36}H_{48}N_5O_{11}S$, requires 758.34.

Synthesis biotin affinity tag precursor S45

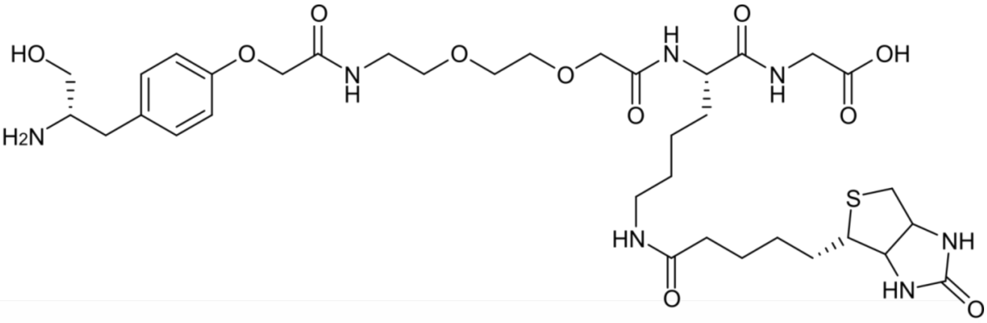

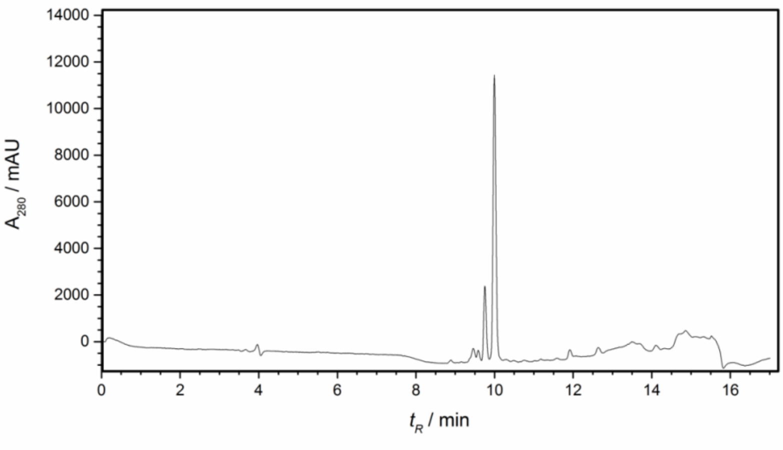

Synthesised using 100 mg resin (0.54 mmol g^{-1} loading)

Yield = 41 mg (98%)

HRMS: Found $[M+H]^+$ 782.3781, $C_{35}H_{56}N_7O_{11}S$, requires 782.3753. **HPLC**: t_R 9.99 min.

Synthesis of biotin affinity tag 12

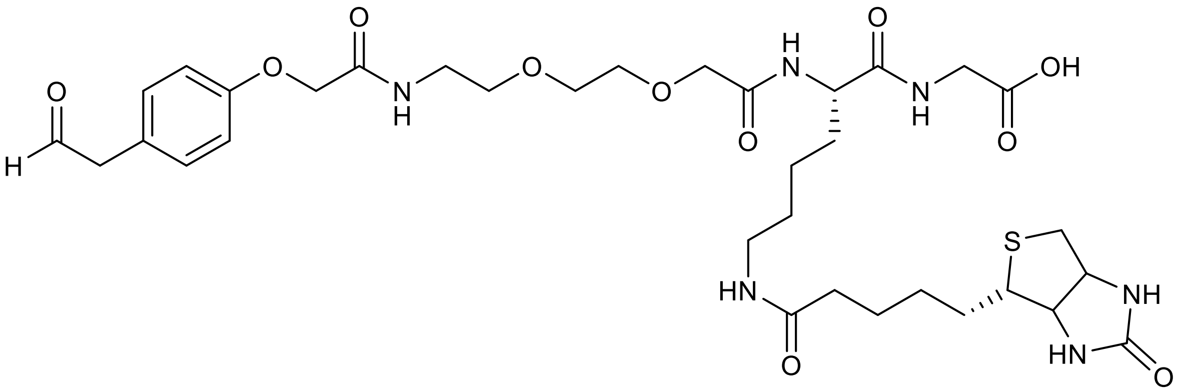

To a solution of **S45** (10 mg in 500 µL, 10 mM, 0.1 M PB, 0.1 M NaCl pH 7.0) was added methionine (250 µL, 200 mM, 0.1 M PB, 0.1M NaCl pH 7.0) and $NaIO_4$ (210 µL, 112 mM, 0.1 M PB, 0.1 M NaCl pH 7.0). The reaction was mixed thoroughly, and allowed to sit for 2 min on ice in the dark. The solution was then loaded onto a solid phase extraction cartridge (Grace Davison Extract Clean, 8 ml reservoir, Fisher Scientific) equilibrated with water/acetonitrile. After initial washing with water, the product was eluted over a gradient of acetonitrile. The product was then diluted with water, and subsequently lyophilised to give **12** as a white, fluffy powder (9 mg, 84%). **LRMS**: Found $[M+H]^+$ 751.39, $C_{34}H_{51}N_6O_{11}S$, requires 751.40.

Synthesis of folate targeting moiety precursor S46

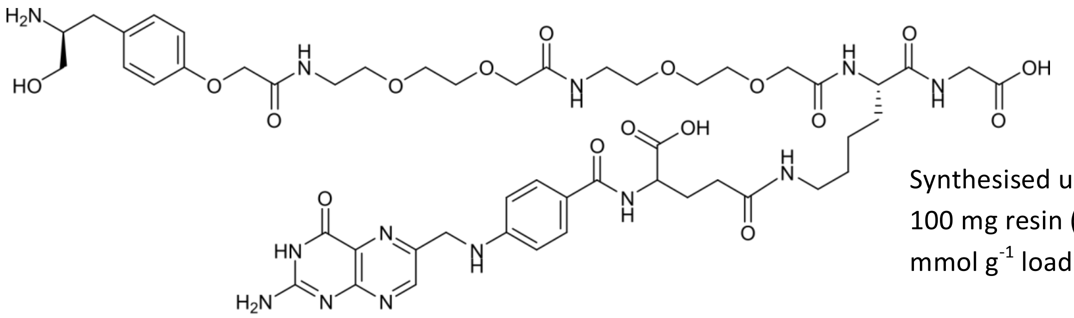

Synthesised using 100 mg resin (0.54 mmol g^{-1} loading)

Yield = 11 mg (18%)

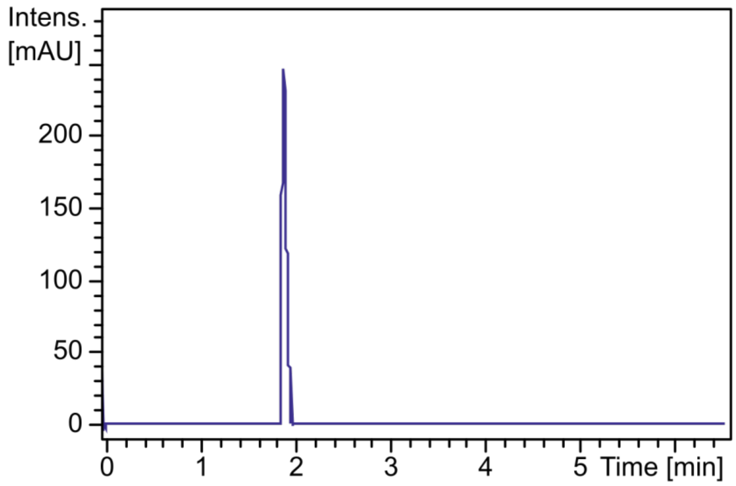

Note: HPLC analysis of ***S46*** *was instead performed using the* ***'LC-MS analysis of peptide and protein ligations'*** *method as described previously for peptide analysis.*

HRMS: Found $[M+H]^+$ 1124.5037, $C_{50}H_{70}N_{13}O_{17}$, requires 1124.5007. **HPLC**:t_R 1.9 min.

Synthesis of folate targeting moiety 13

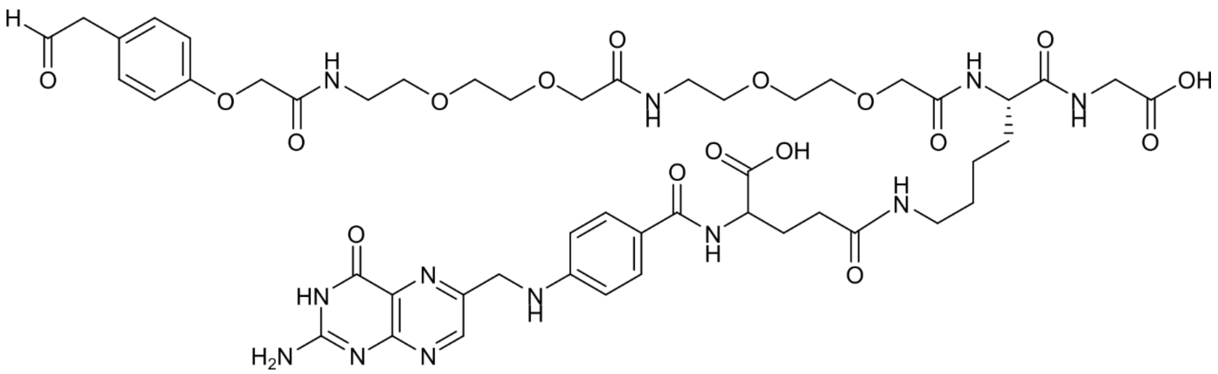

To a solution of **S46** (10 mg in 500 µL, 9 mM, 0.1 M PB, 0.1 M NaCl pH 7.0) was added methionine (250 µL, 200 mM, 0.1 M PB, 0.1M NaCl pH 7.0) and $NaIO_4$ (210 µL, 112 mM, 0.1 M PB, 0.1 M NaCl pH 7.0). The reaction was mixed thoroughly, and allowed to sit for 2 min on ice in the dark. The solution was then loaded onto a solid phase extraction cartridge (Grace Davison Extract Clean, 8 ml reservoir, Fisher Scientific) equilibrated with water/acetonitrile. After initial washing with water, the product was eluted over a gradient of acetonitrile. The product was then diluted with water, and subsequently lyophilised to give **13** as a yellow, fluffy powder (3 mg, 31%). **LRMS**: Found $[M+2H]^{2+}$ 547.33, $C_{49}H_{66}N_{12}O_{17}$, requires 547.72.

Synthesis of bioorthogonal azide handle precursor S47

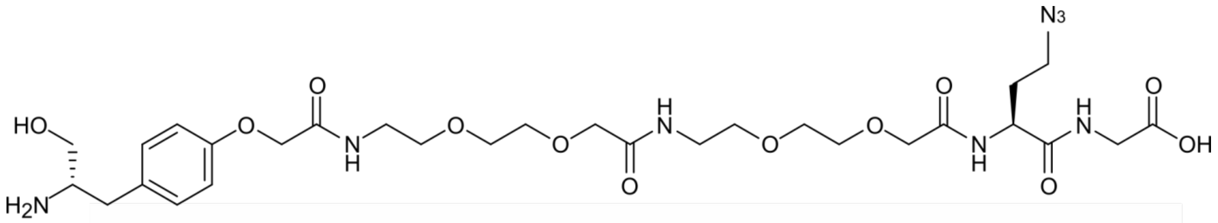

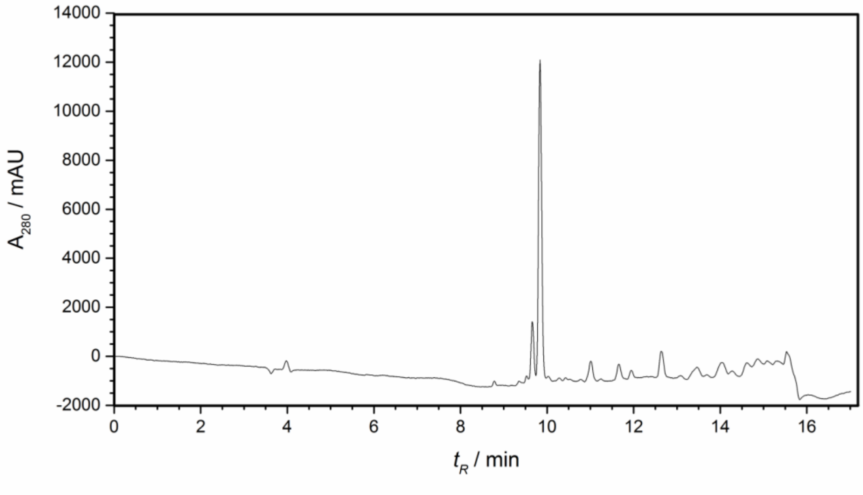

Synthesised using 100 mg resin (0.54 mmol g^{-1} loading)

Yield = 32 mg (84%)

HRMS: Found $[M+H]^+$ 699.3322, $C_{29}H_{47}N_8O_{12}$, requires 699.3308. **HPLC**:t_R = 9.83 min.

Synthesis of bioorthogonal azide handle 14

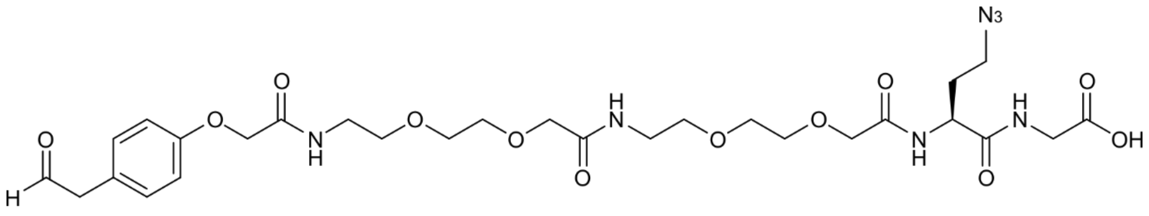

To a solution of **S47** (10 mg in 500 µL, 10 mM, 0.1 M PB, 0.1 M NaCl pH 7.0) was added methionine (250 µL, 200 mM, 0.1 M PB, 0.1M NaCl pH 7.0) and $NaIO_4$ (210 µL, 112 mM, 0.1 M PB, 0.1 M NaCl pH 7.0). The reaction was mixed thoroughly, and allowed to sit for 2 min on ice in the dark. The solution was then loaded onto a solid phase extraction cartridge (Grace Davison Extract Clean, 8 ml reservoir, Fisher Scientific) equilibrated with water/acetonitrile. After initial washing with water, the product was eluted over a gradient of acetonitrile. The product was then diluted with water, and subsequently lyophilised to give **14** as a pale green, fluffy powder (8 mg, 80%). **LRMS**: Found $[M+Na]^+$ 690.29, $C_{28}H_{41}N_7NaO_{12}$, requires 690.27.

Notes on chemical probes and storage

Protected probes **S44-S47** and chemical probes **11-14** can be stored long term as lyophilised powders at -20 °C. The lyophilised chemical probes **11-14** are highly water soluble and can be stored as 50 mM stock solutions in water at -20 °C for over 3 months (typically as 5 µL aliquots). Stock solutions in this form can be defrosted and used when required. Throughout this work, defrosted stock solutions of probes **11-14** were kept at 4°C and were typically used within four days for bioconjugation reactions. We have noted that, after 3 months of storage in solution at -20 °C, a minor decrease in reactivity of probes **11-14** towards protein modification may be observed. Probes stored for longer than 6 months in solution at -20 °C could still be successfully used for site-selective protein modification, but these procedures may require higher concentrations of probe to achieve complete conversion to the desired protein bioconjugates after 1 h of incubation at 37 °C. We therefore recommend that, if incomplete conversion to the desired modified protein is noted for a given bioconjugation, an additional 0.5-1 mM of probe is added to a given bioconjugation reaction, and allowed to react for a further 30 min at 37 °C.

5. Protein expression and purification

Expression of GFP containing cylooctyne-lysine at position 39

The pBAD construct containing Ser-GFP(Y39TAG)-His6 and the pEVOL pylRS AF[5], were co-transformed into One Shot™TOP10 Electrocomp™*E.coli* (Invitrogen) by electroporation and selected on LB agar with ampicillin (100 µg/mL) and chloramphenicol (35 µg/mL). Starter cultures were prepared by picking single clones into LB with ampicillin (100 µg/mL) and chloramphenicol (35 µg/mL), and grown at 37 °C for 16 h with shaking (220 rpm). For protein expression, Terrific Broth Medium (50 mL) was inoculated with 0.5 mL of starter culture and the culture was grown to an OD600nm of 0.2-0.3, at 37 °C with shaking (220 rpm). Unnatural amino acid cyclooctyne-lysine [stock solution 250 mM in 0.1M NaOH (aq.)] was added to a final concentration of 5 mM. The cultures were allowed to grow to an OD600nm of 0.4-0.6, at which point protein expression was induced by addition of L-arabinose to a final concentration of 0.02% (w/w). After further growth for 4.5 h (37 °C, 220 rpm), the cultures were harvested by centrifugation (6 000 × g, 10 min). Pellets were resuspended in lysis buffer (4 × PBS, pH 8.0, 10 mM imidazole, Pierce Protease Inhibitor tablet, EDTA-free) and lysed by sonication on ice for 6 × 30 s, with 30 s intervals. The lysate was clarified by centrifugation (20 000 × g, 4 °C, 20 min) and loaded onto a HisTrap HP column (5 mL, GE Healthcare) pre-equilibrated in binding buffer (4 × PBS, pH 8.0, 10 mM imidazole). After washing the column with 10 column volumes (cv) of binding buffer, GFP was eluted via a linear gradient of 0-100% elution buffer (4 × PBS, pH 8.0, 500 mM imidazole) over 7.5 cv. Fractions containing full-length protein (as determined by SDS-PAGE) were pooled, dialysed into 1 × PBS, pH 7.4 and concentrated (Vivaspin centrifugal concentrator, 10 000 MWCO) to a final concentration of 330 µM (as determined by UV-visible spectroscopy, $\varepsilon_{280} = 2.0 \times 10^4$ dm³ mol^{-1} cm^{-1}). Proteins were stored at -80 °C.

Expression of *Leishmania major* N-myristoyltransferase

The *Leishmania major* N-myristoyltransferase (NMT) was expressed and purified as previously described[11].

Generation of the HASPA G1S protein expression construct

Upon expression of HASPA proteins, the *N*-terminal Met is removed to generate an amino-terminal Gly and a substrate for *N*-myristoyltransferase. This Gly residue is designated G1 in all HASPA proteins expressed in this work.

The primers HASPLD (5'-TATACCATGGGAGCCTACTCTACGAAGGACTCCGCAAAGG-3') and HASPB3 (5'-TATACTCGAGGTTGCCGGCAGCGTGCTCCTTC-3') were used to amplify by polymerase chain reaction (PCR) the HASPA coding sequence from genomic DNA template isolated from *L. donovani* strain MHOM/ET/67/L28 using KOD polymerase. The ~250 bp PCR product was purified, treated with the restriction endonulceases *NcoI* and *XhoI* and the cleavage products were ligated to *NcoI-XhoI* treated pET28a plasmid vector. The ligation products were introduced into chemically competent *E.coli* NovaBlue (Novagen) cells by heat shock and selected on LB + Kanamycin (50 µg/mL). The plasmid DNA was isolated and sequenced to confirm the presence of the expected insert. The resulting plasmid, HASPA_C4S_pET28a encodes *L. donovani* HASPA with a Cys to Ser substitution at

position 4 (for improved solubility), with a C-terminal His_6 tag. Site directed mutagenesis (QuikChange Lightning 2 kit- Agilent Technologies) with primers MP1 (AGTCCTTCGTAGAGTAGGCGCTCATGGTATATCTCCTTCTT) and MP2 (AAGAAGGAGATATACCATGAGCGCCTACTCTACGAAGGACT) was carried out on HASPA_C4S_pET28a to introduce a Gly to Ser substitution at position 1 in the *L. donovani* HASPA protein sequence. The mutations in the resulting plasmid, HASPA_G1S_C4S_pET28a were confirmed by DNA sequencing.

Expression of 15N labelled and unlabelled G1S HASPA.

The HASPA_G1S_C4S_pET28a construct was introduced into electrocompetent E. coli BL21(DE3) cells by electroporation and selected on LB agar with kanamycin (50 µg/mL) at 37 °C for 16 h. Starter cultures were prepared by picking single clones into LB with kanamycin (50 µg/mL) and grown at 37 °C for 8 h with shaking (180 rpm). For the expression of the 15N labelled G1S HASPA, M9 minimal medium was used. M9 minimal medium consisted of Na_2HPO_4, 6 g/L; KH2PO4, 3 g/L; NaCl, 0.5 g/L; 15NH4Cl or NH4Cl, 1 g/L; supplemented with 0.2% (w/v) glucose; MgSO4, 1 mM; CaCl2, 0.1 mM; MnCl2, 0.1 mM, ZnSO4, 0.05 mM; FeCl3, 0.05 mM and 2 mL/L of vitamin solution. Vitamin solution consisted of 125 mg of thiamine, 2.5 mg of riboflavin and 25 mg of each of the following: pyridoxine, biotine, panthothenate, folic acid, choline chloride and nicotinamide. Unlabelled M9 minimal medium (50 mL) with kanamycin (30 µg/mL) was inoculated with the starter culture to an OD600nm of 0.05 and the culture was incubated 37 °C for 16 h with shaking (180 rpm). 15N labelled M9 minimal medium (1 L) was inoculated with the culture grown in unlabelled M9 medium to an OD600nm of 0.05, and grown at 37 °C with shaking (180 rpm) to an OD600nm of ~ 0.8. Isopropyl β-D-1- 57 thiogalactopyranoside (IPTG) was added to a final concentration of 1 mM and the cells were grown at 30 °C for 6 h with shaking (180 rpm). For the expression of the unlabelled G1S HASPA, 1 L of LB with kanamycin (50 µg/mL) was inoculated with 1 mL of starter culture and grown at 37 °C with shaking (180 rpm) to an OD600nm of ~ 0.6. IPTG was added to a final concentration of 0.3 mM and the cells were grown at 30 °C for 6 h with shaking (180 rpm).

Purification of 15N labelled and unlabelled G1S HASPA.

The 15N labelled and unlabelled recombinant G1S HASPA proteins, both with a C-terminal His6-tag were purified using a two-step purification procedure. Cells were harvested by centrifugation (6000 x g, 6 °C, 15 min) and the pellet was resuspended in lysis buffer (50 mM Tris pH 7.5, 500 mM NaCl, 20 mM imidazole and PierceTM Protease Inhibitor Tablet EDTA-free) and loaded onto a HisTrapTM HP column (5 mL) pre-equilibrated with binding buffer (50 mM Tris pH 7.5, 500mM NaCl mM NaCl, 20 mM imidazole). After washing the column with 16 column volumes of binding buffer, the recombinant HASPA was eluted with elution buffer (50 mM Tris pH 7.5, 500 mM NaCl, 500 mM imidazole). Target protein containing fractions were identified after analysis by SDS-PAGE and concentrated (Vivaspin Protein Concentrator Spin Column, 3000 MWCO). The concentrated protein sample was further purified by gel filtration chromatography (Superdex 75 10/300 column) in gel filtration buffer (20 mM HEPES pH 6.5, 50 mM NaCl). The final sample purity was assessed by SDS-PAGE. Despite its predicted molecular mass of 9606.88 Da, the C-terminal His6-tagged G1S HASPA ran higher on the SDS-PAGE gel than was expected for its relative molecular weight

(Supplementary Figure 26). This is due to the net negative charge of the protein and has been observed previously. Protein concentration was assayed by using the OPA method (described below, see Supplementary Figure 27). The yields of the 15N labelled and unlabelled HASPA proteins were ~ 8 mg/ L and 10 mg/L respectively. The proteins were stored at -80 °C.

Determination of G1S HASPA protein concentration.

Due to having only a single aromatic amino acid residue (tyrosine), the extinction coefficient of HASPA is unacceptably low to enable accurate protein quantitation by measuring the absorbance at 280 nm. Additionally, HASPA is highly hydrophilic and therefore does not react with Bradford reagent. The concentration of the ^{15}N labelled and unlabelled G1S HASPA was measured using O-Phthaldialdehyde (OPA) reagent, a primary amine-reactive fluorescent detection reagent. Protein concentration is determined by comparison to a bovine serum albumin (BSA) standard curve. The linear range of the assay is 10 to 500 µg/mL. Standards were prepared at 50, 100, 200, 300, 400 and 500 µg/mL using BSA in gel filtration buffer. The protein sample to be tested was diluted in gel filtration buffer to 1:10, 1:100 and 1:1000; enabling a range of concentrations to be covered. OPA reagent (Sigma-Aldrich) was regenerated by adding 2.5 µL/mL of β-mercaptoethanol. 20 µL of sample or standard was added to 200 µL of OPA reagent in a microtitre plate. After incubation at room temperature for 90 s, a reading of fluorescence was taken by scanning with a 355 nm, 40 nm bandwidth excitation filter and a 460 nm, 40 nm bandwidth emission filter, using an Infinite M200 Pro (Tecan) microplate reader. All samples and standards were measured in triplicate.

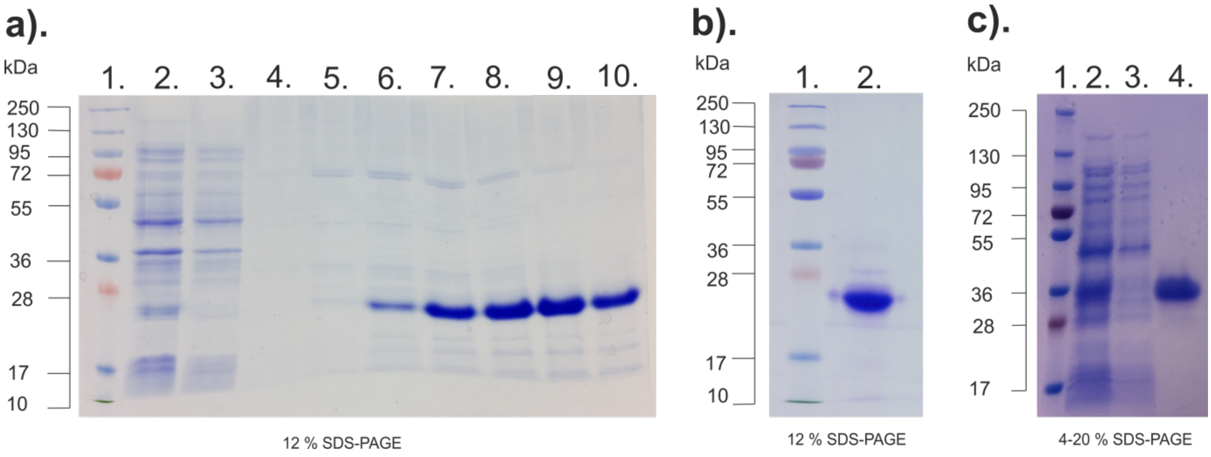

Supplementary Figure 26. SDS-PAGE analysis of purified ^{15}N labelled and unlabelled G1S HASPA. a). SDS-PAGE analysis of ^{15}N labelled G1S HASPA after nickel affinity chromatography. Lanes: 1. Ladder, 2. Total lysate, 3. Unbound lysate, 4 – 10. Nickel column elution fractions. b) SDS-PAGE analysis of purified ^{15}N labelled G1S HASPA after gel filtration chromatography. Lanes: 1. Ladder, 2. purified ^{15}N labelled G1S HASPA. c) SDS-PAGE analysis of unlabelled G1S HASPA after nickel affinity and gel filtration chromatography. Lanes: 1. Ladder, 2. Total lysate, 3. Unbound lysate, 4. Purified unlabelled G1S HASPA

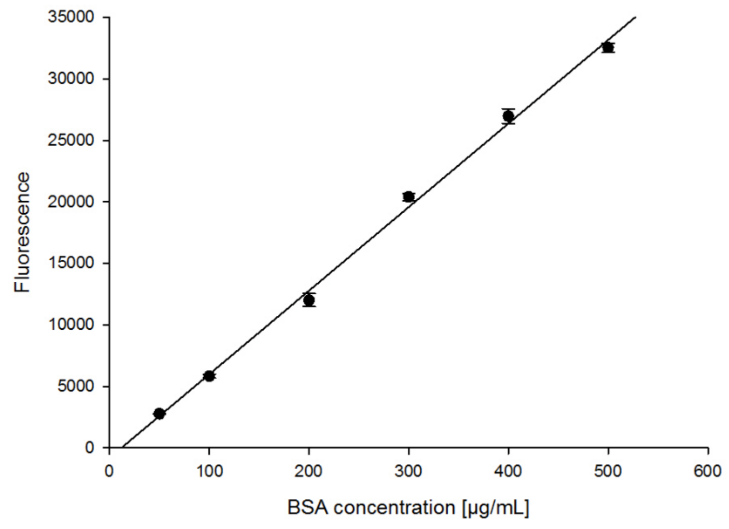

Supplementary Figure 27. A calibration curve produced with OPA reagent using BSA standards of known concentration. The standard curve was used to determine the concentration of ^{15}N labelled and unlabelled G1S HASPA based on the fluorescence readings taken after the addition of OPA reagent.

6. Peptide and protein chemical modifications

Oxidation of SLYRAG S43 to glyoxyl-LYRAG 8

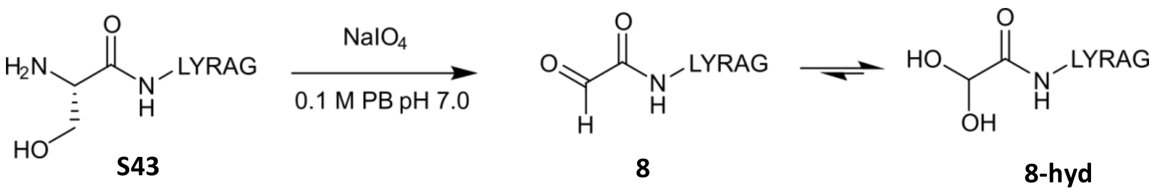

Oxidation of SLYRAG **S43** to glyoxyl-LYRAG **8** was carried out by dissolving a desired amount of peptide in 1 mL of 25 mM phosphate buffer (PB) pH 7.0, followed by addition of 2 equivs. of $NaIO_4$. The solution was vortexed, then allowed to sit at room temperature in the dark for 1 h. The solution was then loaded onto a solid phase extraction cartridge (Grace Davison Extract Clean, 8 mL reservoir, Fisher Scientific) equilibrated with water/acetonitrile. After initial washing with water, the product was eluted over a gradient of acetonitrile. Fractions containing pure, oxidised peptide (as judged by LC-MS analysis) were pooled and subsequently lyophilised to give glyoxyl-LYRAG **8** as an orange solid, which was stored at -20 °C until required.

Validation of OPAL on glyoxyl-LYRAG 8

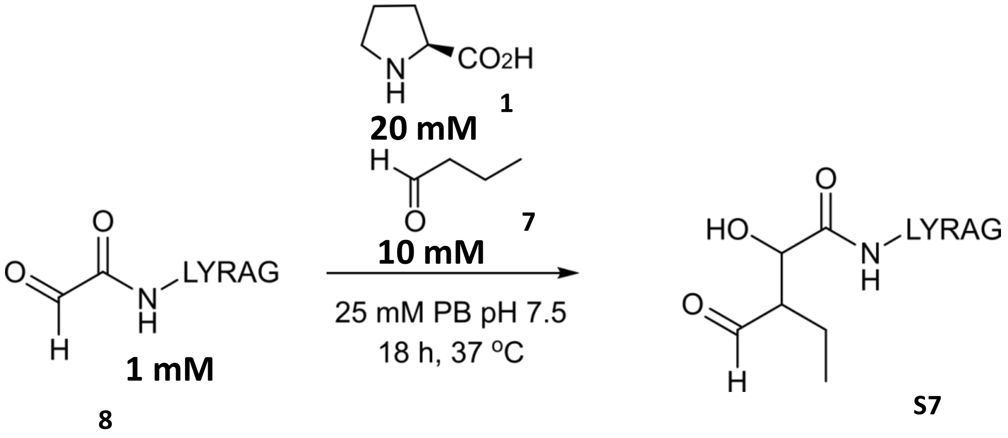

A 200 µL aliquot of a 5 mM glyoxyl-LYRAG **8** stock in 25 mM PB pH 7.5 was charged with 690 µL of 25 mM PB pH 7.5, and then charged with 100 µL of a 200 mM L-proline **1** stock solution in 25 mM PB pH 7.5. The solution was then charged with 10 µL of a 1M butyraldehyde **7** stock solution in 25 mM PB pH 7.5. The reaction was vortexed, and allowed to sit at 37 °C overnight without further agitation. The resulting OPAL product **S7** was then characterised by LC-MS.

Transamination of horse heart myoglobin S1 to glyoxyl myoglobin 5

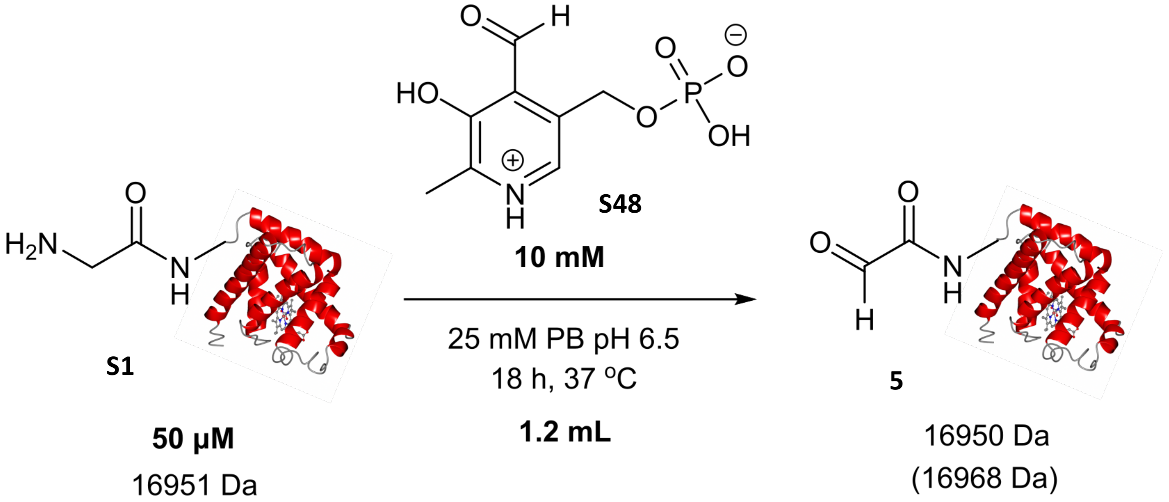

The following procedure was based on previously published literature[12]. A 480 µL aliquot of a 250 µM of myoglobin **S1** stock solution in 25 mM PB pH 6.5 was charged with 1.2 mL of a 25 mM pyridoxal-5-phosphate **S48** solution in 25 mM PB pH 6.5 (pH adjusted to pH 6.5 using 2M NaOH), and then charged with 720 µL of 25 mM PB pH 6.5. Final pH of solution was checked either by pH probe or pH paper. The mixture was briefly agitated, and incubated at 37 °C without further agitation for 24 h. The solution was then purified via spin concentration using 10,000 MWCO, and the resulting glyoxyl-myoglobin solution was concentrated to 200 µM, eluting with water. Oxidation to glyoxyl-myoglobin **5** was confirmed by LC-MS.

Validation of OPAL on glyoxyl-myoglobin 5

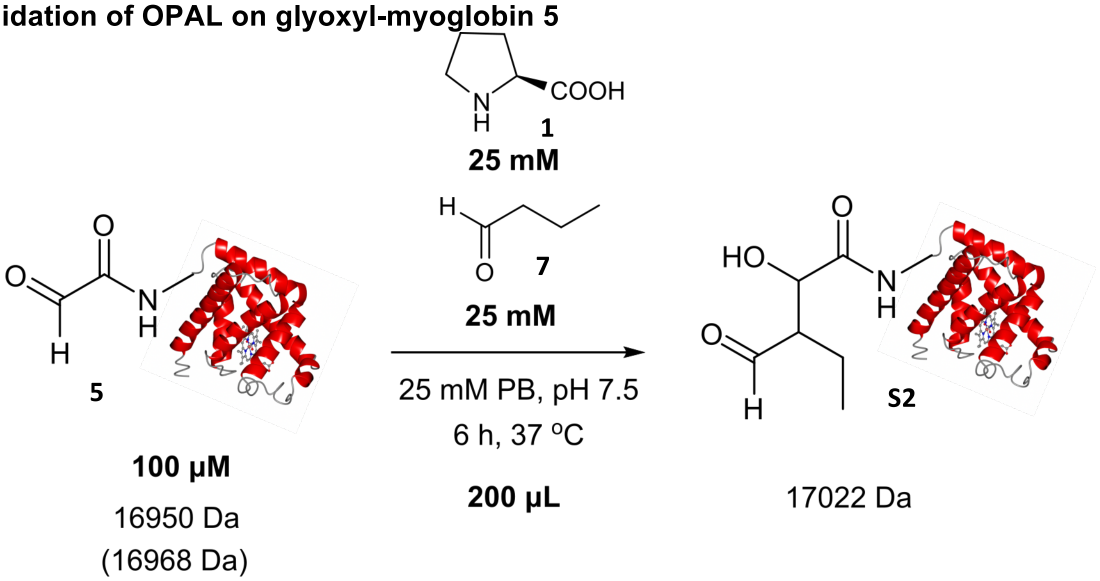

A 100 µL aliquot of 200 µM glyoxyl myoglobin **5** in MQ H_2O was charged with 50 µL of 50 mM PB pH 7.5, and then charged with 25 µL of a 200 mM L-proline **1** stock solution in 50 mM PB pH 7.5. The solution was then charged with 25 µL of a 200 mM butyraldehyde **7** stock solution in 50 mM PB pH 7.5. Following mixing by pipetting, the reaction was allowed to sit at 37 °C for 6 h without further agitation. The resulting OPAL product **S2** was then characterised by LC-MS.

Oxidation of thioredoxin S24 to glyoxyl-thioredoxin 6

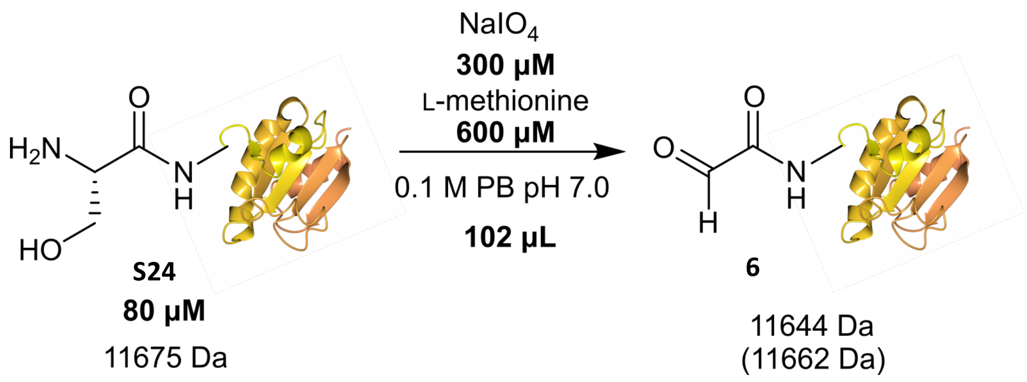

A 100 µL aliquot of an 85 µM thioredoxin **S24** stock in 25 mM PB pH 7.5 was charged with 1 µL of a 66 mM L-methionine stock solution in 0.1 M PB, 0.1 M NaCl, pH 7.0, and 1 µL of a 33 mM $NaIO_4$ stock solution in 0.1 M PB, 0.1 M NaCl, pH 7.0. The solution was mixed by gentle pipetting, and allowed to sit on ice in the dark for 4 min. The reaction was immediately purified using a PD SpinTrap G25 desalting column (GE Healthcare Life Sciences), eluting into 25 mM PB pH 7.5. Quantitative oxidation to glyoxyl-thioredoxin **6** was confirmed by LC-MS analysis.

Validation of OPAL on glyoxyl-thioredoxin 6

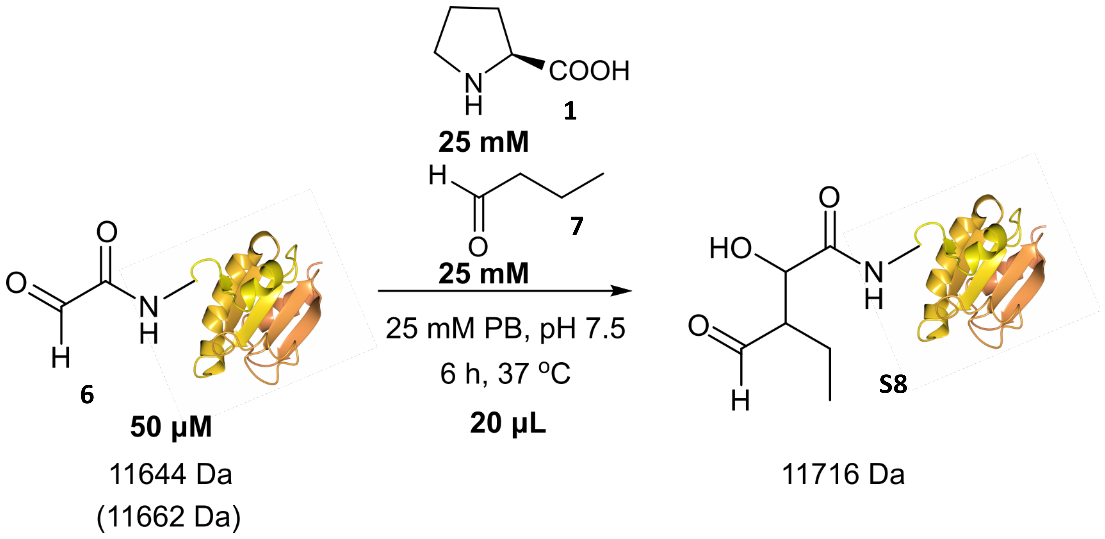

A 12 µL aliquot of a 80 µM glyoxyl-thioredoxin **6** stock (prepared as described earlier) in 25 mM PB pH 7.5 charged with 4 µL of 25 mM PB pH 7.5, and then charged with 2.5 µL of a 200 mM L-proline **1** stock solution in 25 mM PB pH 7.5. The solution was then charged with 1.5 µL of a 200 mM butyraldehyde **7** stock solution in 25 mM PB pH 7.5. Following mixing by pipetting, the reaction was allowed to sit at 37 °C for 6 h without further agitation. Quantitative conversion to the desired OPAL product **S8** was confirmed by LC-MS analysis.

Oxidation of GFP S49 (Y39CycloOctK) to glyoxyl-GFP S50

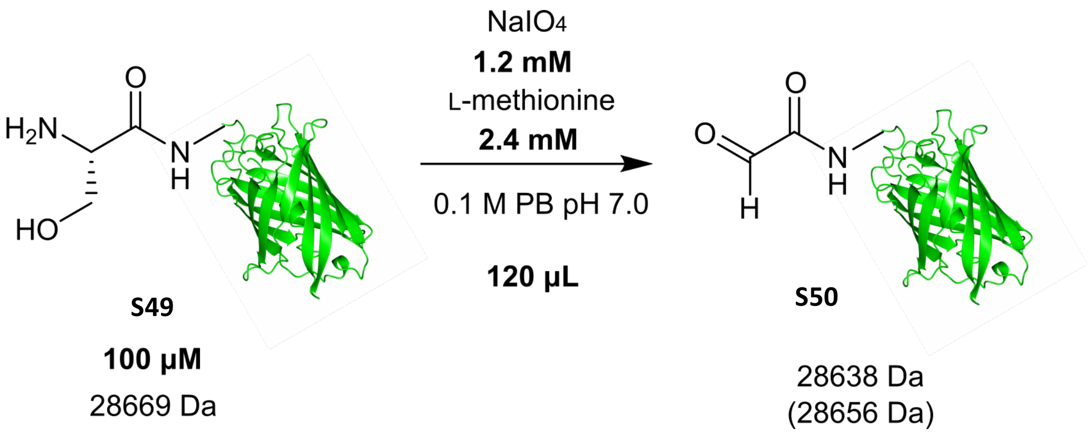

A 100 µL aliquot of a 100 µM GFP **S49** (Y39CycloOctK) stock in 1 x PBS, pH 7.4, was charged with 3 µL of a 66 mM L-methionine stock solution in 0.1 M PB, 0.1 M NaCl, pH 7.0, and 2 µL of a 33 mM $NaIO_4$ stock solution in 0.1 M PB, 0.1 M NaCl, pH 7.0. The solution was mixed by gentle pipetting, and allowed to sit on ice in the dark for 4 min. The reaction was immediately purified using a PD SpinTrap G25 desalting column (GE Healthcare Life Sciences), eluting into 25 mM PB pH 7.5. Quantitative oxidation to glyoxyl-GFP **S50** (Y39CycloOctK) was confirmed by LC-MS analysis.

Palladium decaging of sfGFP(N150ThzK) S11[4]

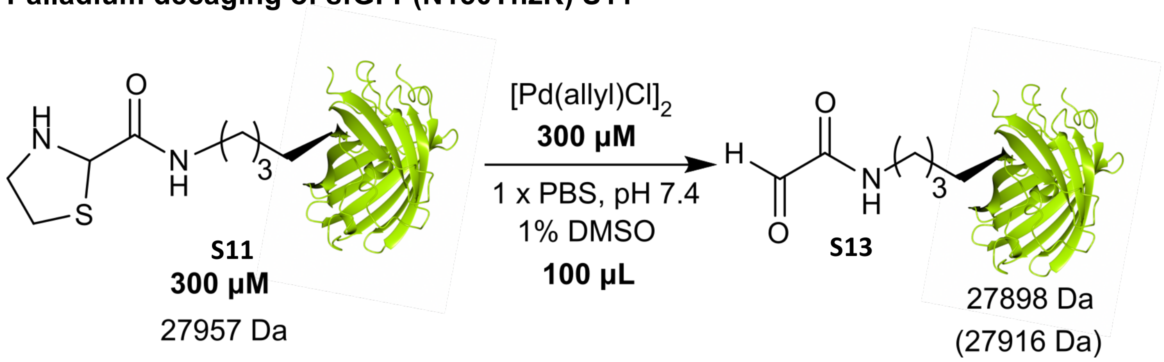

A 99 µl aliquot of a 300 µM sfGFP(N150ThzK) **S11** stock in 1 x PBS, pH 7.4, was charged with 1 µl of a 30 mM allylpalladium(II) chloride dimer. The solution was mixed by gentle pipetting, and allowed to sit at room temperature for 60 min without further agitation. The reaction was then quenched by addition of 10 µL of a 3-mercaptopropanoic acid solution, 1% v/v solution, 10 x PBS (final conc^{n} = 0.1% v/v) to each aliquot, and allowed to sit at 25 ^{o}C for 15 min without further agitation. The reaction was then desalted using a PD MiniTrap G-25 (GE Healthcare Life Sciences), eluting with 25 mM PB pH 7.5. Conversion to the decaged protein aldehyde **S13** was confirmed by ESI-MS analysis.

Palladium decaging of GFP(Y39ThzK) S12 [4]

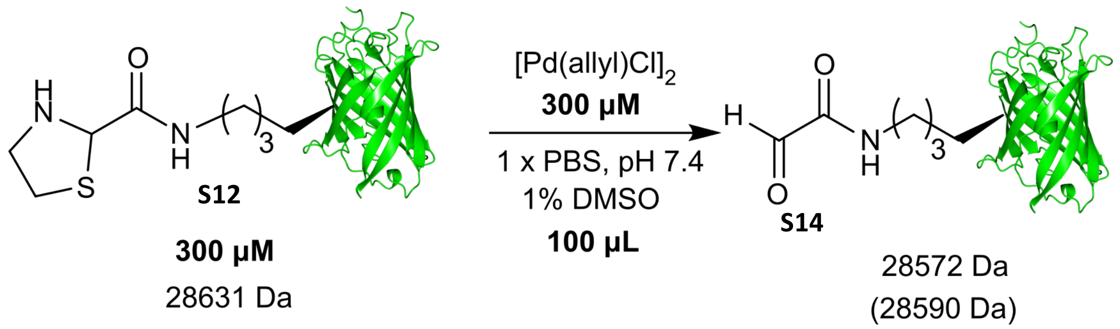

A 99 μl aliquot of a 300 μM GFP(Y39ThzK) **S12** stock in 1 x PBS, pH 7.4, was charged with 1 μL of a 30 mM allylpalladium(II) chloride dimer. The solution was mixed by gentle pipetting, and allowed to sit at room temperature for 60 min without further agitation. The reaction was then quenched by addition of 10 μL of a 3-mercaptopropanoic acid solution, 1% v/v solution, 10 x PBS (final conc^{n} = 0.1% v/v) to each aliquot, and allowed to sit at 25 ^{o}C for 15 min without further agitation. The reaction was then desalted using a PD MiniTrap G-25 (GE Healthcare Life Sciences), eluting with 25 mM PB pH 7.5. Conversion to the decaged protein aldehyde **S14** was confirmed by ESI-MS analysis.

Oxidation of HASPA(G1S) 31 to glyoxyl-HASPA(G1S) S26

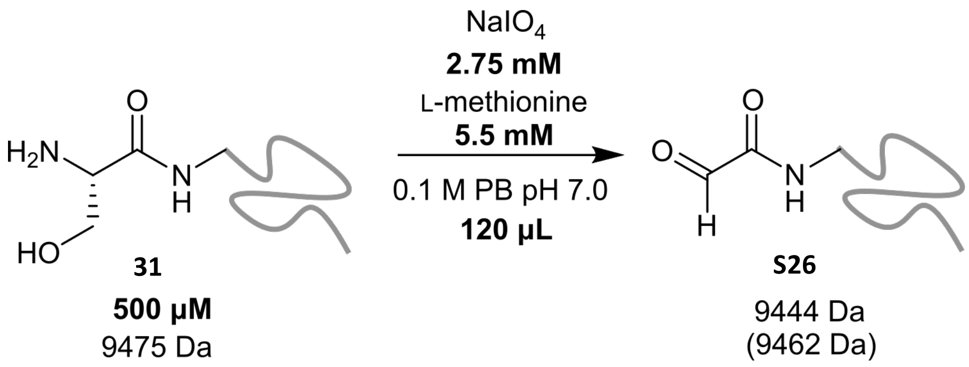

A 100 μL aliquot of an 600 μM HASPA(G1S) **31** stock in 0.1 M PB, 0.1 M NaCl, pH 7.0 was charged with 10 μL of a 66 mM L-methionine stock solution in 0.1 M PB, 0.1 M NaCl, pH 7.0, and 10 μL of a 33 mM $NaIO_4$ stock solution in 0.1 M PB, 0.1 M NaCl, pH 7.0. The solution was mixed by gentle pipetting, and allowed to sit on ice in the dark for 4 min. The reaction was immediately purified using a PD SpinTrap G25 desalting column (GE Healthcare Life Sciences), eluting into 25 mM PB pH 7.5. Quantitative oxidation to glyoxyl-HASPA **S26** was confirmed by LC-MS analysis.

Oxidation of [15N]HASPA(G1S) 31-15N to glyoxyl-[15N]HASPA(G1S) S26-15N

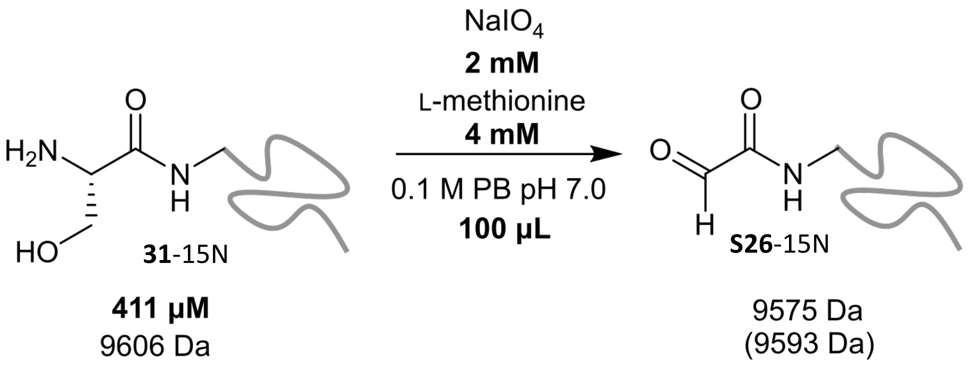

A 50 µL aliquot of an 822 µM [15N]HASPA(G1S) stock in 0.1 M PB, 0.1 M NaCl, pH 7.0 was charged with 36 µL of 0.1 M PB, 0.1 M NaCl, pH 7.0 buffer, 7 µL of a 66 mM L-methionine stock solution in 0.1 M PB, 0.1 M NaCl, pH 7.0, and 7 µL of a 33 mM $NaIO_4$ stock solution in 0.1 M PB, 0.1 M NaCl, pH 7.0. The solution was mixed by gentle pipetting, and allowed to sit on ice in the dark for 4 min. The reaction was immediately purified using a PD SpinTrap G25 desalting column (GE Healthcare Life Sciences), eluting into 25 mM PB pH 7.5. Quantitative oxidation to glyoxyl-HASPA was confirmed by LC-MS analysis.

Synthesis of fluorescently labelled thioredoxin 23

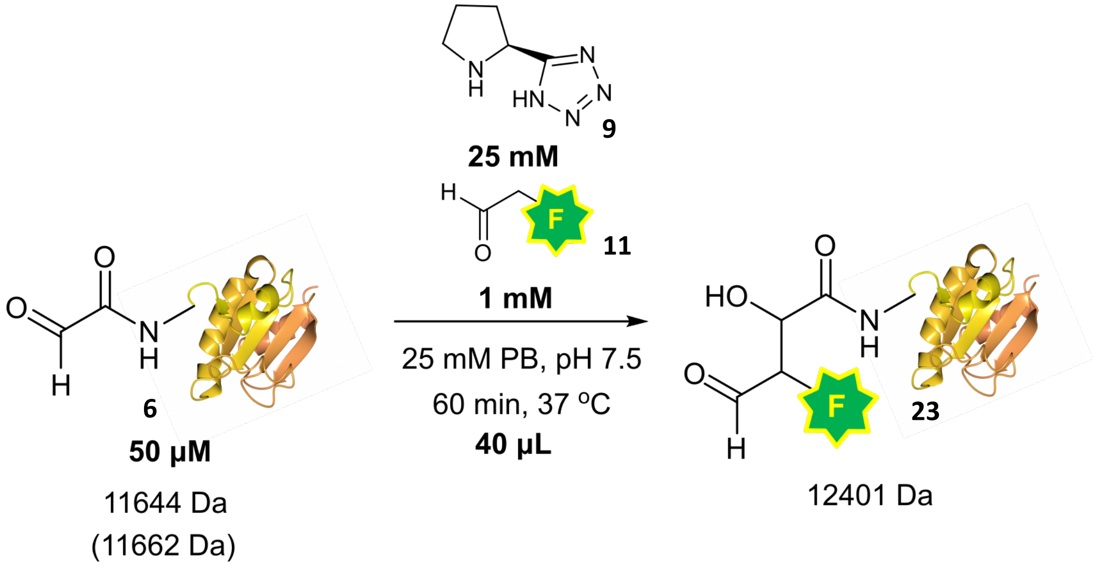

A 25 µL aliquot of 80 µM glyoxyl-thioredoxin **6** stock prepared as described earlier in 25 mM PB pH 7.5 was charged with 5 µL of a 200 mM proline tetrazole **9** stock solution in 25 mM PB pH 7.5. The solution was then charged with 10 µL of a 4 mM fluorescent label **11** stock solution in 25 mM PB pH 7.5. Following mixing by pipetting, the reaction was allowed to sit at 37 °C for 60 min without further agitation. Quantitative labelling to fluorescently labelled thioredoxin **23** was confirmed by LC-MS analysis.

Synthesis of biotinylated thioredoxin S51

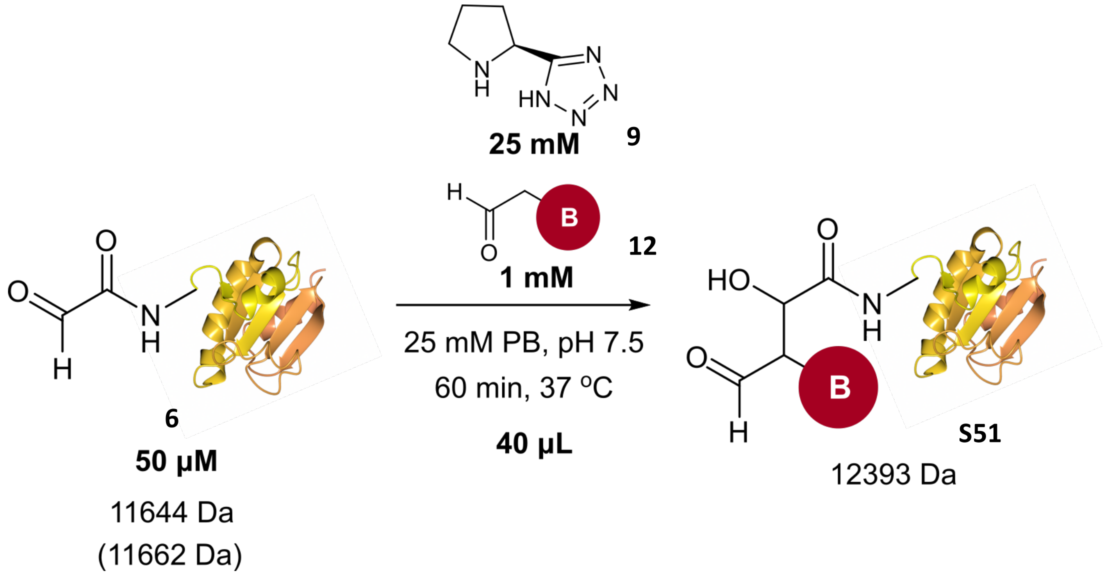

A 25 µL aliquot of 80 µM glyoxyl-thioredoxin **6** stock (prepared as described earlier) in 25 mM PB pH 7.5 was charged with 5 µL of a 200 mM proline tetrazole **9** stock solution in 25 mM PB pH 7.5. The solution was then charged with 10 µL of a 4 mM biotin affinity tag **12** stock solution in 25 mM PB pH 7.5. Following mixing by pipetting, the reaction was allowed to sit at 37 °C for 60 min without further agitation. Quantitative labelling to biotinylated thioredoxin **S51** was confirmed by LC-MS analysis.

Synthesis of azide labelled thioredoxin S15

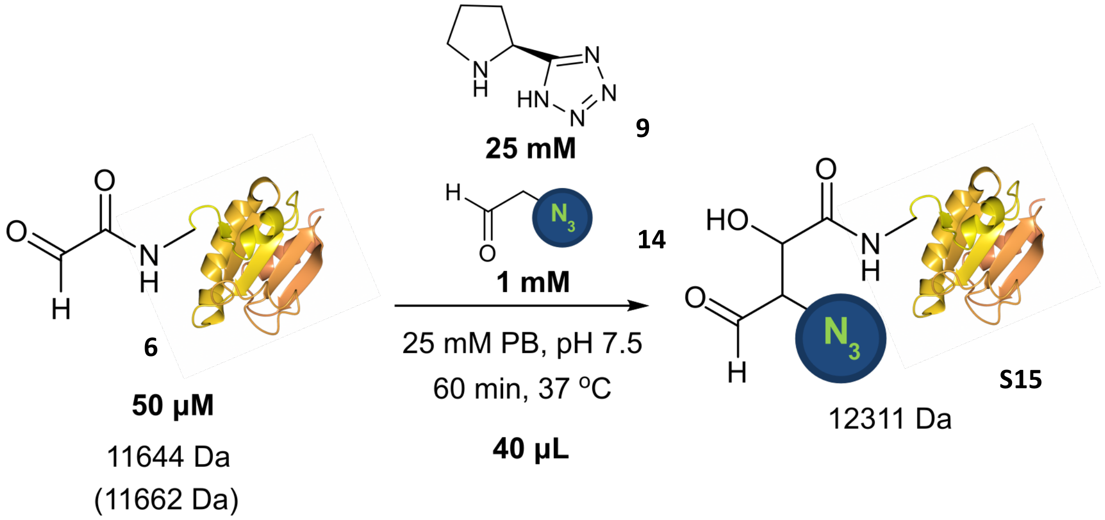

A 25 µL aliquot of 80 µM glyoxyl-thioredoxin **6** stock (prepared as described earlier) in 25 mM PB pH 7.5 was charged with 5 µL of a 200 mM proline tetrazole **9** stock solution in 25 mM PB pH 7.5. The solution was then charged with 10 µL of a 4 mM bioorthogonal azide handle **14** stock solution in 25 mM PB pH 7.5. Following mixing by pipetting, the reaction was allowed to sit at 37 °C for 60 min without further agitation. Quantitative labelling to azide labelled thioredoxin **S15** was confirmed by LC-MS analysis.

Synthesis of fluorescently labelled myoglobin 25

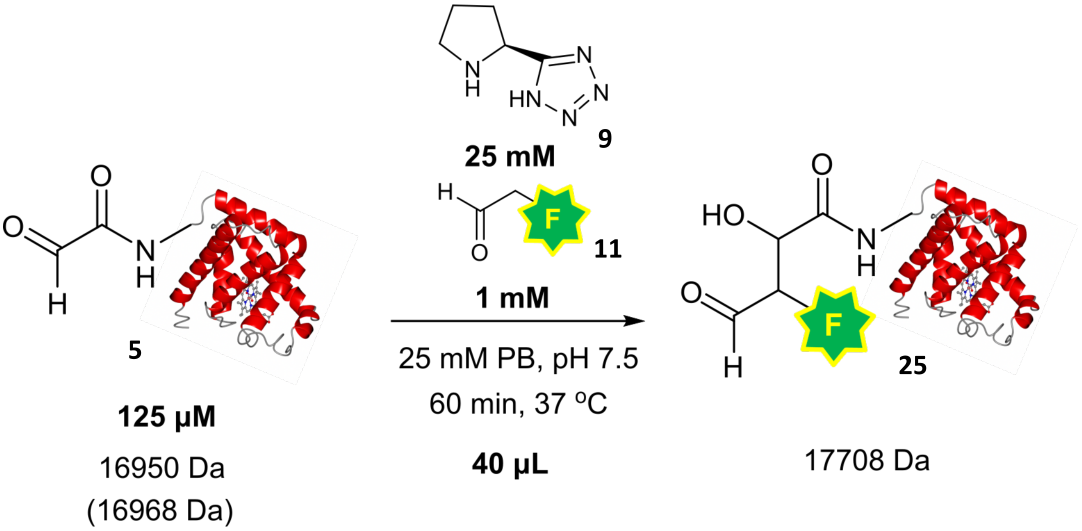

A 25 µL aliquot of 200 µM glyoxyl-myoglobin **5** stock (prepared as described earlier) in 25 mM PB pH 7.5 was charged with 5 µL of a 200 mM proline tetrazole **9** stock solution in 25 mM PB pH 7.5. The solution was then charged with 10 µL of a 4 mM fluorescent label **11** stock solution in 25 mM PB pH 7.5. Following mixing by pipetting, the reaction was allowed to sit at 37 °C for 60 min without further agitation. Quantitative labelling to fluorescently labelled myoglobin **25** was confirmed by LC-MS analysis.

Synthesis of folate labelled GFP S52 (Y39CycloOctK)

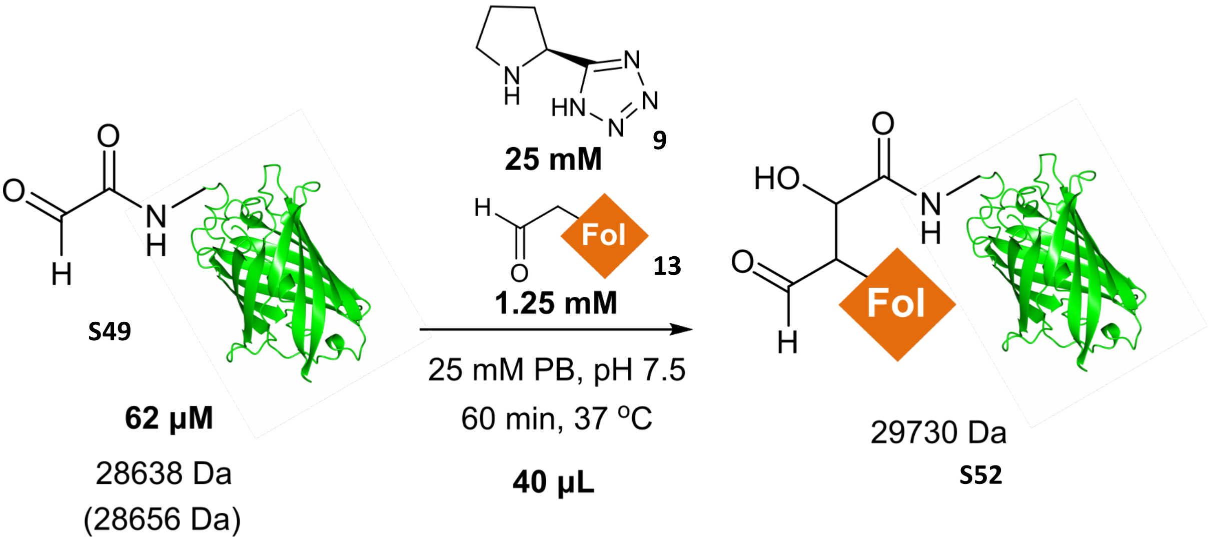

A 25 µL aliquot of 100 µM glyoxyl-GFP **S49** (Y39CycloOctK) stock (prepared as described earlier) in 25 mM PB pH 7.5 was charged with 5 µL of a 200 mM proline tetrazole **9** stock solution in 25 mM PB pH 7.5. The solution was then charged with 10 µL of a 5 mM folate targeting moiety **13** stock solution in 25 mM PB pH 7.5. Following mixing by pipetting, the reaction was allowed to sit at 37 °C for 60 min without further agitation. Quantitative labelling to folate labelled GFP **S52** (Y39CycloOctK) confirmed by ESI-MS analysis.

Synthesis of biotinylated GFP S53 (Y39CycloOctK)

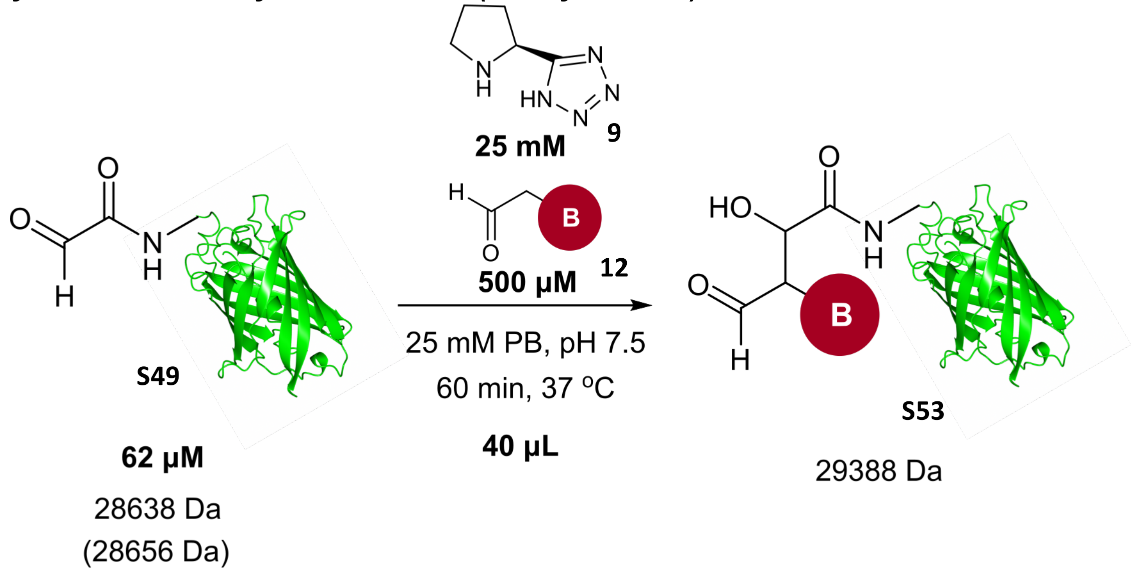

A 25 μL aliquot of 100 μM glyoxyl-GFP (Y39CycloOctK) **6** stock (prepared as described earlier) in 25 mM PB pH 7.5 was charged with 5 μL of a 200 mM proline tetrazole **9** stock solution in 25 mM PB pH 7.5. The solution was then charged with 10 μL of a 2 mM biotin affinity tag **12** stock solution in 25 mM PB pH 7.5. Following mixing by pipetting, the reaction was allowed to sit at 37 °C for 60 min without further agitation. Quantitative labelling to biotinylated GFP (Y39CycloOctK) **S53** was confirmed by ESI-MS analysis.

Synthesis of internally azide labelled sfGFP S54

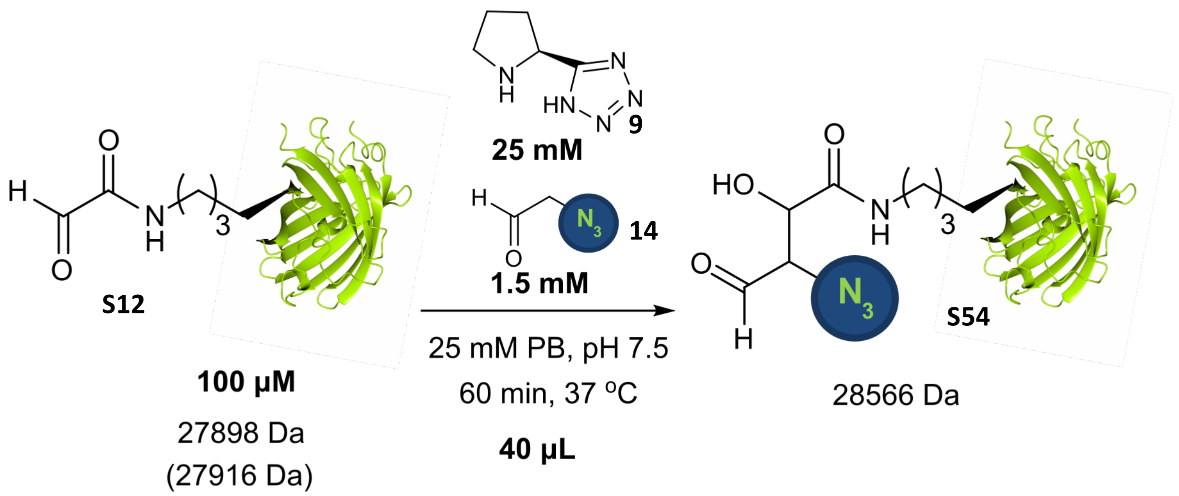

A 25 μL aliquot of 160 μM sfGFP(ThzK150Oxo) **S12** (prepared as described earlier) in 25 mM PB pH 7.5 was charged with 5 μL of a 200 mM proline tetrazole **9** stock solution in 25 mM PB pH 7.5. The solution was then charged with 10 μL of a 5 mM bioorthogonal azide handle **14** stock solution in 25 mM PB pH 7.5. Following mixing by pipetting, the reaction was allowed to sit at 37 °C for 60 min without further agitation. Quantitative labelling to internally azide labelled sfGFP **S54** was confirmed by ESI-MS analysis

Synthesis of internally azide labelled GFP S55

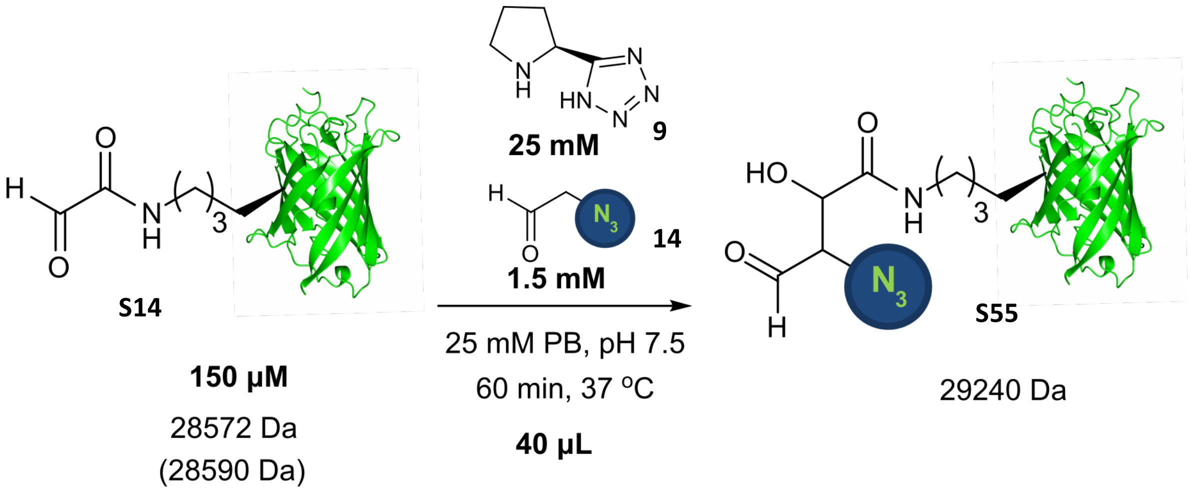

A 25 µL aliquot of 240 µM GFP(ThzK39Oxo) **S14** (prepared as described earlier) in 25 mM PB pH 7.5 was charged with 5 µL of a 200 mM proline tetrazole **9** stock solution in 25 mM PB pH 7.5. The solution was then charged with 10 µL of a 5 mM bioorthogonal azide handle **14** stock solution in 25 mM PB pH 7.5. Following mixing by pipetting, the reaction was allowed to sit at 37 °C for 60 min without further agitation. Quantitative labelling to internally azide labelled GFP **S55** was confirmed by ESI-MS analysis

Synthesis of biotinylated HASPA(G1S) S56

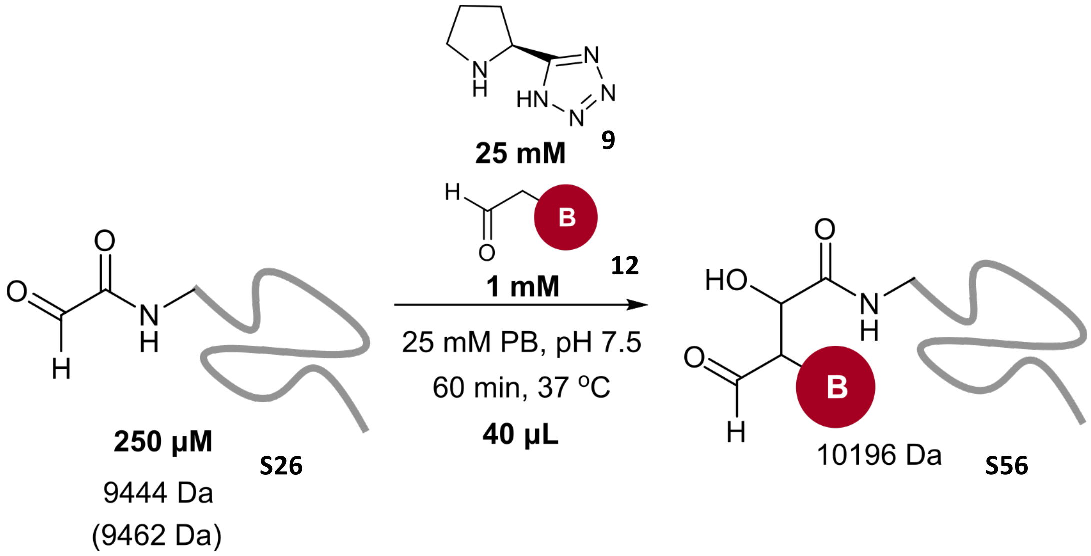

A 25 µL aliquot of 400 µM glyoxyl-HASPAG1S **S26** (prepared as described earlier) in 25 mM PB pH 7.5 was charged with 5 µL of a 200 mM proline tetrazole **9** stock solution in 25 mM PB pH 7.5. The solution was then charged with 10 µL of a 4 mM biotin affinity tag **12** stock solution in 25 mM PB pH 7.5. Following mixing by pipetting, the reaction was allowed to sit at

37 °C for 60 min without further agitation. Quantitative labelling to biotinylated HASPA **S56** was confirmed by LC-MS analysis.

Synthesis of azide labelled HASPA(G1S) S57

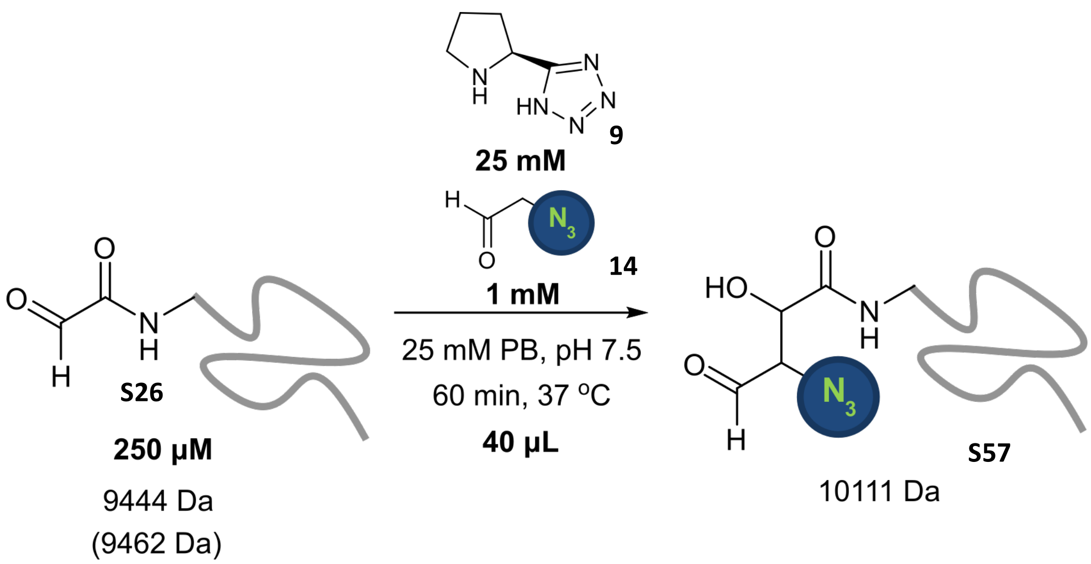

A 25 µL aliquot of 400 µM glyoxyl-HASPAG1S **S26** (prepared as described earlier) in 25 mM PB pH 7.5 was charged with 5 µL of a 200 mM proline tetrazole **9** stock solution in 25 mM PB pH 7.5. The solution was then charged with 10 µL of a 5 mM bioorthogonal azide handle **14** stock solution in 25 mM PB pH 7.5. Following mixing by pipetting, the reaction was allowed to sit at 37 °C for 60 min without further agitation. Quantitative labelling to azide labelled HASPA **S57** was confirmed by LC-MS analysis.

Chemical myristoylation of HASPA

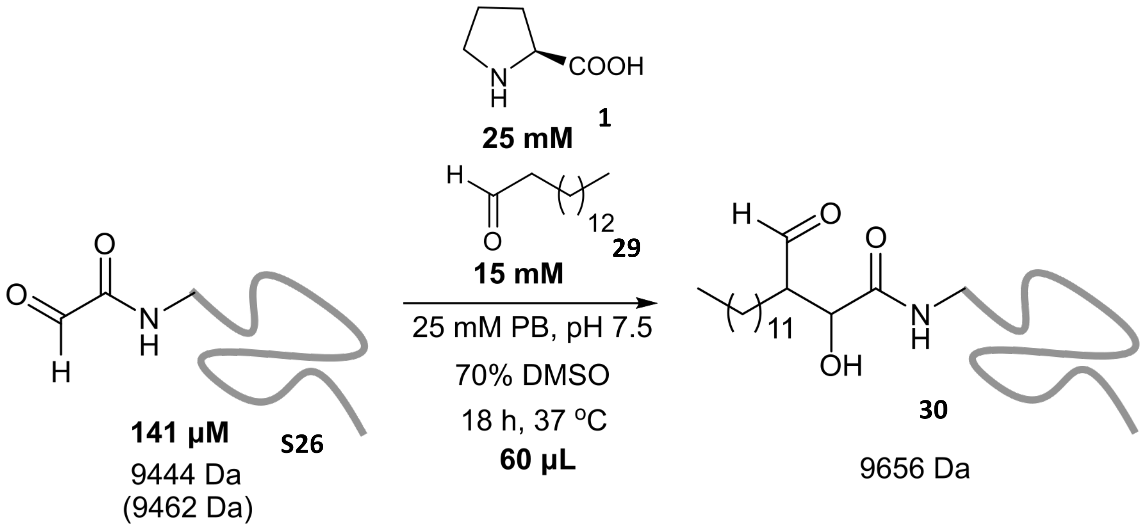

A 17 µL aliquot of 500 µM glyoxyl-HASPA **S26** stock (prepared as described earlier) in 25 mM PB pH 7.5 was charged with 1 µL of a 1.5 M L-proline **1** stock solution in 25 mM PB pH 7.5. The solution was then charged with 6 µL of DMSO, and then charged with 36 µL of a 25 mM tetradecanal **29** stock solution in DMSO. Following mixing by pipetting, the reaction was cinbated at 37 °C for 60 min without further agitation. Quantitative labelling to chemically myristoylated HASPA **30** was confirmed by LC-MS analysis (note elimination of β-hydroxyl to

afford enone of **30** is also observed). Samples were then diluted to >20% DMSO content, purified via PD MiniTrap G-25 columns (GE Healthcare Life Sciences), eluting MQ H_2O, and subsequently lyophilised to give a white powder (stored at -80 °C).

Chemical myristoylation of 15N labelled HASPA

Chemical myristoylation of glyoxyl-[15N]HASPA(G1S) **S26-15N** was identical to that of chemical myristoylation of unlabelled glyoxyl-HASPA(G1S) **S26.**

Enzymatic myristoylation of HASPA

Enzymatic modification of HASPA was performed by incubating 200 µM HASPA with 400 µM myristoyl CoA and 2 µM Leishmania major NMT in 10 mM HEPES pH 7.5, 500 mM NaCl, 0.5 mM DTT. The reaction mixture was incubated overnight at 298 K and the modification confirmed by ESI-MS. Enzymatically myristoylated HASPA was used without further purification.

Synthesis of biotinylated myoglobin S58

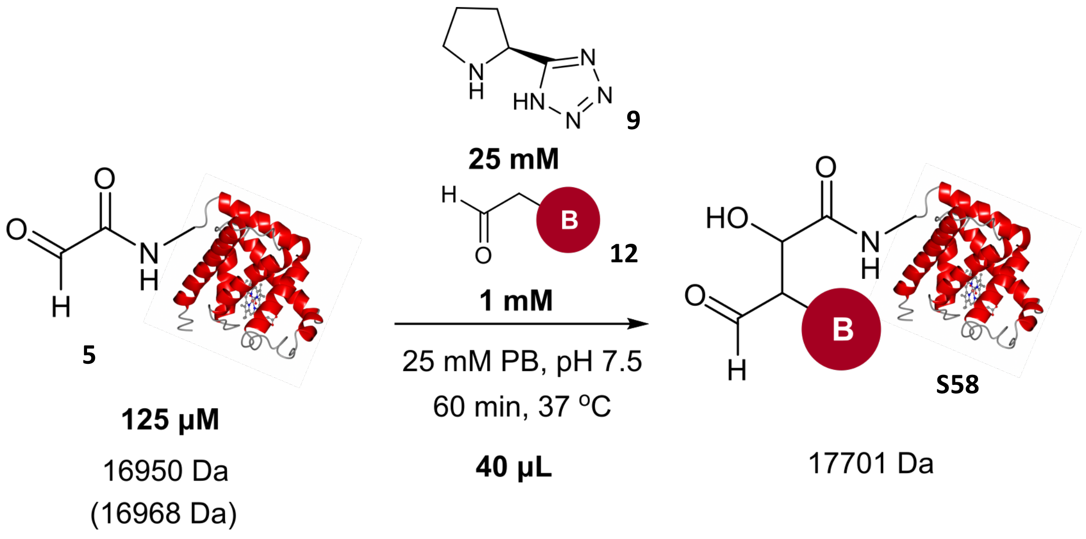

A 25 µL aliquot of 200 µM glyoxyl-myoglobin **5** stock (prepared as described earlier) in 25 mM PB pH 7.5 was charged with 5 µL of a 200 mM proline tetrazole **9** stock solution in 25 mM PB pH 7.5. The solution was then charged with 10 µL of a 4 mM biotin affinity tag **12** stock solution in 25 mM PB pH 7.5. Following mixing by pipetting, the reaction was allowed to sit at 37 °C for 60 min without further agitation. Quantitative labelling to biotinylated myoglobin **S58** was confirmed by LC-MS analysis. Structural integrity of the myoglobin protein was determined by UV/Vis analysis (see Supplementary Figure 28).

UV-Vis analysis of OPAL modified myoglobin

A control sample of myoglobin **S1** was prepared by dissolving lyophilised myoglobin **S1** in 25 mM PB pH 7.5, and a sample of glyoxyl-myoglobin **5** was prepared as described previously, and a sample of biotinylated myoglobin **S58** prepared as described previously. UV-Vis measurements were obtained for unmodified myoglobin **S1** (without desalting), glyoxyl-myoglobin **5** (in 25 mM PB pH 7.5) and biotinylated myoglobin **S58** (desalted using a PD MiniTrap G-25 column (GE Healthcare Life Sciences), eluting into 25 mM PB pH 7.5). Based on the absorbance at 410 nm that is characteristic of the myoglobin heme group, the protein structure is retained post modification.

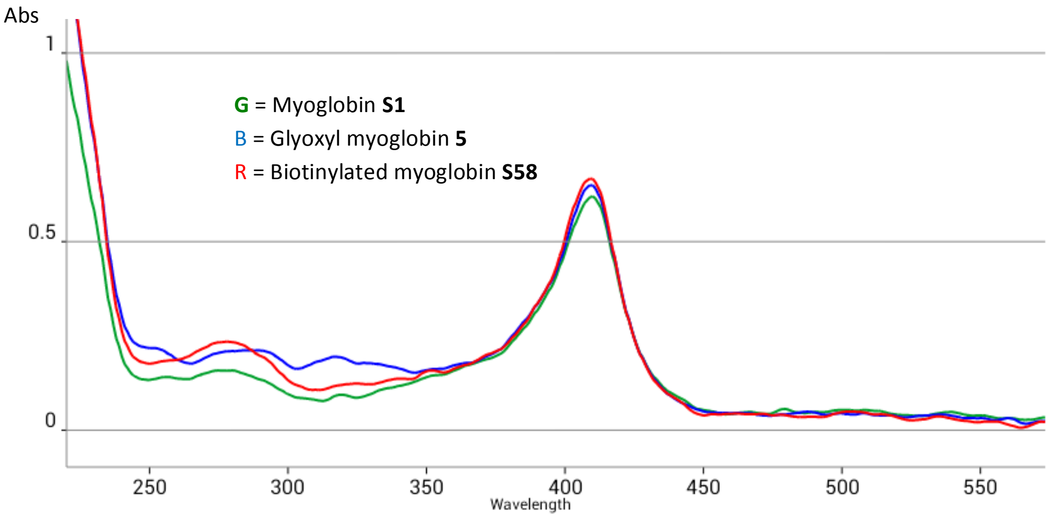

Supplementary Figure 28. UV-Vis measurements of myoglobin **S1** (green line), glyoxyl-myoglobin **5** (blue line), and biotinylated myglobin **S58** (red line).

Synthesis of azide labelled myoglobin S59

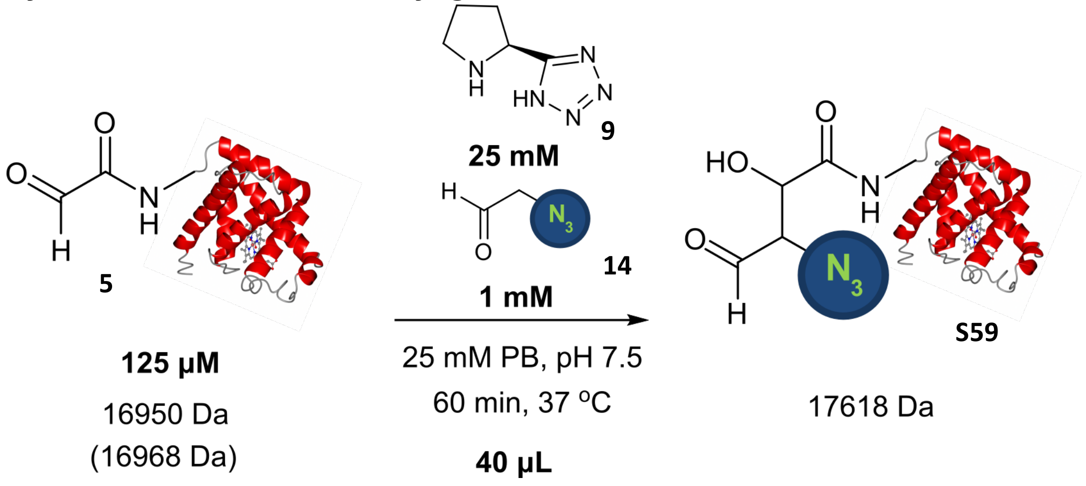

A 25 µL aliquot of 200 µM glyoxyl-myoglobin **5** stock (prepared as described earlier) in 25 mM PB pH 7.5 was charged with 5 µL of a 200 mM proline tetrazole **9** stock solution in 25 mM PB pH 7.5. The solution was then charged with 10 µL of a 4 mM bioorthogonal azide

handle **14** stock solution in 25 mM PB pH 7.5. Following mixing by pipetting, the reaction was allowed to sit at 37 °C for 60 min without further agitation. Quantitative labelling to azide labelled myoglobin **S59** was confirmed by LC-MS analysis.

Synthesis of fluorescently labelled [15N] HASPA(G1S) S60

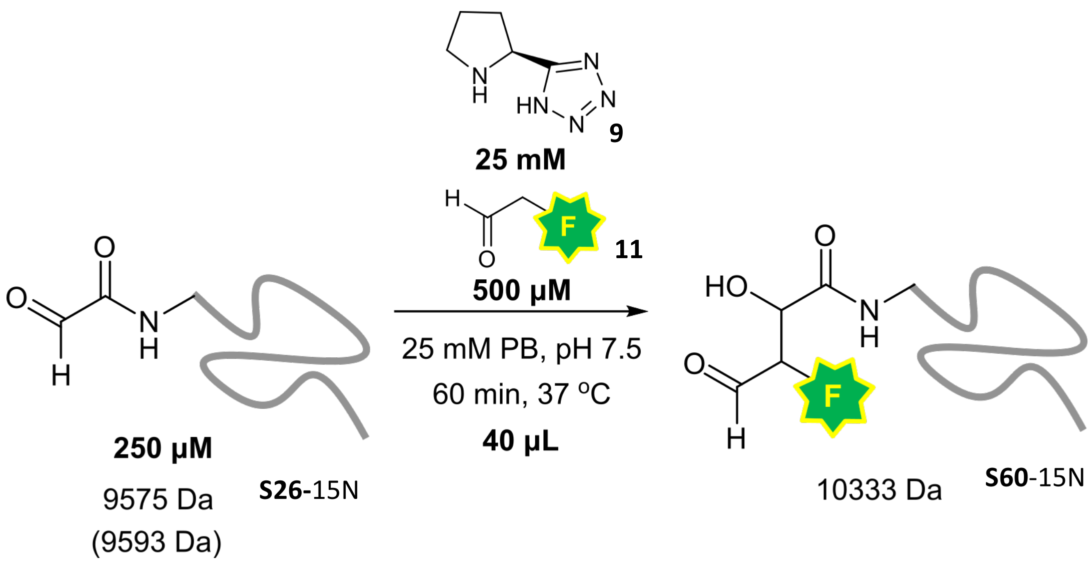

A 25 µL aliquot of 400 µM [15N]HASPA(G1S) **S26**-15N (prepared as described earlier) in 25 mM PB pH 7.5 was charged with 5 µL of a 200 mM proline tetrazole **9** stock solution in 25 mM PB pH 7.5. The solution was then charged with 10 µL of a 2 mM fluorescent label **11** stock solution in 25 mM PB pH 7.5. Following mixing by pipetting, the reaction was allowed to sit at 37 °C for 60 min without further agitation. Quantitative labelling to fluorescently labelled [15N]HASPA **S60**-15N was confirmed by LC-MS analysis.

Synthesis of internally biotinylated GFP S61

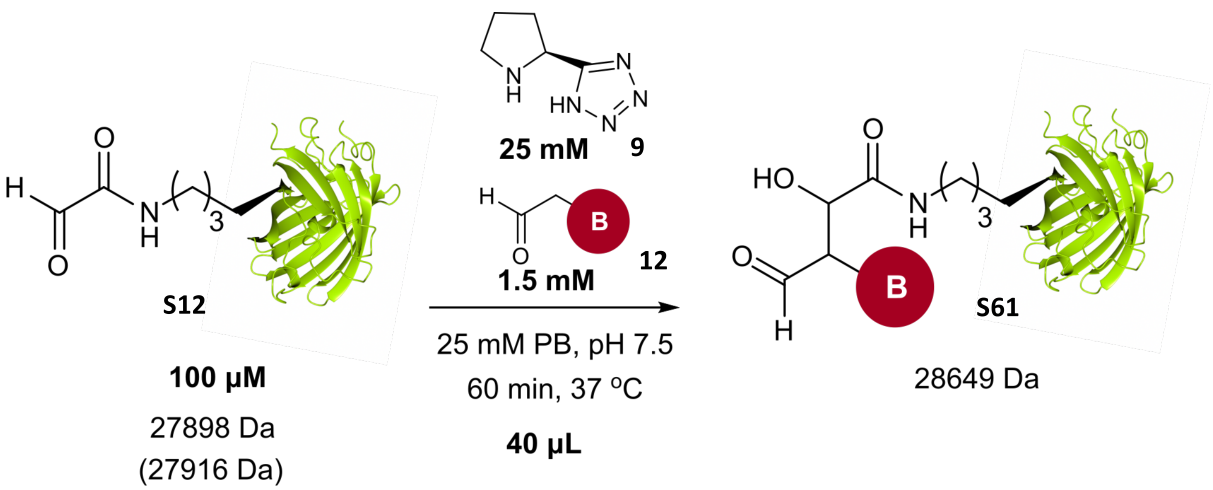

A 25 µL aliquot of 160 µM sfGFP(ThzK150Oxo) **S12** (prepared as described earlier) in 25 mM PB pH 7.5 was charged with 5 µL of a 200 mM proline tetrazole **9** stock solution in 25 mM PB pH 7.5. The solution was then charged with 10 µL of a 5 mM biotin affinity tag **12**

stock solution in 25 mM PB pH 7.5. Following mixing by pipetting, the reaction was allowed to sit at 37 °C for 60 min without further agitation. Quantitative labelling to internally biotinylated sfGFP **S61** was confirmed by ESI-MS analysis

Synthesis of aldol-oxime-LYRAG S19

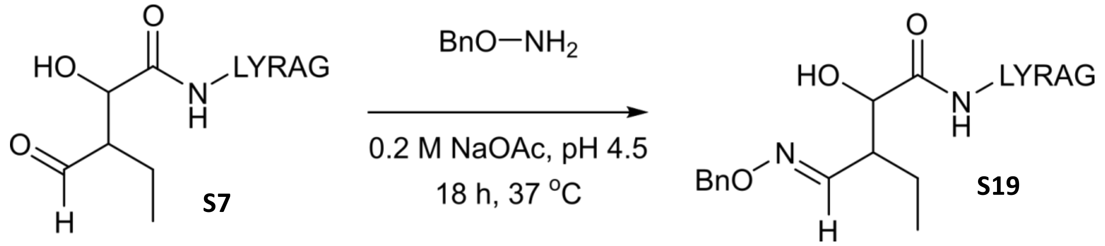

A 20 µL aliquot of a 5 mM α-ethyl-β-hydroxy aldehyde-LYRAG **S7** stock in MQ H_2O was charged with 879 µL of 0.1 M NaOAc, pH 4.5. The solution was then charged with 1 µL of *O*-benzylhydroxylamine. The reaction was vortexed, and incubated 37 °C overnight without further agitation. Successful conversion to dually modified peptide **S19** was confirmed by LC-MS analysis.

Synthesis of aldol-*iso*-Pictet-Spengler-LYRAG S17

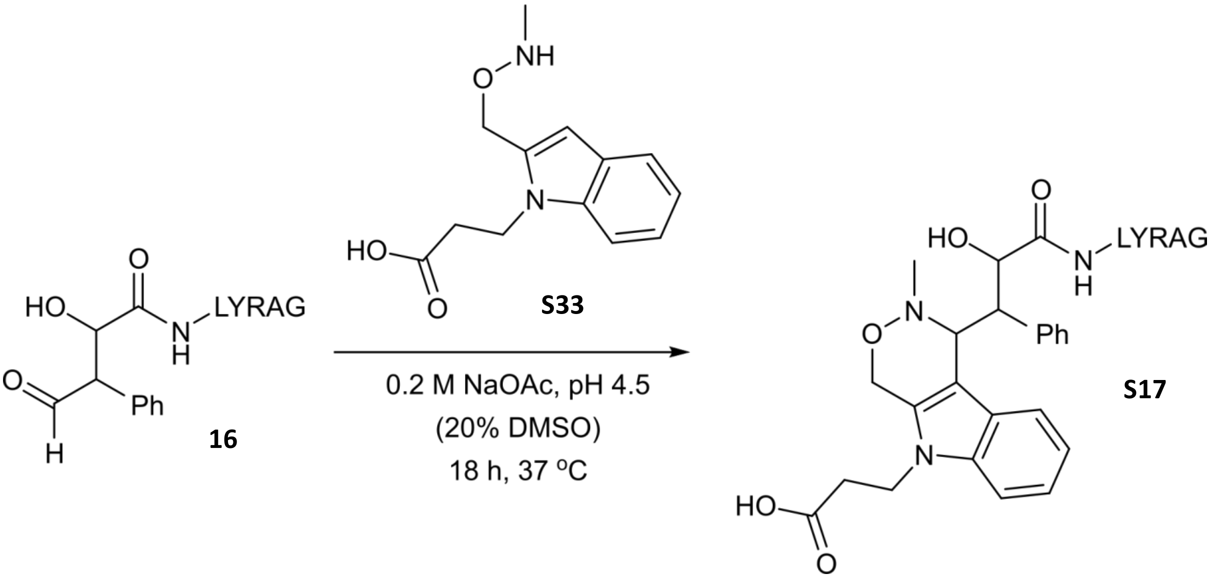

A 20 µL aliquot of a α-phenyl-β-hydroxy aldehyde-LYRAG **16** stock in MQ H_2O was charged with 780 µL of 0.1 M NaOAc. The solution was then charged with 200 µL of a 50 mM indole **S33** stock solution in DMSO. The reaction was vortexed, and incubated 37 °C overnight without further agitation. Successful conversion to dually modified peptide **S17** was confirmed by LC-MS analysis.

Synthesis of aldol-ABAO-LYRAG S18

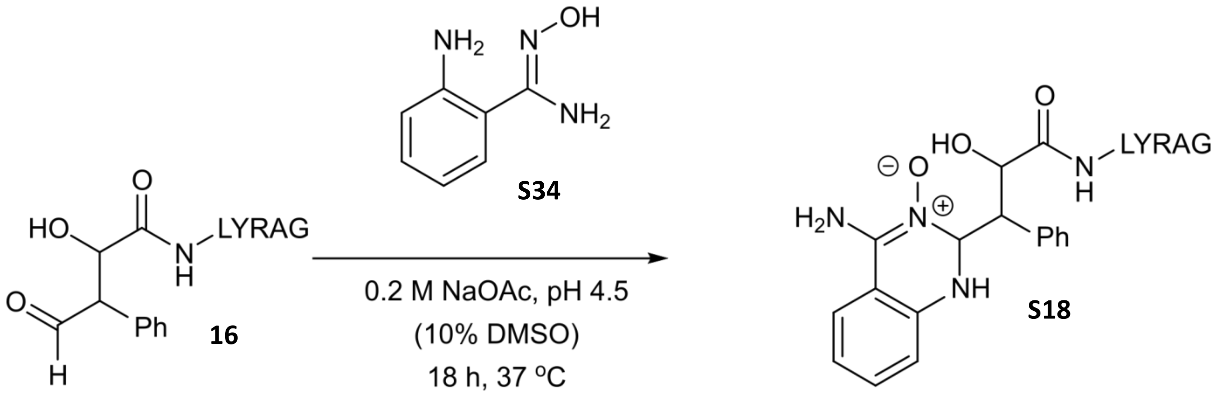

A 20 μL aliquot of a 5 mM α-phenyl-β-hydroxy aldehyde-LYRAG **16** stock in MQ H_2O was charged with 780 μL of 0.1 M NaOAc, pH 4.5. The solution was then charged with 100 μL of ABAO **S34** stock solution in DMSO. The reaction was vortexed, and incubated 37 °C overnight without further agitation. Successful conversion to dually modified peptide **S18** was confirmed by LC-MS analysis.

Screening of aniline catalysts for oxime ligation

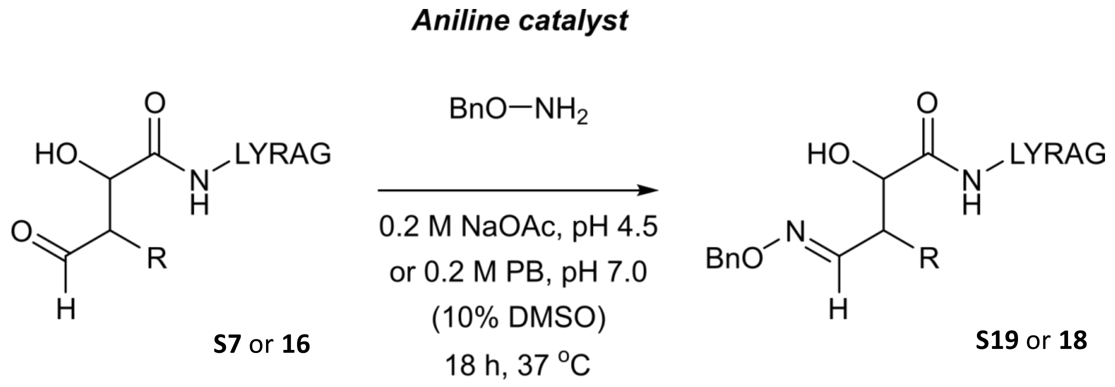

A 10 μL aliquot of a 5 mM **S7** or **16** stock in MQ H_2O was charged with 879 μL of 0.2 M NaOAc, pH 4.5, or with 879 μL of 0.2 M PB pH 7.5. The solution was then charged with 1 μL of *O*-benzylhydroxylamine, and then charged with 100 μL of 1M aniline catalyst in DMSO. The reaction was vortexed, and incubated 37 °C overnight without further agitation. . Successful conversion to dually modified peptide **S19** or **18** was confirmed by LC-MS analysis.

Synthesis of fluorescently labelled, biotinylated thioredoxin 22

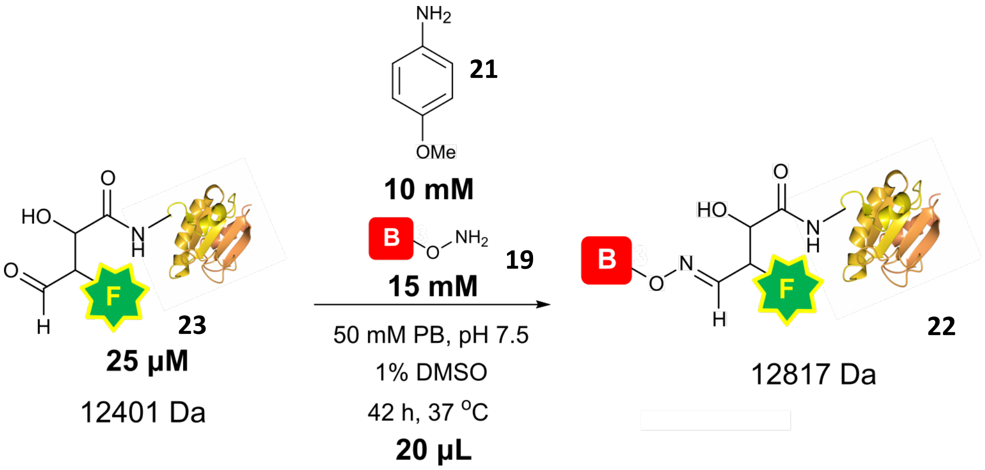

A 120 µL aliquot of fluorescently labelled thioredoxin **23** (prepared as described previously) was desalted using a PD SpinTrap G-25 column (GE Healthcare Life Sciences), eluting with 5 mM PB pH 7.5. A 10 µL aliquot of desalted protein was then charged with 3.8 µL of 0.2 M PB pH 7.5, and 4.8 µL of MQ H_2O. The solution was then charged with 1.2 µL of a 250 mM aminooxy biotin **19** stock solution in 50 mM PB pH 7.5 (pH adjusted to pH 7.5 using 2M NaOH), and then charged with 0.2 µL of a 1M *p*-anisidine **21** stock solution in DMSO. Following mixing by pipetting, the reaction was allowed to sit at 37 °C for 42 h without further agitation. Successful conversion (~70%) to dually modified protein **22** was confirmed by LC-MS.

Synthesis of azide labelled, biotinylated thioredoxin 24

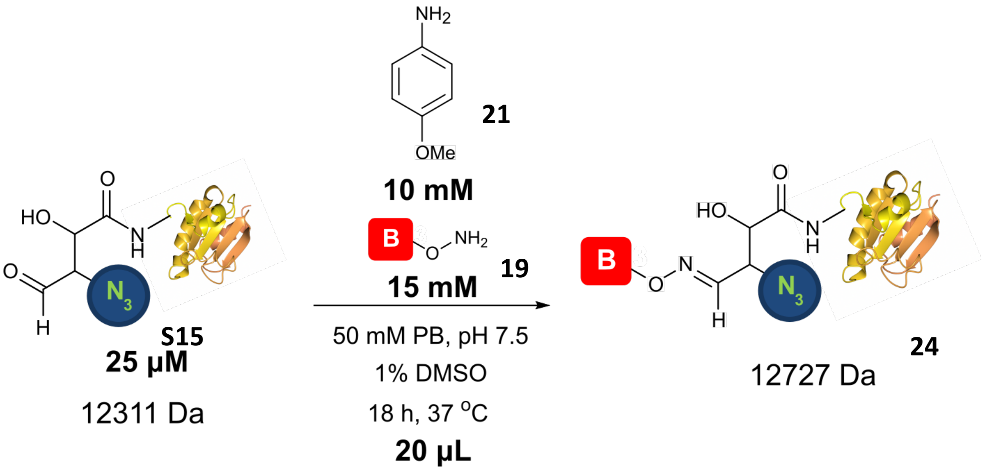

A 120 µL aliquot of 50 µM azide labelled thioredoxin **S15** (prepared as described previously) was desalted using a PD SpinTrap G-25 column (GE Healthcare Life Sciences), eluting with 5 mM PB pH 7.5. A 10 µL aliquot of desalted protein was then charged with 3.8 µL of 0.2 M PB pH 7.5, and 4.8 µL of MQ H_2O. The solution was then charged with 1.2 µL of a 250 mM aminooxy biotin stock **19** solution in 50 mM PB pH 7.5 (pH adjusted to pH 7.5 using 2M NaOH), and then charged with 0.2 µL of a 1M *p*-anisidine **21** stock solution in DMSO. Following mixing by pipetting, the reaction was allowed to sit at 37 °C for 18 h without further

agitation. Successful conversion to dually modified protein **24** was confirmed by Western Blot.

Synthesis of fluorescently labelled, PEGylated myoglobin 26

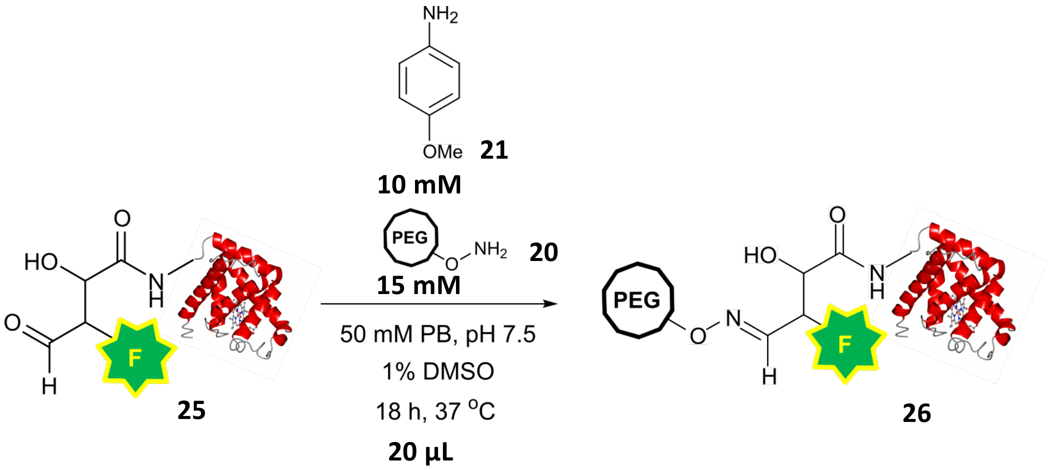

A 120 µL aliquot of 100 µM fluorescently labelled myoglobin **25** (prepared as described previously) was desalted using a PD SpinTrap G-25 column (GE Healthcare Life Sciences), eluting with 5 mM PB pH 7.5. A 10 µL aliquot of desalted protein was then charged with 3.8 µL of 0.2 M PB pH 7.5, and 4.8 µL of MQ H_2O. The solution was then charged with 1.2 µL of a 250 mM aminooxy PEG 2K **20** stock solution in 50 mM PB pH 7.5 (pH adjusted to pH 7.5 using 2M HCl), and then charged with 0.2 µL of a 1 M *p*-anisidine **21** stock solution in DMSO. Following mixing by pipetting, the reaction was allowed to sit at 37 °C for 18 h without further agitation. Successful labelling to give dually modified protein **26** was confirmed SDS PAGE analysis.

Synthesis of azide labelled, biotinylated myoglobin S23

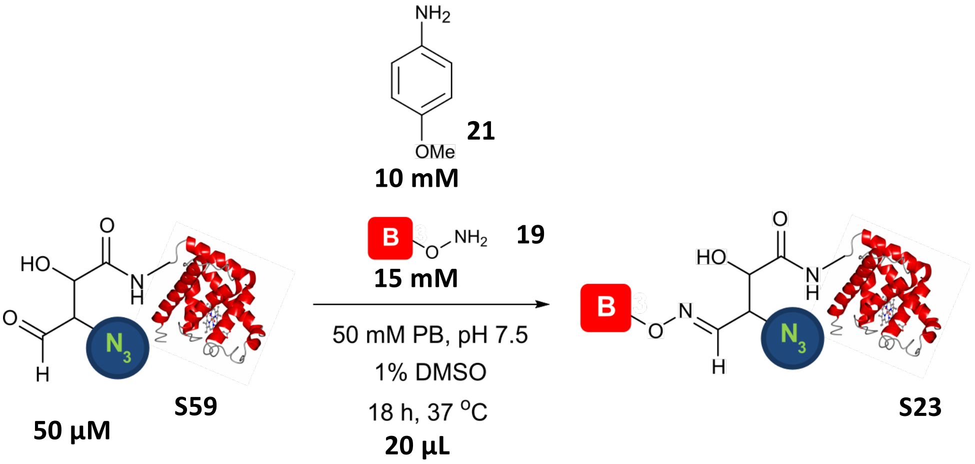

A 120 µL aliquot of 100 µM azide labelled myoglobin **S59** (prepared as described previously) was desalted using a PD SpinTrap G-25 column (GE Healthcare Life Sciences), eluting with 5 mM PB pH 7.5. A 10 µL aliquot of desalted protein was then charged with 3.8 µL of 0.2 M PB pH 7.5, and 4.8 µL of MQ H_2O. The solution was then charged with 1.2 µL of a 250 mM aminooxy biotin stock **19** solution in 50 mM PB pH 7.5 (pH adjusted to pH 7.5 using 2M HCl), and then charged with 0.2 µL of a 1 M *p*-anisidine **21** stock solution in DMSO. Following mixing by pipetting, the reaction was allowed to sit at 37 °C for 18 h without further

agitation. Successful coversion to give dually modified protein **S23** (~70%) was confirmed LC-MS analysis.

Synthesis of dually acylated HASPA 33

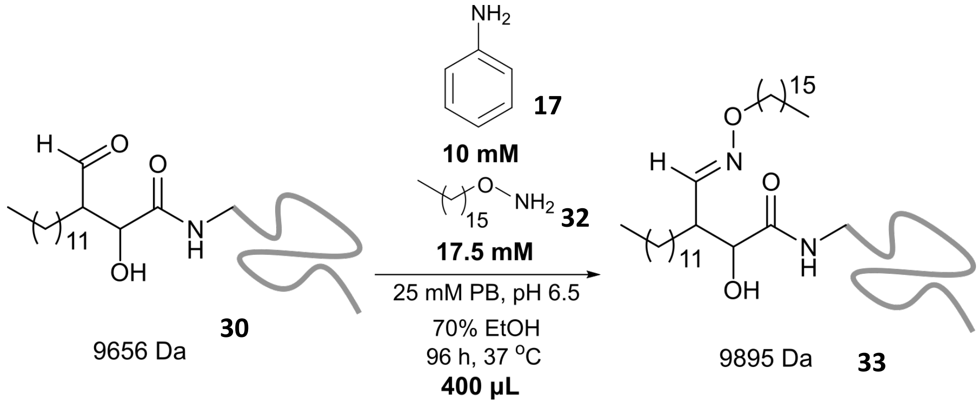

Prior to dual modification, samples of chemically myristoylated HASPA **30** (prepared as described earlier) were pooled to give an estimated maximum protein content of 160 μg (based on initial HASPA protein concentration). The pooled samples were diluted to >20% DMSO content, purified via PD MiniTrap G-25 columns (eluting into water), and subsequently lyophilised to give a white powder that was stored at -80 °C until required. For the dual acylation of HASPA, the lyophilised aliquot of chemically myristoylated HASPA **30** was resuspended in 1 x PBS buffer (120 μL, pH 7.4), and then buffered exchanged using a PD SpinTrap G-25 column (GE Healthcare Life Sciences, eluting into 25 mM PB pH 6.5). The solution was then charged with 280 μL of 25 mM palmitoyl aminooxy **32** in EtOH, and then charged with 3.6 μL of aniline **17**. The solution was briefly vortexed, and the reaction was allowed to sit at 37 °C for 96 h without further agitation. After 96h ~80% conversion to **33** was estimated by LC-MS.

7. Mass spectrometry data of modified peptides

*SLYRAG **S43***
Calculated $[M+H]^+$ *= 666.36*
Found $[M+ H]^+$ *= 666.32*

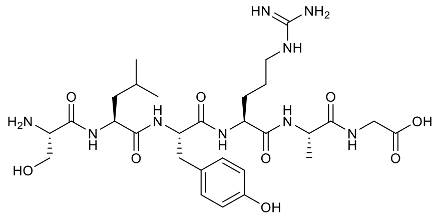

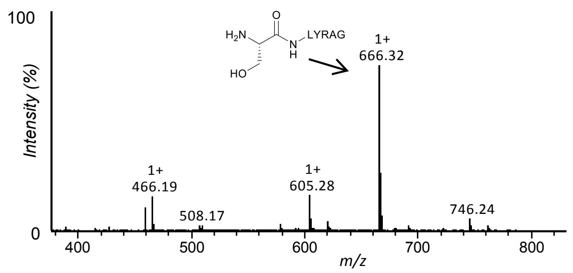

*Glyoxyl-LYRAG **8***
Calculated $[M+H]^+$ *= 635.31 (ald), 653.32 (hyd)*
Found $[M+ H]^+$ *= 635.41 (ald), 653.44 (hyd)*

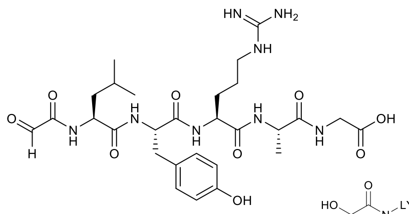

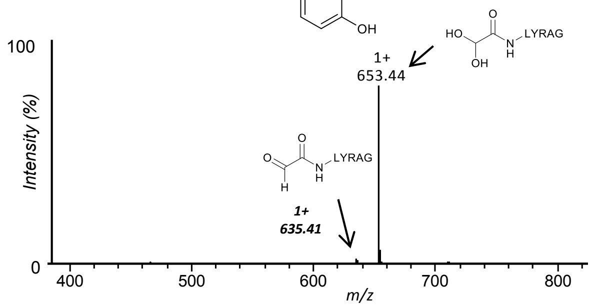

*Aldol-LYRAG **S7***
Calculated $[M+H]^+$ *= 707.36*
Found $[M+ H]^+$ *= 707.26*

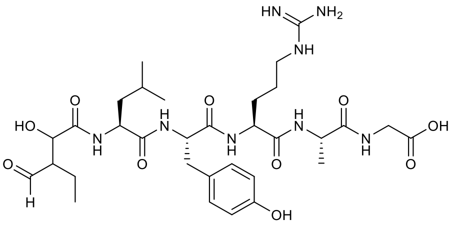

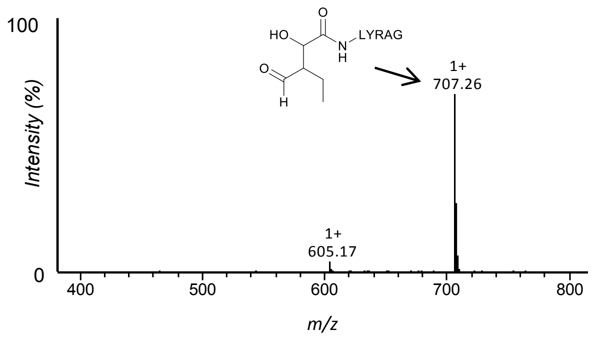

*Aldol-Oxime-LYRAG **S19***
Calculated $[M+H]^+$ *= 812.42*
Found $[M+ H]^+$ *= 812.40*

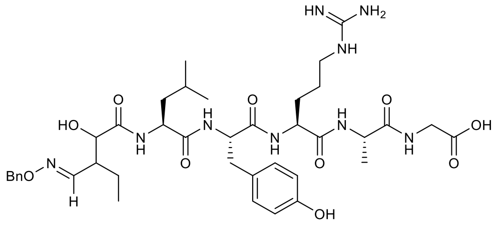

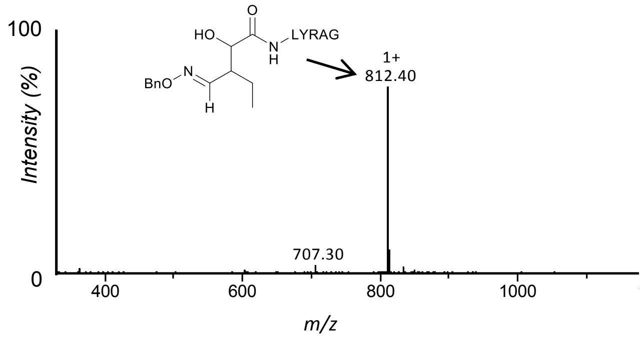

Aldol-LYRAG ***16***

Calculated $[M+H]^+$ = 755.36

Found $[M+ H]^+$ = 755.39

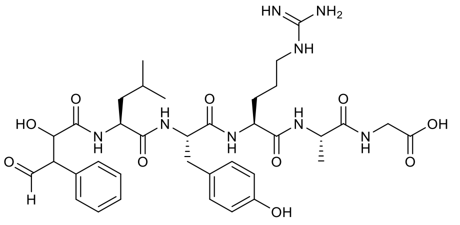

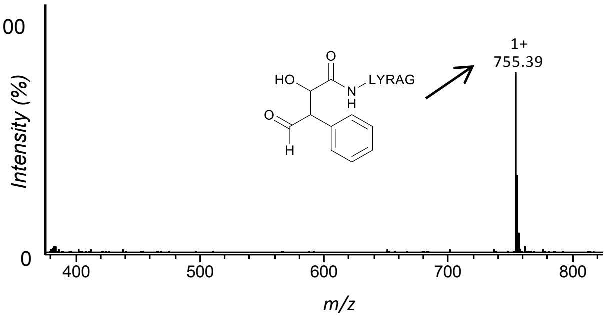

Aldol-IPS ***S17***

Calculated $[M+H]^+$ = 985.47

Found $[M+ H]^+$ = 985.45

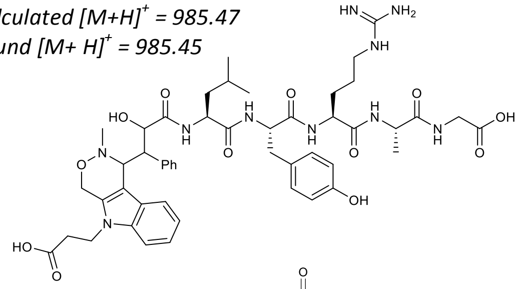

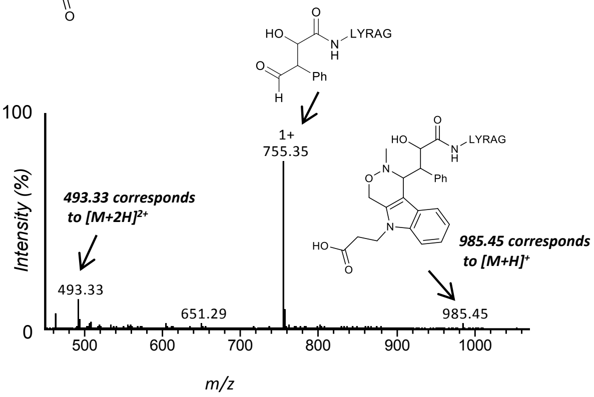

Aldol-ABAO ***S18***

Calculated $[M+H]^+$ = 888.43

Found $[M+ H]^+$ = 888.44

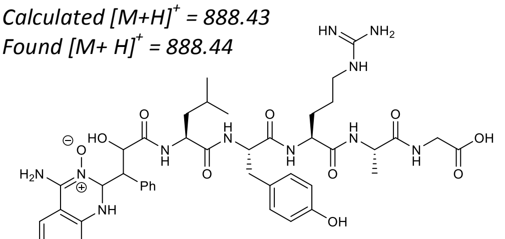

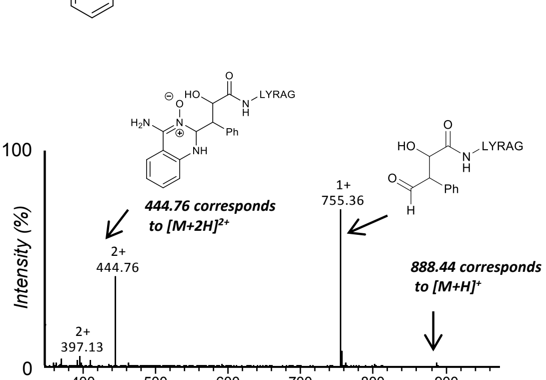

Aldol-Oxime-LYRAG ***18***

Calculated $[M+H]^+$ = 860.42

Found $[M+ H]^+$ = 860.57

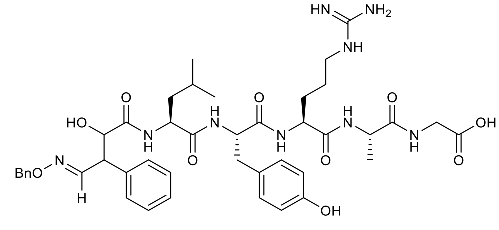

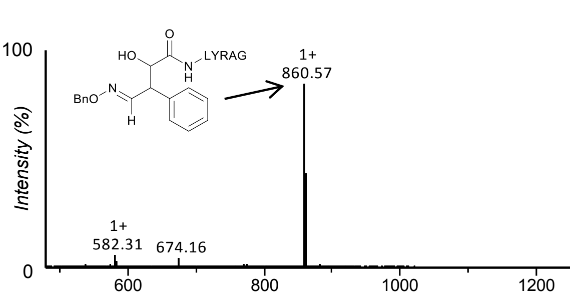

8. Mass spectrometry data of proteins and modified proteins

Horse Heart Myoglobin ***S1***

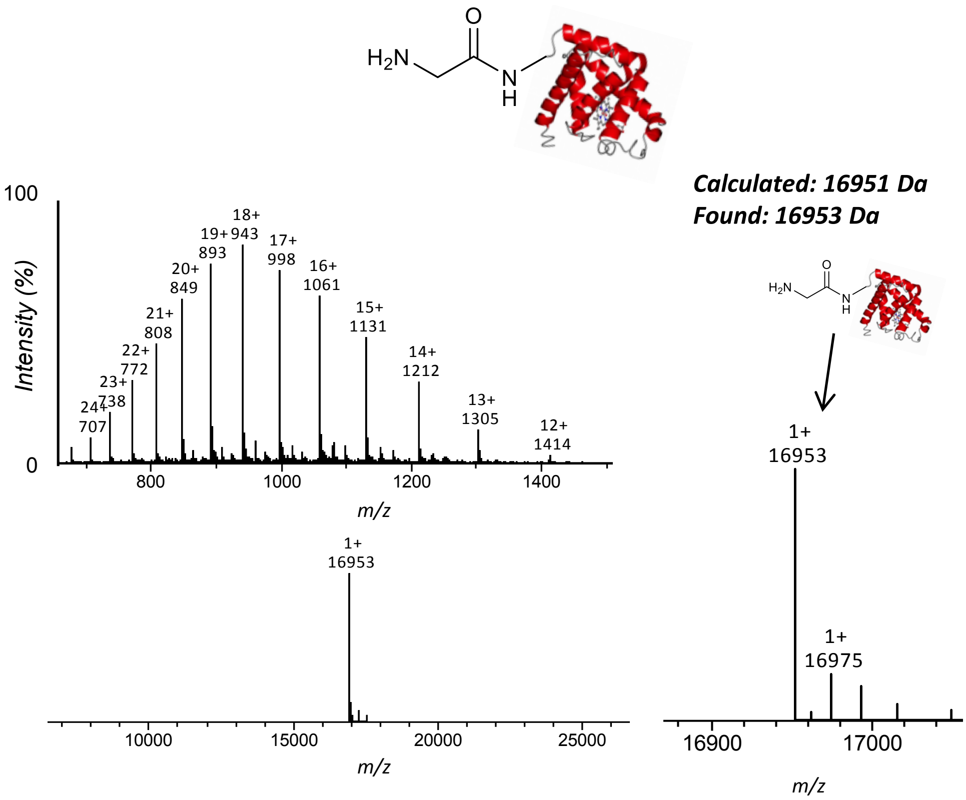

Calculated: 16951 Da
Found: 16953 Da

Glyoxyl-myoglobin ***5***

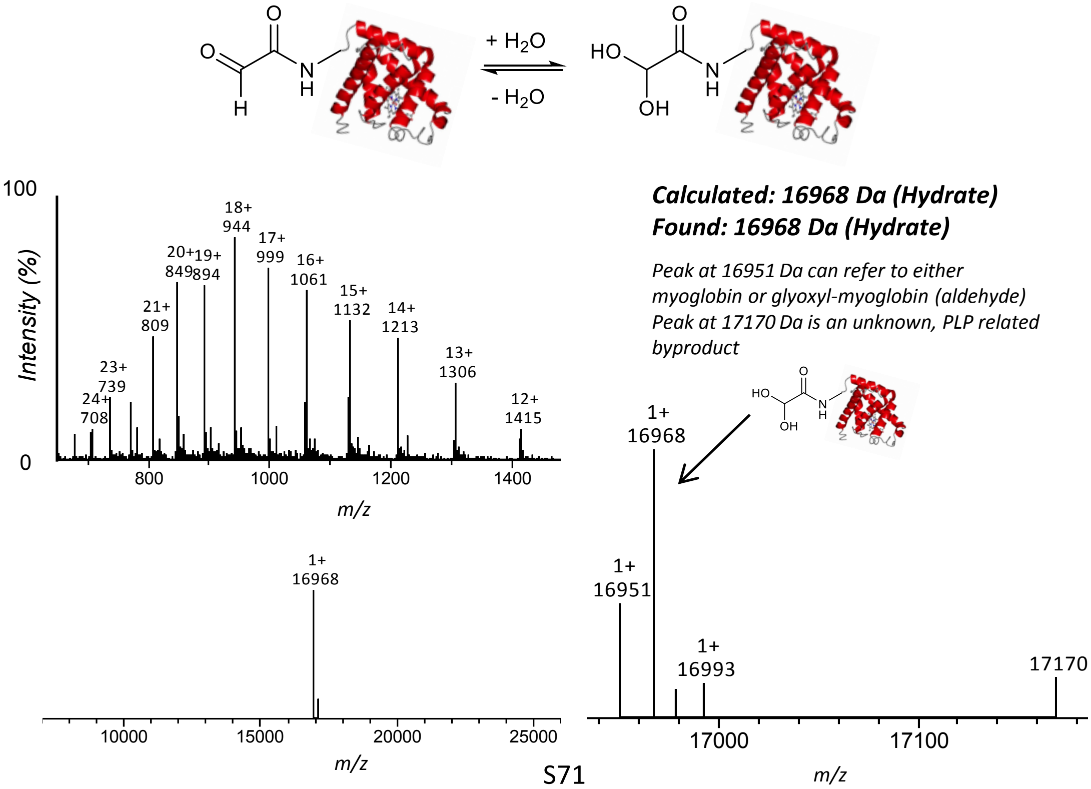

Calculated: 16968 Da (Hydrate)
Found: 16968 Da (Hydrate)

Peak at 16951 Da can refer to either myoglobin or glyoxyl-myoglobin (aldehyde)
Peak at 17170 Da is an unknown, PLP related byproduct

Aldol-myolglobin ***S2***

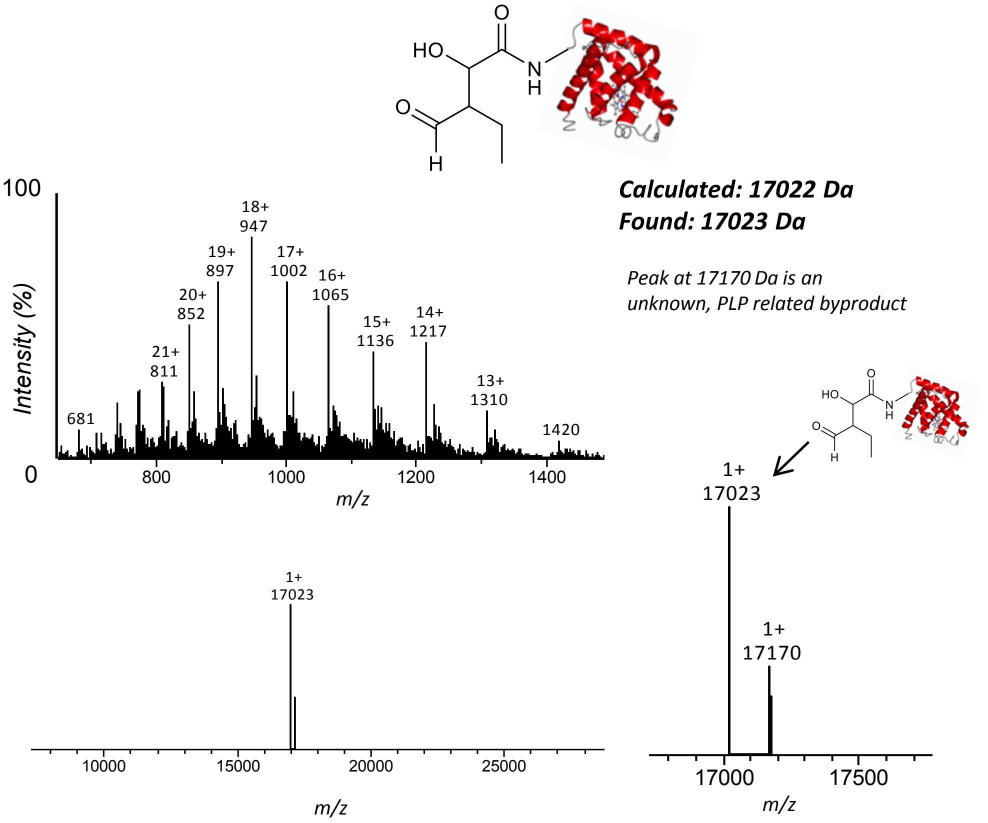

Thioredoxin ***S24***

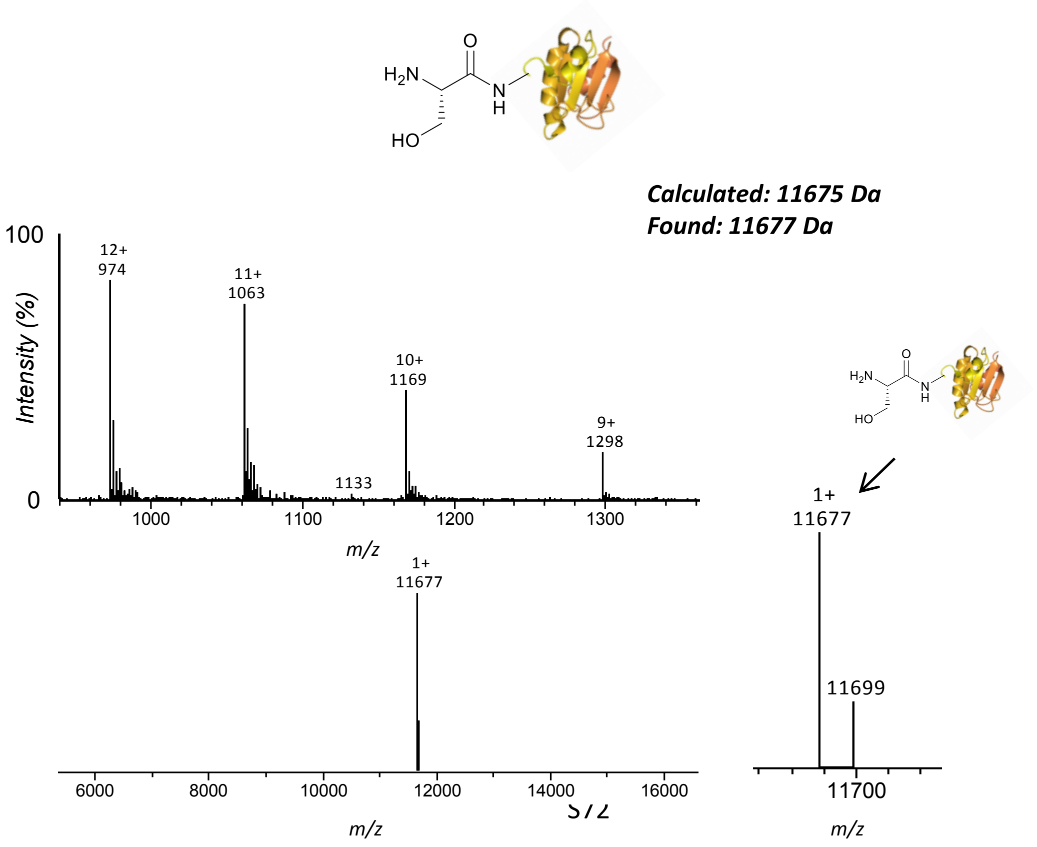

Glyoxyl-thioredoxin ***6***

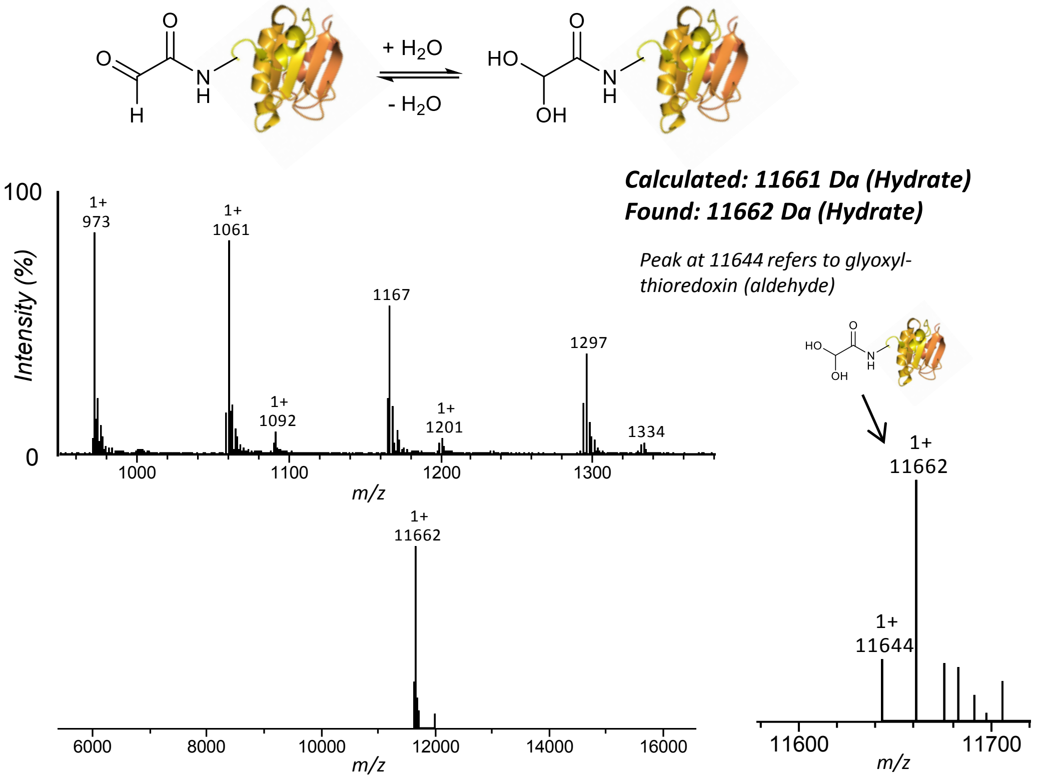

Aldol-thioredoxin ***S8***

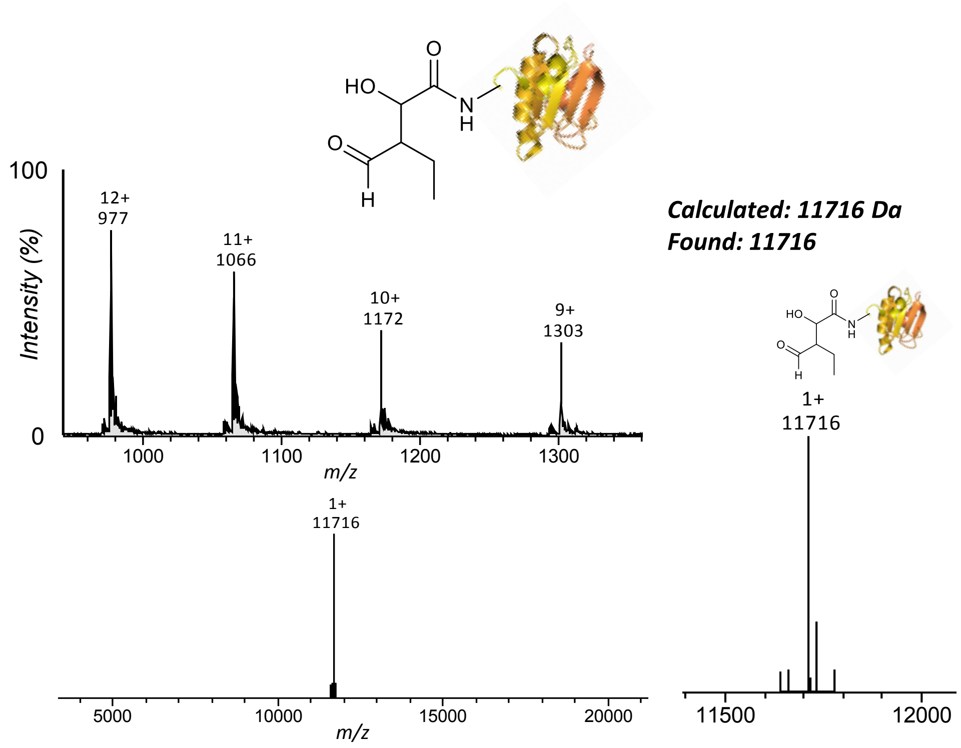

Glyoxyl-GFP (Y39CycloOctK) ***S50***

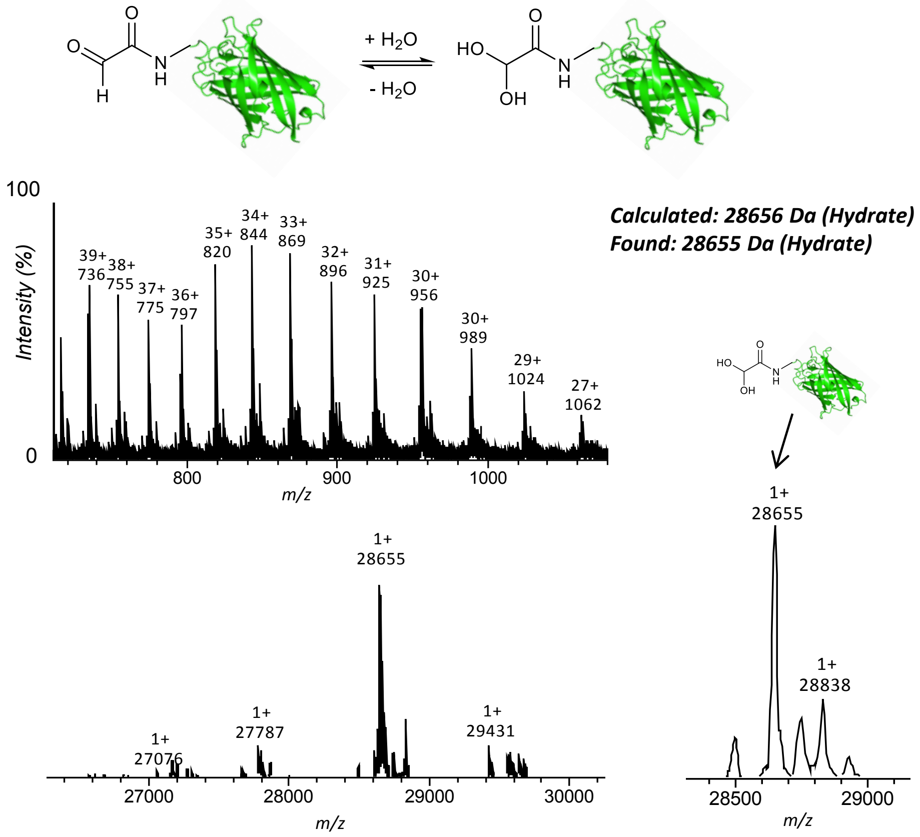

sfGFP(ThzK150Oxo) ***S13***

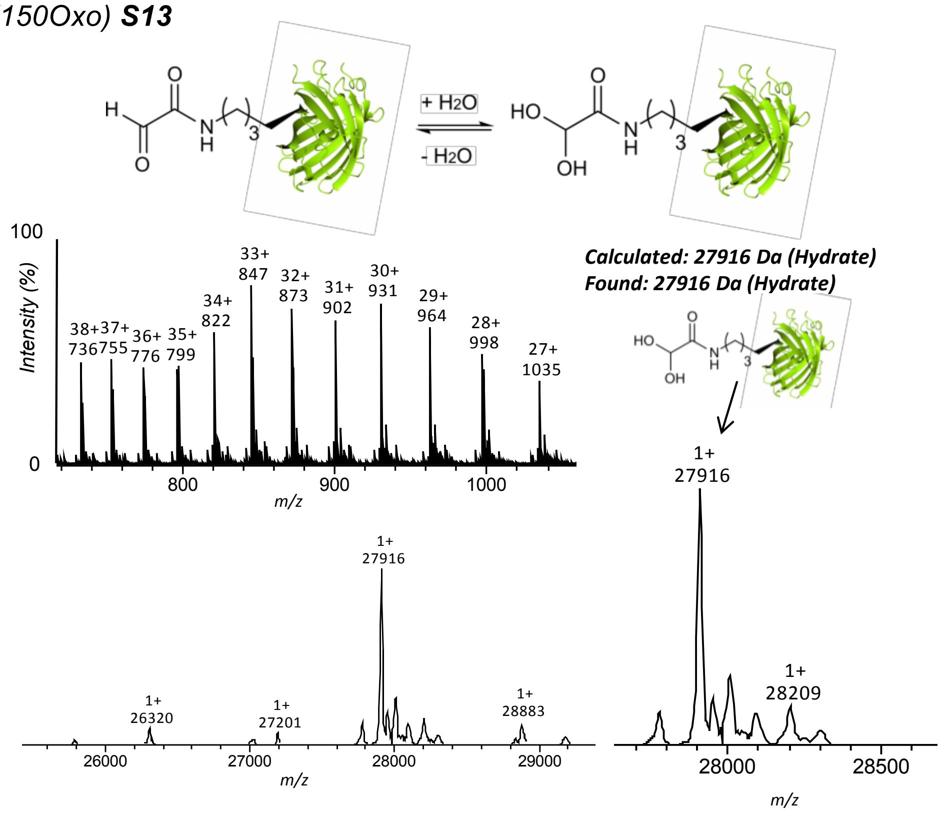

GFP(ThzK39Oxo) ***S14***

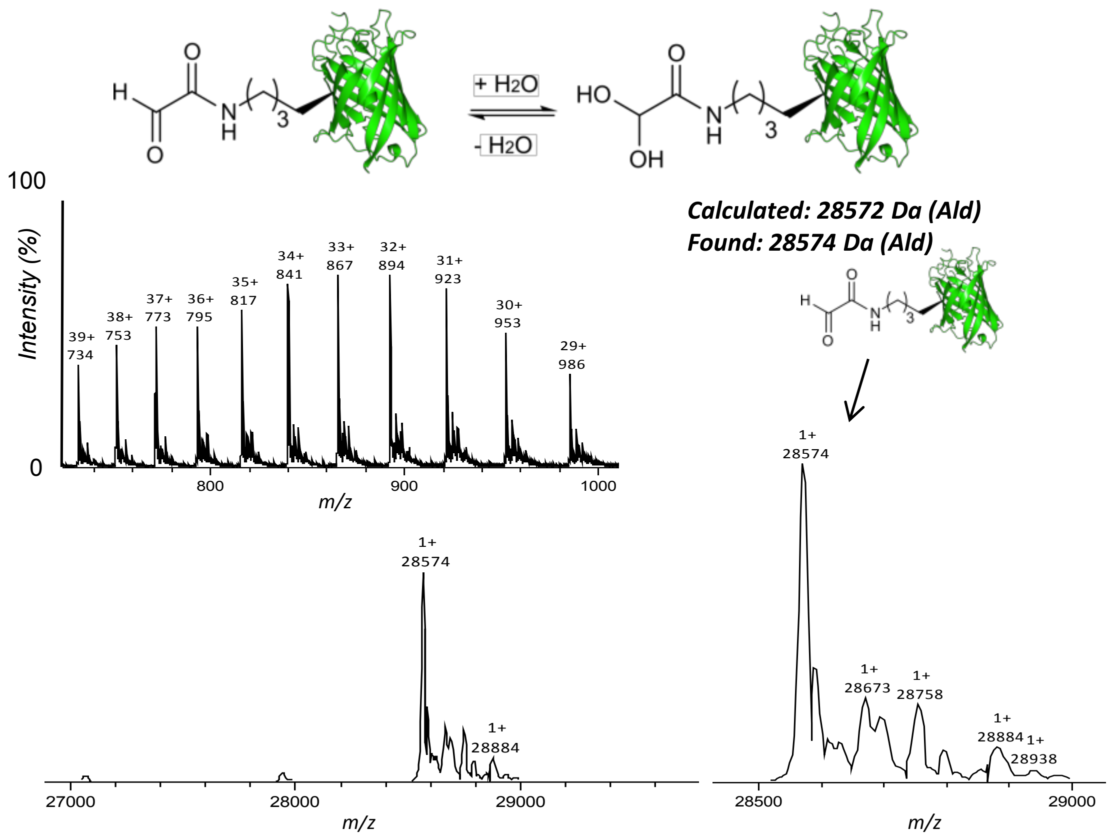

Hydrophilic acylated surface protein A (HASPA) ***S27***

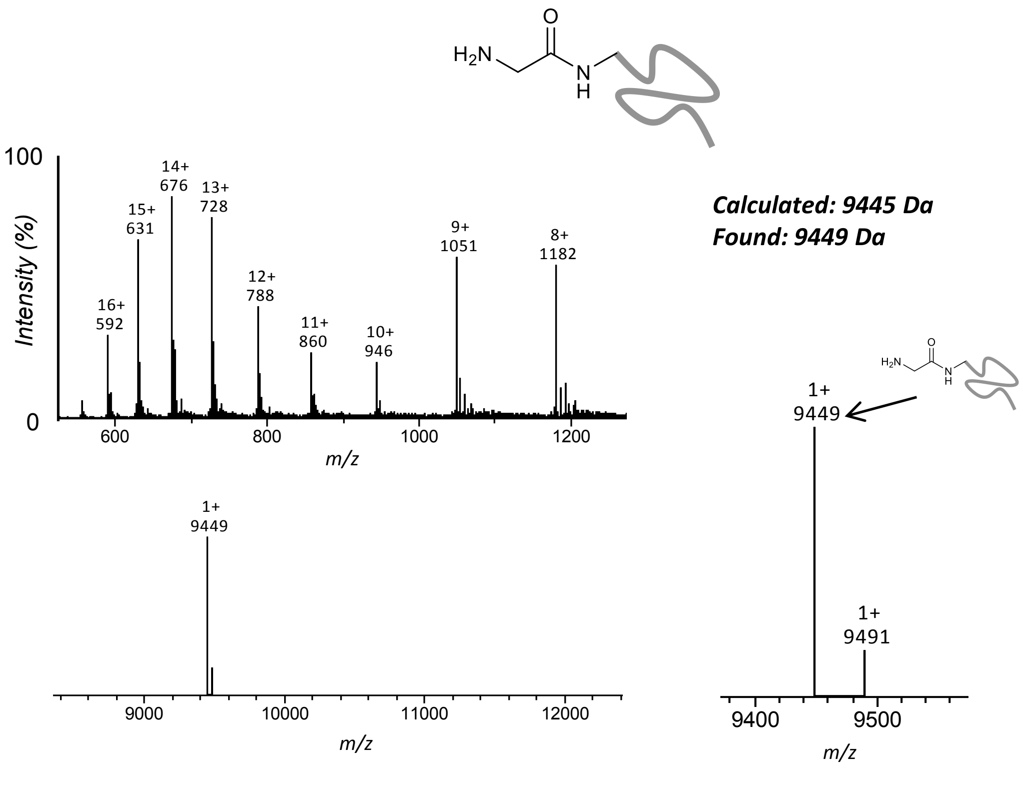

HASPA(G1S) ***31***

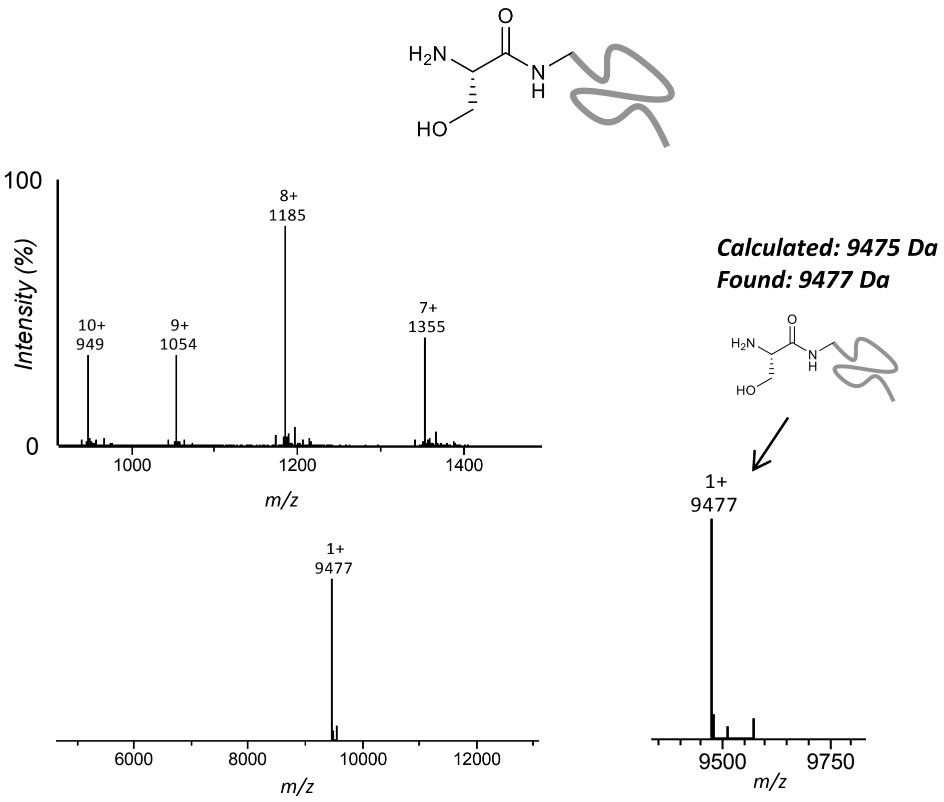

Glyoxyl-HASPA(G1S) ***S26***

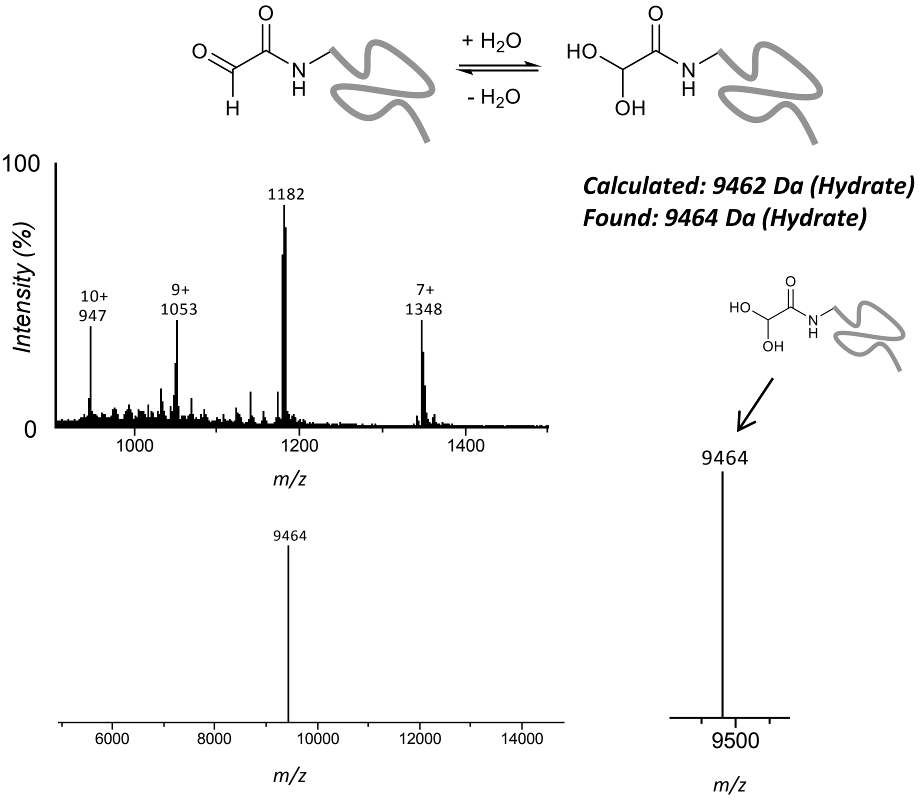

[15N]HASPA(G1S) ***31-15N***

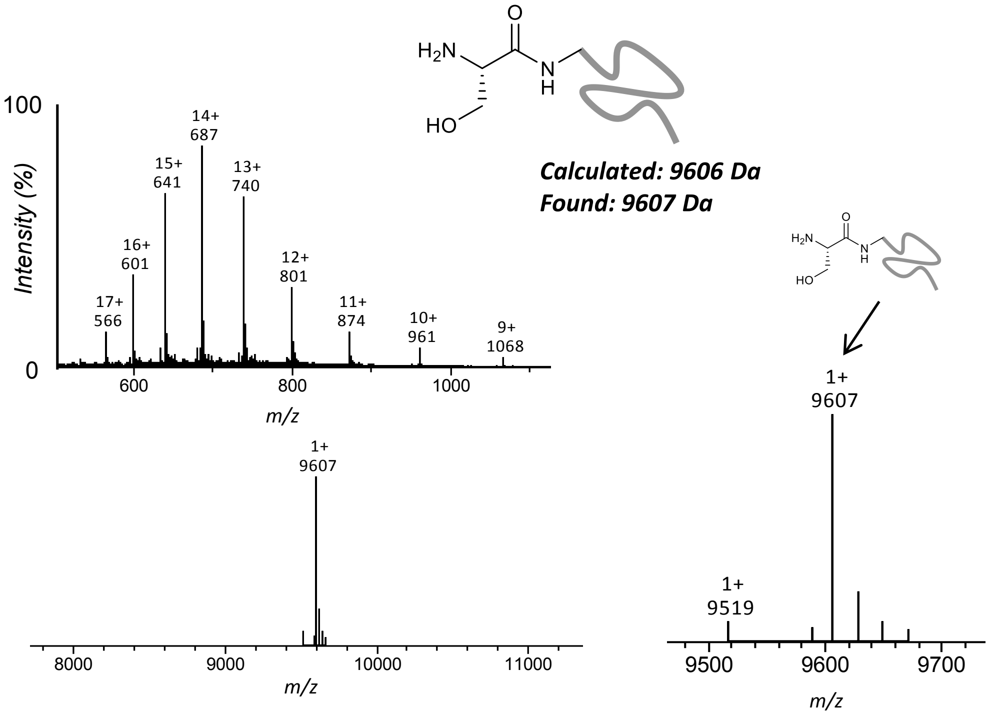

Glyoxyl-[15N]HASPA(G1S) ***S26-15N***

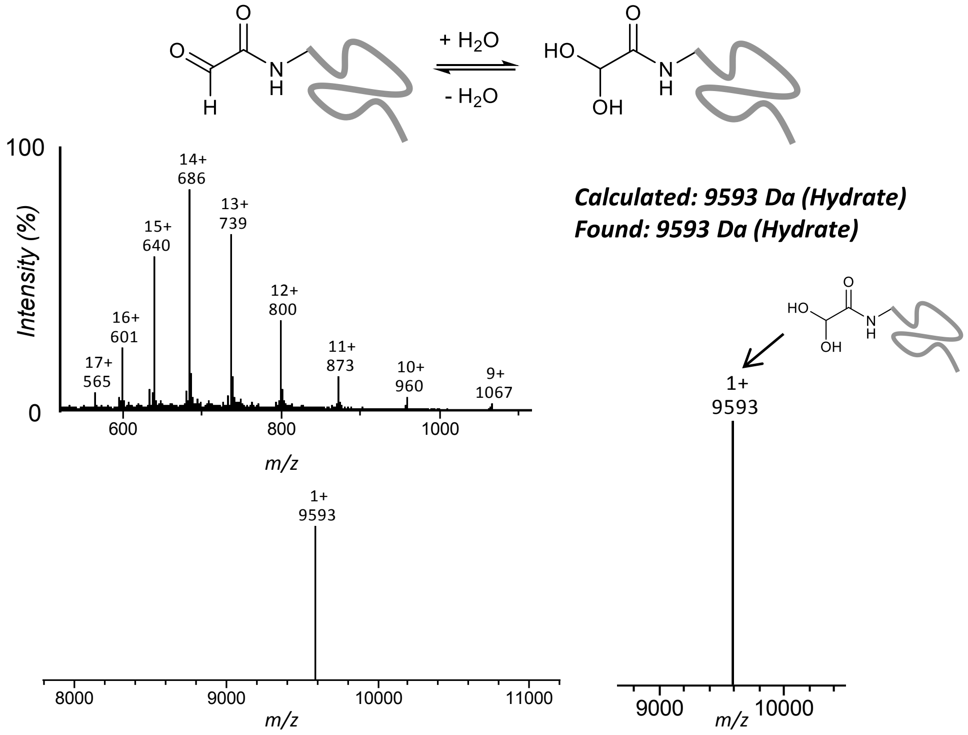

Fluroescently tagged thioredoxin ***23***

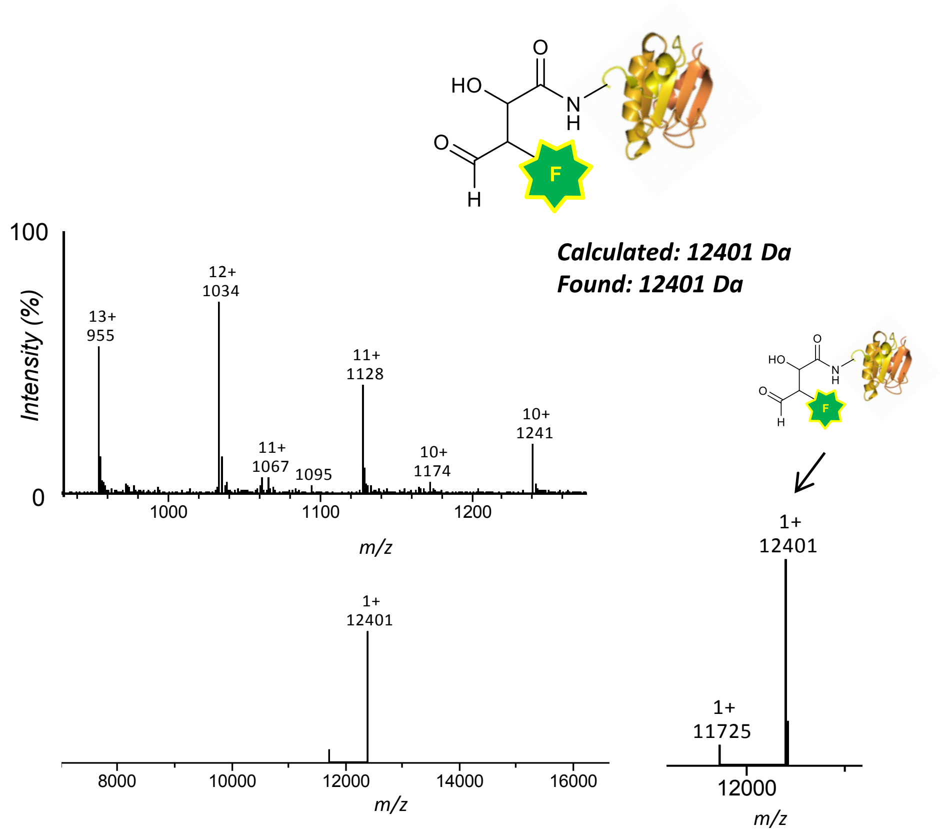

Biotin tagged thioredoxin ***S51***

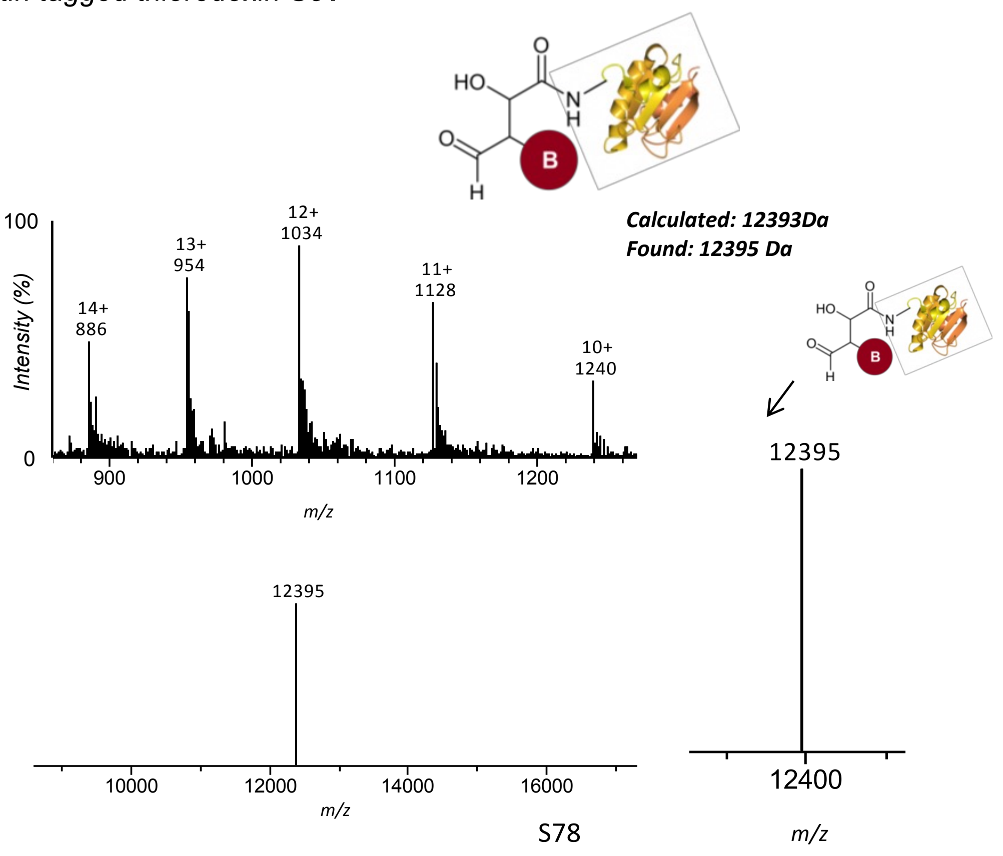

Azide tagged thioredoxin ***S15***

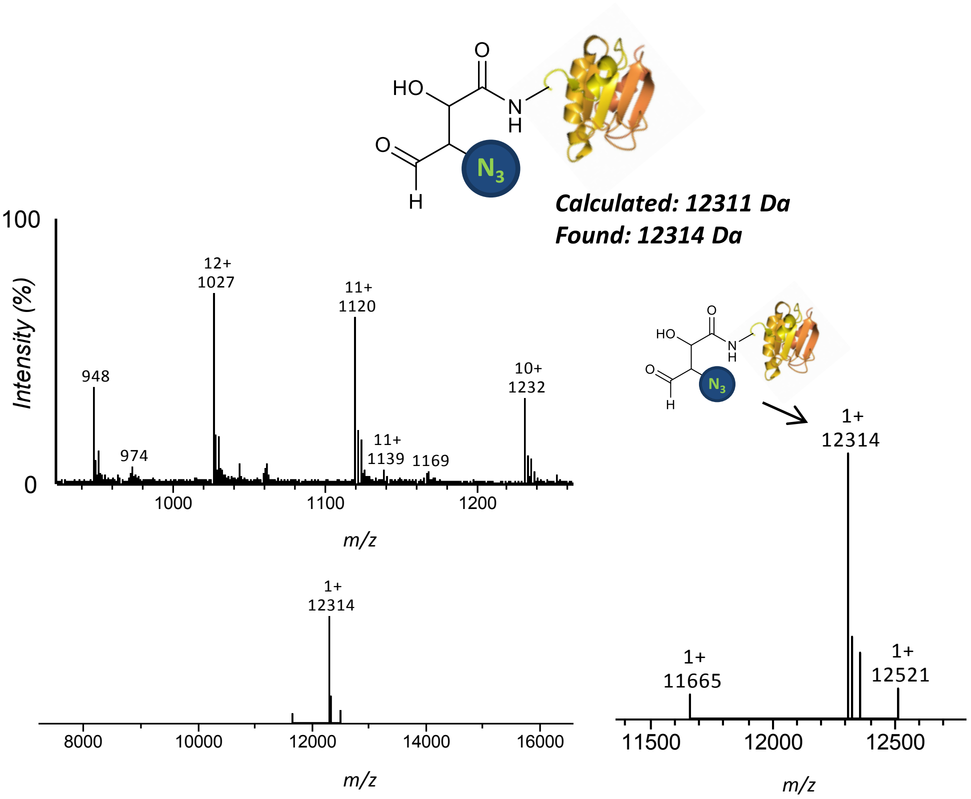

Fluorescently labelled myoglobin ***25***

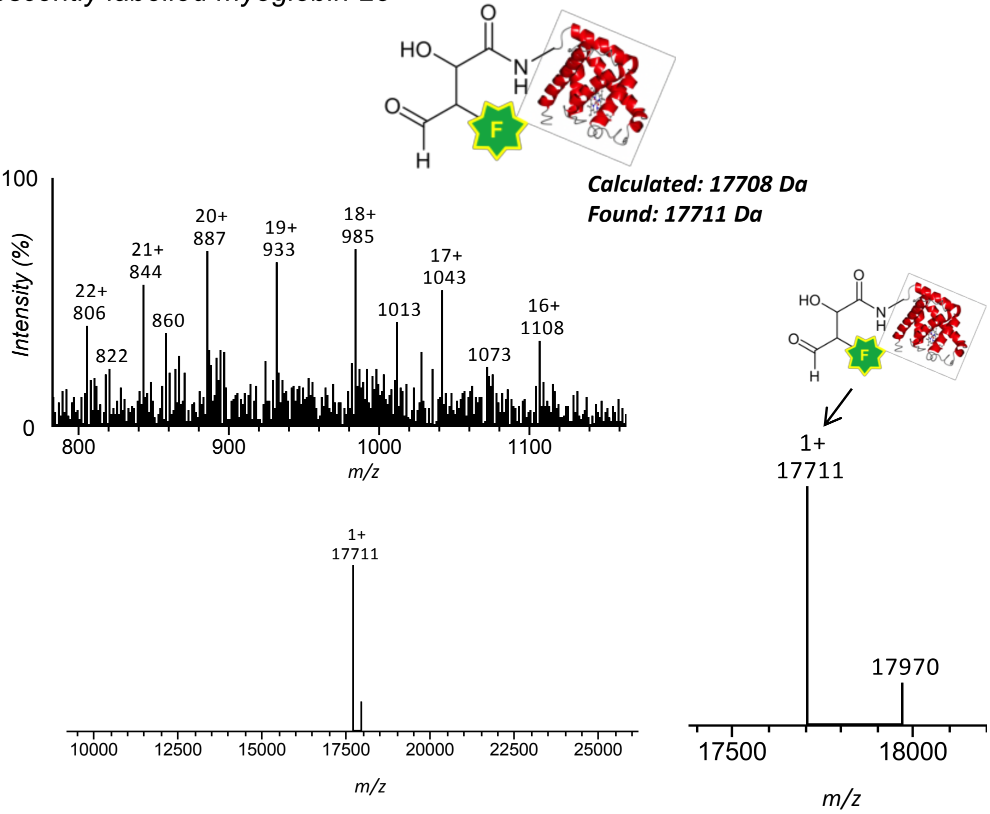

Azide tagged myoglobin ***S59***

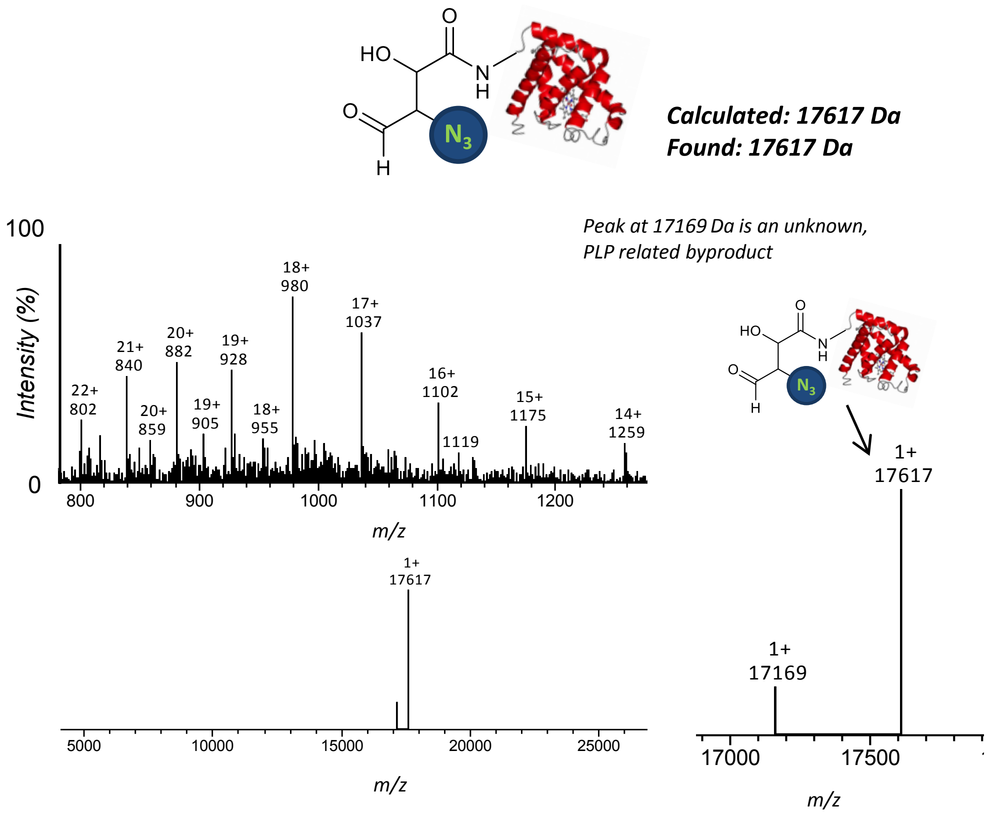

Biotin tagged HASPA(G1S) ***S56***

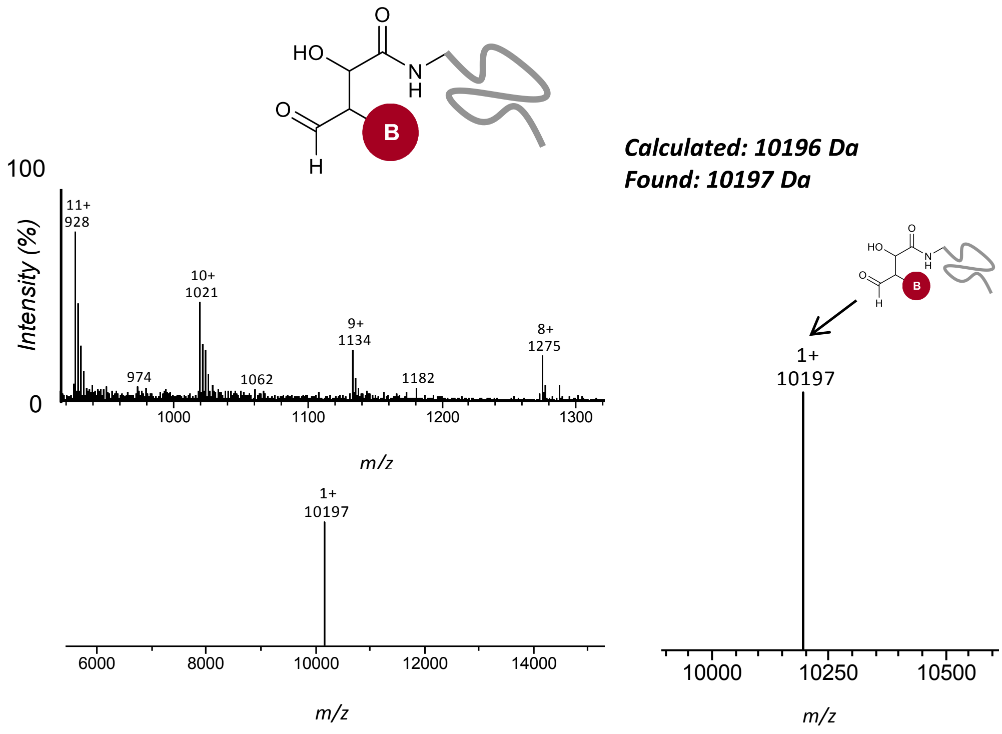

Biotin tagged GFP (Y39CycloOctK) ***S53***

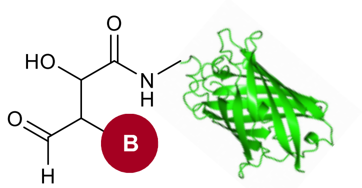

Calculated: 29388 Da
Found: 29388 Da

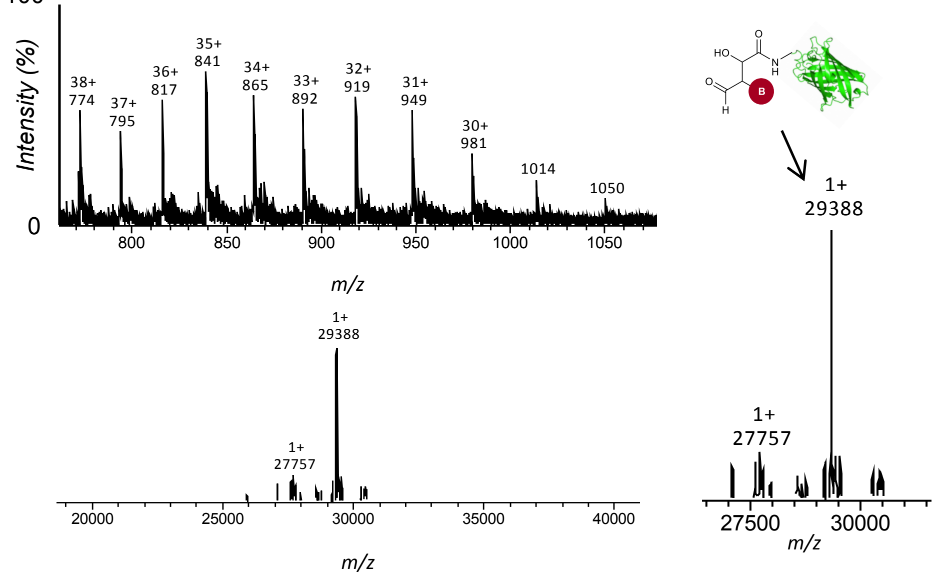

Folate tagged GFP (Y39CycloOctK) ***S52***

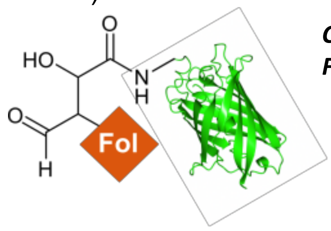

Calculated: 29730 Da
Found: 29731 Da

Peak at 1093 Da corresponds to unreacted probe that is present after SpinTrap purification. Consider further methods of purification if purer protein samples are required for downstream use.

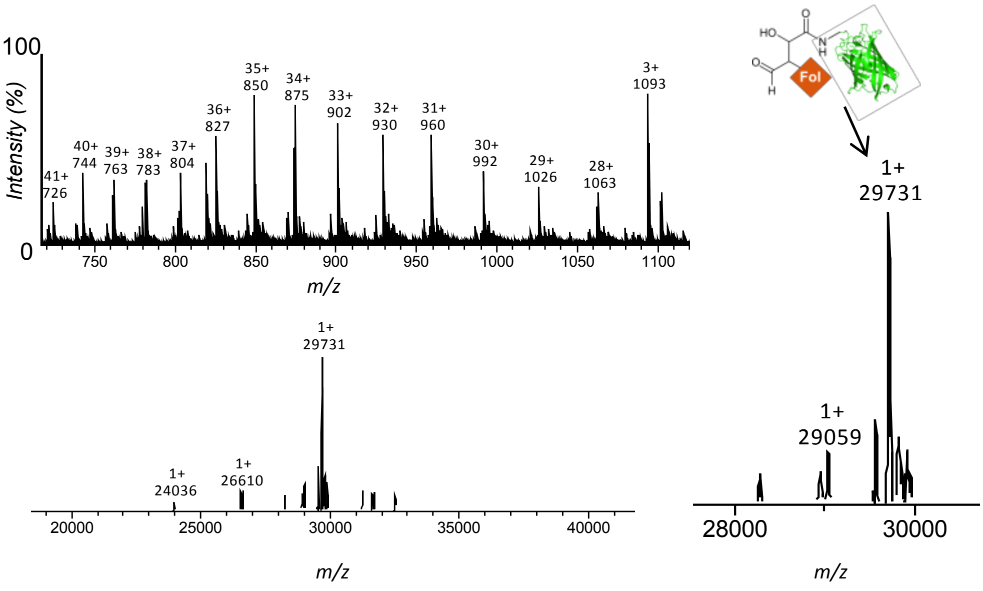

Internal (position 150) azide labelled sfGFP ***S54***

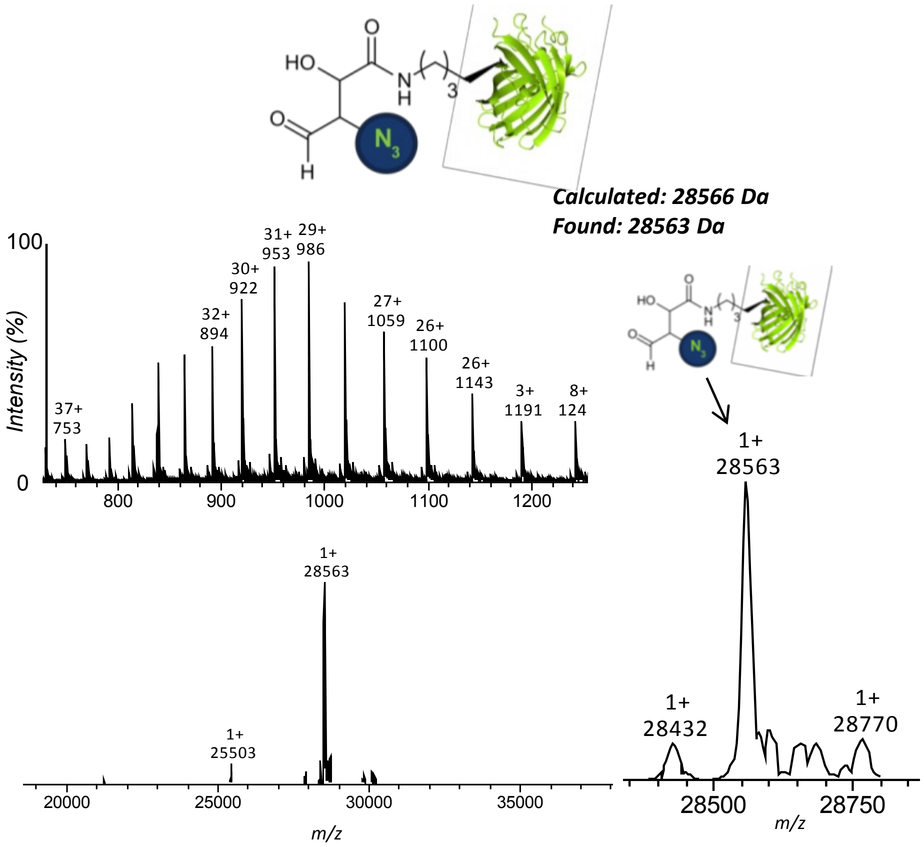

Internal (position 39) azide labelled GFP ***S55***

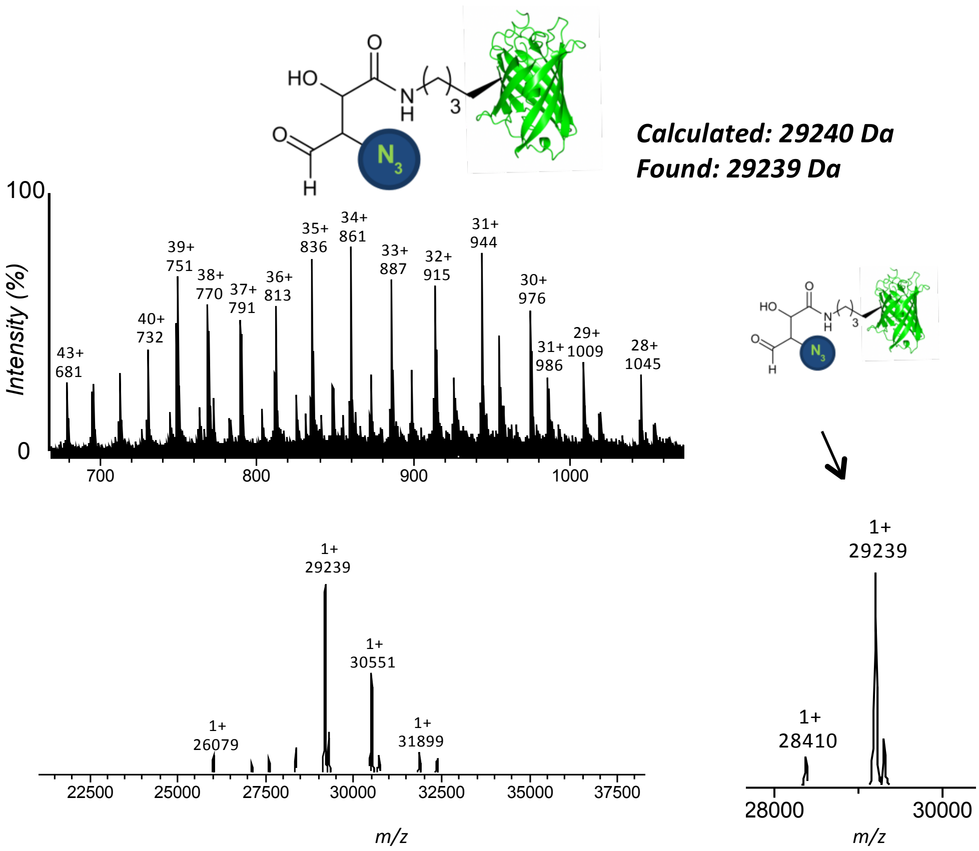

Chemically myristoylated HASPA(G1S) ***30***

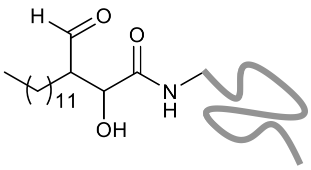

Calculated: 9656 Da
Found: 9657 Da

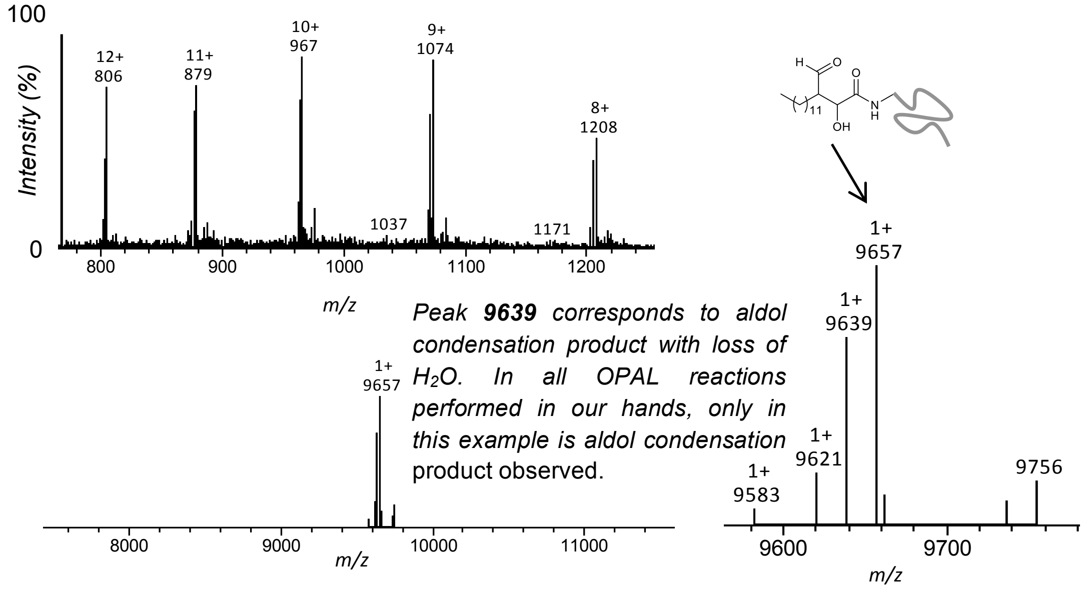

Chemically myristoylated [15N]HASPA(G1S) ***30-15N***

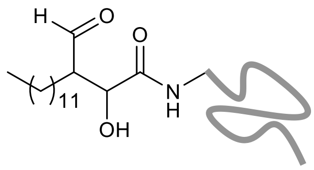

Calculated: 9787 Da
Found: 9786 Da

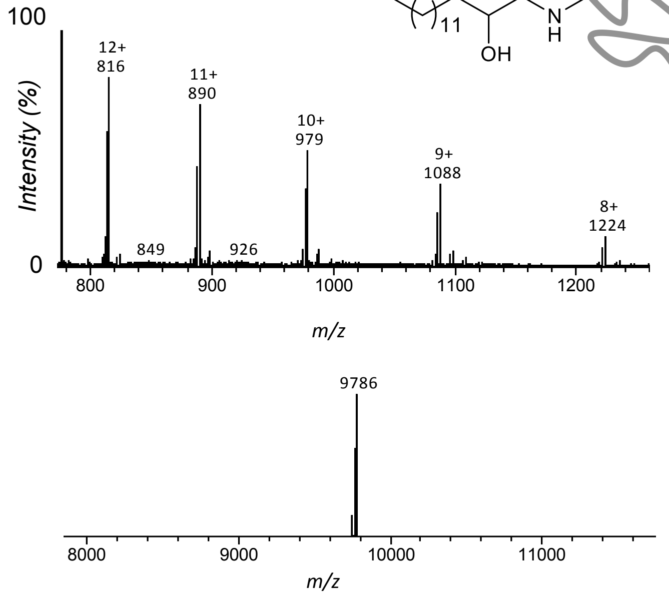

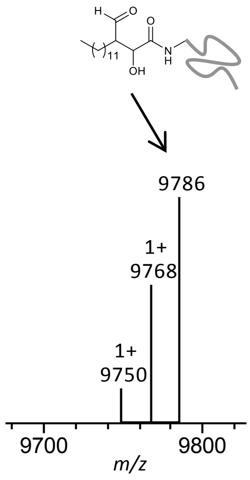

Azide tagged HASPA(G1S) ***S57***

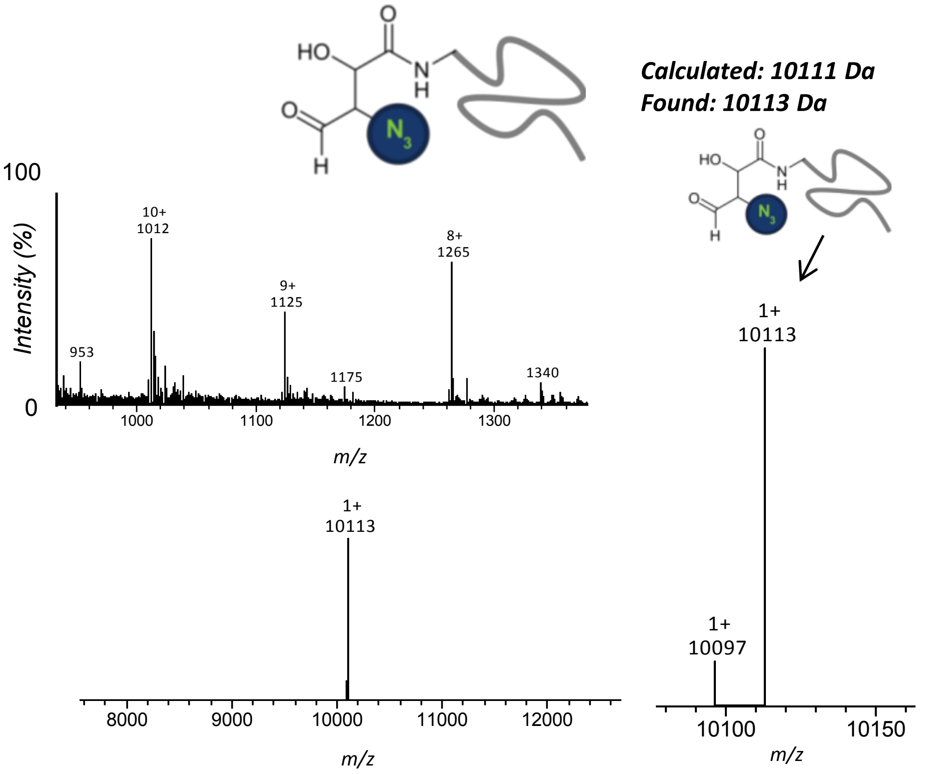

Fluorescently tagged [15N]HASPA(G1S) ***S60-15N***

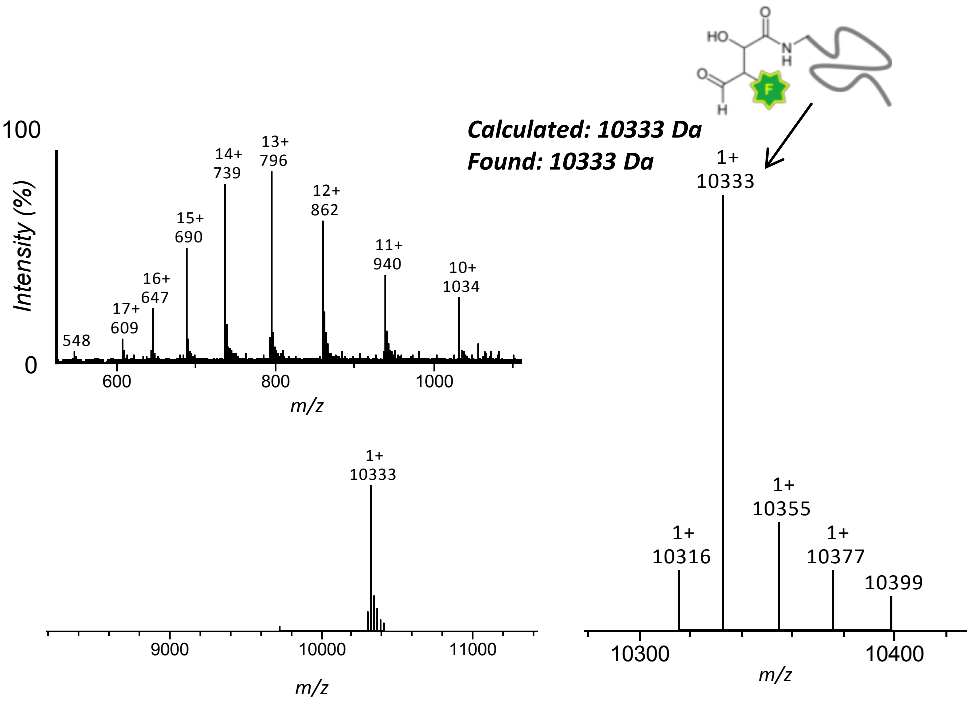

Internal (position 150) biotinylated sfGFP ***S61***

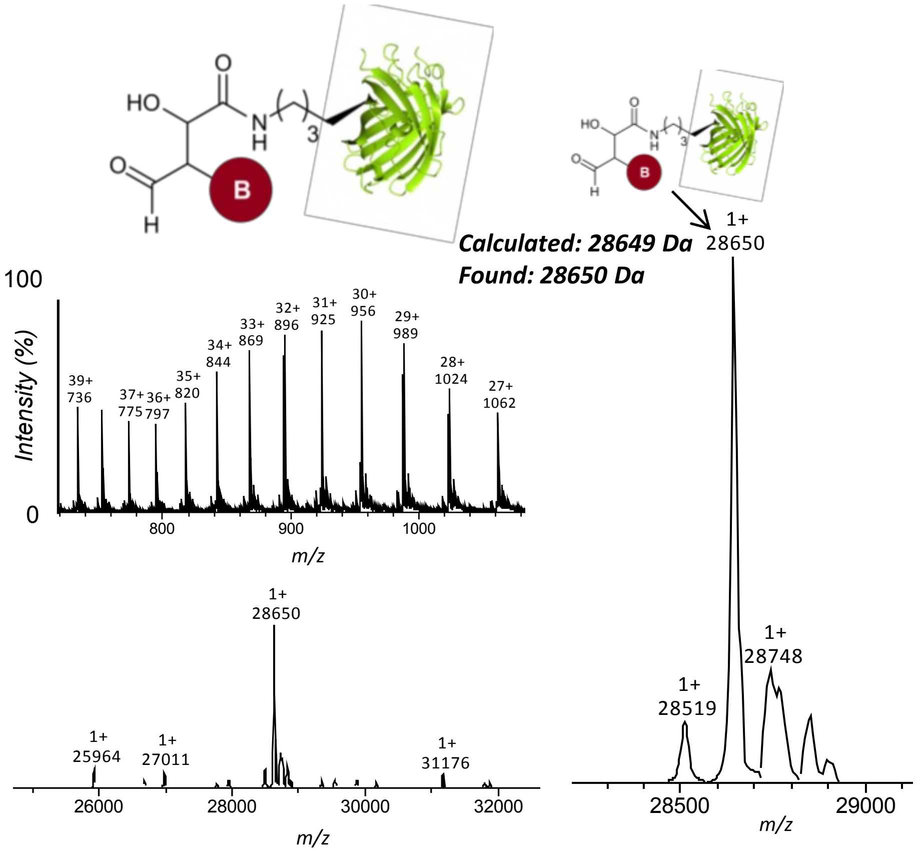

Fluorescently labelled, biotinylated thioredoxin ***22***

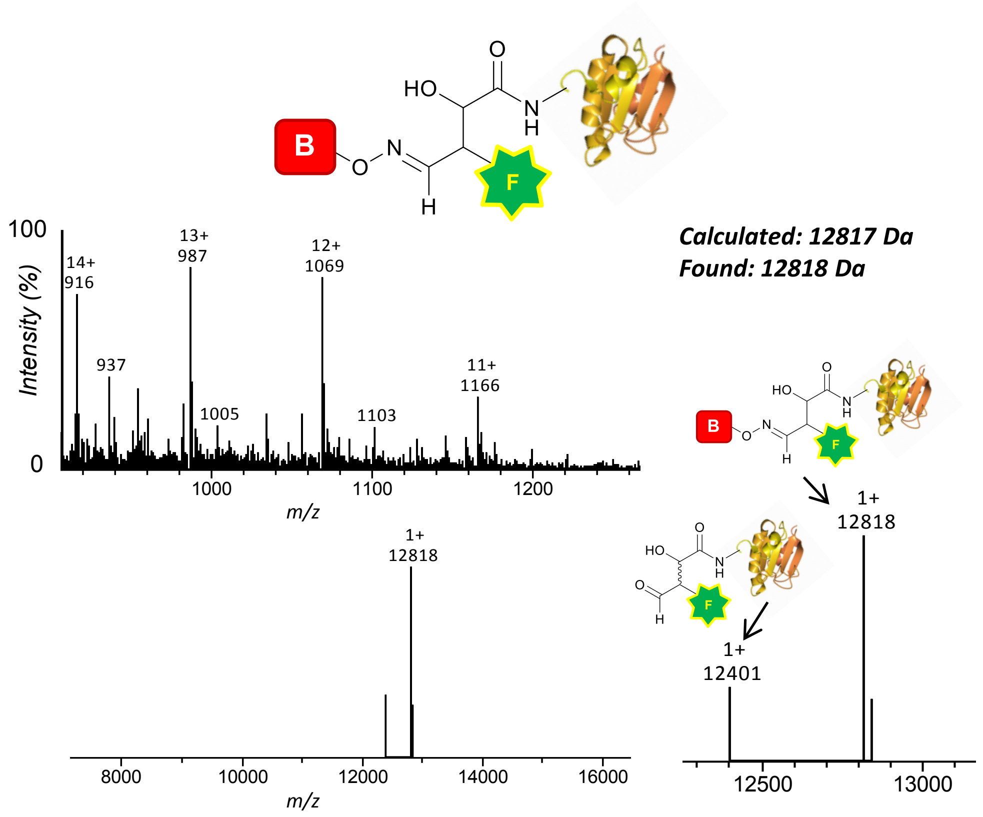

Azide labelled, biotinylated myoglobin ***S23***

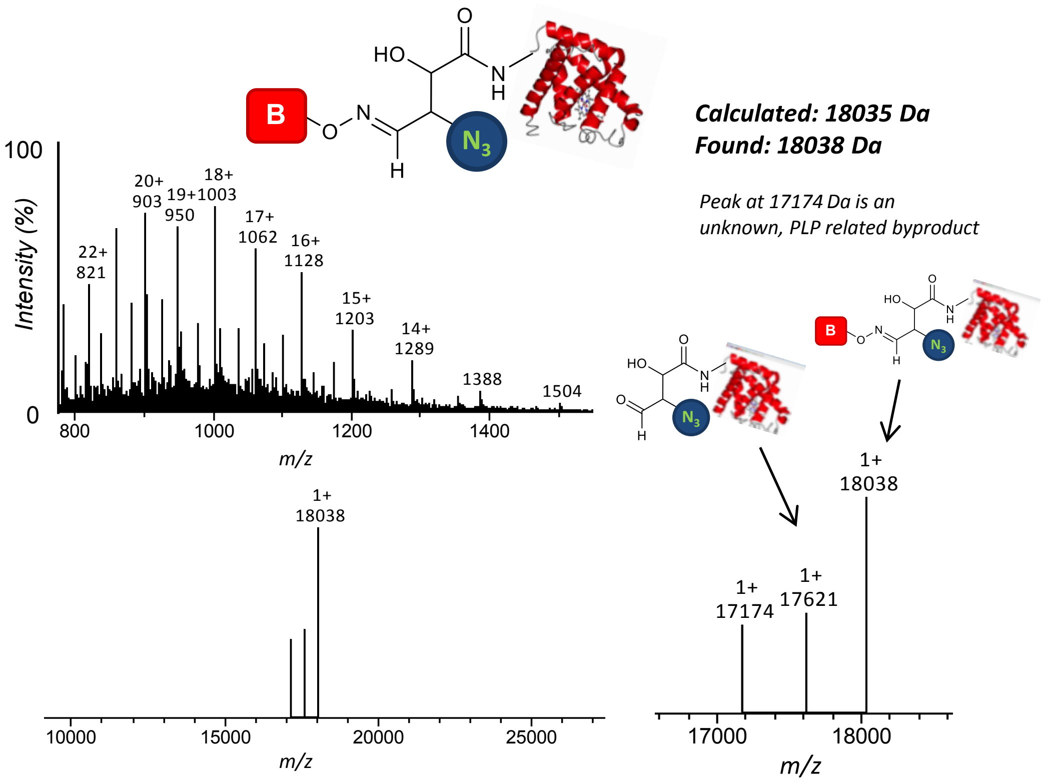

Dually acylated HASPA ***33***

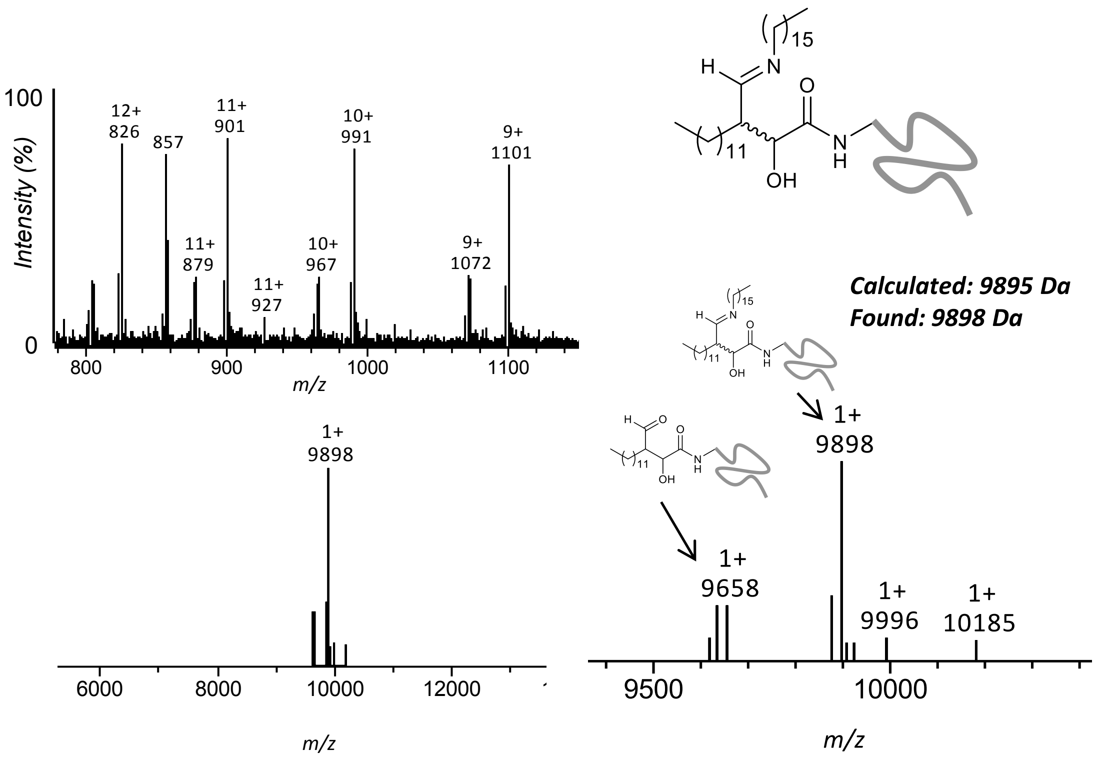

9. Tandem mass spectrometry data of aldol-oxime modified peptide

Note on peptide nomenclature: For all analyses of MS/MS data of aldol/dually modified peptide/protein products, all peptides are treated as ‘H_2N-LYRAG-OH’ species that have been modified at their *N*-terminus. This allows for simplification of the MS/MS data, and is in line conventional peptide fragmentation analysis.

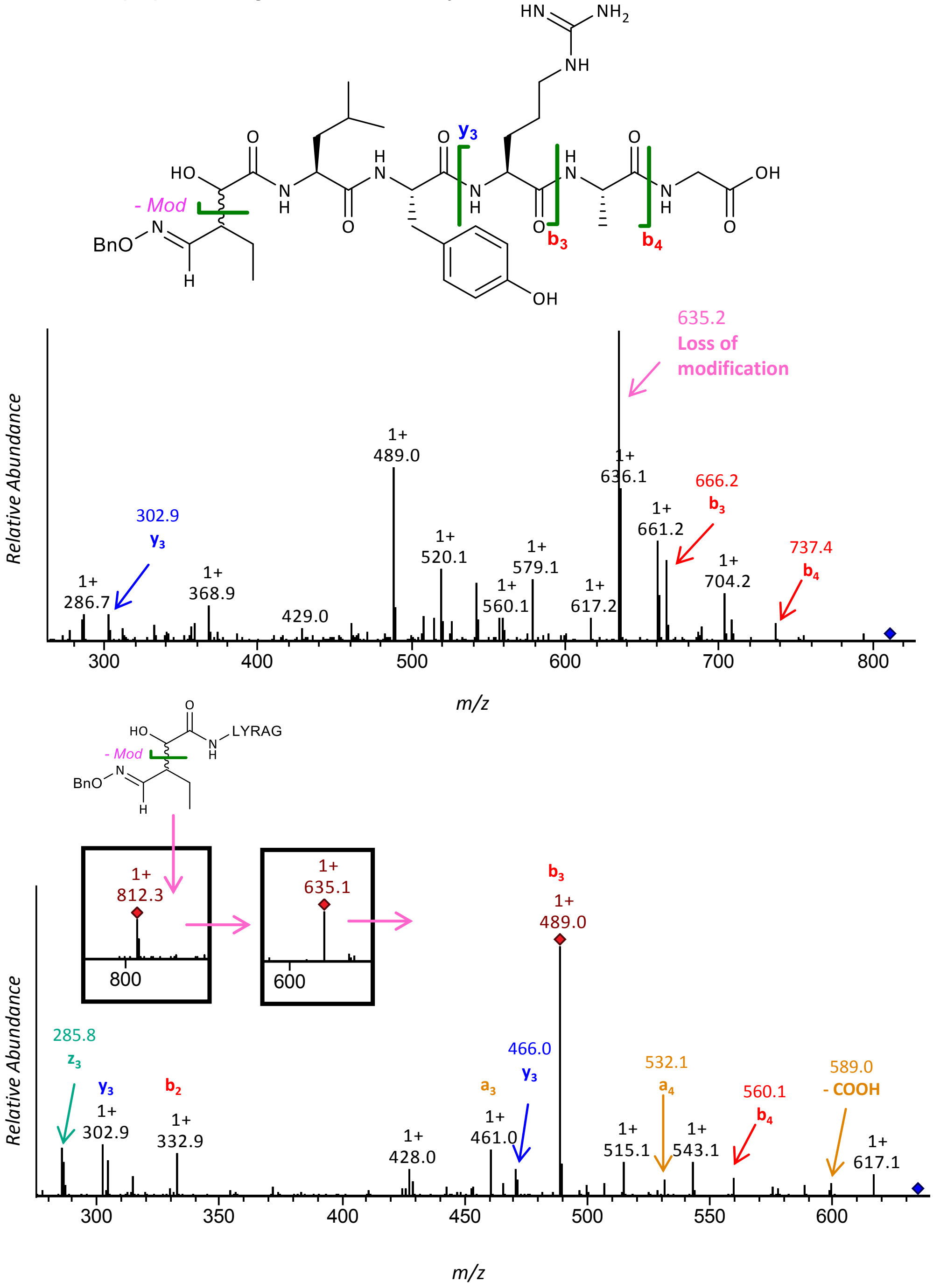

Supplementary Figure 29. a) MS/MS data of **S19**. The major peak corresponds to a loss of 177 Da, corresponding to losing both the aldol and oxime modifications at the *N*-terminus. b) MS/MS, followed by MS/MS of the major fragment of **S19**. The resulting fragments from the 635.1 Da fragment confirm both the presence of the aldol and oxime modifications, and that both modifications have occurred site-selectively at the *N*-terminus.

10. Kinetic data for OPAL

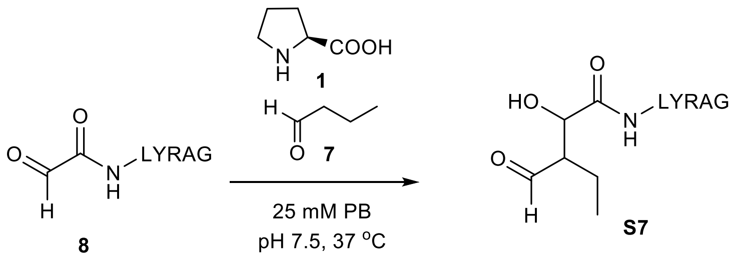

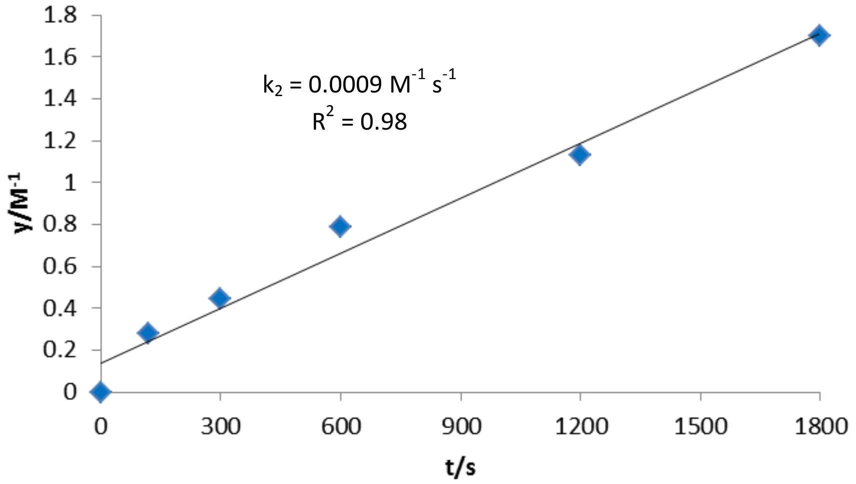

Supplementary Figure 30: Kinetic data for OPAL of glyoxyl-LYRAG **8** (0.5 mM) with donor **7** (100 mM) using 1 mM of catalyst **1**.

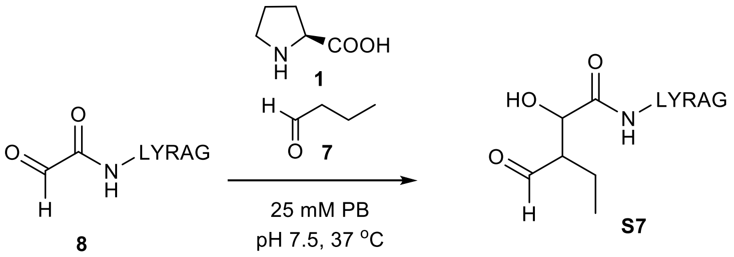

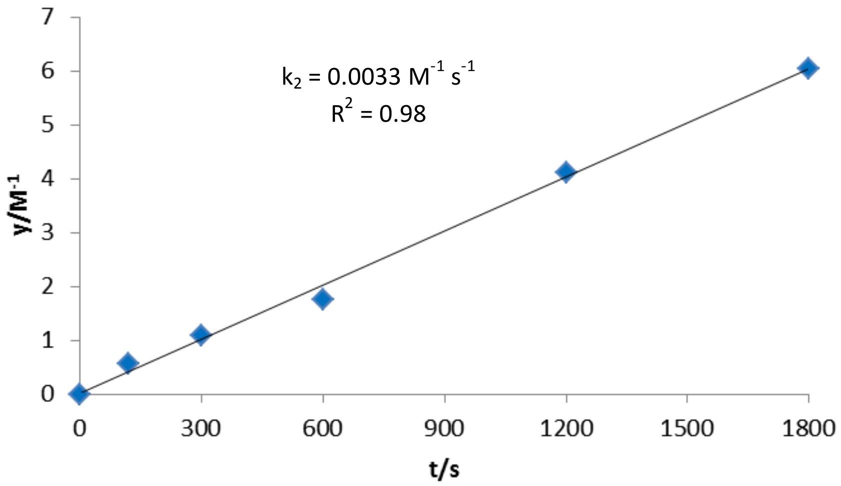

Supplementary Figure 31: Kinetic data for OPAL of glyoxyl-LYRAG **8** (0.5 mM) with donor **7** (100 mM) using 10 mM of catalyst **1**.

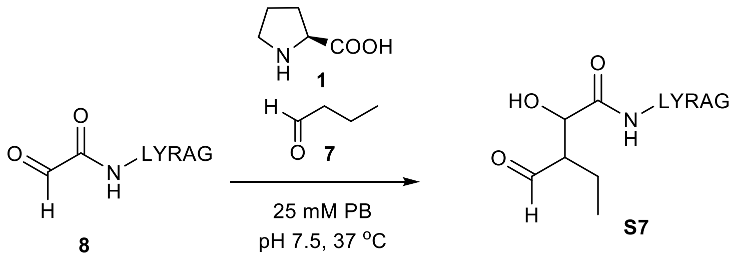

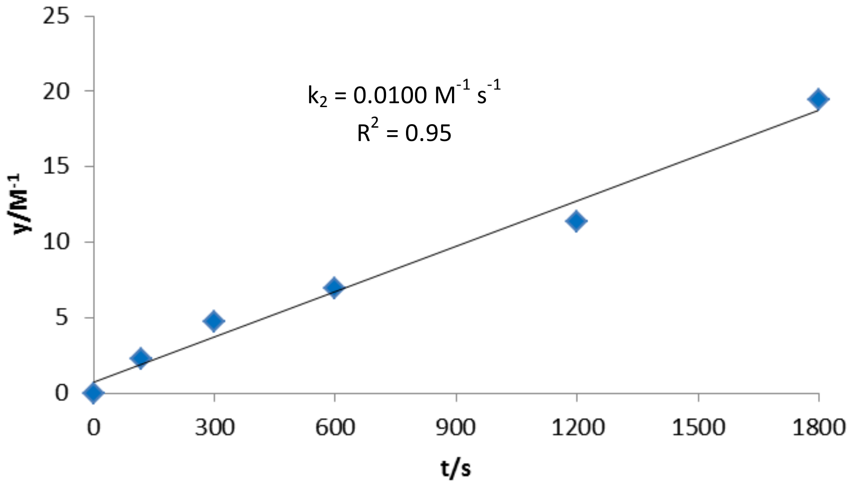

Supplementary Figure 32: Kinetic data for OPAL of glyoxyl-LYRAG **8** (0.5 mM) with donor **7** (100 mM) using 25 mM of catalyst **1**.

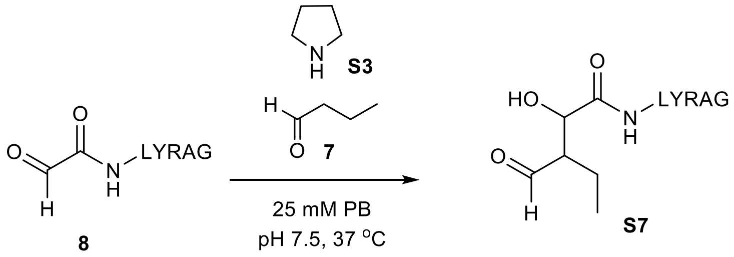

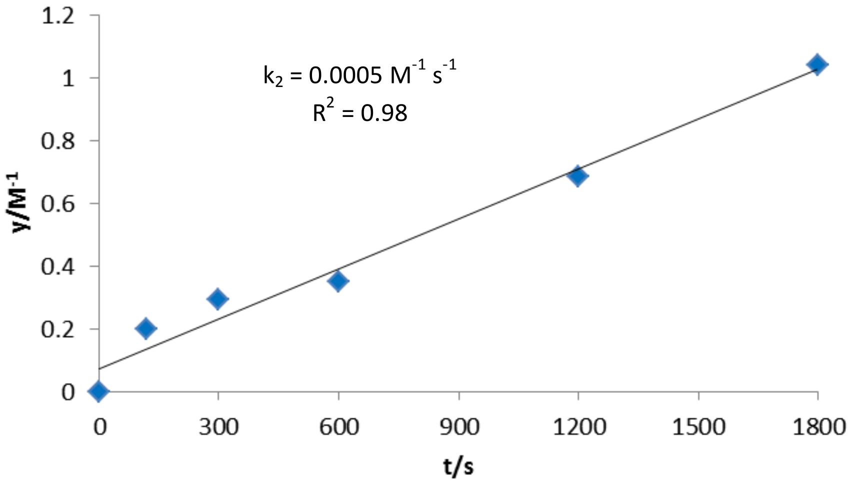

Supplementary Figure 33: Kinetic data for OPAL of glyoxyl-LYRAG **8** (0.5 mM) with donor **7** (100 mM) using 1 mM of catalyst **S3**.

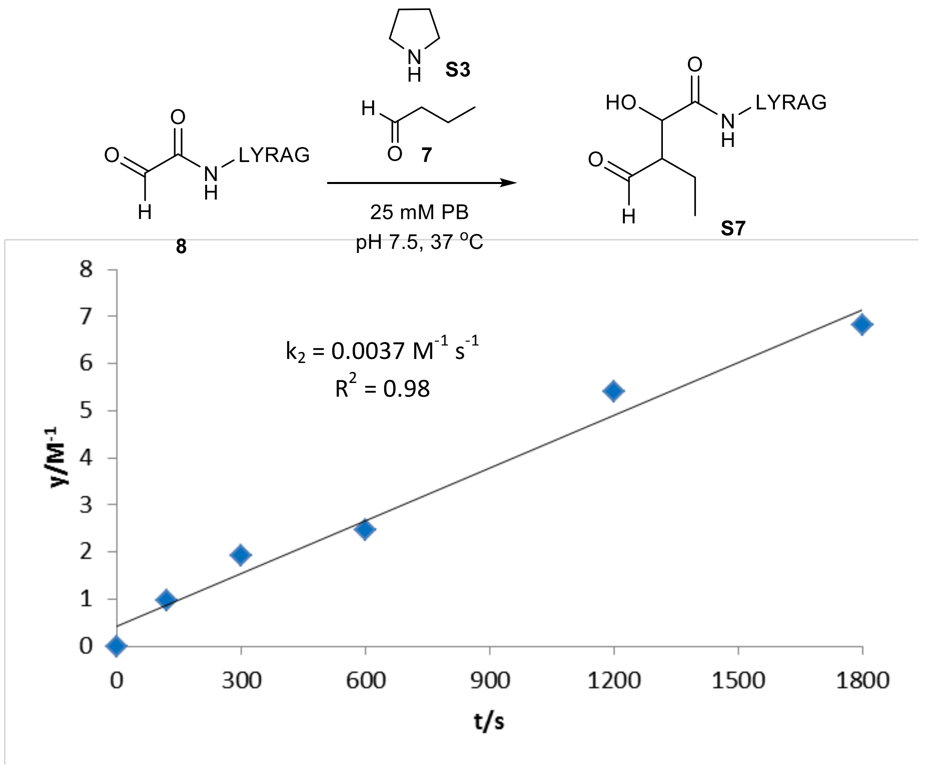

Supplementary Figure 34: Kinetic data for OPAL of glyoxyl-LYRAG **8** (0.5 mM) with donor **7** (100 mM) using 10 mM of catalyst **S3**.

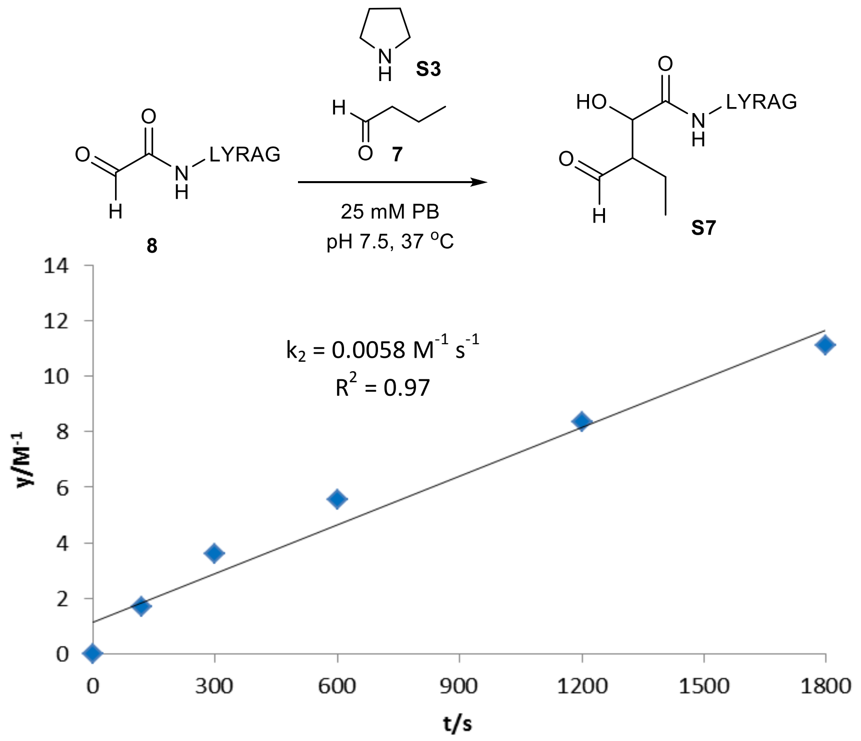

Supplementary Figure 35: Kinetic data for OPAL of glyoxyl-LYRAG **8** (0.5 mM) with donor **7** (100 mM) using 25 mM of catalyst **S3**.

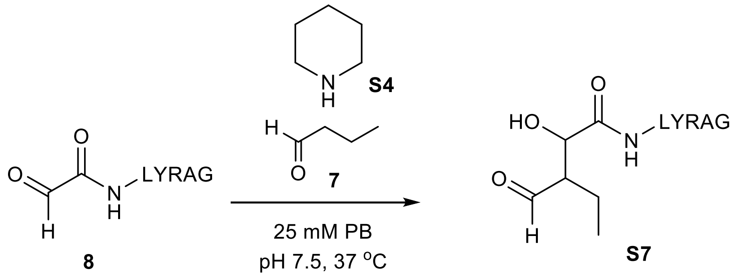

Experimentally determined k_2 is extremely low (>0.0001 M^{-1} s^{-1})

Supplementary Figure 36: Kinetic data for OPAL of glyoxyl-LYRAG **8** (0.5 mM) with donor **7** (100 mM) using 1 mM of catalyst **S4**

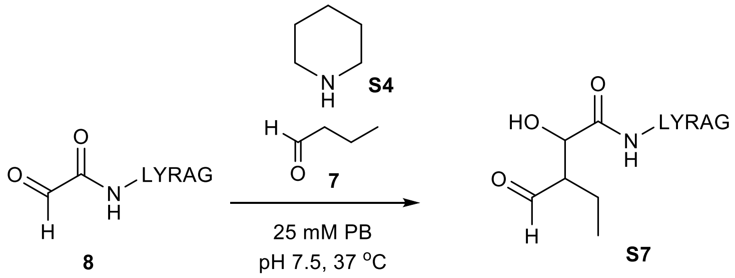

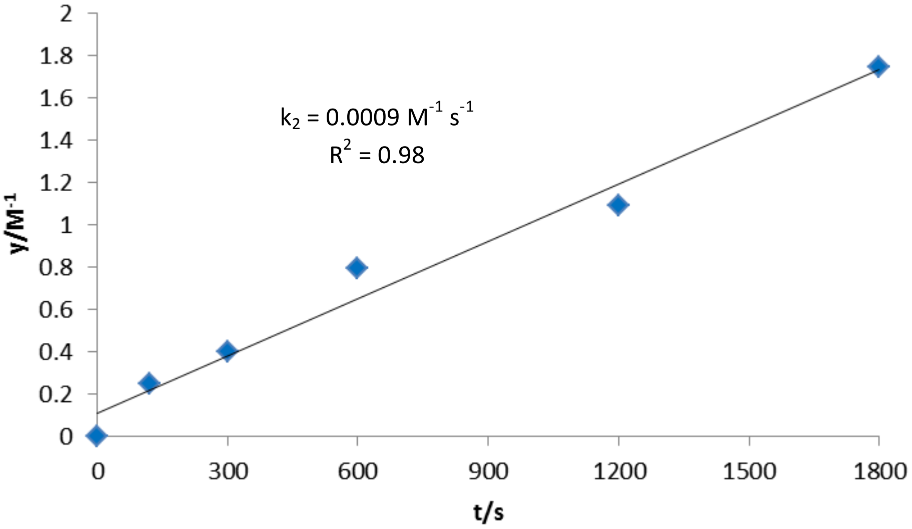

Supplementary Figure 37: Kinetic data for OPAL of glyoxyl-LYRAG **8** (0.5 mM) with donor **7** (100 mM) using 10 mM of catalyst **S4**.

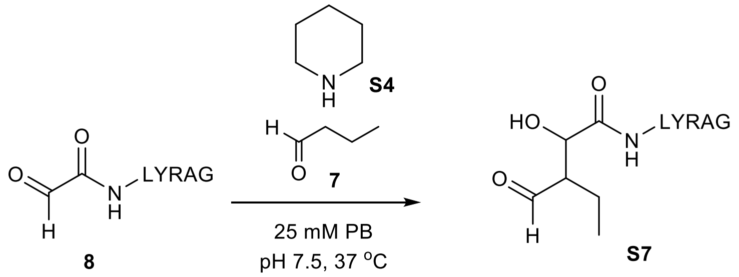

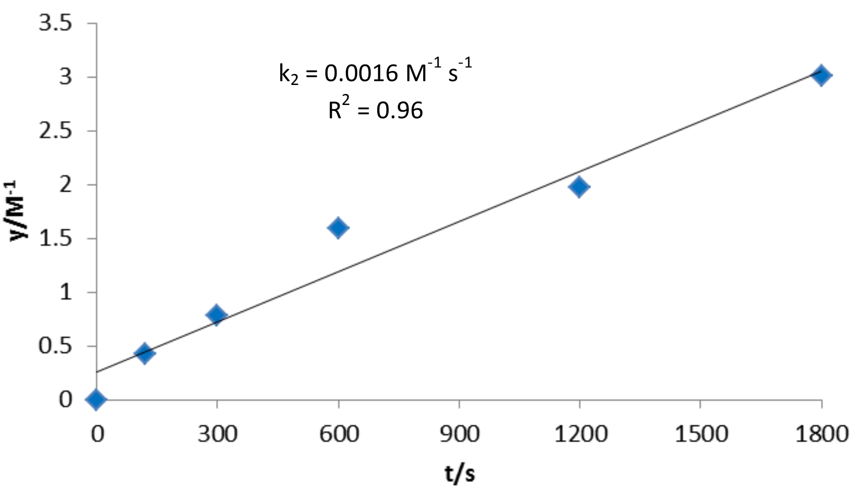

Supplementary Figure 38: Kinetic data for OPAL of glyoxyl-LYRAG **8** (0.5 mM) with donor **7** (100 mM) using 25 mM of catalyst **S4**.

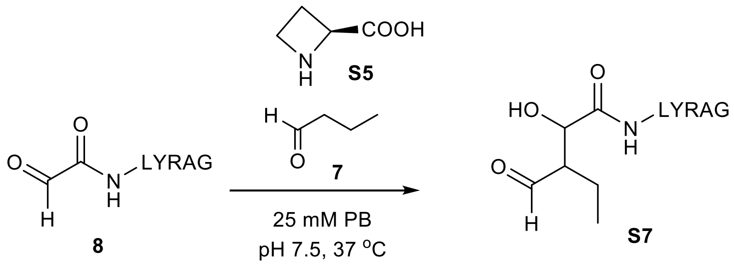

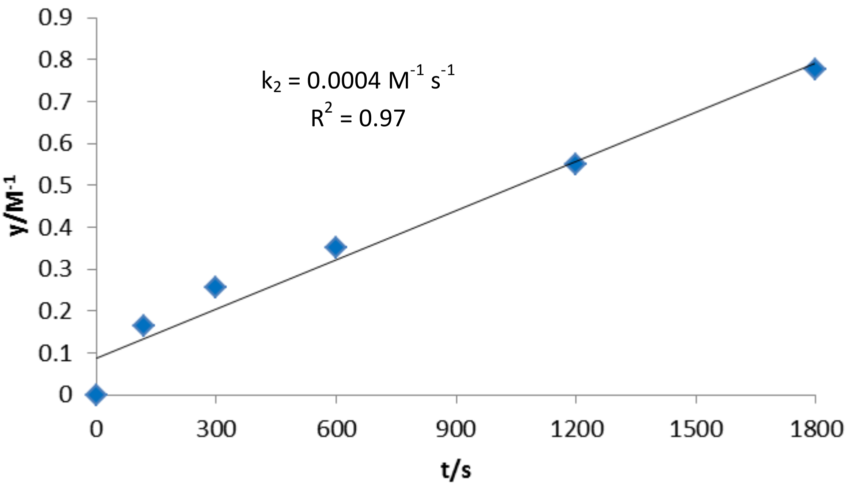

Supplementary Figure 39: Kinetic data for OPAL of glyoxyl-LYRAG **8** (0.5 mM) with donor **7** (100 mM) using 1 mM of catalyst **S5**.

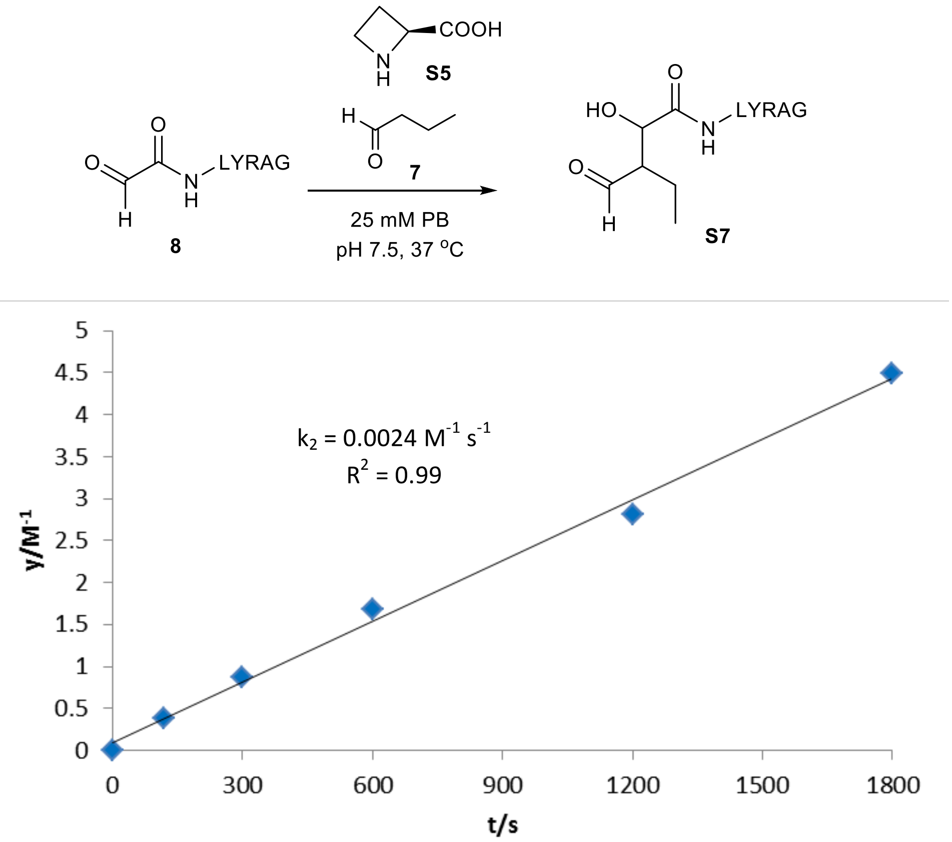

Supplementary Figure 40: Kinetic data for OPAL of glyoxyl-LYRAG **8** (0.5 mM) with donor **7** (100 mM) using 10 mM of catalyst **S5**.

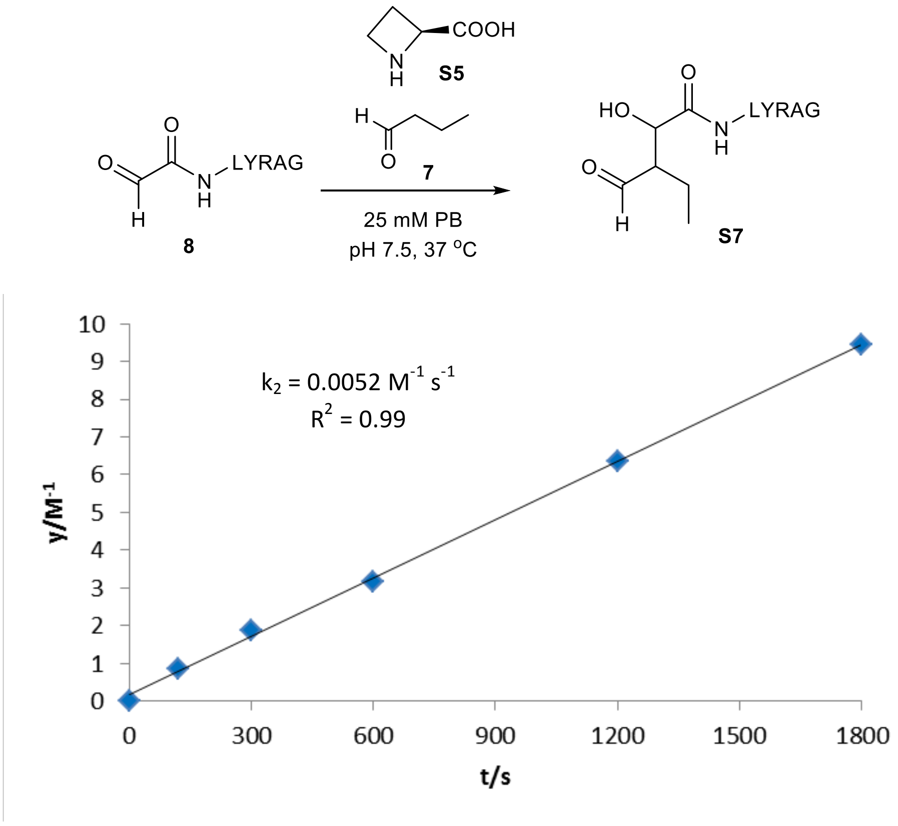

Supplementary Figure 41: Kinetic data for OPAL of glyoxyl-LYRAG **8** (0.5 mM) with donor **7** (100 mM) using 25 mM of catalyst **S5**.

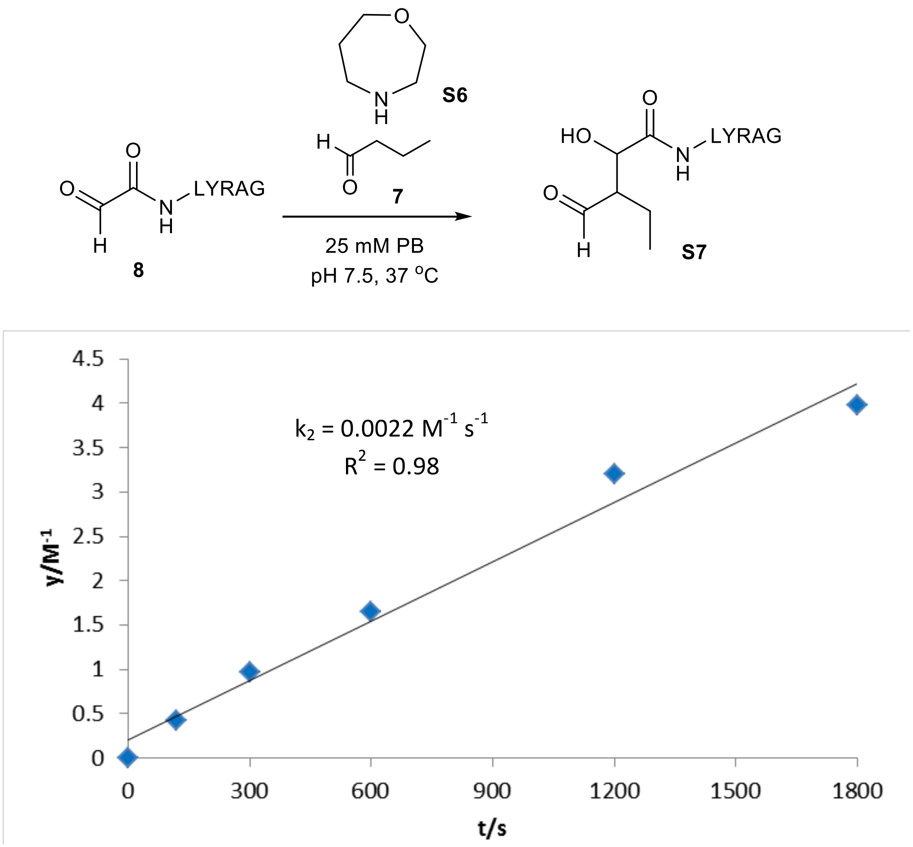

Supplementary Figure 42: Kinetic data for OPAL of glyoxyl-LYRAG **8** (0.5 mM) with donor **7** (100 mM) using 1 mM of catalyst **S6**.

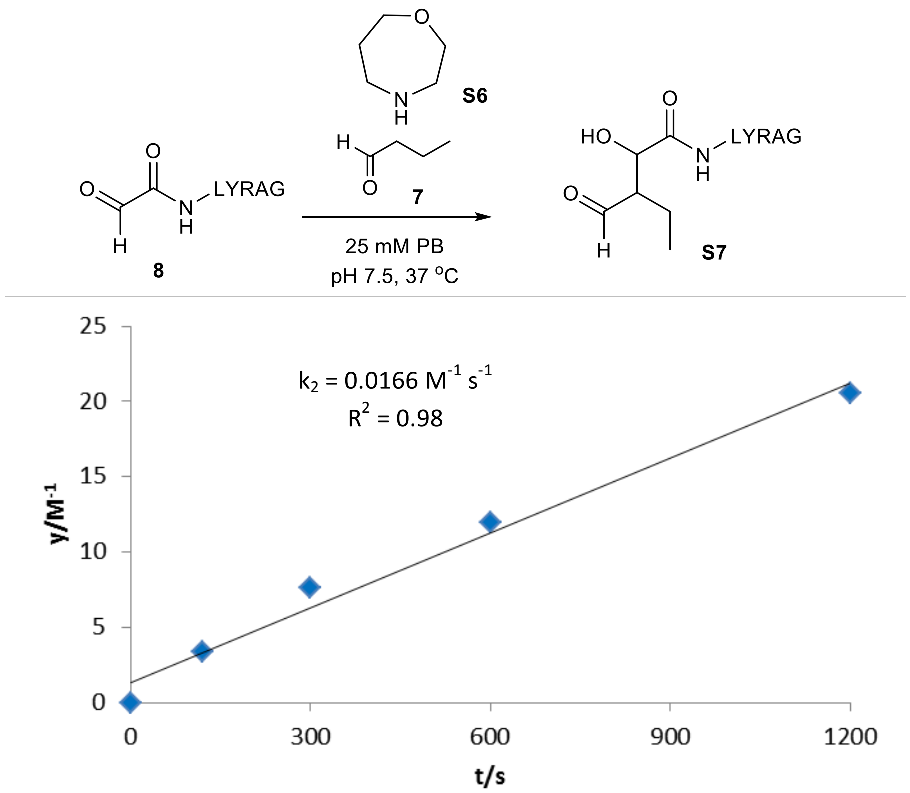

Supplementary Figure 43: Kinetic data for OPAL of glyoxyl-LYRAG **8** (0.5 mM) with donor **7** (100 mM) using 10 mM of catalyst **S6**.

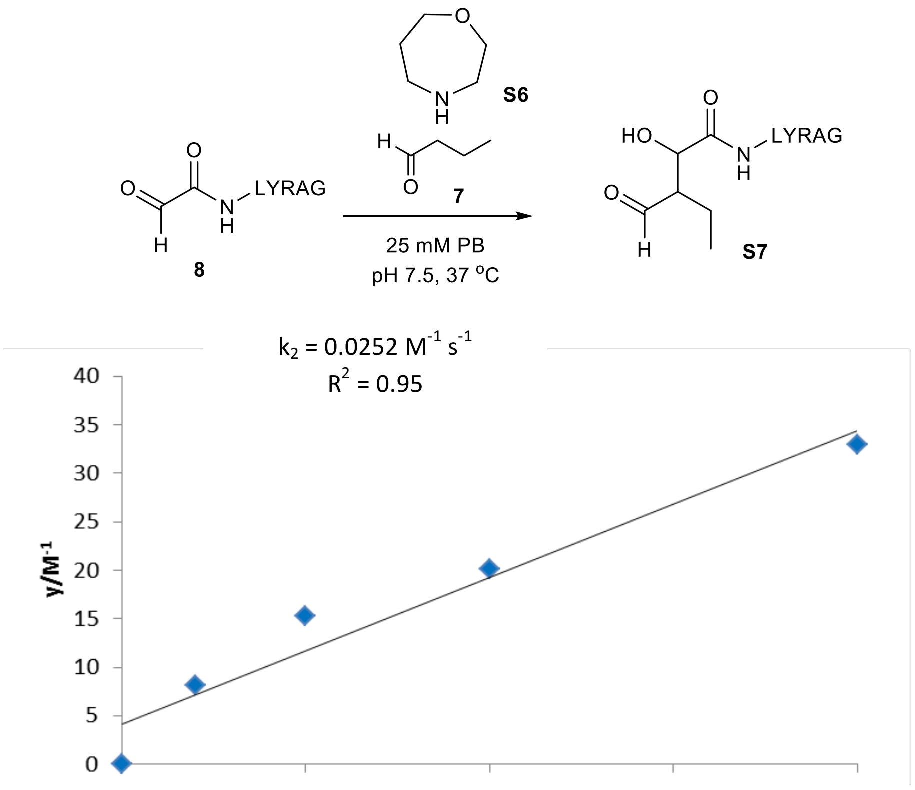

Supplementary Figure 44: Kinetic data for OPAL of glyoxyl-LYRAG **8** (0.5 mM) with donor **7** (100 mM) using 25 mM of catalyst **S6**.

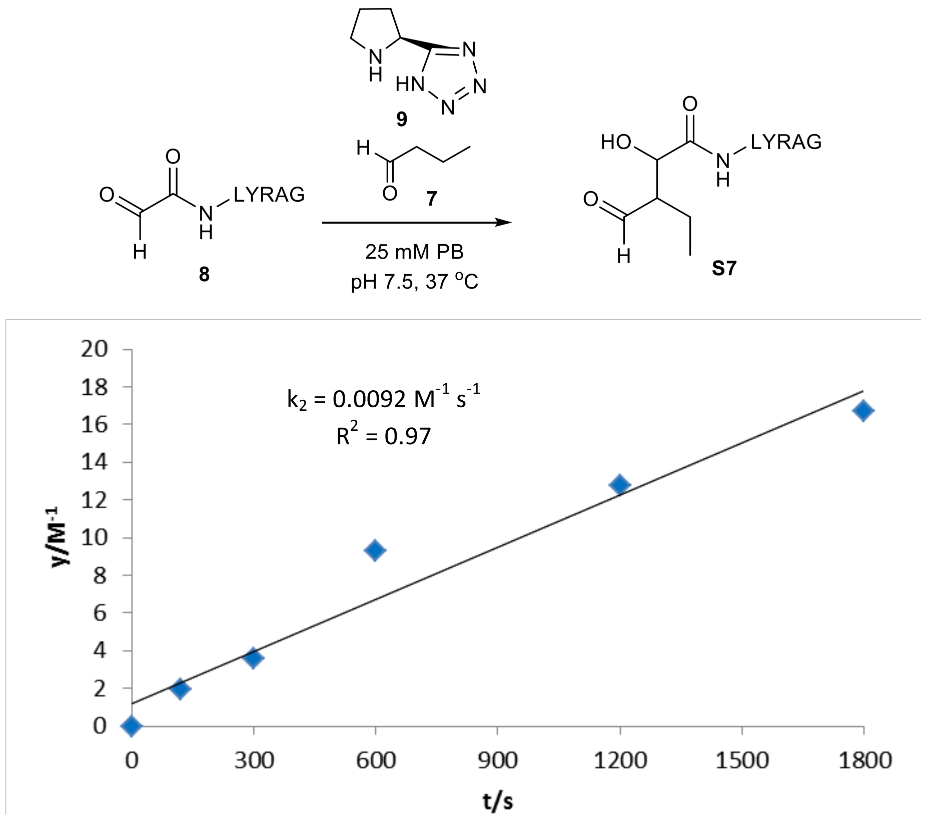

Supplementary Figure 45: Kinetic data for OPAL of glyoxyl-LYRAG **8** (0.5 mM) with donor **7** (10 mM) using 1 mM of catalyst **9**.

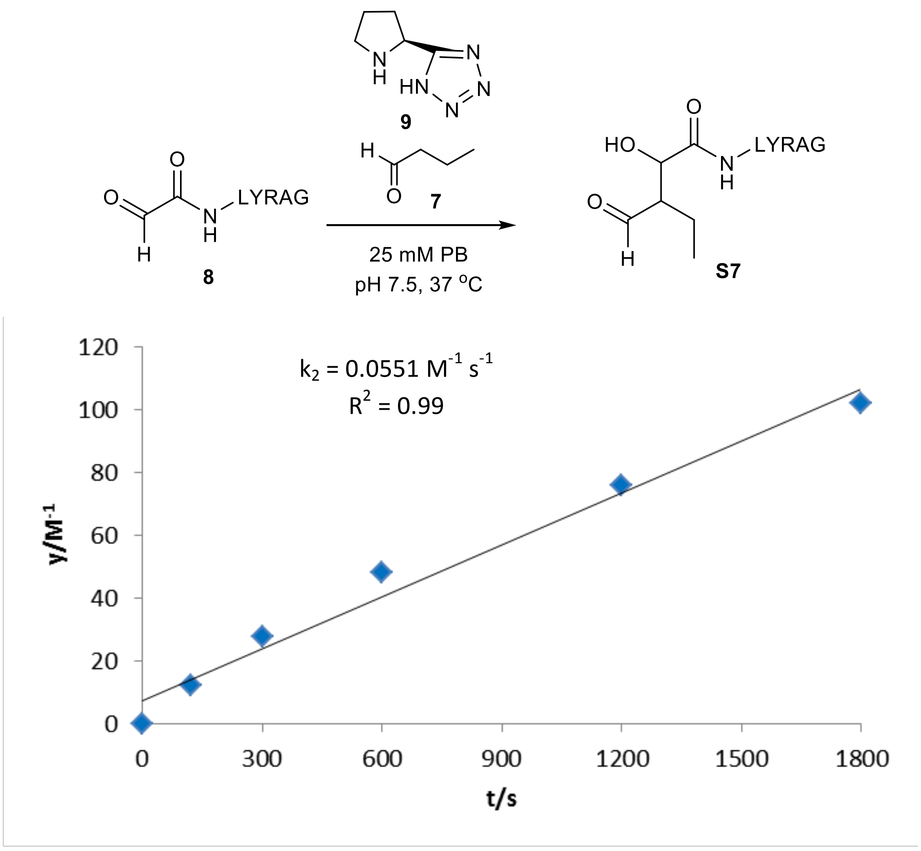

Supplementary Figure 46: Kinetic data for OPAL of glyoxyl-LYRAG **8** (0.5 mM) with donor **7** (10 mM) using 10 mM of catalyst **9**.

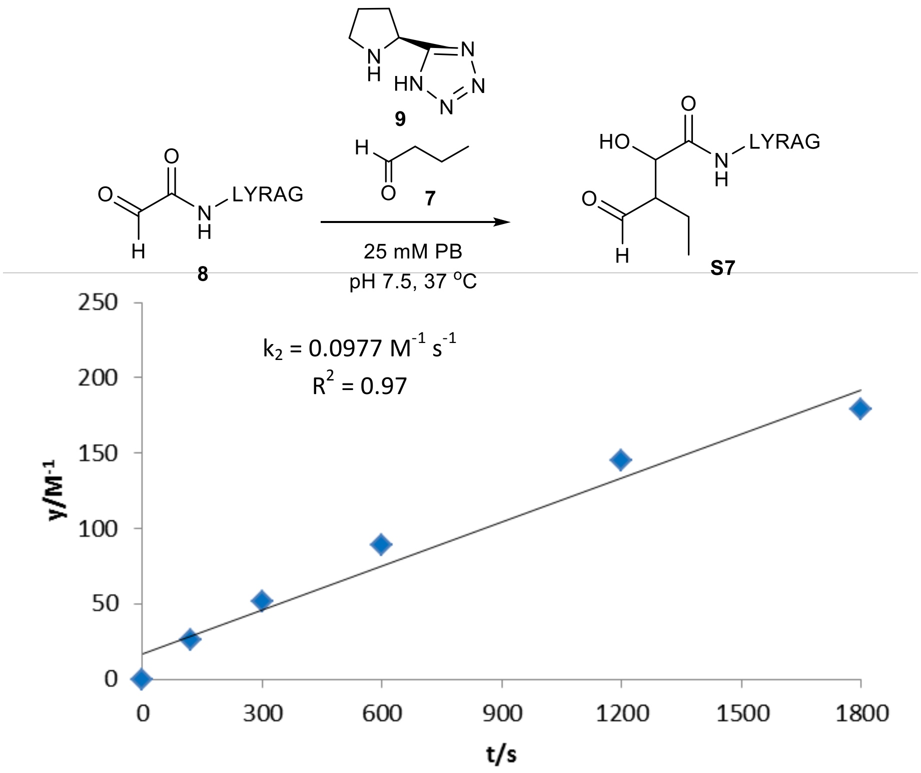

Supplementary Figure 47: Kinetic data for OPAL of glyoxyl-LYRAG **8** (0.5 mM) with donor **7** (10 mM) using 25 mM of catalyst **9**.

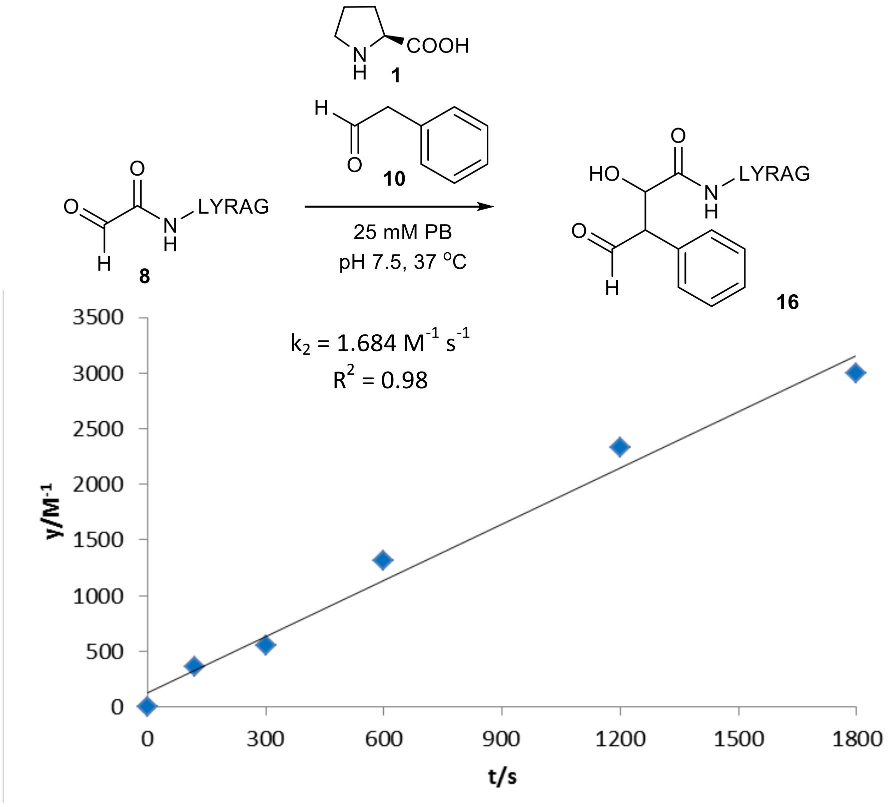

Supplementary Figure 48: Kinetic data for OPAL of glyoxyl-LYRAG **8** (50 µM) with donor **10** (150 µM) using 1 mM of catalyst **1**.

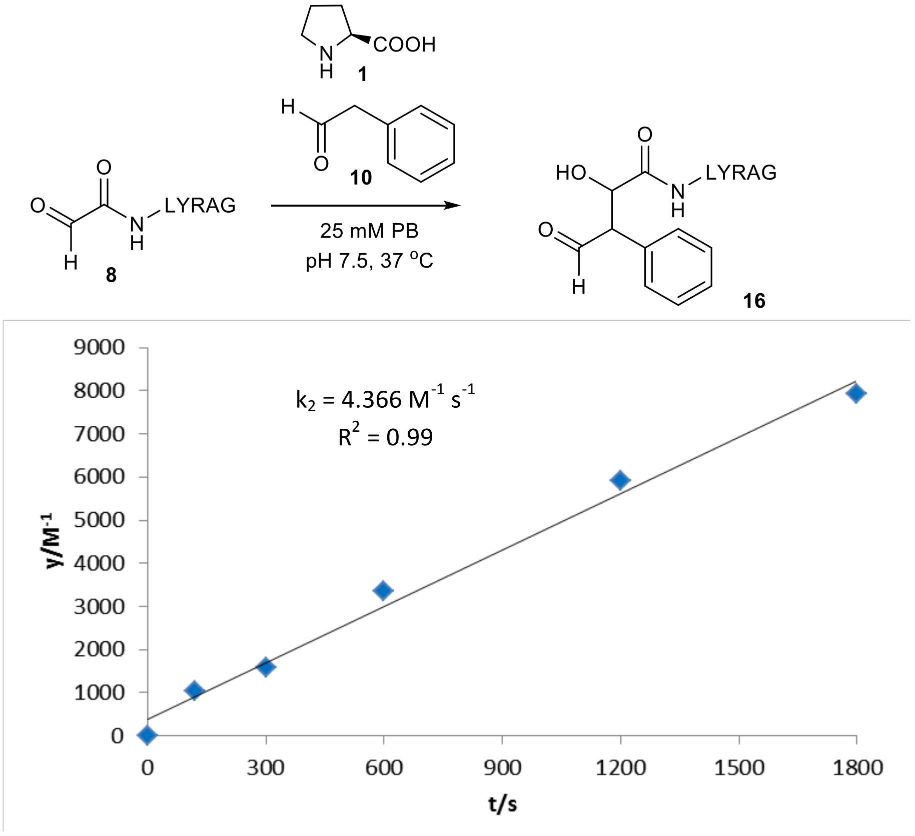

Supplementary Figure 49: Kinetic data for OPAL of glyoxyl-LYRAG **8** (50 µM) with donor **10** (150 µM) using 10 mM of catalyst **1**.

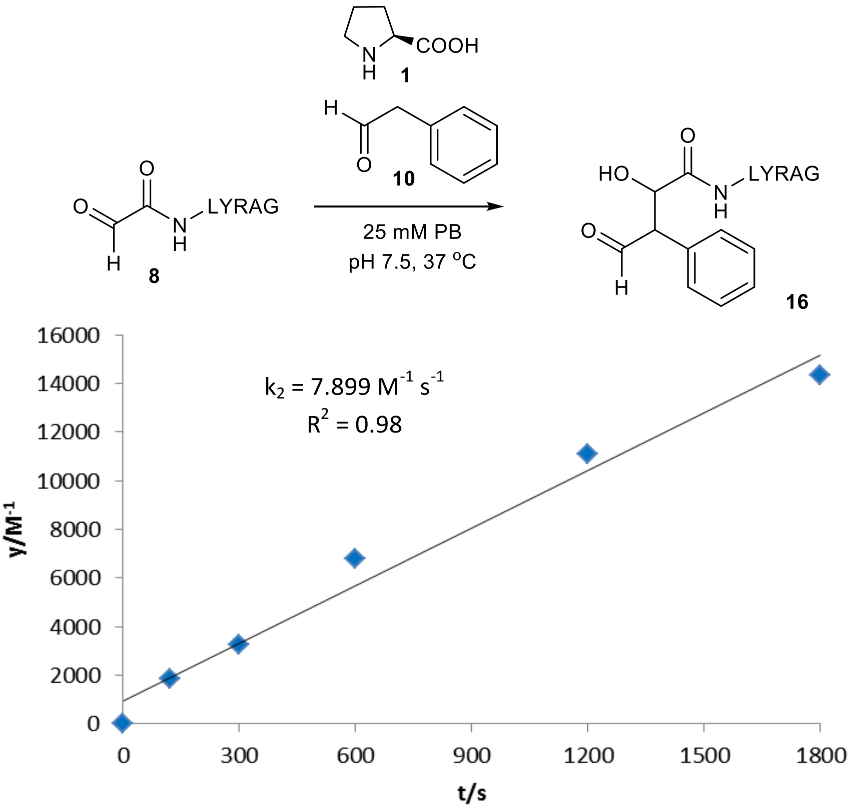

Supplementary Figure 50: Kinetic data for OPAL of glyoxyl-LYRAG **8** (50 µM) with donor **10** (150 µM) using 25 mM of catalyst **1**.

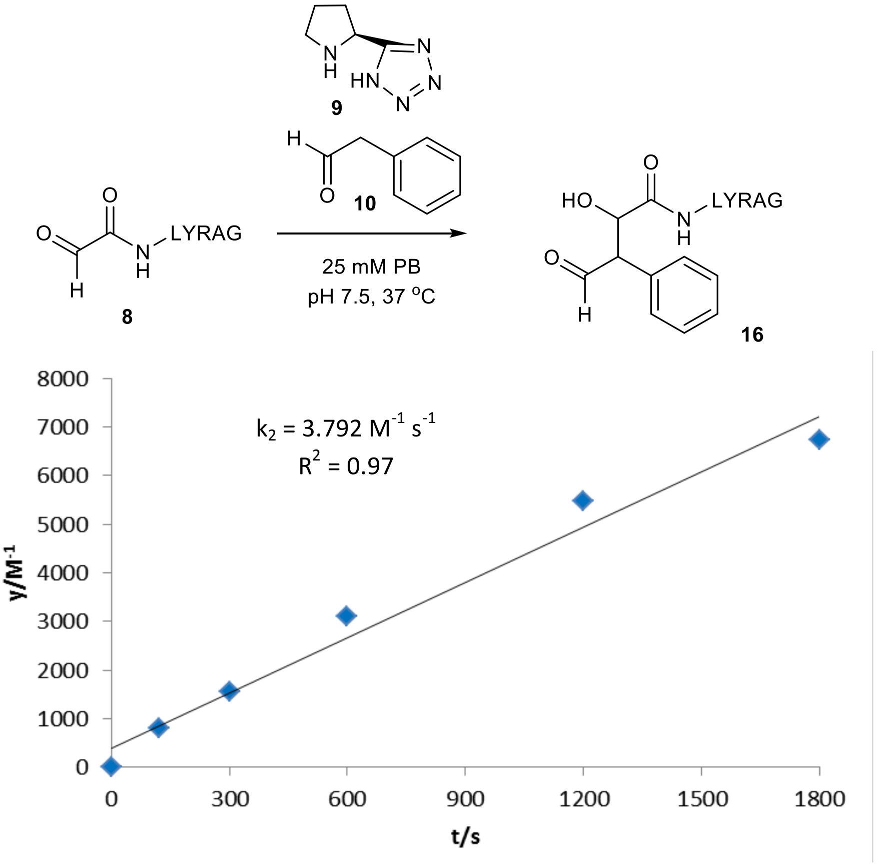

Supplementary Figure 51: Kinetic data for OPAL of glyoxyl-LYRAG **8** (50 µM) with donor **10** (150 µM) using 1 mM of catalyst **1**.

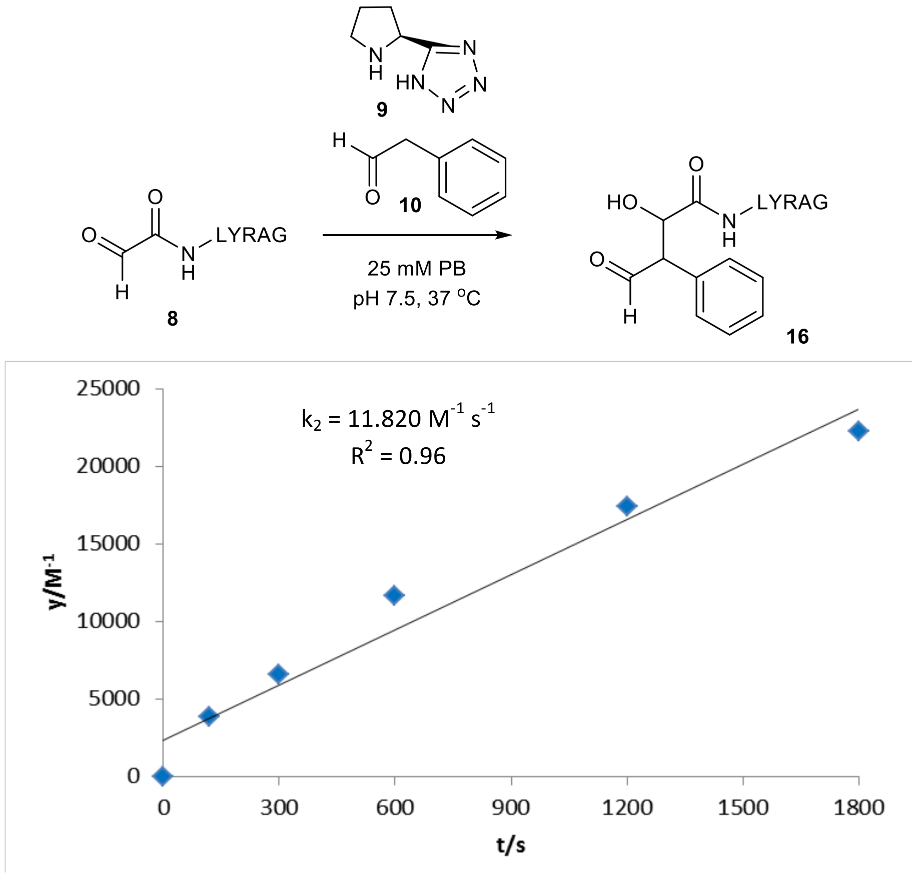

Supplementary Figure 52: Kinetic data for OPAL of glyoxyl-LYRAG **8** (50 µM) with donor **10** (150 µM) using 10 mM of catalyst **1**.

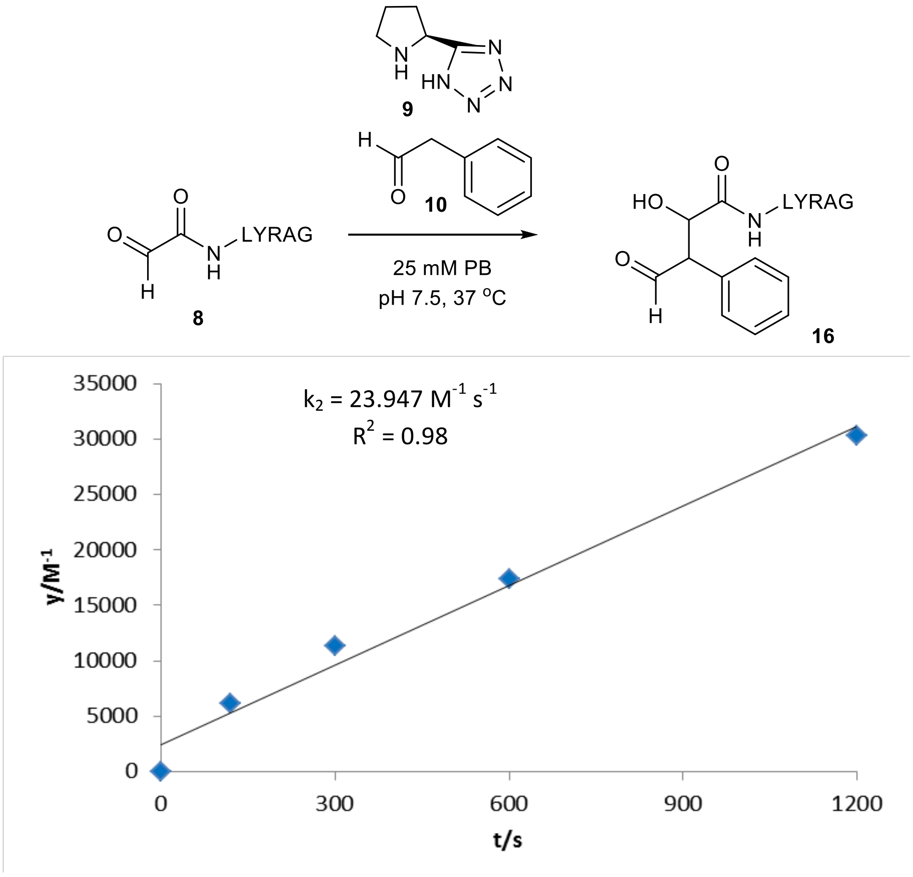

Supplementary Figure 53: Kinetic data for OPAL of glyoxyl-LYRAG **8** (50 µM) with donor **10** (150 µM) using 25 mM of catalyst **1**.

11. NMR Data

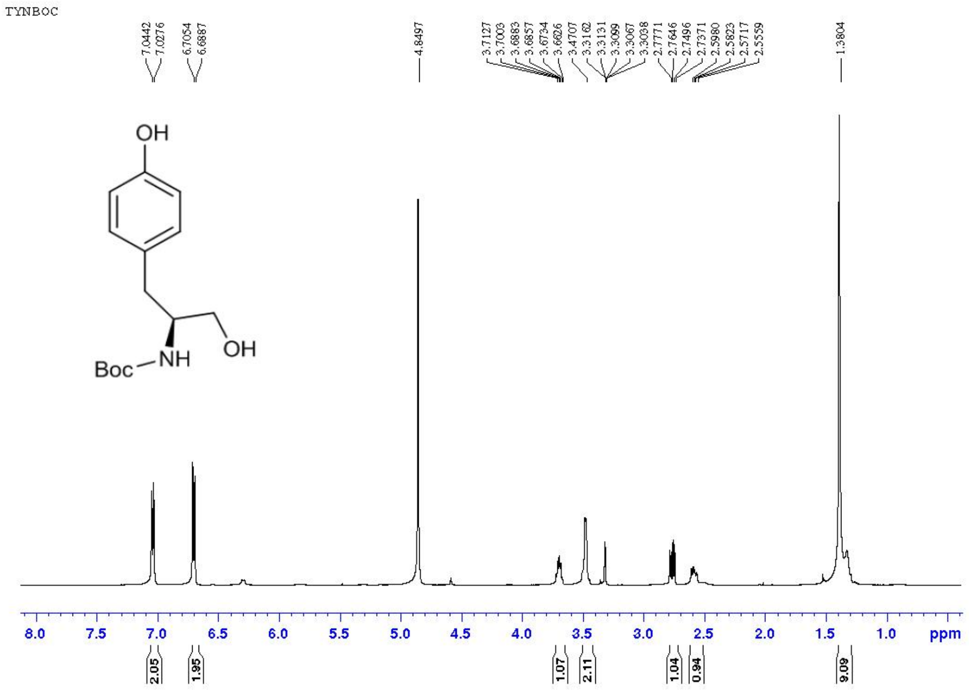
TYNBOC
7.0442
7.0276
6.7054
6.6887
4.8497
3.7127
3.7003
3.6883
3.6857
3.6734
3.6626
3.4707
3.3162
3.3131
3.3099
3.3067
3.3038
2.7771
2.7646
2.7496
2.7371
2.5980
2.5823
2.5717
2.5559
1.3804
OH
OH
Boc
NH
8.0 7.5 7.0 6.5 6.0 5.5 5.0 4.5 4.0 3.5 3.0 2.5 2.0 1.5 1.0 ppm
2.05
1.95
1.07
2.11
1.04
0.94
9.09

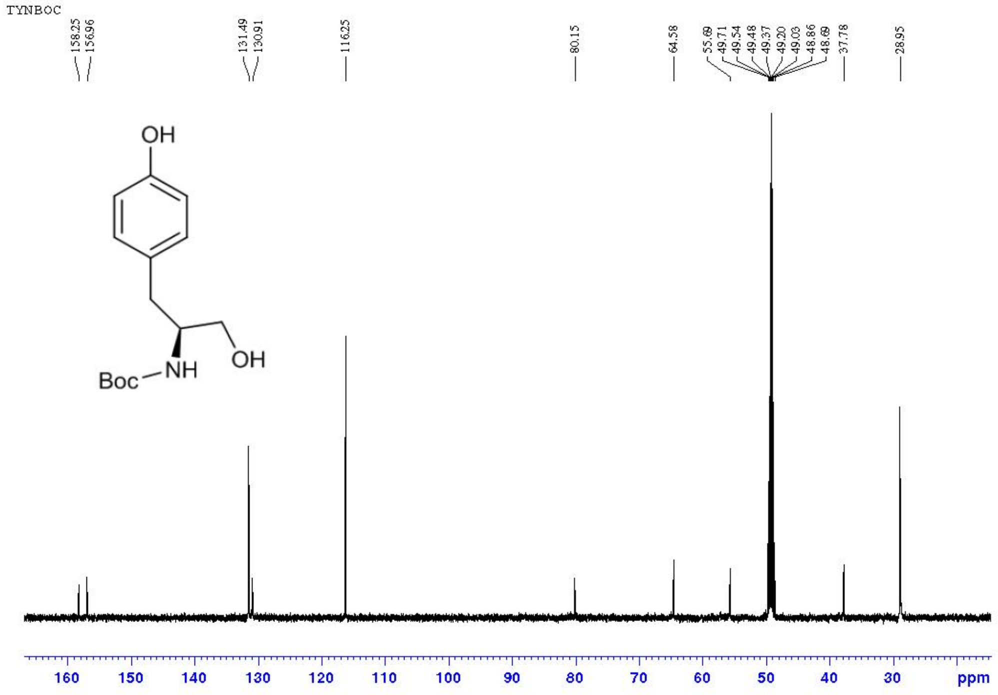
TYNBOC
158.25
156.96
131.49
130.91
116.25
80.15
64.58
55.69
49.71
49.54
49.48
49.37
49.20
49.03
48.86
48.69
37.78
28.95
OH
OH
Boc
NH
160 150 140 130 120 110 100 90 80 70 60 50 40 30 ppm

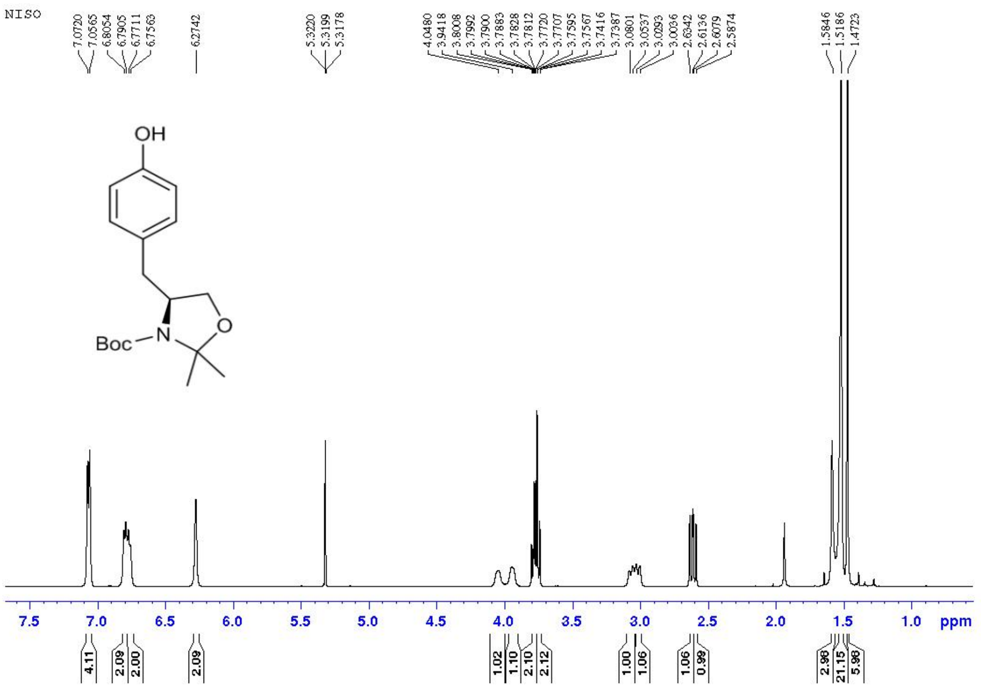

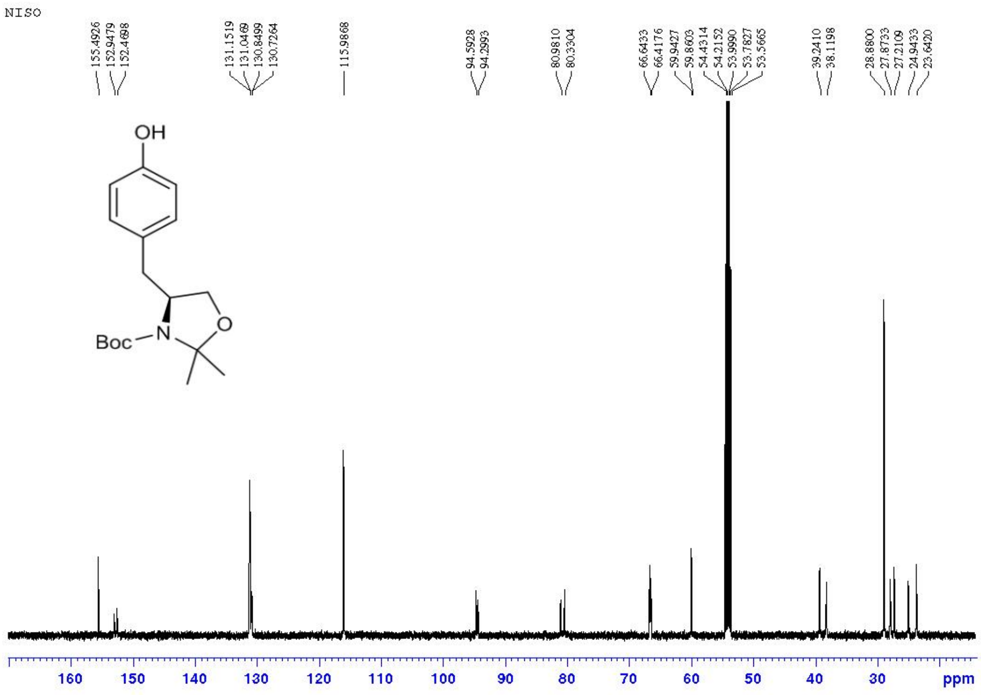

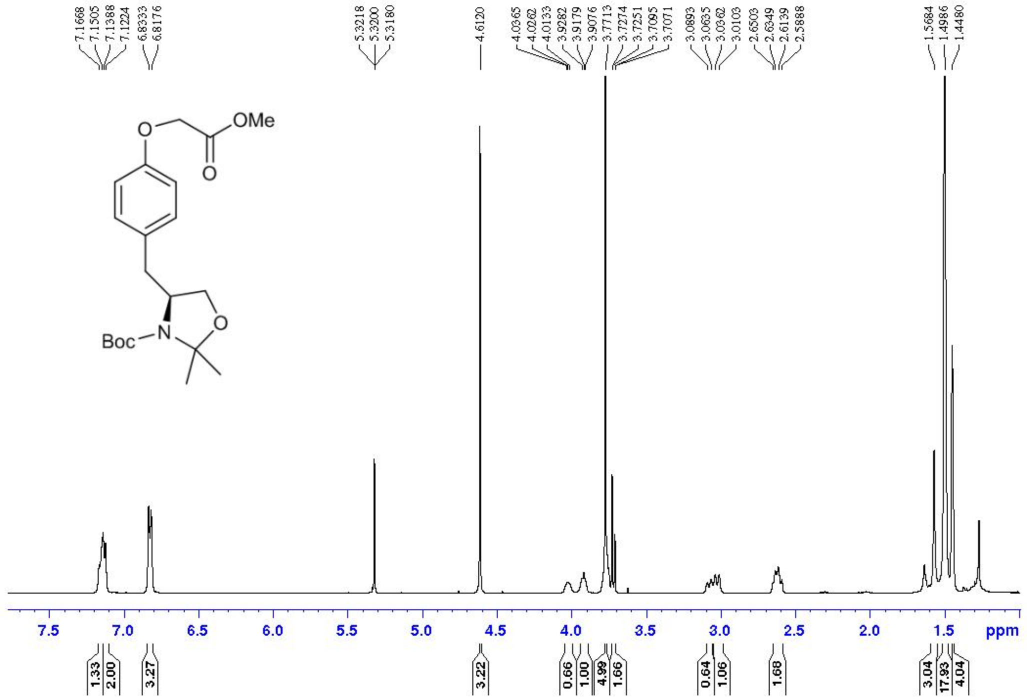
7.1668
7.1505
7.1388
7.1224
6.8333
6.8176
5.3218
5.3200
5.3180
4.6120
4.0365
4.0262
4.0133
3.9282
3.9179
3.9076
3.7713
3.7274
3.7251
3.7095
3.7071
3.0893
3.0635
3.0362
3.0103
2.6503
2.6349
2.6139
2.5888
1.5684
1.4986
1.4480
OMe
O
O
O
Boc
N
O
ppm
1.33
2.00
3.27
3.22
0.66
1.00
4.99
1.66
0.64
1.06
1.68
3.04
17.93
4.04

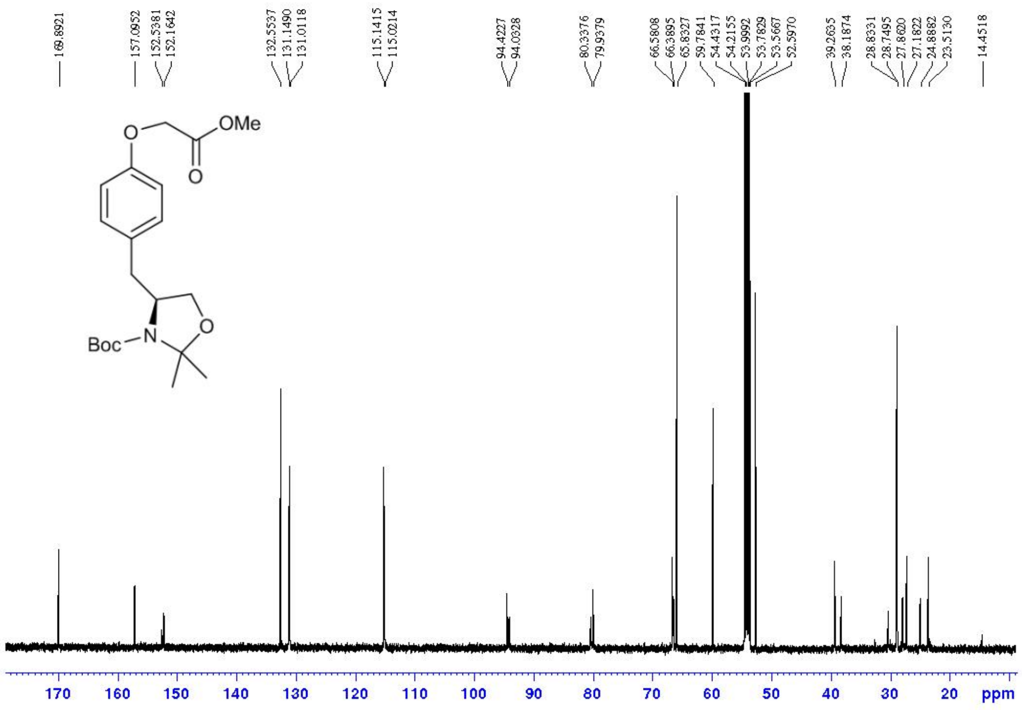
169.8921
157.0952
152.5381
152.1642
132.5537
131.1490
131.0118
115.1415
115.0214
94.4227
94.0328
80.3376
79.9379
66.5808
66.3895
65.8327
59.7841
54.4317
54.2155
53.9992
53.7829
53.5667
52.5970
39.2635
38.1874
28.8331
28.7495
27.8620
27.1822
24.8882
23.5130
14.4518
OMe
O
O
O
Boc
N
O
ppm

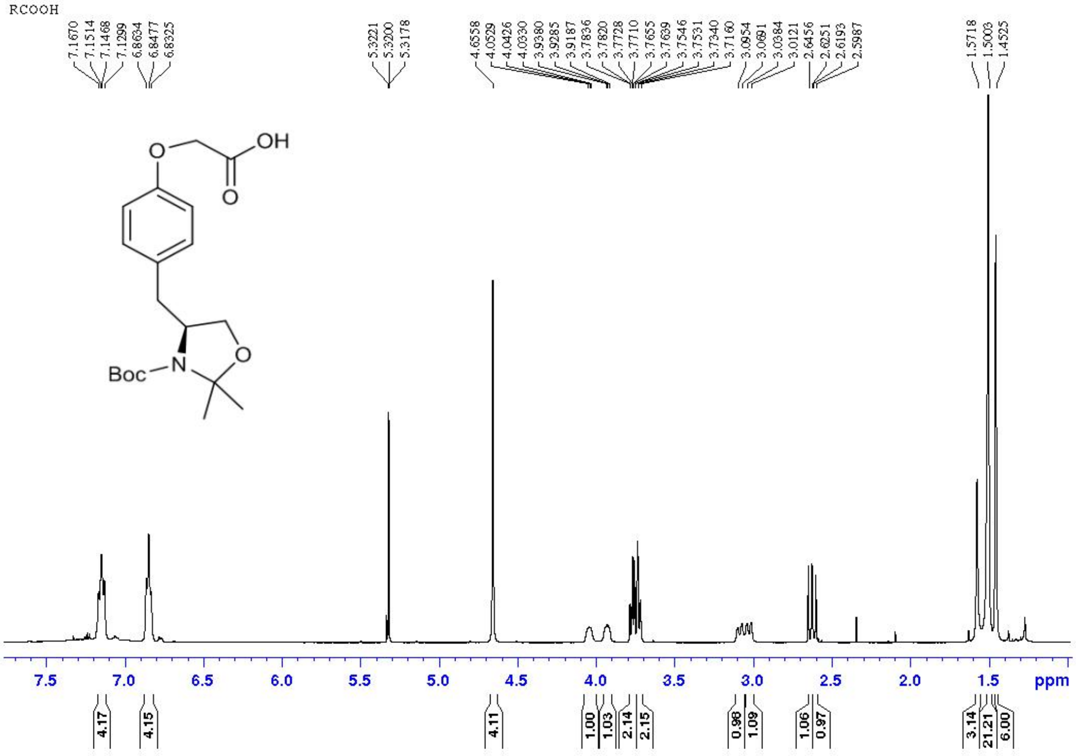
RCOOH
7.1670
7.1514
7.1468
7.1299
6.8634
6.8477
6.8325
5.3221
5.3200
5.3178
4.6558
4.0529
4.0426
4.0330
3.9380
3.9285
3.9187
3.7836
3.7820
3.7728
3.7710
3.7655
3.7639
3.7546
3.7531
3.7340
3.7160
3.0954
3.0691
3.0384
3.0121
2.6456
2.6251
2.6193
2.5987
1.5718
1.5003
1.4525
OH
O
O
O
Boc
N
O
7.5
7.0
6.5
6.0
5.5
5.0
4.5
4.0
3.5
3.0
2.5
2.0
1.5
ppm
4.17
4.15
4.11
1.00
1.03
2.14
2.15
0.98
1.09
1.06
0.97
3.14
21.21
6.00

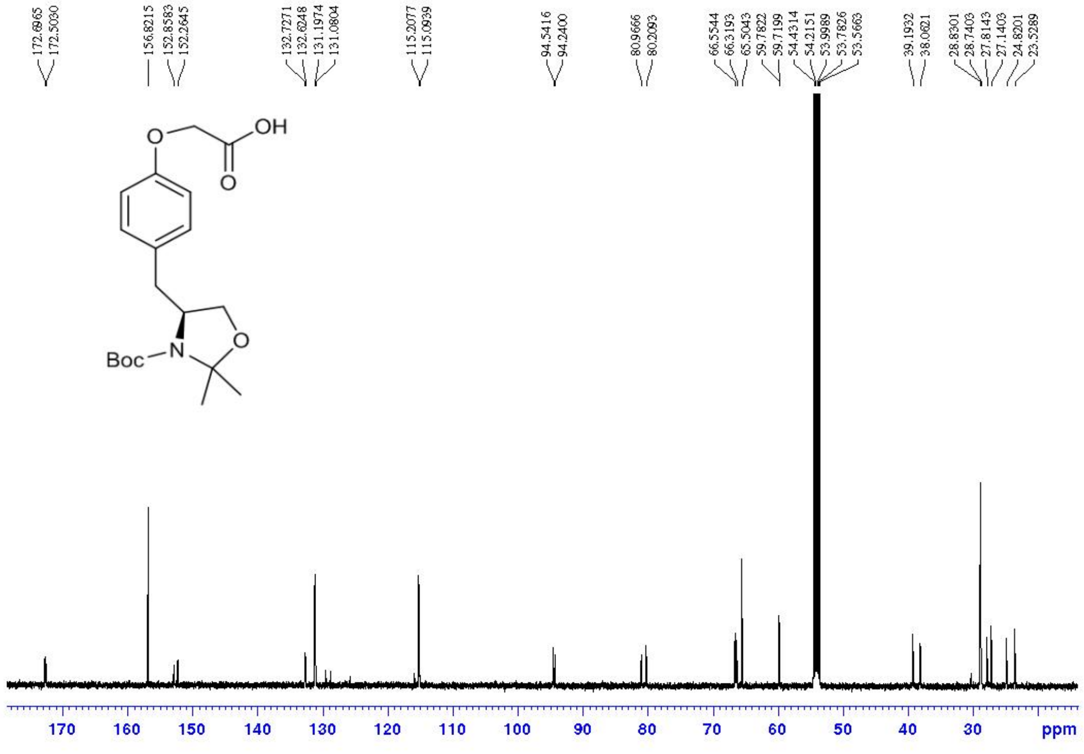
172.6965
172.5030
156.8215
152.8583
152.2645
132.7271
132.6248
131.1974
131.0804
115.2077
115.0939
94.5416
94.2400
80.9666
80.2093
66.5544
66.3193
65.5043
59.7822
59.7199
54.4314
54.2151
53.9989
53.7826
53.5663
39.1932
38.0621
28.8301
28.7403
27.8143
27.1403
24.8201
23.5289
OH
O
O
O
Boc
N
O
170
160
150
140
130
120
110
100
90
80
70
60
50
40
30
ppm

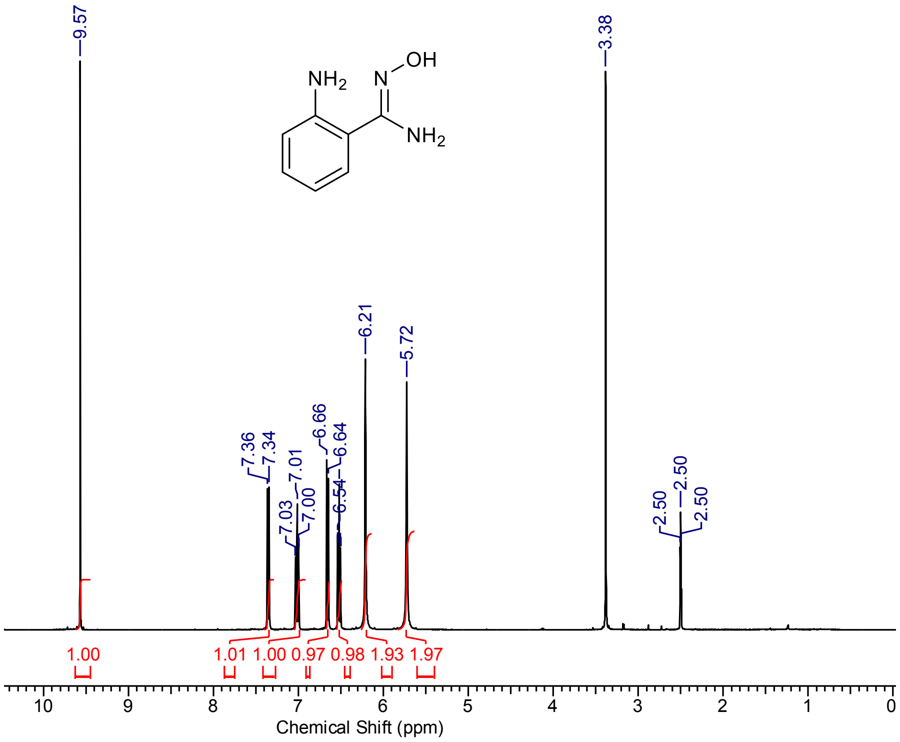

NH2
N
OH
NH2
9.57
7.36
7.34
7.03
7.01
7.00
6.66
6.64
6.54
6.21
5.72
3.38
2.50
2.50
2.50
1.00
1.01
1.00
0.97
0.98
1.93
1.97
Chemical Shift (ppm)

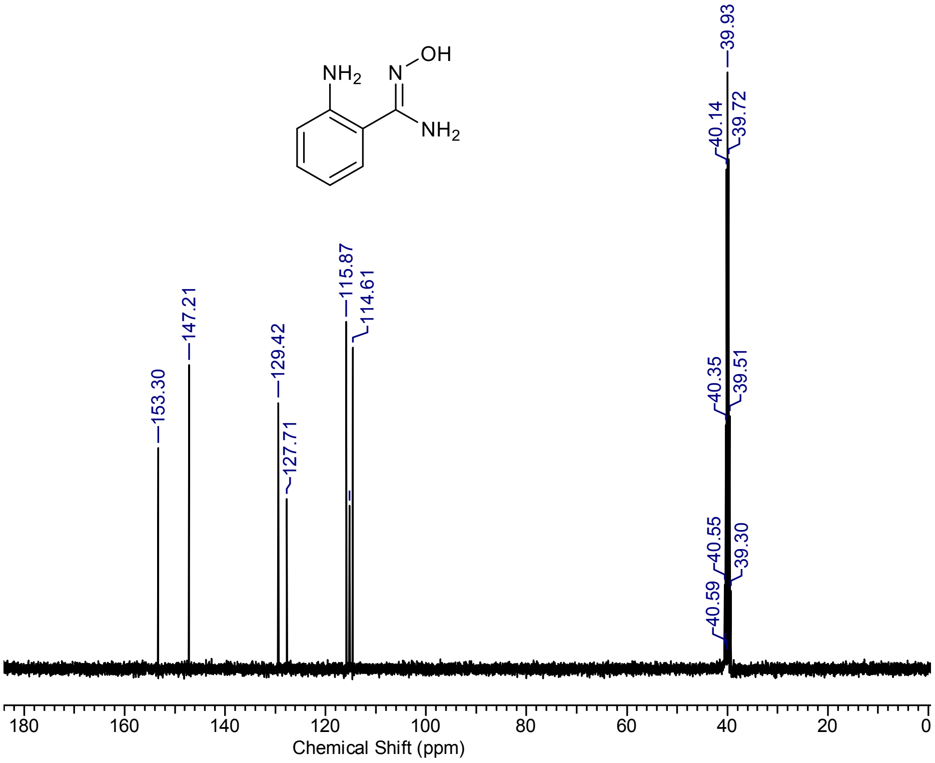

NH2
N
OH
NH2
153.30
147.21
129.42
127.71
115.87
114.61
40.59
40.55
40.35
40.14
39.93
39.72
39.51
39.30
Chemical Shift (ppm)

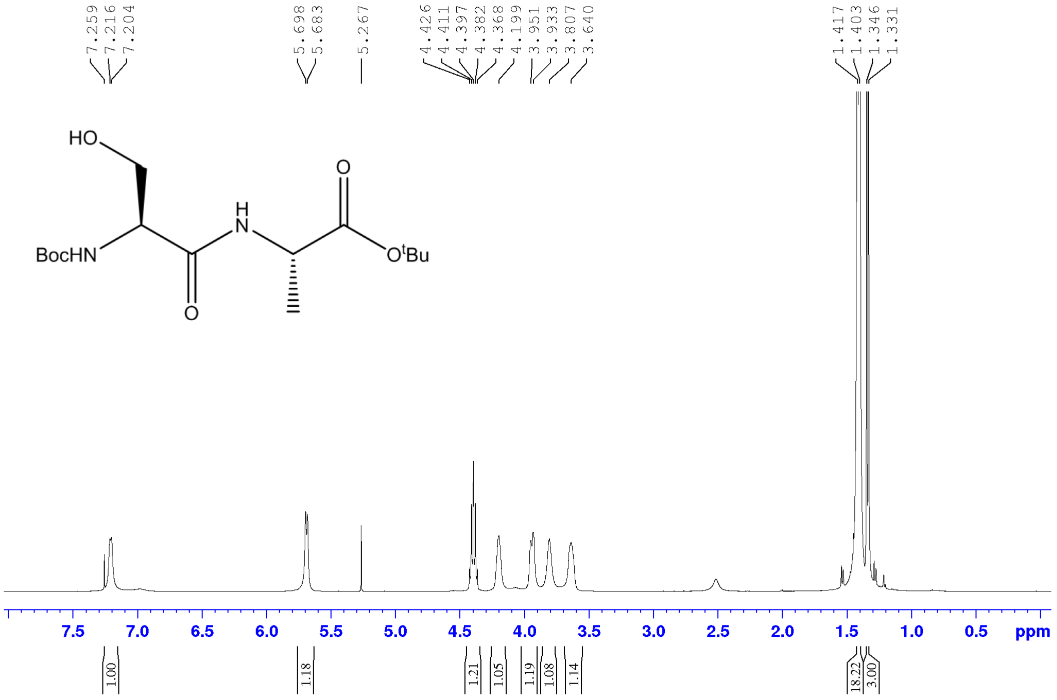
7.259
7.216
7.204
5.698
5.683
5.267
4.426
4.411
4.397
4.382
4.368
4.199
3.951
3.933
3.807
3.640
1.417
1.403
1.346
1.331
HO
O
H
N
BocHN
O
O^{t}Bu
1.00
1.18
1.21
1.05
1.19
1.08
1.14
18.22
3.00
ppm

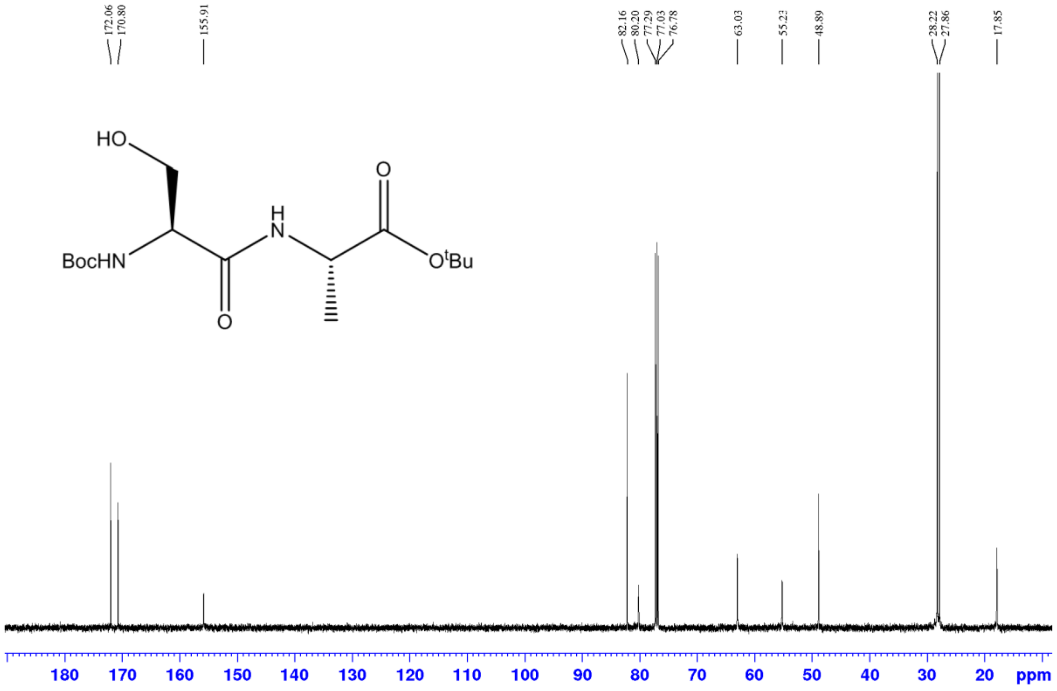
172.06
170.80
155.91
82.16
80.20
77.29
77.03
76.78
63.03
55.23
48.89
28.22
27.86
17.85
HO
O
H
N
BocHN
O
O^{t}Bu
ppm

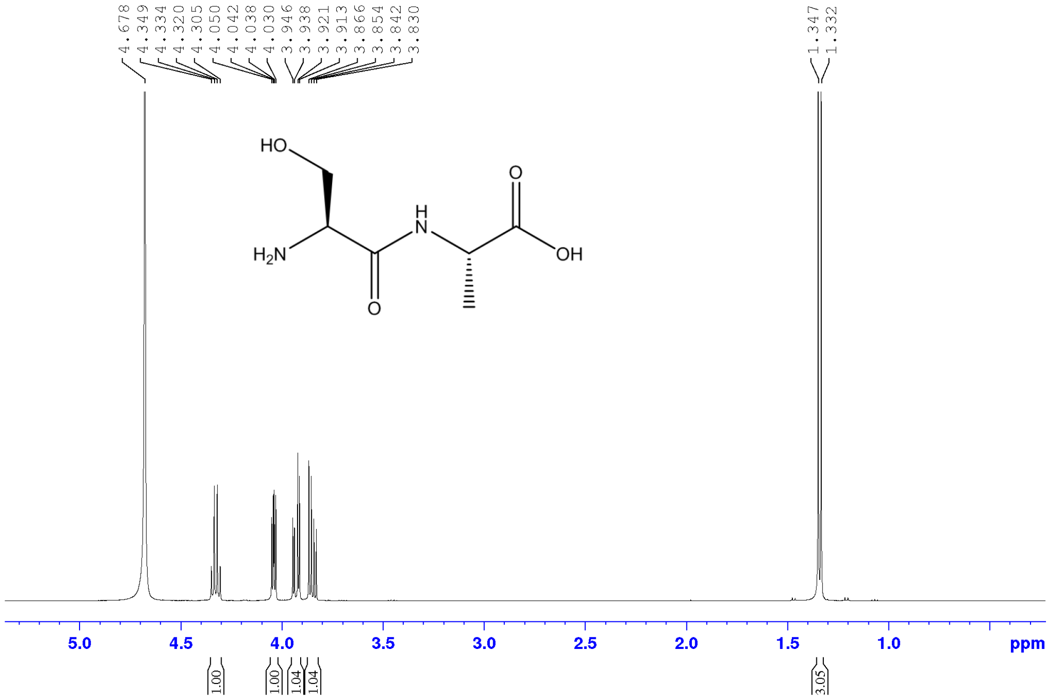
4.678
4.349
4.334
4.320
4.305
4.050
4.042
4.038
4.030
3.946
3.938
3.921
3.913
3.866
3.854
3.842
3.830
1.347
1.332
HO
O
H
N
H_2N
OH
O
5.0
4.5
4.0
3.5
3.0
2.5
2.0
1.5
1.0
ppm
1.00
1.00
1.04
1.04
3.05

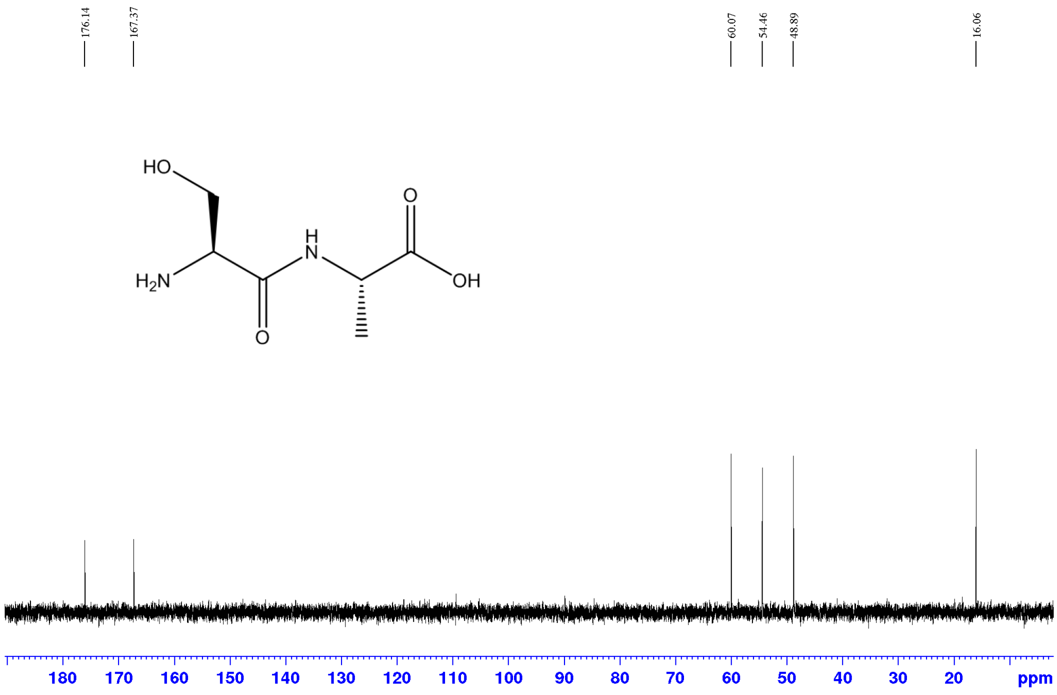
176.14
167.37
60.07
54.46
48.89
16.06
HO
O
H
N
H_2N
OH
O
180
170
160
150
140
130
120
110
100
90
80
70
60
50
40
30
20
ppm

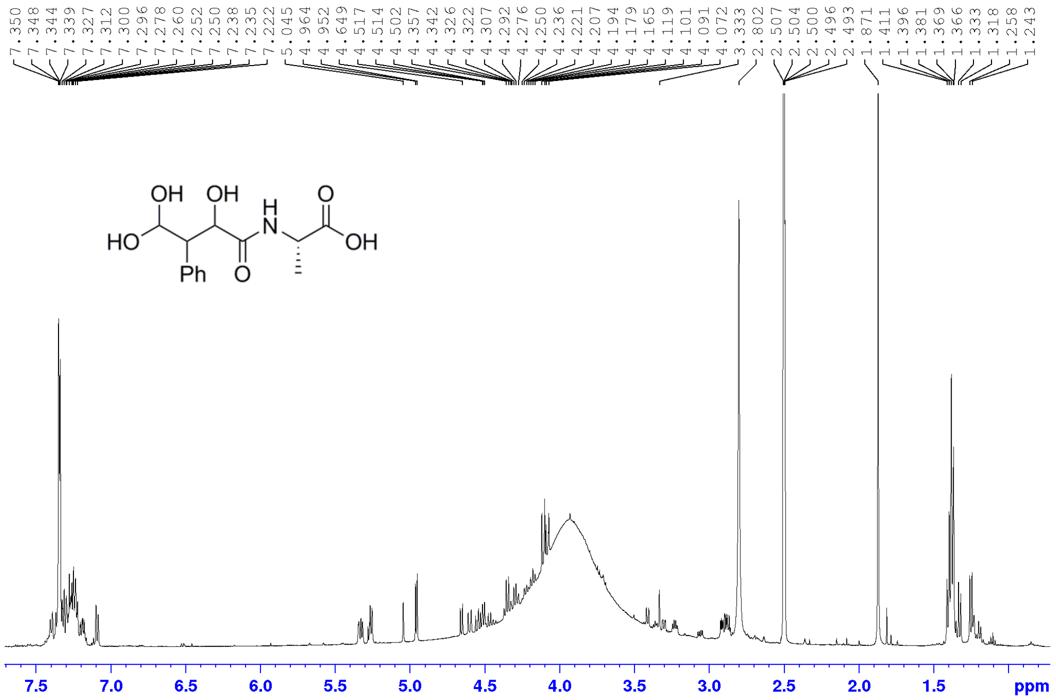
7.350 7.348 7.344 7.339 7.327 7.312 7.300 7.296 7.278 7.260 7.252 7.250 7.238 7.235 7.222 5.045 4.964 4.952 4.649 4.517 4.514 4.502 4.357 4.342 4.326 4.322 4.307 4.292 4.276 4.250 4.236 4.221 4.207 4.194 4.179 4.165 4.119 4.101 4.091 4.072 3.333 2.802 2.507 2.504 2.500 2.496 2.493 1.871 1.411 1.396 1.381 1.369 1.366 1.333 1.318 1.258 1.243
OH
OH
HO
Ph
O
H
N
O
OH
7.5
7.0
6.5
6.0
5.5
5.0
4.5
4.0
3.5
3.0
2.5
2.0
1.5
ppm

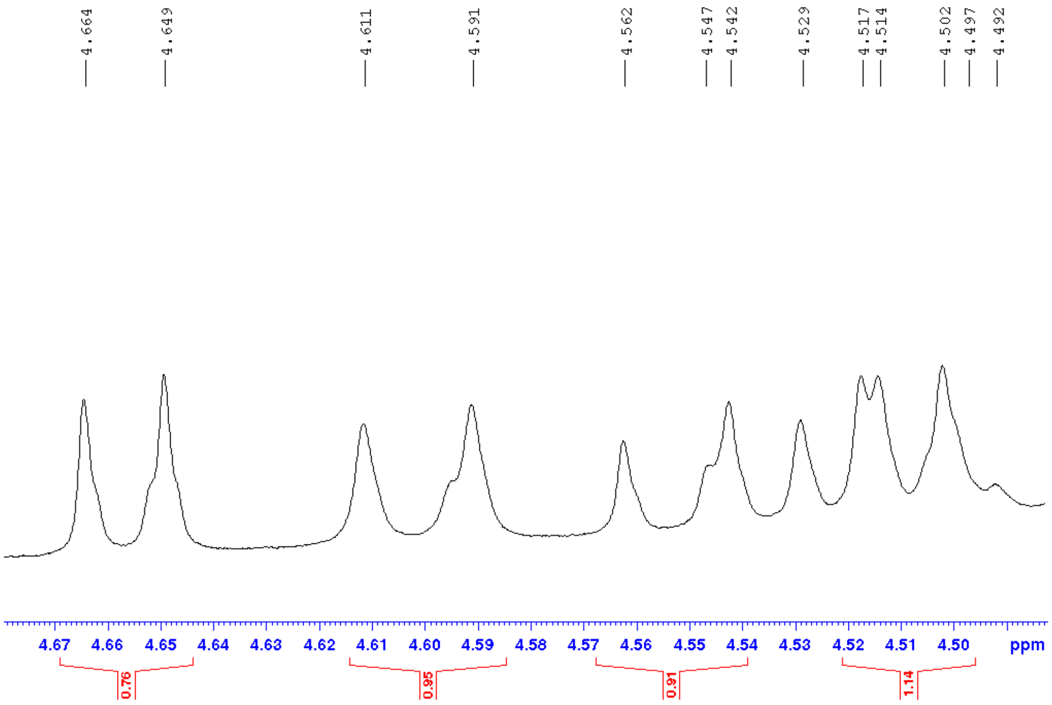
4.664 4.649 4.611 4.591 4.562 4.547 4.542 4.529 4.517 4.514 4.502 4.497 4.492
4.67 4.66 4.65 4.64 4.63 4.62 4.61 4.60 4.59 4.58 4.57 4.56 4.55 4.54 4.53 4.52 4.51 4.50 ppm
0.76
0.95
0.91
1.14

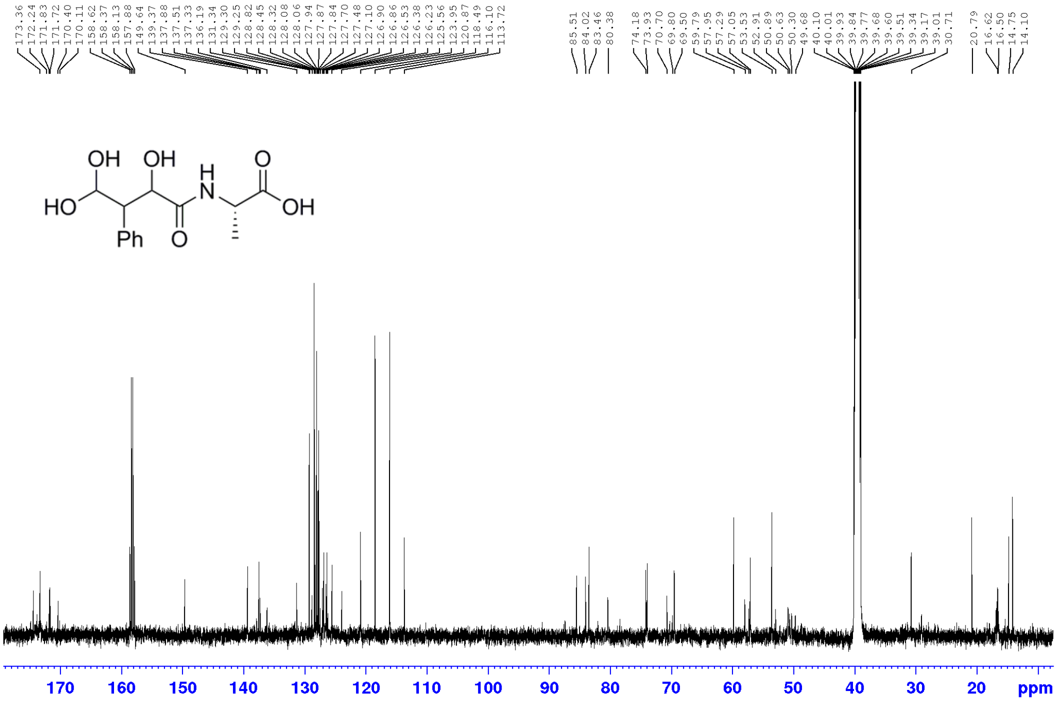
173.36
172.24
171.83
171.72
170.40
170.11
158.62
158.37
158.13
157.88
149.64
139.37
137.88
137.51
137.33
136.19
131.34
129.30
129.25
128.82
128.45
128.32
128.08
128.06
127.94
127.87
127.84
127.70
127.48
127.10
126.90
126.86
126.53
126.38
126.23
125.56
123.95
120.87
118.49
116.10
113.72
85.51
84.02
83.46
80.38
74.18
73.93
70.70
69.80
69.50
59.79
57.95
57.29
57.05
53.53
52.91
50.89
50.63
50.30
49.68
40.10
40.01
39.93
39.84
39.77
39.68
39.60
39.51
39.34
39.17
39.01
30.71
20.79
16.62
16.50
14.75
14.10
OH
OH
HO
H
N
O
OH
Ph
O
170
160
150
140
130
120
110
100
90
80
70
60
50
40
30
20
ppm

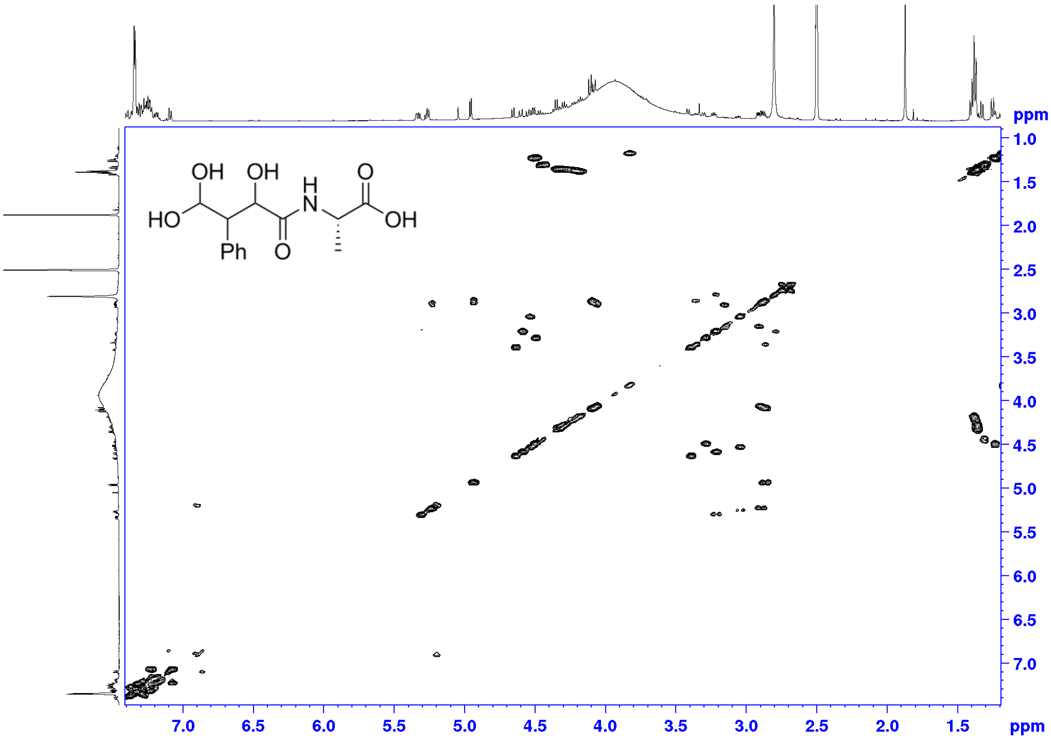
OH
OH
HO
H
N
O
OH
Ph
O
ppm
1.0
1.5
2.0
2.5
3.0
3.5
4.0
4.5
5.0
5.5
6.0
6.5
7.0
7.0
6.5
6.0
5.5
5.0
4.5
4.0
3.5
3.0
2.5
2.0
1.5
ppm

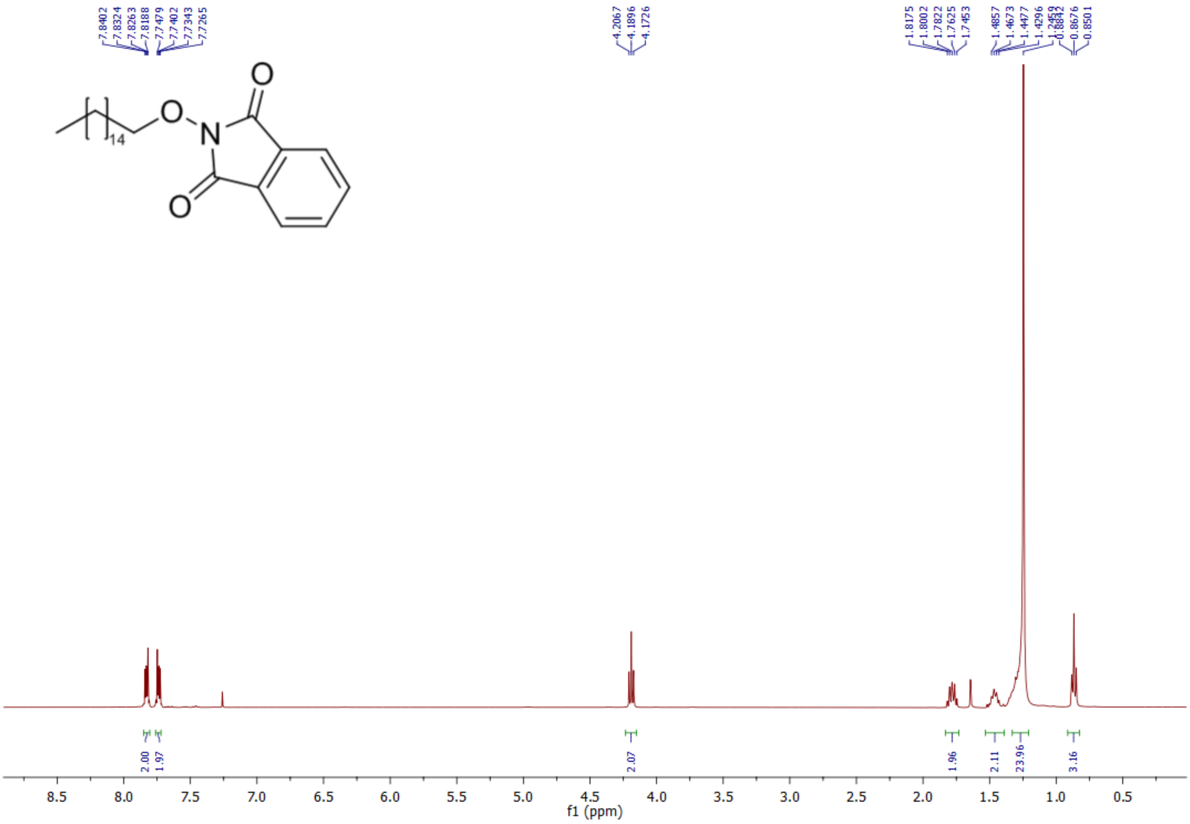

7.8402
7.8324
7.8263
7.8188
7.7479
7.7402
7.7343
7.7265
4.2067
4.1896
4.1726
1.8175
1.8002
1.7822
1.7625
1.7453
1.4857
1.4673
1.4477
1.4296
0.8676
0.8501
O
N
O
O
14
2.00
1.97
2.07
1.96
2.11
23.96
3.16
f1 (ppm)

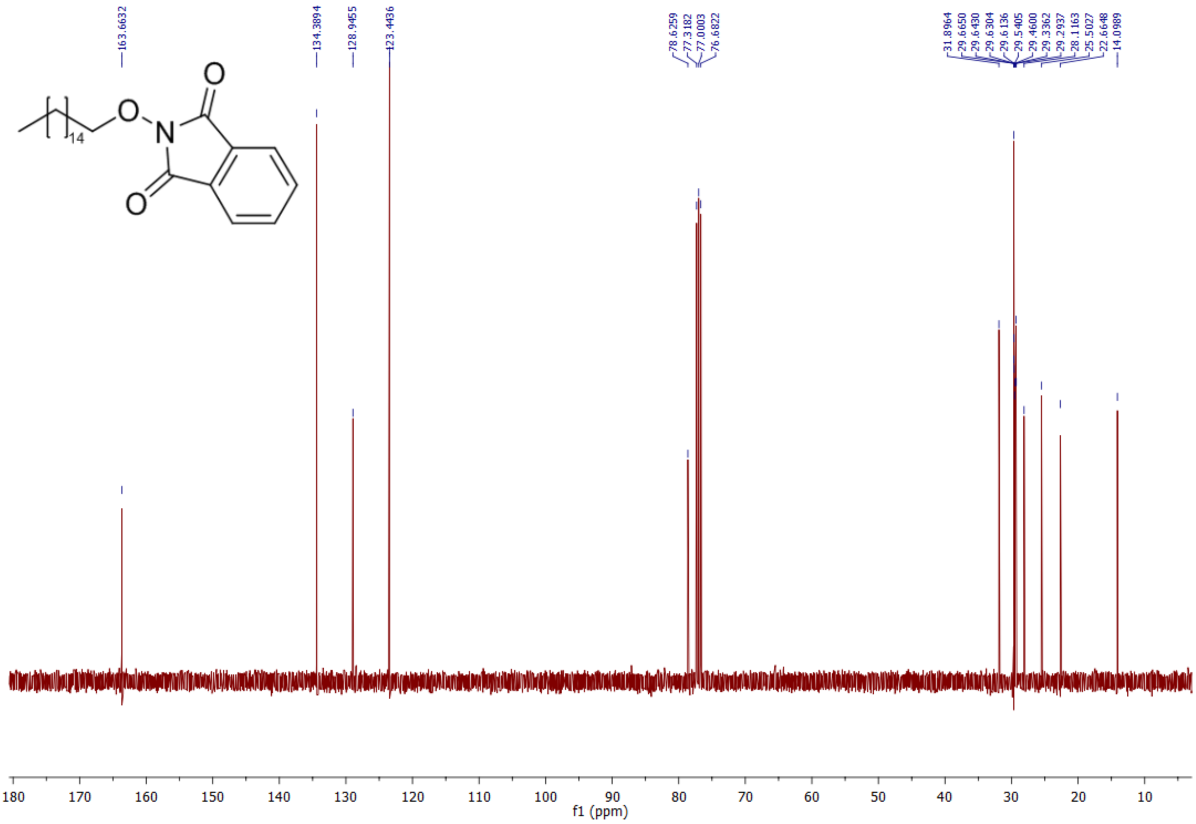

163.6632
134.3894
128.9455
123.4436
78.6259
77.3182
77.0003
76.6822
31.8964
29.6650
29.6430
29.6304
29.6136
29.5405
29.4600
29.3362
29.2937
28.1163
25.5027
22.6648
14.0989
O
N
O
O
14
f1 (ppm)

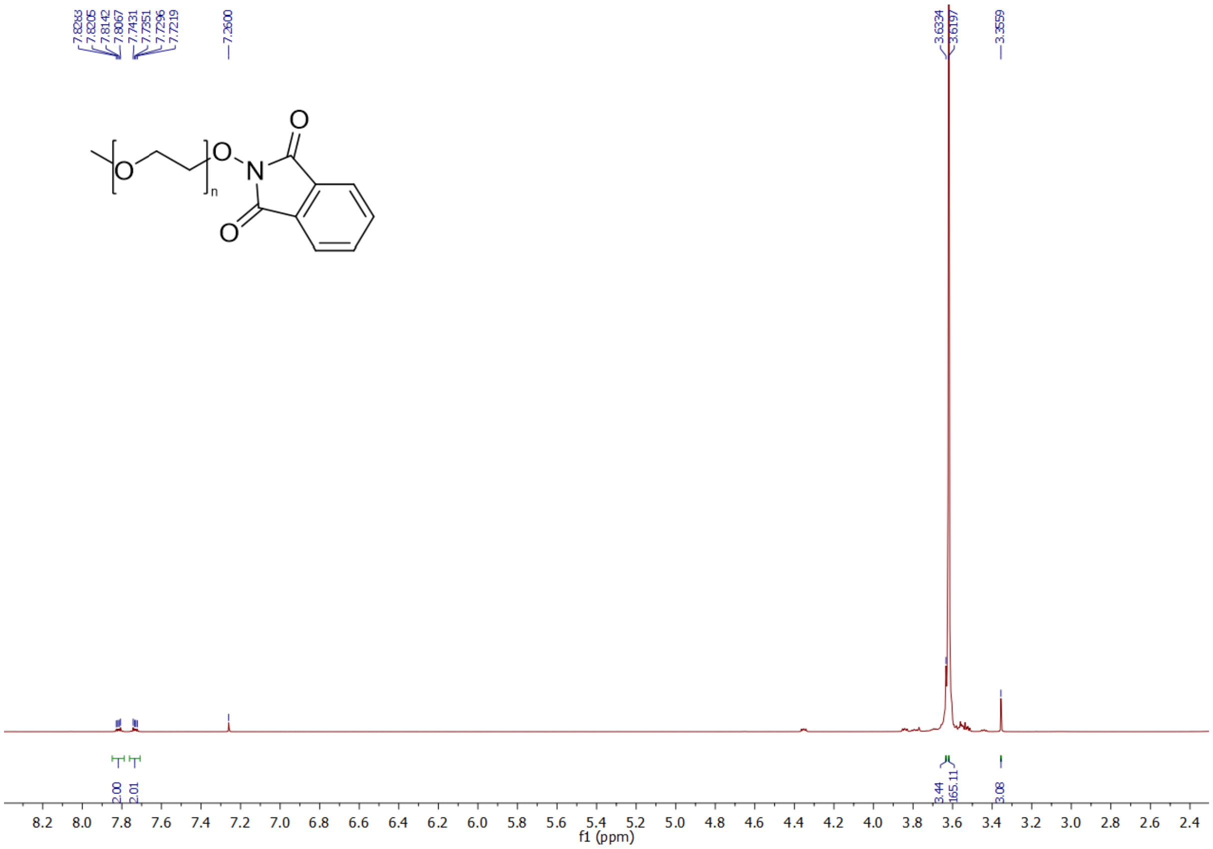
7.8283
7.8205
7.8142
7.8067
7.7431
7.7351
7.7296
7.7219
7.2600
3.6334
3.6197
3.3559
O
N
O
O
O
n
2.00
2.01
3.44
165.11
3.08
f1 (ppm)

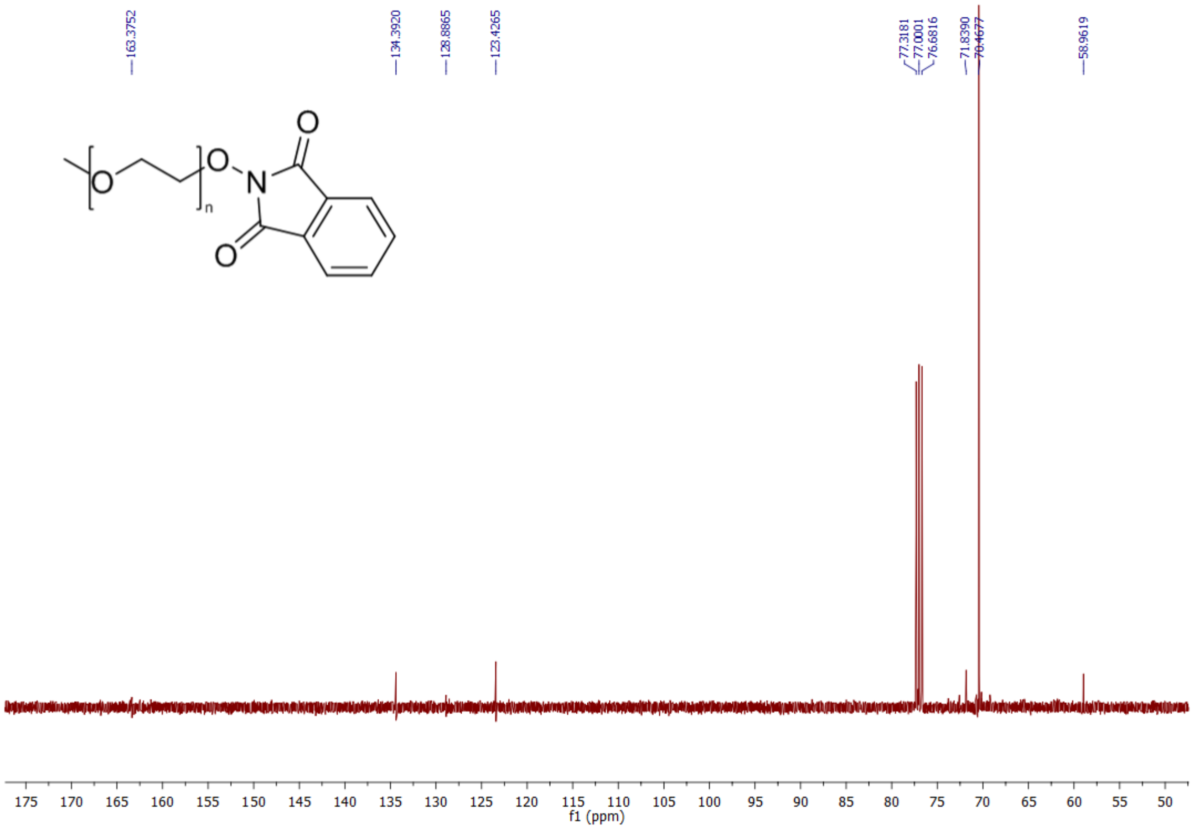
163.3752
134.3920
128.8865
123.4265
77.3181
77.0001
76.6816
71.8390
70.4677
58.9619
O
N
O
O
O
n
f1 (ppm)

12. References

1 Rasia, R. M., Brutscher, B. & Plevin, M. J. Selective Isotopic Unlabeling of Proteins Using Metabolic Precursors: Application to NMR Assignment of Intrinsically Disordered Proteins. *ChemBioChem* **13**, 732-739, doi:10.1002/cbic.201100678 (2012).

2 Zhang, C. *et al.* π-Clamp Mediated Cysteine Conjugation. *Nature Chem.* **8**, 120-128, doi:10.1038/nchem.2413 (2016).

3 Laemmli, U. K. Cleavage of Structural Proteins during the Assembly of the Head of Bacteriophage T4. *Nature* **227**, 680-685 (1970).

4 Brabham, R. L. *et al.* Palladium-unleashed proteins: gentle aldehyde decaging for site-selective protein modification. *Chem. Commun.* **54**, 1501-1504, doi:10.1039/C7CC07740H (2018).

5 Plass, T., Milles, S., Koehler, C., Schultz, C. & Lemke, E. A. Genetically Encoded Copper-Free Click Chemistry. *Angew. Chem. Int. Ed.* **50**, 3878-3881, doi:10.1002/anie.201008178 (2011).

6 Agarwal, P., van der Weijden, J., Sletten, E. M., Rabuka, D. & Bertozzi, C. R. A Pictet-Spengler ligation for protein chemical modification. *Proc. Natl. Acad. Sci. U. S. A.* **110**, 46-51, doi:10.1073/pnas.1213186110 (2013).

7 Kitov, P. I., Vinals, D. F., Ng, S., Tjhung, K. F. & Derda, R. Rapid, Hydrolytically Stable Modification of Aldehyde-Terminated Proteins and Phage Libraries. *J. Am. Chem. Soc.* **136**, 8149-8152, doi:10.1021/ja5023909 (2014).

8 Qi, X. *et al.* A solid-phase approach to DDB derivatives. *Eur. J. Med. Chem.* **40**, 805-810, doi:http://dx.doi.org/10.1016/j.ejmech.2005.03.024 (2005).

9 Schlick, T. L., Ding, Z., Kovacs, E. W. & Francis, M. B. Dual-Surface Modification of the Tobacco Mosaic Virus. *J. Am. Chem. Soc.* **127**, 3718-3723, doi:10.1021/ja046239n (2005).

10 Dirksen, A., Hackeng, T. M. & Dawson, P. E. Nucleophilic Catalysis of Oxime Ligation. *Angew. Chem. Int. Ed.* **45**, 7581-7584, doi:10.1002/anie.200602877 (2006).

11 Brannigan, J. A. *et al.* N-Myristoyltransferase from Leishmania donovani: Structural and Functional Characterisation of a Potential Drug Target for Visceral Leishmaniasis. *J. Mol. Biol.* **396**, 985-999, doi:https://doi.org/10.1016/j.jmb.2009.12.032 (2010).

12 Gilmore, J. M., Scheck, R. A., Esser-Kahn, A. P., Joshi, N. S. & Francis, M. B. N-Terminal Protein Modification through a Biomimetic Transamination Reaction. *Angew. Chem. Int. Ed.* **45**, 5307-5311, doi:10.1002/anie.200600368 (2006).
